# Supplementary material for: Female reproductive history in relation to chronic obstructive pulmonary disease and lung function in UK biobank: a prospective population-based cohort study
Source: BMJ Open. 2019 Oct 28;9(10):e030318. doi: 10.1136/bmjopen-2019-030318 (PMC6830692; doi:10.1136/bmjopen-2019-030318)
Supplement: Supplementary data [file bmjopen-2019-030318supp001.pdf]

Online Supplementary File

Table S1. Variables used in multiple imputation model

Variable	Type of variable	Regression model to predict missing in this variable	How variable was entered when used to predict missing in other variables
Incident COPD hospitalisation	Dichotomous	Logistic regression	1 indicator variable
Incident COPD death	Dichotomous	Logistic regression	1 indicator variable
FEV_1 z-score	Continuous	Linear regression	Continuous
FVC z-score	Continuous	Linear regression	Continuous
FEV_1/FVC z-score	Continuous	Linear regression	Continuous
Age at menarche, years	Categorical (3)	Ordered logistic regression	2 indicator variables
Menopause status	Categorical (3)	Polytomous (unordered) regression	2 indicator variables
Age at menopause, years	Categorical (4)	Ordered logistic regression	3 indicator variables
Parity	Categorical (5)	Ordered logistic regression	4 indicator variables
History of ovarian cysts or polycystic ovary syndrome	Dichotomous	Logistic regression	Dichotomous
History of endometriosis	Dichotomous	Logistic regression	Dichotomous
Oral contraception ever use	Dichotomous	Passively imputed using years of OC use	N/A
Years of oral contraception	Categorical (6)	Ordered logistic regression	5 indicator variables
Gynaecological surgery	Categorical (4)	Polytomous (unordered) regression	3 indicator variables
HRT ever use	Dichotomous	Passively imputed using years of HRT use	N/A

Tang R, *et al. BMJ Open* 2019; 9:e030318. doi: 10.1136/bmjopen-2019-030318

Years of HRT use	Categorical (6)	Polytomous (unordered) regression	5 indicator variables
Age, years	Continuous	Linear regression	Continuous
Height, cm	Continuous	Linear regression	Continuous
Log body mass index	Continuous	Linear regression	Continuous
Ethnicity	Dichotomous	Logistic regression	Dichotomous
Qualifications	Categorical (6)	Polytomous (unordered) regression	6 indicator variables
Income	Categorical (6)	Polytomous (unordered) regression	5 indicator variables
Townsend deprivation index	Continuous	Linear regression	Continuous
Smoking pack-years	Categorical (5)	Ordered logistic regression	4 indicator variables
Smoking duration, years	Categorical (5)	Ordered logistic regression	4 indicator variables
Smoking intensity, cigarettes/day	Continuous	Linear regression	Continuous
Smoking status	Dichotomous	Logistic regression	Dichotomous
Maternal history of COPD	Dichotomous	Logistic regression	Dichotomous
Paternal history of COPD	Dichotomous	Logistic regression	Dichotomous
History of asthma	Dichotomous	Logistic regression	Dichotomous
History of pneumonia	Dichotomous	Logistic regression	Dichotomous
History of tuberculosis	Dichotomous	Logistic regression	Dichotomous
History of cardiovascular disease	Dichotomous	Logistic regression	Dichotomous
History of diabetes	Dichotomous	Logistic regression	Dichotomous

COPD, chronic obstructive pulmonary disease; FEV_1, forced expiratory volume in one-second; FVC, forced vital capacity; HRT, hormone replacement therapy

Tang R, *et al. BMJ Open* 2019; 9:e030318. doi: 10.1136/bmjopen-2019-030318

Table S2. Characteristics in UK Biobank women with and without spirometry data

Characteristic	Mean (SE) or % in women with spirometry	Mean (SE) or % in women without spirometry	*P* value
N	195 824	77 617	
Age (years)	56.18 (0.02)	56.78 (0.03)	<0.001
<55	20.9	20.3	
55-59	23.0	20.2	
60-64	30.5	29.7	
65 or higher	25.6	29.7	
Missing	0	0	
Height (cm)	162.6 (0.01)	162.0 (0.02)	<0.001
Missing	0.1	2.5	
Body mass index (kg/m^2)			<0.001
<18.5	0.6	1.2	
18.5-24.9	38.3	36.1	
25.0-29.9	37.5	35.7	
>29.9	23.4	25.3	
Missing	0.2	1.6	
Ethnicity			<0.001
White	95.0	92.2	
Other	4.7	7.1	
Missing	0.3	0.8	
Education			<0.001
College, university or other professional degree	37.9	34.4	
A levels, AS levels or equivalent	12.2	10.6	
O levels, GCSEs or equivalent	23.5	22.7	
CSE or equivalent	5.3	5.6	
NVQ, HND, HNC or equivalent	4.4	4.8	
Other	15.7	20.1	
Missing	1.0	1.8	
Annual household income (£)			<0.001
<18 000	19.2	23.4	

>18 000 and <31 000	21.7	21.6	
>31 000 and <52 000	21.7	19.0	
>52 000 and <100 000	16.3	12.8	
>100 000	4.3	3.0	
Do not know/prefer not to answer	16.4	19.5	
Missing	0.3	0.7	
Townsend deprivation index	-1.41 (0.01)	-1.14 (0.01)	<0.001
Missing	0.1	0.1	
Smoking history (pack-years)			<0.001
None	58.4	61.4	
<11	8.3	6.8	
11-20	7.7	6.7	
21-30	5.4	4.9	
>30	5.7	6.2	
Missing	14.5	14.0	
Incident COPD hospitalisation/death	0.3	0.6	<0.001
Missing	0.6	1.2	
Prevalent COPD at baseline	0.6	1.2	<0.001
Missing	<0.1	0	
Asthma			<0.001
No	86.9	87.6	
Yes	13.1	12.4	
Missing	<0.1	0	
Maternal history of COPD			<0.001
No	90.3	89.4	
Yes	6.0	5.8	
Don't know	2.0	2.4	
Missing	1.7	2.4	
Paternal history of COPD			<0.001
No	82.2	80.6	
Yes	10.2	10.3	
Don't know	4.8	5.5	
Missing	2.8	3.6	
Age at menarche			<0.001

Tang R, *et al. BMJ Open* 2019; 9:e030318. doi: 10.1136/bmjopen-2019-030318

<12	19.3	19.5	
12-15	72.2	70.3	
>15	5.5	6.2	
Missing	3.0	4.0	
Menopause status			<0.001
No	24.0	22.1	
Yes	60.3	61.0	
Missing	15.7	16.9	
Age at natural menopause			<0.001
<47	11.5	12.6	
47-49	9.4	9.4	
50-52	21.4	20.9	
>52	17.1	16.9	
Did not undergo natural menopause	35.6	34.6	
Missing	4.9	5.7	
Parity, binary			<0.001
Nulliparous	14.5	15.4	
Parous	83.8	82.3	
Parity, ordinal (5)			<0.001
0	14.5	15.4	
1	11.9	11.9	
2	34.1	32.7	
3	25.1	24.1	
>3	12.7	13.5	
Missing	1.7	2.4	
PCOS/ovarian cysts	1.7	1.7	0.60
Missing	0	0	
Endometriosis	1.5	1.5	0.81
Missing	0	0	
Oral contraception ever used	81.9	77.4	<0.001
Years of oral contraception use			<0.001
0	17.7	21.6	
1	10.8	11.6	

2-4	18.6	18.6	
5-9	18.1	16.5	
10-15	17.9	16.2	
>15	16.5	14.6	
Missing	0.3	1.0	
HRT ever used	37.8	38.7	<0.001
Years of HRT			<0.001
0	61.9	60.3	
1-2	10.5	11.1	
3-5	8.3	8.6	
6-10	5.7	5.5	
>10	8.2	8.2	
Missing	5.5	6.3	
Gynaecological surgery			<0.001
No	81.2	78.5	
Hysterectomy	9.6	10.5	
Bilateral oophorectomy	0.4	0.4	
Both	7.3	8.2	
Missing	1.5	2.4	

SE, standard error; A levels, Advanced levels; AS levels, Advanced Subsidiary levels; O levels, Ordinary levels; GCSE, General Certificate of Secondary Educations; CSE, certificate of secondary education; NVQ, national vocational qualifications; HND, higher national diploma; HNC, higher national certificate; COPD, chronic obstructive pulmonary disease; PCOS, polycystic ovary syndrome; HRT, hormone replacement therapy

Tang R, *et al. BMJ Open* 2019; 9:e030318. doi: 10.1136/bmjopen-2019-030318

Table S3. Cox regression analyses of female reproductive health indicators with incident COPD-related hospitalisation/death during follow-up in **women with FEV_1/FVC ≥ 0.7 and without a history of respiratory illness**[a] (N=217 512)

Reproductive health indicator	% of participants	Person-years of follow-up	Number of COPD events	Age-adjusted Hazard ratio	Age-adjusted 95% CI	Multiple-adjusted[b] Hazard ratio	Multiple-adjusted[b] 95% CI
Age at menarche							
<12 years	19.8	259 259	97	1.39	1.10, 1.76	1.27	1.00, 1.62
12-15 years	74.4	975 565	259	1.0	NA	1.0	NA
>15 years	5.8	75 410	36	1.84	1.29, 2.62	1.53	1.08, 2.19
Menopause status							
No	27.2	359 354	34	1.0	NA	1.0	NA
Yes	72.8	950 881	358	1.09	1.07, 1.12	0.88	0.56, 1.39
Age at natural menopause[c]							
<47 years	19.8	157 942	90	2.25	1.60, 3.15	1.59	1.14, 2.23
47-49 years	15.9	127 134	50	1.67	1.12, 2.47	1.40	0.94, 2.08
50-52 years	35.7	285 678	74	1.0	NA	1.0	NA
>52 years	28.6	228 061	57	0.89	0.61, 1.30	0.97	0.67, 1.42
Parity, binary							
Nulliparous	15.3	199 136	43	0.84	0.60, 1.15	0.93	0.67, 1.29
Parous	84.7	1 111 099	349	1.0	NA	1.0	NA
Parity, ordered categories							
0	15.3	199 136	43	1.0	NA	1.0	NA
1	12.2	159 386	49	1.37	0.91, 2.07	1.10	0.72, 1.66

2	34.7	455 068	115	0.95	0.67, 1.36	0.97	0.68, 1.39
3	25.2	330 200	98	1.12	0.77, 1.61	1.03	0.71, 1.49
>3	12.7	166 444	87	1.86	1.28, 2.70	1.34	0.92, 1.95
PCOS/ovarian cysts							
No	98.3	1 288 468	378	1.0	NA	1.0	NA
Yes	1.7	21 767	14	2.20	1.29, 3.76	2.23	1.31, 3.81
Endometriosis							
No	98.5	1 291 165	387	1.0	NA	1.0	NA
Yes	1.5	459 189	5	1.07	0.44, 2.58	1.09	0.45, 2.65
Oral contraception							
Never used	18.9	247 243	117	1.0	NA	1.0	NA
Ever used	81.1	1 062 992	275	0.81	0.65, 1.01	0.72	0.57, 0.91
Years of oral contraception							
0	18.9	247 243	117	1.0	NA	1.0	NA
1	10.9	143 083	41	0.83	0.58, 1.18	0.66	0.46, 0.94
2-4	18.6	244 583	86	0.94	0.71, 1.25	0.88	0.66, 1.17
5-9	17.8	234 184	57	0.75	0.54, 1.03	0.70	0.50, 0.97
10-15	17.6	230 642	51	0.74	0.53, 1.04	0.65	0.46, 0.92
>15	16.1	210 498	40	0.71	0.49, 1.03	0.62	0.43, 0.90
HRT							
Never used	65.0	851 046	181	1.0	NA	1.0	NA
Ever used	35.0	459 189	211	1.41	1.14, 1.74	1.11	0.90, 1.38

Years of HRT							
0	65.0	851 046	181	1.0	NA	1.0	NA
1-2	11.2	146 764	83	1.81	1.37, 2.38	1.33	1.01, 1.75
3-5	8.8	116 043	51	1.43	1.03, 1.98	1.07	0.77, 1.49
6-10	6.1	80 081	25	0.97	0.63, 1.50	0.85	0.55, 1.32
>10	8.8	116 300	52	1.21	0.87, 1.69	1.05	0.75, 1.46
Gynaecological surgery							
No	82.3	1 078 722	263	1.0	NA	1.0	NA
Hysterectomy	9.7	127 997	73	1.75	1.34, 2.29	1.54	1.18, 2.00
Bilateral oophorectomy	0.4	4749	4	2.86	1.06, 7.68	2.49	0.92, 6.70
Both	7.6	98 766	52	1.62	1.19, 2.19	1.39	1.03, 1.89

COPD, chronic obstructive pulmonary disease; FEV_1, forced expiratory volume in one-second; FVC, forced vital capacity; CI, confidence interval; PCOS, polycystic ovary syndrome; HRT, hormone replacement therapy; NA, not applicable

[a]Respiratory illnesses include COPD, asthma, tuberculosis or pneumonia reported at baseline

[b]Multiple-adjusted for age, height, BMI (log-transformed), ethnicity, education, household income, Townsend deprivation index, smoking history in pack-years, maternal COPD and paternal COPD

[c]Age at menopause was analysed only among women who experienced natural menopause before baseline (n=127 270)

Tang R, *et al. BMJ Open* 2019; 9:e030318. doi: 10.1136/bmjopen-2019-030318

Table S4. Cox regression analyses of female reproductive health indicators with incident COPD-related hospitalisation/death during follow-up, **further adjusting for smoking status or smoking duration and intensity** (N=271 271)

Reproductive health indicator	% of participants	Person-years of follow-up	Number of COPD events	Multiple-adjusted[a]		Smoking duration and intensity-adjusted[b]		Smoking status-adjusted[c]	
				Hazard ratio	95% CI	Hazard ratio	95% CI	Hazard ratio	95% CI
Age at menarche									
<12 years	20.0	326 027	275	1.15	1.00, 1.32	1.18	1.03, 1.36	1.17	1.01, 1.34
12-15 years	74.1	1 210 146	768	1.0	NA	1.0	NA	1.0	NA
>15 years	5.9	95 771	95	1.37	1.11, 1.71	1.33	1.07, 1.65	1.32	1.06, 1.64
Menopause status									
No	26.6	437 890	97	1.0	NA	1.0	NA	1.0	NA
Yes	73.4	1 194 055	1041	1.07	0.82, 1.41	1.06	0.81, 1.39	1.03	0.78, 1.36
Age at natural menopause[d]				1.44	1.19, 1.75	1.47	1.21, 1.79	1.43	1.18, 1.74
<47 years	20.3	202 811	270	1.25	1.00, 1.57	1.25	1.00, 1.57	1.24	0.99, 1.56
47-49 years	15.8	158 718	140	1.0	NA	1.0	NA	1.0	NA
50-52 years	35.4	354 774	219	0.93	0.75, 1.15	0.91	0.74, 1.13	0.94	0.76, 1.16
>52 years	28.4	283 721	161						
Parity, binary									
Nulliparous	15.1	244 938	116	0.92	0.76, 1.12	0.90	0.74, 1.09	0.92	0.75, 1.12
Parous	84.9	1 387 009	1022	1.0	NA	1.0	NA	1.0	NA

Parity, ordered categories									
0	15.1	244 938	116	1.0	NA	1.0	NA	1.0	NA
1	12.2	198 124	139	1.06	0.82, 1.35	1.10	0.86, 1.41	1.06	0.82, 1.36
2	34.4	562 339	308	0.92	0.74, 1.14	0.93	0.75, 1.16	0.93	0.75, 1.15
3	25.3	413 347	293	1.06	0.85, 1.33	1.10	0.88, 1.37	1.07	0.86, 1.34
>3	13.1	213 195	282	1.45	1.16, 1.82	1.50	1.20, 1.88	1.43	1.15, 1.79
PCOS/ovarian cysts									
No	98.3	1 604 641	1108	1.0	NA	1.0	NA	1.0	NA
Yes	1.7	27 304	30	1.61	1.12, 2.32	1.66	1.16, 2.39	1.65	1.14, 2.37
Endometriosis									
No	98.5	1 607 817	1127	1.0	NA	1.0	NA	1.0	NA
Yes	1.5	24 131	11	0.85	0.47, 1.53	0.84	0.46, 1.52	0.84	0.46, 1.53
Oral contraception									
Never used	18.9	308 641	295	1.0	NA	1.0	NA	1.0	NA
Ever used	81.1	1 323 303	843	0.85	0.74, 0.97	0.90	0.78, 1.03	0.87	0.75, 1.00
Years of oral									
contraception use	18.9	308 641	295	1.0	NA	1.0	NA	1.0	NA
0	11.1	180 195	137	0.84	0.69, 1.04	0.89	0.72, 1.09	0.86	0.70, 1.06
1	18.7	306 053	246	0.95	0.80, 1.13	1.00	0.84, 1.19	0.98	0.82, 1.16
2-4	17.8	290 554	181	0.86	0.71, 1.05	0.92	0.76, 1.11	0.87	0.72, 1.06
5-9	17.5	285 766	153	0.74	0.61, 0.91	0.80	0.66, 0.98	0.77	0.63, 0.94
10-15	16.1	260 734	126	0.75	0.60, 0.93	0.80	0.64, 1.00	0.77	0.62, 0.96

Tang R, *et al. BMJ Open* 2019; 9:e030318. doi: 10.1136/bmjopen-2019-030318

>15									
HRT									
Never used	63.9	1 042 083	510	1.0	NA	1.0	NA	1.0	NA
Ever used	36.1	589 861	628	1.15	1.01, 1.30	1.20	1.06, 1.36	1.16	1.03, 1.32
Years of HRT									
0	63.9	1 042 083	510	1.0	NA	1.0	NA	1.0	NA
1-2	11.7	190 902	240	1.33	1.12, 1.57	1.39	1.17, 1.65	1.36	1.14, 1.61
3-5	9.1	147 962	162	1.17	0.97, 1.42	1.23	1.01, 1.49	1.17	0.96, 1.42
6-10	6.2	100 921	74	0.87	0.66, 1.14	0.90	0.69, 1.18	0.88	0.67, 1.15
>10	9.2	150 077	152	1.06	0.87, 1.29	1.12	0.92, 1.36	1.07	0.88, 1.31
Gynaecological surgery									
No	81.8	1 335 512	765	1.0	NA	1.0	NA	1.0	NA
Hysterectomy	10.1	164 869	213	1.49	1.28, 1.74	1.49	1.27, 1.74	1.47	1.26, 1.72
Bilateral oophorectomy	0.4	5957	6	1.36	0.61, 3.04	1.34	0.60, 3.00	1.38	0.62, 3.08
Both	7.8	125 607	154	1.42	1.19, 1.69	1.41	1.18, 1.68	1.39	1.17, 1.66

COPD, chronic obstructive pulmonary disease; CI, confidence interval; PCOS, polycystic ovary syndrome; HRT, hormone replacement

[a]Multiple-adjusted for age, height, BMI (log-transformed), ethnicity, education, household income, Townsend deprivation index, smoking history in pack years, maternal COPD and paternal COPD

[b]Multiple-adjusted with smoking history as two covariates (duration in years and intensity in average number of cigarettes/day) rather than smoking pack-years

[c]Further adjusted from the multiple-adjusted analyses[a] for smoking status at baseline

[d]Age at menopause was analysed only among women who experienced natural menopause before baseline (n=159 571)

Tang R, *et al. BMJ Open* 2019; 9:e030318. doi: 10.1136/bmjopen-2019-030318

Table S5. Cox regression analyses of female reproductive health indicators with incident COPD-related hospitalisation/death during follow-up in **never smokers** (N=161 626)

**Reproductive health indicator**	**% of participants**	**Person-years of follow-up**	**Number of COPD events**	**Age-adjusted**		**Multiple-adjusted[a]**	
				**Hazard ratio**	**95% CI**	**Hazard ratio**	**95% CI**
Age at menarche							
<12 years	19.9	193 712	59	1.63	1.20, 2.22	1.39	1.02, 1.90
12-15 years	74.4	725 115	134	1.0	NA	1.0	NA
>15 years	5.7	55 624	18	1.82	1.11, 2.98	1.76	1.07, 2.89
Menopause status							
No	30.4	252 038	40	1.0	NA	1.0	NA
Yes	69.6	571 767	181	0.99	0.55, 1.77	0.98	0.55, 1.76
Age at natural menopause[b]							
<47 years	18.7	108 538	37	1.66	1.05, 2.64	1.53	0.96, 2.43
47-49 years	15.5	90 293	18	1.05	0.58, 1.91	1.03	0.57, 1.86
50-52 years	36.1	210 322	44	1.0	NA	1.0	NA
>52 years	29.7	172 378	33	0.85	0.54, 1.35	0.83	0.52, 1.32
Parity, binary							
Nulliparous	16.3	157 900	27	0.92	0.61, 1.38	0.94	0.62, 1.42
Parous	83.7	816 551	184	1.0	NA	1.0	NA
Parity, ordered categories							
0	16.3	157 900	27	1.0	NA	1.0	NA
1	11.2	109 141	24	1.21	0.70, 2.11	1.15	0.66, 2.00

2	35.2	343 543	69	0.97	0.62, 1.52	0.98	0.62, 1.54
3	24.8	242 317	60	1.17	0.74, 1.85	1.16	0.73, 1.84
>3	12.5	121 548	31	1.17	0.70, 1.96	1.03	0.61, 1.74
PCOS/ovarian cysts							
No	98.3	958 600	205	1.0	NA	1.0	NA
Yes	1.7	15 851	6	1.77	0.79, 3.99	1.73	0.77, 3.91
Endometriosis							
No	98.5	950 101	208	1.0	NA	1.0	NA
Yes	1.5	14 350	3	1.15	0.37, 3.61	1.10	0.35, 3.46
Oral contraception							
Never used	21.6	210 051	71	1.0	NA	1.0	NA
Ever used	78.4	764 401	140	0.74	0.55, 1.00	0.79	0.58, 1.06
Years of oral contraception use							
0	21.6	210 051	71	1.0	NA	1.0	NA
1	10.9	106 163	19	0.65	0.39, 1.10	0.67	0.40, 1.12
2-4	18.9	184 203	48	0.94	0.65, 1.36	0.97	0.67, 1.40
5-9	17.2	167 797	32	0.77	0.50, 1.17	0.82	0.53, 1.26
10-15	16.3	159 341	25	0.67	0.42, 1.07	0.73	0.45, 1.17
>15	15.1	146 894	16	0.50	0.29, 0.88	0.56	0.32, 0.97
HRT							
Never used	65.7	639 250	106	1.0	NA	1.0	NA
Ever used	34.3	335 202	105	1.25	0.94, 1.67	1.22	0.92, 1.62

Tang R, *et al. BMJ Open* 2019; 9:e030318. doi: 10.1136/bmjopen-2019-030318

Years of HRT							
0	65.7	639 250	106	1.0	NA	1.0	NA
1-2	9.6	93 783	39	1.79	1.23, 2.61	1.74	1.19, 2.53
3-5	7.6	74 345	17	0.99	0.59, 1.66	0.91	0.54, 1.53
6-10	5.3	51 998	15	1.16	0.67, 2.01	1.15	0.67, 1.99
>10	11.8	73 060	34	1.00	0.61, 1.64	1.02	0.61, 1.91
Gynaecological surgery							
No	82.2	801 428	136	1.0	NA	1.0	NA
Hysterectomy	9.9	96 682	40	1.92	1.34, 2.76	1.66	1.15, 2.39
Bilateral oophorectomy	0.4	3385	1	1.49	0.21, 10.63	1.47	0.21, 10.50
Both	7.5	72 956	34	2.12	1.45, 3.12	1.85	1.26, 2.72

COPD, chronic obstructive pulmonary disease; CI, confidence interval; PCOS, polycystic ovary syndrome; HRT, hormone replacement

[a]Multiple-adjusted for age, height, BMI (log-transformed), ethnicity, education, household income, Townsend deprivation index, maternal COPD and paternal COPD

[b]Age at menopause analysed only among women who experienced natural menopause before baseline (n=92 561)

Tang R, *et al*. *BMJ Open* 2019; 9:e030318. doi: 10.1136/bmjopen-2019-030318

Table S6. Cox regression analyses of female reproductive health indicators with incident COPD-related hospitalisation/death during follow-up, **further adjusting for comorbidities** (N=271 271)

Reproductive health indicator	% of participants	Person-years of follow-up	Number of COPD events	Multiple-adjusted[a]		Comorbidity-adjusted[c]	
				Hazard ratio	95% CI	Hazard ratio	95% CI
Age at menarche							
<12 years	20.0	326 027	275	1.15	1.00, 1.32	1.17	1.02, 1.35
12-15 years	74.1	1 210 146	768	1.0	NA	1.0	NA
>15 years	5.9	95 771	95	1.37	1.11, 1.71	1.32	1.06, 1.64
Menopause status							
No	26.6	437 890	97	1.0	NA	1.0	NA
Yes	73.4	1 194 055	1041	1.07	0.82, 1.41	1.06	0.81, 1.39
Age at natural menopause[d]							
<47 years	20.3	202 811	270	1.44	1.19, 1.75	1.46	1.21, 1.78
47-49 years	15.8	158 718	140	1.25	1.00, 1.57	1.26	1.01, 1.58
50-52 years	35.4	354 774	219	1.0	NA	1.0	NA
>52 years	28.4	283 721	161	0.93	0.75, 1.15	0.91	0.74, 1.13
Parity, binary							
Nulliparous	15.1	244 938	116	0.92	0.76, 1.12	0.90	0.74, 1.09
Parous	84.9	1 387 009	1022	1.0	NA	1.0	NA
Parity, ordered categories							
0	15.1	244 938	116	1.0	NA	1.0	NA
1	12.2	198 124	139	1.06	0.82, 1.35	1.10	0.86, 1.41

2	34.4	562 339	308	0.92	0.74, 1.14	0.93	0.75, 1.16
3	25.3	413 347	293	1.06	0.85, 1.33	1.10	0.88, 1.37
>3	13.1	213 195	282	1.45	1.16, 1.82	1.50	1.20, 1.87
PCOS/ovarian cysts							
No	98.3	1 604 641	1108	1.0	NA	1.0	NA
Yes	1.7	27 304	30	1.61	1.12, 2.32	1.64	1.14, 2.36
Endometriosis							
No	98.5	1 607 817	1127	1.0	NA	1.0	NA
Yes	1.5	24 131	11	0.85	0.47, 1.53	0.84	0.46, 1.53
Oral contraception							
Never used	18.9	308 641	295	1.0	NA	1.0	NA
Ever used	81.1	1 323 303	843	0.85	0.74, 0.97	0.91	0.79, 1.04
Years of oral contraception use							
0	18.9	308 641	295	1.0	NA	1.0	NA
1	11.1	180 195	137	0.84	0.69, 1.04	0.89	0.73, 1.10
2-4	18.7	306 053	246	0.95	0.80, 1.13	1.01	0.85, 1.20
5-9	17.8	290 554	181	0.86	0.71, 1.05	0.93	0.76, 1.12
10-15	17.5	285 766	153	0.74	0.61, 0.91	0.81	0.66, 0.99
>15	16.1	260 734	126	0.75	0.60, 0.93	0.81	0.65, 1.01
HRT							
Never used	63.9	1 042 083	510	1.0	NA	1.0	NA
Ever used	36.1	589 861	628	1.15	1.01, 1.30	1.20	1.05, 1.35

Tang R, *et al. BMJ Open* 2019; 9:e030318. doi: 10.1136/bmjopen-2019-030318

Years of HRT							
0	63.9	1 042 083	510	1.0	NA	1.0	NA
1-2	11.7	190 902	240	1.33	1.12, 1.57	1.38	1.16, 1.63
3-5	9.1	147 962	162	1.17	0.97, 1.42	1.22	1.01, 1.48
6-10	6.2	100 921	74	0.87	0.66, 1.14	0.90	0.69, 1.17
>10	9.2	150 077	152	1.06	0.87, 1.29	1.11	0.91, 1.35
Gynaecological surgery							
No	81.8	1 335 512	765	1.0	NA	1.0	NA
Hysterectomy	10.1	164 869	213	1.49	1.28, 1.74	1.47	1.26, 1.72
Bilateral oophorectomy	0.4	5957	6	1.36	0.61, 3.04	1.34	0.60, 2.99
Both	7.8	125 607	154	1.42	1.19, 1.69	1.39	1.17, 1.66

COPD, chronic obstructive pulmonary disease; CI, confidence interval; PCOS, polycystic ovary syndrome; HRT, hormone replacement

[a]Multiple-adjusted for age, height, BMI (log-transformed), ethnicity, education, household income, Townsend deprivation index, smoking history in pack years, maternal COPD and paternal COPD

[b]Further adjusted from the multiple-adjusted analyses[a] for a history of cardiovascular disease and/or diabetes at baseline

[c]Analysed in women who experienced natural menopause before baseline (n=159 571)

Table S7. Linear regression analyses of female reproductive health indicators with baseline spirometry measures, **further adjusting for smoking status or smoking duration and intensity** (N=273 441)

**Reproductive health indicator**	**Mean change in FEV_1 z-score (95% CI)**		**Mean change in FVC z-score (95% CI)**		**Mean change in FEV_1/FVC z-score (95% CI)**	
	**Smoking duration and intensity-adjusted[a]**	**Smoking status-adjusted[b]**	**Smoking duration and intensity-adjusted[a]**	**Smoking status-adjusted[b]**	**Smoking duration and intensity-adjusted [a]**	**Smoking status-adjusted [b]**
Age at menarche						
<12 years	-0.01 (-0.02, 0.00)	-0.01 (-0.02, 0.00)	-0.01 (-0.02, 0.01)	-0.01 (-0.02, 0.01)	-0.02 (-0.03, -0.01)	-0.01 (-0.03, 0.00)
12-15 years	0	0	0	0	0	0
>15 years	-0.03 (-0.05, -0.01)	-0.03 (-0.05, -0.01)	-0.03 (-0.05, -0.02)	-0.03 (-0.05, -0.01)	0.00 (-0.02, 0.02)	0.00 (-0.02, 0.02)
Menopause status						
No	0	0	0	0	0	0
Yes	-0.04 (-0.05, -0.02)	-0.03 (-0.05, -0.02)	-0.04 (-0.05, -0.02)	-0.04 (-0.05, -0.02)	0.00 (-0.02, 0.01)	0.01 (0.00, 0.03)
Age at natural menopause[c]						
<47 years	-0.04 (-0.05, -0.02)	-0.03 (-0.05, -0.02)	-0.04 (-0.05, -0.02)	-0.04 (-0.05, -0.02)	-0.01 (-0.03, 0.00)	0.00 (-0.02, 0.01)
47-49 years	-0.02 (-0.04, 0.00)	-0.02 (-0.04, 0.00)	-0.02 (-0.04, 0.00)	-0.02 (-0.03, 0.00)	-0.01 (-0.03, 0.01)	-0.01 (-0.03, 0.01)
50-52 years	0	0	0	0	0	0
>52 years	0.00 (-0.01, 0.02)	0.00 (-0.01, 0.02)	0.00 (-0.02, 0.01)	0.00 (-0.02, 0.01)	0.01 (0.00, 0.03)	0.01 (-0.01, 0.02)
Parity, binary						
Nulliparous	-0.04 (-0.06, -0.03)	-0.04 (-0.06, -0.03)	-0.07 (-0.08, -0.05)	-0.07 (-0.08, -0.06)	0.04 (0.03, 0.05)	0.04 (0.03, 0.05)
Parous	0	0	0	0	0	0

Tang R, *et al. BMJ Open* 2019; 9:e030318. doi: 10.1136/bmjopen-2019-030318

Parity, ordered categories	0	0	0	0	0	0
0	0.01 (-0.01, 0.03)	0.01 (0.00, 0.03)	0.02 (0.01, 0.04)	0.03 (0.01, 0.04)	-0.02 (-0.04, -0.01)	-0.02 (-0.04, 0.00)
1	0.05 (0.03, 0.06)	0.04 (0.03, 0.06)	0.07 (0.06, 0.08)	0.07 (0.06, 0.08)	-0.04 (-0.06, -0.03)	-0.04 (-0.06, -0.03)
2	0.05 (0.04, 0.07)	0.05 (0.04, 0.07)	0.08 (0.07, 0.09)	0.08 (0.06, 0.09)	-0.04 (-0.06, -0.03)	-0.04 (-0.06, -0.03)
3	0.05 (0.04, 0.07)	0.06 (0.04, 0.07)	0.08 (0.07, 0.10)	0.09 (0.07, 0.10)	-0.06 (-0.08, -0.04)	-0.05 (-0.07, -0.03)
>3						
PCOS/ovarian cysts						
No	0	0	0	0	0	0
Yes	-0.01 (-0.04, 0.03)	-0.01 (-0.04, 0.02)	-0.03 (-0.07, 0.00)	-0.04 (-0.07, 0.00)	0.05 (0.02, 0.08)	0.06 (0.03, 0.09)
Endometriosis						
No	0	0	0	0	0	0
Yes	0.01 (-0.02, 0.05)	0.01 (-0.03, 0.04)	-0.02 (-0.05, 0.01)	-0.03 (-0.06, 0.01)	0.07 (0.03, 0.10)	0.07 (0.03, 0.10)
Oral contraception						
Never used	0	0	0	0	0	0
Ever used	0.02 (0.01, 0.03)	0.02 (0.01, 0.04)	0.02 (0.01, 0.03)	0.02 (0.01, 0.03)	0.01 (0.00, 0.02)	0.01 (0.00, 0.03)
Years of oral contraception						
0	0	0	0	0	0	0
1	0.01 (-0.01, 0.03)	0.01 (-0.01, 0.03)	0.01 (-0.01, 0.02)	0.01 (-0.01, 0.02)	0.01 (-0.01, 0.02)	0.01 (0.00, 0.03)

Tang R, *et al. BMJ Open* 2019; 9:e030318. doi: 10.1136/bmjopen-2019-030318

2-4	0.02 (0.01, 0.03)	0.02 (0.01, 0.04)	0.02 (0.00, 0.03)	0.02 (0.00, 0.03)	0.01 (0.00, 0.02)	0.01 (0.00, 0.03)
5-9	0.03 (0.01, 0.04)	0.03 (0.01, 0.04)	0.03 (0.01, 0.04)	0.02 (0.01, 0.04)	0.01 (-0.01, 0.02)	0.02 (0.00, 0.03)
10-15	0.03 (0.02, 0.05)	0.04 (0.02, 0.05)	0.03 (0.02, 0.05)	0.03 (0.02, 0.05)	0.00 (-0.01, 0.02)	0.01 (0.00, 0.03)
>15	0.02 (0.00, 0.03)	0.02 (0.01, 0.04)	0.02 (0.00, 0.03)	0.02 (0.00, 0.03)	0.00 (-0.01, 0.02)	0.01 (0.00, 0.03)
HRT						
Never used	0	0	0	0	0	0
Ever used	0.00 (-0.01, 0.01)	0.00 (-0.01, 0.01)	0.00 (-0.01, 0.01)	0.00 (-0.01, 0.01)	0.01 (0.00, 0.02)	0.02 (0.01, 0.03)
Years of HRT						
0	0	0	0	0	0	0
1-2	0.00 (-0.02, 0.01)	0.00 (-0.01, 0.02)	0.00 (-0.02, 0.01)	0.00 (-0.02, 0.01)	0.01 (0.00, 0.03)	0.02 (0.01, 0.04)
3-5	0.00 (-0.02, 0.02)	0.01 (-0.01, 0.02)	0.00 (-0.02, 0.01)	0.00 (-0.01, 0.02)	0.01 (-0.01, 0.02)	0.02 (0.00, 0.03)
6-10	0.00 (-0.02, 0.02)	0.01 (-0.01, 0.02)	0.00 (-0.02, 0.02)	0.00 (-0.02, 0.02)	0.01 (-0.01, 0.03)	0.02 (0.00, 0.04)
>10	-0.01 (-0.03, 0.01)	0.00 (-0.01, 0.02)	0.00 (-0.02, 0.02)	0.00 (-0.01, 0.02)	0.00 (-0.02, 0.02)	0.01 (0.01, 0.03)
Gynaecological surgery						
No	0	0	0	0	0	0
Hysterectomy	0.01 (0.00, 0.03)	0.02 (0.00, 0.03)	0.00 (-0.02, 0.01)	0.00 (-0.01, 0.01)	0.04 (0.02, 0.05)	0.04 (0.03, 0.06)
Bilateral oophorectomy	-0.02 (-0.09, 0.05)	-0.02 (-0.09, 0.05)	-0.04 (-0.11, 0.03)	-0.05 (-0.11, 0.03)	0.05 (-0.02, 0.12)	0.04 (-0.04, 0.12)
Both	0.00 (-0.01, 0.02)	0.00 (-0.01, 0.02)	-0.02 (-0.04, -0.01)	-0.02 (-0.04, 0.00)	0.05 (0.04, 0.07)	0.05 (0.04, 0.07)

FEV1, forced expiratory volume in one-second; FVC, forced vital capacity; CI, confidence interval; PCOS, polycystic ovary syndrome; HRT, hormone replacement therapy

[a]Adjusted for age, BMI (log-transformed), ethnicity, education, household income, Townsend deprivation index, smoking history duration, smoking history intensity, asthma, maternal COPD and paternal COPD

[b]Multiple-adjusted as above with an additional covariate of smoking status and adjusting for smoking history as one covariate (pack-years) rather than two covariates (smoking duration and smoking intensity)

[c]Analysed in women who experienced natural menopause before baseline (n=161 069)

Tang R, *et al. BMJ Open* 2019; 9:e030318. doi: 10.1136/bmjopen-2019-030318

Table S8. Linear regression analyses of female reproductive health indicators with baseline spirometry measures, **further adjusting for comorbidities** (N=273 441)

Reproductive health indicator	Mean change in FEV_1 z-score (95% CI)		Mean change in FVC z-score (95% CI)		Mean change in FEV_1/FVC z-score (95% CI)	
	**Multiple-adjusted[a]**	**Further adjusted for comorbidities[b]**	**Multiple-adjusted[a]**	**Further adjusted for comorbidities[b]**	**Multiple-adjusted[a]**	**Further adjusted for comorbidities[b]**
Age at menarche						
<12 years	-0.01 (-0.02, 0.00)	-0.01 (-0.02, 0.00)	-0.01 (-0.02, -0.01)	-0.01 (-0.02, 0.01)	-0.02 (-0.03, -0.01)	-0.02 (-0.03, -0.01)
12-15 years	0	0	0	0	0	0
>15 years	-0.03 (-0.05, -0.01)	-0.03 (-0.05, -0.01)	-0.03 (-0.05, -0.02)	-0.03 (-0.05, -0.02)	0.00 (-0.02, 0.02)	0.00 (-0.02, 0.02)
Menopause status						
No	0	0	0	0	0	0
Yes	-0.04 (-0.05, -0.02)	-0.04 (-0.06, -0.03)	-0.04 (-0.06, -0.03)	-0.04 (-0.05, -0.02)	0.01 (-0.01, 0.02)	0.00 (-0.02, 0.01)
Age at natural menopause[c]						
<47 years	-0.04 (-0.05, -0.02)	-0.03 (-0.05, -0.02)	-0.04 (-0.05, -0.02)	-0.04 (-0.05, -0.02)	-0.01 (-0.02, 0.01)	-0.01 (-0.02, 0.01)
47-49 years	-0.02 (-0.04, 0.00)	-0.02 (-0.04, 0.00)	-0.02 (-0.03, 0.00)	-0.02 (-0.03, 0.00)	-0.01 (-0.03, 0.01)	-0.01 (-0.03, 0.01)
50-52 years	0	0	0	0	0	0
>52 years	0.00 (-0.01, 0.02)	0.00 (-0.01, 0.02)	0.00 (-0.02, 0.01)	0.00 (-0.02, 0.01)	0.01 (0.00, 0.02)	0.01 (0.00, 0.02)
Parity, binary						
Nulliparous	-0.04 (-0.06, -0.03)	-0.04 (-0.06, -0.03)	-0.07 (-0.08, -0.05)	-0.07 (-0.08, -0.05)	0.04 (0.03, 0.05)	0.04 (0.03, 0.05)
Parous	0	0	0	0	0	0
Parity, ordered categories						
0	0	0	0	0	0	0

1	0.01 (0.00, 0.03)	0.01 (0.00, 0.03)	0.02 (0.01, 0.04)	0.02 (0.01, 0.04)	-0.02 (-0.04, 0.00)	-0.02 (-0.04, 0.00)
2	0.04 (0.03, 0.06)	0.04 (0.03, 0.06)	0.07 (0.06, 0.08)	0.07 (0.06, 0.08)	-0.04 (-0.06, -0.03)	-0.04 (-0.06, -0.03)
3	0.05 (0.04, 0.07)	0.05 (0.04, 0.07)	0.08 (0.06, 0.09)	0.08 (0.06, 0.09)	-0.04 (-0.06, -0.03)	-0.04 (-0.06, -0.03)
>3	0.05 (0.04, 0.07)	0.05 (0.04, 0.07)	0.08 (0.07, 0.10)	0.08 (0.07, 0.10)	-0.06 (-0.07, -0.04)	-0.06 (-0.07, -0.04)
PCOS/ovarian cysts						
No	0	0	0	0	0	0
Yes	-0.01 (-0.04, 0.03)	-0.01 (-0.04, 0.03)	-0.03 (-0.07, 0.00)	-0.03 (-0.07, 0.00)	0.05 (0.02, 0.09)	0.05 (0.02, 0.09)
Endometriosis						
No	0	0	0	0	0	0
Yes	0.01 (-0.02, 0.05)	0.01 (-0.02, 0.05)	-0.02 (-0.05, 0.01)	-0.02 (-0.05, 0.01)	0.07 (0.03, 0.10)	0.07 (0.03, 0.10)
Oral contraception						
Never used	0	0	0	0	0	0
Ever used	0.03 (0.01, 0.04)	0.03 (0.01, 0.04)	0.02 (0.01, 0.03)	0.02 (0.01, 0.03)	0.01 (0.003, 0.03)	0.01 (0.00, 0.03)
Years of oral contraception						
0	0	0	0	0	0	0
1	0.01 (-0.01, 0.03)	0.01 (0.00, 0.03)	0.01 (-0.01, 0.03)	0.01 (-0.01, 0.03)	0.01 (0.00, 0.03)	0.01 (0.00, 0.03)
2-4	0.02 (0.01, 0.04)	0.02 (0.01, 0.04)	0.02 (0.01, 0.03)	0.02 (0.01, 0.03)	0.02 (0.00, 0.03)	0.02 (0.00, 0.03)
5-9	0.03 (0.02, 0.05)	0.03 (0.02, 0.05)	0.03 (0.01, 0.04)	0.03 (0.01, 0.04)	0.02 (0.00, 0.03)	0.02 (0.00, 0.03)
10-15	0.04 (0.02, 0.05)	0.04 (0.02, 0.05)	0.04 (0.02, 0.05)	0.03 (0.02, 0.05)	0.01 (0.00, 0.03)	0.01 (0.00, 0.03)
>15	0.02 (0.01, 0.04)	0.02 (0.01, 0.04)	0.02 (0.00, 0.03)	0.02 (0.00, 0.03)	0.01 (0.00, 0.03)	0.01 (0.00, 0.03)
HRT						
Never used	0	0	0	0	0	0

Tang R, *et al. BMJ Open* 2019; 9:e030318. doi: 10.1136/bmjopen-2019-030318

Ever used	0.00 (0.00, 0.01)	0.00 (-0.01, 0.01)	0.00 (-0.01, 0.01)	0.00 (-0.01, 0.01)	0.02 (0.01, 0.03)	0.02 (0.01, 0.03)
Years of HRT						
0	0	0	0	0	0	0
1-2	0.00 (-0.01, 0.02)	0.01 (-0.01, 0.02)	0.00 (-0.02, 0.01)	0.00 (-0.02, 0.01)	0.02 (0.01, 0.04)	0.02 (0.00, 0.04)
3-5	0.00 (-0.01, 0.02)	0.00 (-0.01, 0.02)	0.00 (-0.02, 0.02)	0.00 (-0.01, 0.02)	0.02 (0.00, 0.03)	0.01 (0.00, 0.03)
6-10	0.01 (-0.01, 0.02)	0.01 (-0.01, 0.03)	0.00 (-0.02, 0.02)	0.00 (-0.02, 0.02)	0.02 (0.00, 0.04)	0.02 (0.00, 0.04)
>10	0.00 (-0.01, 0.02)	0.00 (-0.01, 0.02)	0.00 (-0.01, 0.02)	0.00 (-0.01, 0.02)	0.01 (-0.01, 0.03)	0.01 (-0.01, 0.03)
Gynaecological surgery						
No	0	0	0	0	0	0
Hysterectomy	0.01 (0.00, 0.03)	0.02 (0.00, 0.03)	0.00 (-0.02, 0.01)	0.00 (-0.01, 0.01)	0.04 (0.02, 0.06)	0.04 (0.02, 0.06)
Bilateral oophorectomy	-0.02 (-0.08, 0.05)	-0.01 (-0.08, 0.05)	-0.04 (-0.11, 0.03)	-0.04 (-0.11, 0.03)	0.05 (-0.02, 0.12)	0.05 (-0.02, 0.12)
Both	0.00 (-0.01, 0.02)	0.01 (-0.01, 0.02)	-0.02 (-0.04, -0.01)	-0.02 (-0.03, 0.00)	0.05 (0.04, 0.07)	0.05 (0.04, 0.07)

FEV1, forced expiratory volume in one-second; FVC, forced vital capacity; CI, confidence interval; PCOS, polycystic ovary syndrome; HRT, hormone replacement therapy

[a]Adjusted for age, BMI (log-transformed), ethnicity, education, household income, Townsend deprivation index, smoking history in pack-years, asthma, maternal COPD and paternal COPD

[b]Further adjusted from the multiple-adjusted analyses[a] for a history of cardiovascular disease and/or diabetes at baseline

[c]Analysed in women who experienced natural menopause before baseline (n=161 069)

Table S9. Cox regression analyses of female reproductive health indicators with incident COPD-related **death** during follow-up (N=273 441)

Reproductive health indicator	% of participants	Person-years of follow-up	Number of COPD deaths	Age-adjusted Hazard ratio	Age-adjusted 95% CI	Multiple-adjusted[a] Hazard ratio	Multiple-adjusted[a] 95% CI
Age at menarche							
<12 years	20.0	332 700	51	1.62	1.15, 2.27	1.52	1.08, 2.13
12-15 years	74.1	1 229 881	115	1.0	NA	1.0	NA
>15 years	5.9	97 672	13	1.49	0.84, 2.65	1.18	0.66, 2.11
Menopausal status							
Pre	26.4	461 595	6	1.0	NA	1.0	NA
Post	73.6	1 198 658	173	2.03	0.76	1.39	0.54, 3.63
Age at natural menopause[b]							
<47 years	20.7	200 666	47	2.47	1.54, 3.97	1.49	0.92, 2.42
47-49 years	12.8	154 965	28	1.92	1.10, 3.34	1.49	0.85, 2.61
50-52 years	23.7	345 140	35	1.0	NA	1.0	NA
>52 years	16.3	275 067	28	0.87	0.51, 1.48	0.96	0.56, 1.64
Parity, binary							
Nulliparous	15.0	248 399	18	0.83	0.51, 1.35	1.04	0.63, 1.70
Parous	85.0	1 411 854	161	1.0	NA	1.0	NA
Parity, ordered categories							
0	15.0	248 399	18	1.0	NA	1.0	NA
1	12.2	201 277	28	1.79	0.99, 3.23	1.24	0.69, 2.26
2	34.4	571 145	44	0.82	0.47, 1.42	0.77	0.45, 1.35

Tang R, *et al. BMJ Open* 2019; 9:e030318. doi: 10.1136/bmjopen-2019-030318

3	25.3	420 337	41	1.03	0.59, 1.80	0.86	0.49, 1.50
>3	13.2	219 093	48	2.07	1.20, 3.58	1.22	0.70, 2.13
PCOS/ovarian cysts							
No	98.3	1 632 388	175	1.0	NA	1.0	NA
Yes	1.7	27 861	4	1.45	0.54, 3.91	1.52	0.56, 4.11
Endometriosis							
No	98.5	1 635 728	178	1.0	NA	1.0	NA
Yes	1.5	24 526	1	0.50	0.07, 3.60	0.61	0.08, 4.34
Oral contraception							
Never used	19.0	315 862	50	1.0	NA	1.0	NA
Ever used	81.0	1 344 391	129	1.03	0.74, 1.45	0.87	0.62, 1.22
Years of oral contraception							
0	19.0	315 862	50	1.0	NA	1.0	NA
1	11.1	183 949	26	1.30	0.80, 2.12	0.97	0.60, 1.57
2-4	18.7	311 688	33	0.92	0.59, 1.45	0.81	0.52, 1.27
5-9	17.7	294 898	22	0.83	0.50, 1.38	0.74	0.45, 1.24
10-15	17.4	289 787	25	1.04	0.64, 1.71	0.85	0.52, 1.39
>15	16.0	264 067	23	1.25	0.75, 2.09	1.05	0.63, 1.75
HRT							
Never used	63.7	1 024 004	72	1.0	NA	1.0	NA
Ever used	36.6	636 249	107	1.33	0.98, 1.81	0.99	0.73, 1.35
Years of HRT							

Tang R, *et al. BMJ Open* 2019; 9:e030318. doi: 10.1136/bmjopen-2019-030318

0	63.7	1 024 004	72	1.0	NA	1.0	NA
1-2	11.8	178 712	38	1.53	1.00, 2.34	1.05	0.69, 1.61
3-5	9.1	140 626	35	1.82	1.17, 2.82	1.29	0.83, 2.00
6-10	6.2	94 520	10	0.71	0.34, 1.48	0.64	0.31, 1.33
>10	9.2	222 391	24	1.00	0.60, 1.67	0.83	0.50, 1.38
Gynaecological surgery							
No	81.7	1 350 007	120	1.0	NA	1.0	NA
Hysterectomy	10.1	172 764	36	1.63	1.11, 2.38	1.37	0.94, 2.01
Bilateral oophorectomy	0.4	6130	1	1.50	0.21, 10.71	1.32	0.18, 9.48
Both	7.8	131 351	23	1.40	0.89, 2.20	1.19	0.75, 1.87

COPD, chronic obstructive pulmonary disease; CI, confidence interval; PCOS, polycystic ovary syndrome; HRT, hormone replacement

[a]Multiple-adjusted for age, height, BMI (log-transformed), ethnicity, education, household income, Townsend deprivation index, smoking history, maternal COPD and paternal COPD

[b]Age at menopause was analysed only among women who experienced natural menopause before baseline (n=161 069)

Table S10. Cox regression analyses of female reproductive health indicators with incident COPD-related hospitalisation/death during follow-up in **complete cases** (N=220 773)

Reproductive health indicator	% of participants[a]	Person-years of follow-up	Number of COPD events	Age-adjusted Hazard ratio	Age-adjusted 95% CI	Multiple-adjusted[b] Hazard ratio	Multiple-adjusted[b] 95% CI
Age at menarche							
<12 years	19.4	257 579	222	1.37	1.17, 1.59	1.20	1.03, 1.41
12-15 years	72.1	958 578	598	1.0	NA	1.0	NA
>15 years	5.6	74 151	71	1.57	1.23, 2.01	1.34	1.05, 1.72
Menopausal status							
Pre	23.8	319 918	53	1.0	NA	1.0	NA
Post	60.4	799 833	673	1.83	1.32, 2.54	1.20	0.85, 1.68
Age at natural menopause[c]							
<47 years	32.4	174 207	224	2.12	1.75, 2.57	1.39	1.12, 1.72
47-49 years	17.9	139 602	124	1.58	1.26, 1.98	1.19	0.92, 1.53
50-52 years	28.1	314 777	194	1.0	NA	1.0	NA
>52 years	21.6	252 180	149	0.89	0.72, 1.10	0.92	0.73, 1.17
Parity, binary							
Nulliparous	15.1	200 749	98	0.81	0.65, 1.00	0.97	0.78, 1.20
Parous	83.3	1 107 959	807	1.0	NA	1.0	NA
Parity, ordered categories							
0	15.1	200 749	98	1.0	NA	1.0	NA
1	11.9	157 577	110	1.36	1.03, 1.78	1.00	0.76, 1.32

Tang R, *et al. BMJ Open* 2019; 9:e030318. doi: 10.1136/bmjopen-2019-030318

2	34.0	453 260	240	0.90	0.71, 1.13	0.84	0.67, 1.07
3	24.7	328 906	236	1.21	0.95, 1.53	1.03	0.81, 1.31
>3	12.7	168 215	221	2.11	1.66, 2.68	1.43	1.12, 1.83
PCOS/ovarian cysts							
No	98.3	1 307 646	895	1.0	NA	1.0	NA
Yes	1.7	22 016	22	1.47	0.97, 2.25	1.43	0.94, 2.19
Endometriosis							
No	98.5	1 310 012	907	1.0	NA	1.0	NA
Yes	1.5	19 651	10	0.89	0.48, 1.67	0.97	0.52, 1.80
Oral contraception							
Never used	19.1	253 792	222	1.0	NA	1.0	NA
Ever used	80.7	1 072 330	691	1.07	0.92, 1.25	0.94	0.80, 1.10
Years of oral contraception							
0	19.1	253 792	222	1.0	NA	1.0	NA
1	10.7	141 914	108	1.17	0.92, 1.47	0.93	0.74, 1.17
2-4	18.7	249 392	202	1.17	0.97, 1.42	1.04	0.86, 1.27
5-9	17.8	236 848	147	1.03	0.83, 1.27	0.94	0.76, 1.17
10-15	17.5	232 589	130	0.99	0.79, 1.23	0.85	0.68, 1.06
>15	16.0	211 587	104	0.96	0.75, 1.22	0.84	0.66, 1.07
HRT							
Never used	62.2	827 356	352	1.0	NA	1.0	NA
Ever used	37.5	497 994	562	1.78	1.55, 2.05	1.31	1.13, 1.51

Years of HRT							
0	62.2	827 356	352	1.0	NA	1.0	NA
1-2	10.5	138 603	173	2.12	1.76, 2.56	1.52	1.27, 1.83
3-5	8.3	110 741	117	1.82	1.47, 2.24	1.28	1.04, 1.58
6-10	5.6	74 932	57	1.21	0.92, 1.61	1.03	0.77, 1.36
>10	8.1	107 945	113	1.53	1.23, 1.90	1.24	1.00, 1.54
Gynaecological surgery							
No	81.0	1 077 823	603	1.0	NA	1.0	NA
Hysterectomy	9.7	129 561	163	1.75	1.47, 2.09	1.49	1.25, 1.77
Bilateral oophorectomy	0.4	4611	5	1.63	0.68, 3.94	1.45	0.60, 3.50
Both	7.5	98 833	119	1.67	1.37, 2.04	1.42	1.17, 1.74

COPD, chronic obstructive pulmonary disease; CI, confidence interval; PCOS, polycystic ovary syndrome; HRT, hormone replacement therapy; NA, not applicable

[a]Totals may not equal 100% due to missing values in this complete case analysis

[b]Multiple-adjusted for age, height, BMI (log-transformed), ethnicity, education, household income, Townsend deprivation index, smoking history in pack years, maternal COPD and paternal COPD

[c]Analysed in women who experienced natural menopause before baseline (n=129 961)

Table S11. Linear regression analyses of female reproductive health indicators with baseline spirometry measures in **complete cases** (N=160 116)

Reproductive health indicator	Mean change in FEV_1 z-score (95% CI)		Mean change in FVC z-score (95% CI)		Mean change in FEV_1/FVC z-score (95% CI)	
	Unadjusted	Multiple-adjusted[a]	Unadjusted	Multiple-adjusted[a]	Unadjusted	Multiple-adjusted[a]
Age at menarche						
<12 years	-0.05 (-0.07, -0.04)	-0.01 (-0.03, 0.00)	-0.07 (-0.08, -0.06)	0.00 (-0.02, 0.01)	0.02 (0.01, 0.03)	-0.02 (-0.03, -0.01)
12-15 years	0	0	0	0	0	0
>15 years	-0.10 (-0.13, -0.08)	-0.04 (-0.06, -0.02)	-0.09 (-0.11, -0.07)	-0.05 (-0.07, -0.03)	-0.06 (-0.08, -0.04)	0.00 (-0.02, 0.02)
Menopause status						
No	0	0	0	0	0	0
Yes	-0.04 (-0.05, -0.03)	-0.06 (-0.08, -0.04)	-0.03 (-0.05, -0.02)	-0.06 (-0.07, -0.04)	-0.02 (-0.03, -0.01)	-0.02 (-0.03, 0.00)
Age at natural menopause[b]						
<47 years	-0.15 (-0.17, -0.13)	-0.06 (-0.07, -0.04)	-0.13 (-0.15, -0.11)	-0.06 (-0.08, -0.04)	-0.07 (-0.09, -0.05)	-0.01 (-0.03, 0.01)
47-49 years	-0.07 (-0.09, -0.05)	-0.03 (-0.05, -0.01)	-0.06 (-0.08, -0.04)	-0.03 (-0.04, -0.01)	-0.04 (-0.06, -0.02)	-0.01 (-0.03, 0.01)
50-52 years	0	0	0	0	0	0
>52 years	0.01 (-0.01, 0.02)	0.00 (-0.02, 0.02)	-0.01 (-0.03, 0.00)	-0.01 (-0.03, 0.01)	0.04 (0.03, 0.06)	0.02 (0.00, 0.03)
Parity, binary						
Nulliparous	-0.03 (-0.04, -0.01)	-0.06 (-0.07, -0.04)	-0.05 (-0.07, -0.04)	-0.08 (-0.09, -0.07)	0.05 (0.03, 0.06)	0.05 (0.03, 0.06)
Parous	0	0	0	0	0	0
Parity, ordered categories						
0	0	0	0	0	0	0
1	-0.03 (-0.05, -0.02)	0.01 (0.00, 0.03)	-0.01 (-0.02, 0.01)	0.03 (0.01, 0.04)	-0.06 (-0.08, -0.04)	-0.02 (-0.04, -0.01)
2	0.06 (0.04, 0.08)	0.06 (0.05, 0.07)	0.08 (0.07, 0.10)	0.09 (0.07, 0.10)	-0.03 (-0.05, -0.02)	-0.05 (-0.06, -0.03)

3	0.04 (0.03, 0.06)	0.07 (0.05, 0.08)	0.07 (0.05, 0.09)	0.10 (0.08, 0.11)	-0.05 (-0.06, -0.03)	-0.05 (-0.07, -0.03)
>3	-0.04 (-0.06, -0.02)	0.06 (0.04, 0.08)	-0.01 (-0.03, 0.01)	0.10 (0.08, 0.11)	-0.07 (-0.09, -0.06)	-0.06 (-0.08, -0.04)
PCOS/ovarian cysts						
No	0	0	0	0	0	0
Yes	-0.02 (-0.06, 0.02)	0.00 (-0.04, 0.03)	-0.06 (-0.09, -0.02)	-0.03 (-0.07, 0.00)	0.07 (0.03, 0.11)	0.07 (0.03, 0.11)
Endometriosis						
No	0	0	0	0	0	0
Yes	-0.01 (-0.05, 0.04)	0.00 (-0.03, 0.04)	-0.04 (-0.08, 0.01)	-0.03 (-0.07, 0.01)	0.06 (0.02, 0.10)	0.06 (0.03, 0.10)
Oral contraception						
Never used	0	0	0	0	0	0
Ever used	0.08 (0.07, 0.09)	0.03 (0.02, 0.04)	0.09 (0.08, 0.11)	0.03 (0.02, 0.04)	-0.01 (-0.02, 0.00)	0.02 (0.01, 0.03)
Years of oral contraception						
0	0	0	0	0	0	0
1	0.04 (0.02, 0.06)	0.02 (0.00, 0.03)	0.05 (0.03, 0.07)	0.01 (-0.01, 0.03)	-0.03 (-0.05, -0.01)	0.01 (-0.01, 0.03)
2-4	0.07 (0.05, 0.08)	0.03 (0.01, 0.05)	0.07 (0.06, 0.09)	0.02 (0.01, 0.04)	0.00 (-0.01, 0.02)	0.02 (0.01, 0.04)
5-9	0.10 (0.08, 0.11)	0.04 (0.02, 0.05)	0.11 (0.09, 0.12)	0.03 (0.02, 0.05)	0.00 (-0.01, 0.02)	0.02 (0.00, 0.04)
10-15	0.10 (0.09, 0.12)	0.05 (0.03, 0.06)	0.12 (0.11, 0.14)	0.05 (0.03, 0.06)	-0.02 (-0.03, 0.00)	0.02 (0.00, 0.03)
>15	0.08 (0.07, 0.10)	0.03 (0.01, 0.04)	0.10 (0.08, 0.12)	0.02 (0.01, 0.04)	-0.03 (-0.05, -0.01)	0.01 (-0.01, 0.03)
HRT						
Never used	0	0	0	0	0	0
Ever used	-0.03 (-0.04, -0.02)	0.00 (-0.01, 0.01)	-0.01 (-0.02, 0.00)	-0.01 (-0.02, 0.00)	-0.04 (-0.05, -0.03)	0.02 (0.01, 0.03)
Years of HRT						

Tang R, *et al. BMJ Open* 2019; 9:e030318. doi: 10.1136/bmjopen-2019-030318

0	0	0	0	0	0	0
1-2	-0.03 (-0.05, -0.02)	0.00 (-0.02, 0.01)	-0.02 (-0.03, 0.00)	-0.01 (-0.03, 0.01)	-0.05 (-0.06, -0.03)	0.02 (0.00, 0.03)
3-5	-0.04 (-0.06, -0.02)	0.00 (-0.02, 0.02)	-0.03 (-0.05, -0.01)	0.00 (-0.02, 0.02)	-0.04 (-0.05, -0.02)	0.01 (-0.01, 0.03)
6-10	0.00 (-0.02, 0.02)	0.01 (-0.01, 0.03)	0.01 (-0.02, 0.03)	0.00 (-0.02, 0.02)	-0.01 (-0.03, 0.01)	0.02 (0.00, 0.04)
>10	-0.01 (-0.03, 0.01)	0.00 (-0.02, 0.02)	0.01 (-0.01, 0.03)	0.00 (-0.02, 0.02)	-0.03 (-0.05, -0.02)	0.01 (-0.01, 0.03)
Gynaecological surgery						
No	0	0	0	0	0	0
Hysterectomy	-0.04 (-0.06, -0.03)	0.02 (0.00, 0.04)	-0.06 (-0.08, -0.05)	0.00 (-0.01, 0.02)	0.03 (0.02, 0.05)	0.05 (0.03, 0.07)
Bilateral oophorectomy	-0.07 (-0.15, 0.01)	-0.05 (-0.13, 0.03)	-0.10 (-0.18, -0.01)	-0.08 (-0.16, 0.00)	0.05 (-0.03, 0.14)	0.06 (-0.02, 0.14)
Both	-0.05 (-0.07, -0.03)	0.00 (-0.02, 0.02)	-0.08 (-0.10, -0.06)	-0.03 (-0.04, -0.01)	0.06 (0.04, 0.08)	0.06 (0.04, 0.08)

FEV_1, forced expiratory volume in one-second; FVC, forced vital capacity; CI, confidence interval; PCOS, polycystic ovary syndrome; HRT, hormone replacement therapy; NA, not applicable

[a]Adjusted for age, BMI (log-transformed), ethnicity, education, household income, Townsend deprivation index, smoking history pack-years, asthma, maternal COPD and paternal COPD

[b]Analysed in women who experienced natural menopause before baseline (n=121 153)
