# Supplementary material for: Female reproductive history in relation to chronic obstructive pulmonary disease and lung function in UK biobank: a prospective population-based cohort study
Source: BMJ Open. 2019 Oct 28;9(10):e030318. doi: 10.1136/bmjopen-2019-030318 (PMC6830692; doi:10.1136/bmjopen-2019-030318)
Supplement: Author's manuscript [file bmjopen-2019-030318.draft_revisions.pdf]

BMJ Open is committed to open peer review. As part of this commitment we make the peer review history of every article we publish publicly available.

When an article is published we post the peer reviewers' comments and the authors' responses online. We also post the versions of the paper that were used during peer review. These are the versions that the peer review comments apply to.

The versions of the paper that follow are the versions that were submitted during the peer review process. They are not the versions of record or the final published versions. They should not be cited or distributed as the published version of this manuscript.

BMJ Open is an open access journal and the full, final, typeset and author-corrected version of record of the manuscript is available on our site with no access controls, subscription charges or pay-per-view fees (http://bmjopen.bmj.com).

If you have any questions on BMJ Open's open peer review process please email info.bmjopen@bmj.com

BMJ Open

Female reproductive history in relation to chronic obstructive pulmonary disease and lung function in UK Biobank: a prospective population-based cohort study

Journal:	*BMJ Open*
Manuscript ID	bmjopen-2019-030318
Article Type:	Research
Date Submitted by the Author:	08-Mar-2019
Complete List of Authors:	Tang, Rosalind; University of Bristol Faculty of Health Sciences<br>Fraser, Abigail; University of Bristol, MRC Integrative Epidemiology Unit; University of Bristol, Department of Population Health Sciences<br>Magnus, Maria; University of Bristol, Department of Population Health Sciences; Norwegian Institute of Public Health, Centre for Fertility and Health
Keywords:	chronic obstructive pulmonary disease, spirometry, menarche, menopause, oral contraceptives, hormone replacement therapy

SCHOLARONE™
Manuscripts

Female reproductive history in relation to chronic obstructive pulmonary disease and lung function in UK Biobank: a prospective population-based cohort study

Authors: Rosalind Tang[1,2]*, Abigail Fraser[3,4], Maria C. Magnus[3,4,5]

Author affiliations: [1]Bristol Medical School, Faculty of Health Sciences, University of Bristol, Bristol, United Kingdom; [2]Keenan Research Centre for Biomedical Science, St. Michael's Hospital, Toronto, Canada; [3]MRC Integrative Epidemiology Unit at the University of Bristol, Bristol, United Kingdom; [4]Department of Population Health Sciences, Bristol Medical School, University of Bristol, Bristol, United Kingdom; [5]Centre for Fertility and Health, Norwegian Institute of Public Health, Oslo, Norway.

***Corresponding author:** Rosalind Tang

Address: Musgrove Park Academy, Musgrove Park Hospital, Taunton, Somerset, TA1 5DA, United Kingdom

Email: rt16358@my.bristol.ac.uk

For peer review only - http://bmjopen.bmj.com/site/about/guidelines.xhtml

ABSTRACT

Objectives: Sex differences in respiratory physiology and predilection for developing chronic obstructive pulmonary disease (COPD) have been documented, suggesting that female sex hormones may influence pathogenesis. We investigated whether aspects of female reproductive health might play a role in risk of COPD among women.

Design: Population-based prospective cohort study.

Setting: UK Biobank recruited across 22 centres in the United Kingdom between 2006-2010.

Primary and secondary outcomes measures: We examined a range of female reproductive health indicators in relation to risk of COPD-related hospitalisation/death (n=271 271) using Cox proportional hazards regression; and lung function (n=273 441) using linear regression.

Results: Parity >3 was associated with greater risk of COPD-related hospitalisation/death (adjusted HR 1.45; 95% CI: 1.16, 1.82) and lower FEV_1/FVC (adjusted mean difference -0.06; 95% CI: -0.07, -0.04). Any oral contraception use was associated with lower risk of COPD-related hospitalisation/death (adjusted HR 0.85; 95% CI: 0.74, 0.97) and greater FEV_1/FVC (adjusted mean difference 0.01; 95% CI: 0.003, 0.03). Late menarche (age >15) and early menopause (age <47) were also associated with greater risk of COPD-related hospitalisation/death (but not lung function), while endometriosis was associated with greater FEV_1/FVC (not COPD-related hospitalisation/death). Early menarche (age <12 years) was associated with lower FEV_1/FVC (but not COPD hospitalisation/death). Associations with PCOS or ovarian cysts; any HRT use; hysterectomy-alone; and both hysterectomy and bilateral oophorectomy were in opposing directions for COPD-related hospitalisation/death (greater risk) and FEV_1/FVC (positive association).

Conclusions: Multiple female reproductive health indicators across the life course are associated with COPD-related hospitalisation/death and lung function. Further studies are necessary to understand the opposing associations of PCOS/ovarian cysts, HRT and hysterectomy with COPD and objective measures of airway obstruction.

For peer review only - http://bmjopen.bmj.com/site/about/guidelines.xhtml

Strengths and limitations of this study

- This large, population-based, prospective cohort study is the first to assess COPD risk with age at menarche, menopause status, hysterectomy, gynaecological surgery, endometriosis or PCOS.
- No study has examined a wide range of reproductive health indicators, spanning the reproductive and post-reproductive periods, with regards to COPD.
- We included a comprehensive list of potential confounders, such as age, height, body mass index, ethnicity, education, household income, Townsend deprivation index, smoking history, asthma, maternal COPD and paternal COPD.
- We relied on participants to self-report reproductive history and we cannot exclude a potential influence of selection bias, due to the low participation rate in UK Biobank.

For peer review only

For peer review only - http://bmjopen.bmj.com/site/about/guidelines.xhtml

INTRODUCTION

There are clear sex differences in lung anatomy and physiology throughout the life course.[1] This is also reflected in the risk of obstructive lung disease. For example, boys have greater risk of asthma during early childhood compared to girls, but this sex difference reverses after puberty, when girls are found to have the greater risk of new-onset asthma.[2] Chronic obstructive pulmonary disease (COPD) has traditionally been seen as a disease predominantly affecting men. However, in more recent years, the overall burden of the disease has shifted more towards women.[2 3] Women with COPD also have, on average, fewer pack-years of smoking history than men with COPD; and non-smokers with COPD are more often female.[1] This points towards a greater underlying susceptibility among women, and highlights the importance of understanding female-specific risk factors for COPD.

Female sex hormones are putative mediators of respiratory health, and regulate bronchodilation, cell proliferation, inflammation and metabolism of toxic cigarette smoke-related metabolites.[1 4-7] Notably, oestrogen and progesterone appear to have varying effects on lung function across the lifespan.[1] We know little, however, about female reproductive health indicators in relation to COPD risk.[1] These reproductive health indicators include markers of endogenous hormone exposure (age at menarche, age at menopause and length of reproductive lifespan), exogenous hormone exposure (oral contraceptive use and hormone replacement therapy), as well as pregnancy history. Of two existing studies, one study found no evidence of an association between hormone replacement therapy (HRT) and COPD risk,[8] while another reported a positive association between early age at first pregnancy (<20 years) and COPD-related mortality.[9] There is more evidence regarding the relationship between female reproductive health indicators and lung function. Early menarche,[10-12] nulliparity,[13] younger age at first pregnancy,[12] and postmenopausal status[14-19] have each been associated with reduced lung function. Evidence of the effects of HRT is contradictory.[14 15 20 21] In the present study, we therefore investigate associations of multiple female

For peer review only - http://bmjopen.bmj.com/site/about/guidelines.xhtml

reproductive health indicators across the lifespan with incidence of COPD hospitalisation/death and lung function in the UK Biobank cohort.

METHODS

Study population

Our study included female participants in UK Biobank (Figure 1), a population-based cohort study which recruited men and women age 40-69 years across 22 centres in England, Scotland and Wales between 2006-2010.[22] The response rate was 5.5%.[22] All participants provided written informed consent. Data on 273 441 women were available after withdrawals. At baseline, participants self-reported sociodemographic information, family medical history, early-life exposures, psychosocial history and medical history. Physical measurements included spirometry. Information on health outcomes after recruitment into the cohort was gathered prospectively via data linkages with hospital and death registers. Register data were available up to 28 February 2015 for England, 16 March 2015 for Wales and 28 October 2014 for Scotland. Data collection in UK Biobank was approved by the NHS National Research Ethics Service (Ref 11/NW/0382).

Female reproductive health indicators

We investigated the following self-reported female reproductive health indicators: age at menarche (<12, 12-15, and >15 years), menopause status (no, yes), age at natural menopause (<47, 47-49, 50-52 and >52 years), parity (0, 1, 2, 3, and >3), history of polycystic ovary syndrome (PCOS) or ovarian cysts, history of endometriosis, oral contraception (OC) use (never/ever), years of OC use (0, 1, 2-4, 5-9, 10-15 and >15), hormone replacement therapy (HRT) use (never/ever), years of HRT use (0, 1-2, 3-5, 6-10, and >10), and gynaecological surgery (hysterectomy, oophorectomy and hysterectomy with bilateral oophorectomy). We calculated years of OC and HRT use as the differences between age at first and last use, and created categories according to quintiles.

For peer review only - http://bmjopen.bmj.com/site/about/guidelines.xhtml

Amaral et al. previously reported decreased lung function in postmenopausal women in UK Biobank.[16] We repeat these analyses for completeness alongside analyses of other female reproductive health indicators.

Respiratory outcomes

Prevalent COPD at baseline was identified using self-report and registrations before baseline in the hospital registers. Incident hospitalisations/deaths from COPD after baseline were identified using hospital and death registers. The following International Classification of Diseases (ICD) codes were used to capture COPD: ICD-9 codes 490-492, 494 and 496; or ICD-10 codes J40-J44.

Forced expiratory volume at one-second (FEV_1) and forced vital capacity (FVC) were measured using Vitalograph Pneumotrac 6800 (Vitalograph, UK) at baseline. Spirometry was not performed if participants had experienced a chest infection in the last month; had a lifetime history of detached retina or collapsed lung; had experienced a heart attack, eye surgery or surgery to the chest or abdomen in the last 3 months; were currently pregnant in the first or third trimester; or were currently using medication for tuberculosis. Two measurements were conducted if the difference between these measurements was ≤5%. A third measurement was conducted if the difference was >5%. We used the single greatest measurements of FEV_1 and FVC per participant. Post-bronchodilator spirometry was not available, although drug treatment was not withheld. Spirometry measurements were converted to internally standardised z-scores by age and height for all analyses.

Statistical analyses

We used Cox proportional hazards regression to examine the association of each female reproductive health indicator in relation to incidence of COPD hospitalisation/death. Participants without COPD at baseline were followed from enrolment until hospitalisation/death from COPD, death from other causes, or the end of follow-up if the participants were still alive and disease-free. The time axis for the Cox regression was calendar time. We examined deviations from the proportional hazard assumption using Schoenfeld residuals. Age- and confounder-adjusted

For peer review only - http://bmjopen.bmj.com/site/about/guidelines.xhtml

estimates are reported as hazard ratios (HR) with 95% confidence intervals (95% CI). The following potential confounders were included in multivariable analyses: age, height, log-transformed body mass index (BMI) at baseline, ethnicity (White or non-White), educational attainment (college, university or other professional degree; Advanced levels, Advanced Subsidiary levels or equivalent; Ordinary levels, General Certificate of Secondary Educations or equivalent; Certificate of Secondary Educations or equivalent; National Vocational Qualifications, Higher National Diploma, Higher National Certificate or equivalent; other), annual household income (<£18 000; £18 001-31 000; £31 001-£52 000; £52 001-£100 000; >£100 000), Townsend deprivation index, smoking history in pack-years (none, <11, 11-20, 21-30, >30), and parental history of COPD. We performed sensitivity analyses of menopause and age at natural menopause with further adjustment for HRT. In addition, we performed sensitivity analyses excluding women with FEV_1/FVC<0.7 at baseline, as they possibly had undiagnosed COPD,[23] and separate analyses further adjusting for baseline smoking status (never, previous, current).

We used linear regression in the analysis of standardised spirometry measurements. The multivariable linear regression models were adjusted for the same covariates as for hospitalisation/death from COPD, with the addition of asthma at baseline. We reported results as mean difference in the spirometry measurements with 95% CI.

We performed multiple imputation by chained equations to account for missing data. Exposures, covariates and outcomes were included in the imputation model. We imputed 20 datasets, which were then analysed using Rubin's rules.[24] Details of the imputation model are given in the online supplementary file (Table S1). All analyses were performed using Stata/MP 15 (StataCorp LLC, College Station, USA). Sensitivity analyses of the complete cases was performed for comparison with our imputed data.

For peer review only - http://bmjopen.bmj.com/site/about/guidelines.xhtml

RESULTS

Of the 273 441 women included in this study, 2170 had prevalent COPD at baseline (Figure 1). These prevalent cases were excluded from the analysis of incident COPD-related hospitalisation/death. By the end of follow-up (median 6 years), there were 1138 incident cases of hospitalisation/death from COPD, yielding an incidence rate of 0.19 per 100 000 person years.

In UK Biobank, 28.4% of women did not have spirometry data, 16.1% were uncertain of or did not report menopause status and 14.3% did not report smoking history. The proportion of missing information on the other covariates were modest (Table 1). Overall, women with spirometry data were younger, more often White, had higher educational attainment, higher annual household income, less deprivation (lower Townsend index), greater smoking history and were more likely to have asthma than women who did not undergo spirometry assessment (online supplementary file Table S2). The distribution of participants' characteristics across the imputed datasets is shown in Table 1.

Late (age >15 years) menarche was associated with increased risk of hospitalisation/death from COPD compared to menarche between age 12-15. The multiple-adjusted HR was 1.37 (95% CI: 1.11, 1.71) (Table 2). There was a modest association between early menarche and COPD hospitalisation/death (HR 1.15; 95% CI: 1.00, 1.32). Early menarche (age <12 years) was associated with lower FEV_1/FVC (Table 3).

Risk of hospitalisation/death from COPD did not differ between pre- and postmenopausal women (Table 2), and this finding was similar after further adjusting for HRT use (HR 1.02; 95% CI: 0.78, 1.35). In examining age at menopause and risk hospitalisation/death from COPD among women who experienced natural menopause (n=159 571), the risk was higher in women who experienced menopause age <47 years (HR 1.44; 95% CI: 1.19, 1.75) when compared to age 50-52 (Table 2). This association remained after further adjustment for HRT use (HR 1.43; 95% CI: 1.18, 1.74).

Postmenopausal status at baseline and early natural menopause were each associated with lower

For peer review only - http://bmjopen.bmj.com/site/about/guidelines.xhtml

FEV_1 and FVC but not FEV_1/FVC (Table 3). These associations did not change after further adjustment for HRT ever use (results not shown).

Hospitalisation/death from COPD did not differ between parous and nulliparous women, but parity >3 was associated with increased risk (HR 1.45; 95% CI: 1.16, 1.82) compared to nulliparity. Women who were nulliparous had lower FEV_1 and FVC but greater FEV_1/FVC compared to parous women, while women with parity >3 had greater FEV_1 and FVC but lower FEV_1/FVC compared to nulliparous women (Table 3).

A history of PCOS or ovarian cysts was associated with increased risk of hospitalisation/death from COPD (HR 1.61; 95% CI: 1.12, 2.32) and greater FEV_1/FVC (mean difference 0.05; 95% CI: 0.02, 0.09), while there was no evidence of an association with FEV_1 or FVC. A history of endometriosis was not associated with COPD hospitalisation/death, FEV_1 or FVC (Tables 2 and 3), though we observed a modest positive association with FEV_1/FVC (mean difference 0.07; 95% CI: 0.03, 0.10).

We observed several associations between exogenous hormone use and COPD hospitalisation/death. Women who had ever used OC had lower risk of hospitalisation/death from COPD compared to never users (HR 0.85; 95% CI: 0.74, 0.97) (Table 2). This appeared to be driven by OC use of >9 years (Table 2). In line with the observed findings for COPD, OC ever use and duration of use showed positive associations with FEV_1, FVC and FEV_1/FVC (Table 3).

Ever using HRT was associated with a modest increased risk of hospitalisation/death from COPD (HR 1.15; 95% CI: 1.01, 1.30) (Table 2). We observed no associations of HRT ever use with FEV_1 or FVC, but a positive association with FEV_1/FVC (Table 3). When we examined the duration of HRT use, we only observed increased hospitalisation/death from COPD (HR 1.33; 95% CI: 1.12, 1.57) and greater FEV_1/FVC (mean difference 0.02; 95% CI: 0.01, 0.04) with 1-2 years of use (Tables 2 and 3). There was no evidence of a dose-response relationship.

For peer review only - http://bmjopen.bmj.com/site/about/guidelines.xhtml

A history of hysterectomy (with or without bilateral oophorectomy), but not bilateral oophorectomy-alone, were associated with greater risk of COPD hospitalisation/death (Table 2). At the same time, hysterectomy was associated with higher, rather than lower, FEV_1/FVC (Tables 3). Hysterectomy with bilateral oophorectomy was also associated with lower FVC (Table 3).

The directions of association between individual female reproductive health indicators with COPD risk and spirometry measures are summarised in Table 4. 24 732 women had FEV_1/FVC<0.7 at baseline. However, our findings after excluding these women were similar to our main findings (online supplementary file Table S3). We also report the results of our sensitivity analyses, after further adjusting for baseline smoking status, in the online supplementary file (Tables S4 and S5). The direction and magnitude of associations remained the same for risk of COPD hospitalisation/death (online supplementary file Table S4) and change in spirometry measures (online supplementary file Table S5). Complete-case analyses of the risk of COPD hospitalisation/death (online supplementary file Table S6) and lung function (online supplementary file Table S7) also produced similar results to those from our imputed dataset.

DISCUSSION

In this large, prospective study, several female reproductive health indicators were associated with COPD hospitalisation/death and/or lung function. Parity greater than three was associated with decreased baseline FEV_1/FVC, a marker of airway obstruction, and increased risk of hospitalisation/death from COPD during follow-up. Ever using OC was associated with greater FEV_1/FVC and lower risk of COPD hospitalisation/death. Associations of hysterectomy, HRT use, and a history of PCOS or ovarian cysts showed inconsistent findings for FEV_1/FVC and COPD hospitalisation/death. This inconsistency may be explained by the fact that spirometry was measured in participants at baseline (age 40-69) and FEV_1 and FEV_1/FVC may have declined during follow-up. Conversely, many patients diagnosed with stage 1 COPD do not go on to develop COPD symptoms or COPD resulting in hospitalisation/death.[25] There is also high intra-individual variability

For peer review only - http://bmjopen.bmj.com/site/about/guidelines.xhtml

in FEV_1.[26] Spirometry assessment at one time point may therefore not be as accurate at identifying clinically-relevant COPD compared to register data on hospitalisation/death.

A study of 424 797 postmenopausal women in the United States reported no difference in COPD mortality by parity.[9] In contrast, our present study reports higher risk of COPD hospitalisation/death among women with more than three births compared to nulliparous women. However, we investigated both COPD-related hospitalisation and death, and we were therefore able to capture less severe COPD cases. A study of 70 965 women in the Nurses' Health Study reported no evidence of an association between HRT ever use and COPD incidence, although the investigators included both probable (self-reported physician diagnosis along with a record of spirometry or imaging at the time of diagnosis or COPD documented on a death register) and definite (self-reported physician diagnosis and FEV_1 <80% predicted) cases of COPD.[8] Here we report a positive association between HRT use and COPD hospitalisation/death. This is the first study to assess COPD risk with regards to age at menarche, menopause status, hysterectomy, gynaecological surgery, endometriosis or PCOS.

Two previous studies reported reduced FEV_1 in adult women who experienced menarche before age 10[10 12] but neither found evidence of an association between age at menarche and FEV_1/FVC. A Mendelian randomisation study of adolescent and adult women across multiple European cohorts, including a subset from UK Biobank, estimated a 24.8 mL increase in FVC per year increase in age at menarche among adult women and a -56.6 mL decrease in FVC per year increase in age at menarche among adolescent girls.[11] This study reported no association between age at menarche and FEV_1/FVC.[11] Notably, this Mendelian randomisation study did not explore non-linear associations between age at menarche and measures of lung function. We observed lower FEV_1 and FVC in adult women with late menarche, and lower FVC and FEV_1/FVC in adult women with early menarche.

A recent systematic review, which included studies from UK Biobank,[16] reported that postmenopausal status was associated with reduced FEV_1 and FVC but not FEV_1/FVC.[18] Current HRT use has been associated with both higher[14] and lower[15] lung function. One study reported an

For peer review only - http://bmjopen.bmj.com/site/about/guidelines.xhtml

association between HRT use and greater FEV_1/FVC.[14] Others have identified differences in lung function according to continuous/cyclic and combined/oestrogen-only HRT.[20 21] Based on the information from participants in UK Biobank, we could not stratify by HRT subtypes.

There are sex differences in lung anatomy and physiology throughout the life course.[1 2 4] During gestation, female foetuses have fewer bronchi, smaller lungs and produce surfactant earlier than male foetuses.[1] Female neonates and children have greater lung function and faster lung growth than their male counterparts, and boys are more likely to be diagnosed with asthma during childhood than girls, while girls are twice as likely as boys to be diagnosed with asthma after puberty.[1] These findings suggest that female sex steroids can accelerate lung maturation and termination of lung growth, which may partially explain why early menarche associates with lower FEV_1/FVC in adulthood. During the reproductive years, parity and PCOS relate to an increase in circulating oestrogen, which has been implicated in the pathogenesis of inflammatory lung diseases including COPD.[6 27-29] Although OC use appears to be protective against COPD, some researchers have suggested that OC use attenuates the fluctuations in sex steroids, which correlates with fluctuations in endothelial nitric oxide, a potent bronchodilator.[1] Additionally, exogenous hormones may not be as influential during reproductive years but may become more influential in after menopause, when endogenous hormone production is low.[6] This is supported by our findings of opposing associations with COPD risk for OC use (lower risk) during reproductive period and HRT use (higher risk) after menopause.

The effect of female sex hormones on COPD and lung health may vary depending on the net effects on airway smooth muscle, inflammation and cigarette smoke metabolism. Oestrogens can regulate the synthesis of nitric oxide, a potent bronchodilator, within human bronchial epithelium,[1 30] as well as regulate airway bronchoconstriction in response to acetylcholine or histamine.[31 32] There is also evidence that progesterone may affect airway smooth muscle constriction, although these reports are limited to animal studies.[33] In addition to regulating bronchodilation and bronchoconstriction, in

For peer review only - http://bmjopen.bmj.com/site/about/guidelines.xhtml

vitro studies report that oestrogen can also modulate the proliferation of airway smooth muscle cells,[34 35] which is a key feature of COPD.[36] Oestrogen's pro- and anti-inflammatory effects have been documented in other female-predominant diseases[37] and may also play a role in COPD, as chronic inflammation is another hallmark of the disease. Finally, oestrogen is an important regulator of toxic cigarette smoke metabolites through cytochrome P450 enzymes.[1] In a mouse model of COPD, Glassberg et al. reported that oestradiol administration after ovariectomy protected against cigarette smoke-induced alveolar septal destruction, macrophage infiltration and decreased other markers of inflammation compared to ovariectomy without oestradiol administration.[38] In the same mouse model, however, non-ovariectomised female mice also had increased small airway remodelling following cigarette smoke exposure when compared to both male mice and ovariectomised female mice.[39] These timing-, dose- and cell-dependent signalling pathways may explain why exposure to female sex steroids, and oestrogen in particular, have potentially contradictory effects on respiratory health.

We cannot exclude a potential influence of selection bias, due to the low participation rate in UK Biobank. Women in the cohort are less likely to have ever smoked, have greater educational attainment, but a similar number of offspring, as compared to the general UK population (https://www.ons.gov.uk/). There is also a lower prevalence of self-reported COPD in UK Biobank (0.8%) compared to physician-diagnosed COPD in the general female UK population (1.68%).[40] Overall, women participating in UK Biobank reflect a higher-educated, more health-conscious group, with more modest underlying risk for COPD compared to the general population. We can only speculate as to how these selection factors might have influenced our associations of interest, but we propose that the most likely direction of any selection bias is towards the null (resulting in an underestimation). Strengths of this study include the large, prospective design. However, we relied on participants to self-report reproductive history which might have led to measurement error. Years of OC or HRT use were estimated as the difference between ages at first and last use. This likely resulted in overestimation of the duration of use, which might have attenuated our findings. In

For peer review only - http://bmjopen.bmj.com/site/about/guidelines.xhtml

addition, we were unable to distinguish between reversible and non-reversible airway obstruction at baseline, as a bronchodilator was not administered before spirometry was conducted. Although we adjusted for self-reported asthma at baseline, this may have led to overestimation of COPD-related airway obstruction among participants with undiagnosed asthma.

In conclusion, our findings indicate that several female reproductive health indicators across the life course are associated with risk of COPD hospitalisation/death and add to the indirect evidence suggesting that that female reproductive hormones may have a role in COPD pathogenesis. Further studies are necessary to understand the opposing associations of hysterectomy, HRT and PCOS/ovarian cysts with COPD and objective measures of airway obstruction.

ACKNOWLEDGEMENTS

This research has been conducted using the UK Biobank Resource under Application Number 6326. We thank all the participants and researchers involved.

CONTRIBUTORS

All authors provided substantial contributions to the design of the work. RT analysed the data and drafted the manuscript. MCM and AF supervised the study, provided further data interpretation and revised the manuscript. All authors approved the final version of the manuscript and agree to be accountable for all aspects of the work in ensuring that questions related to the accuracy or integrity of any part of the work are appropriately investigated and resolved.

DATA SHARING:

Data were obtained from the UK Biobank Resource via its Access Management System (http://www.ukbiobank.ac.uk/wp-content/uploads/2012/09/Access-Procedures-2011-1.pdf).

Funding: This work was supported by the UK Medical Research Council grant MC/UU/12013/5, which provides infrastructure funding to the MRC Integrative Epidemiology Unit at the University of

For peer review only - http://bmjopen.bmj.com/site/about/guidelines.xhtml

Bristol, and grant MR/M009351/1 as a fellowship to AF. This work was partly supported by the Research Council of Norway's Centres of Excellence scheme grant 262700 to MCM.

Competing interests: None declared

Patient and public involvement: The present study did not involve patients and the public.

For peer review only

For peer review only - http://bmjopen.bmj.com/site/about/guidelines.xhtml

REFERENCES

1. Townsend EA, Miller VM, Prakash YS. Sex differences and sex steroids in lung health and disease. *Endocr Rev* 2012;33(1):1-47.

2. Raghavan D, Jain R. Increasing awareness of sex differences in airway diseases. *Respirology* 2016;21(3):449-59.

3. Raghavan D, Varkey A, Bartter T. Chronic obstructive pulmonary disease: the impact of gender. *Curr Opin Pulm Med* 2017;23(2):117-23.

4. Gonzalez-Arenas A, Agramonte-Hevia J. Sex steroid hormone effects in normal and pathologic conditions in lung physiology. *Mini Rev Med Chem* 2012;12(11):1055-62.

5. Kamil F, Pinzon I, Foreman MG. Sex and race factors in early-onset COPD. *Curr Opin Pulm Med* 2013;19(2):140-4.

6. Sathish V, Martin YN, Prakash YS. Sex steroid signaling: implications for lung diseases. *Pharmacol Ther* 2015;150:94-108.

7. Fuentes N, Silveyra P. Endocrine regulation of lung disease and inflammation. *Exp Biol Med (Maywood)* 2018;243(17-18):1313-22.

8. Barr RG, Wentowski CC, Grodstein F, et al. Prospective study of postmenopausal hormone use and newly diagnosed asthma and chronic obstructive pulmonary disease. *Arch Intern Med* 2004;164(4):379-86.

9. Gaudet MM, Carter BD, Hildebrand JS, et al. Associations of parity and age at first pregnancy with overall and cause-specific mortality in the Cancer Prevention Study II. *Fertil Steril* 2017;107(1):179-88.e6.

10. Macsali F, Real FG, Plana E, et al. Early age at menarche, lung function, and adult asthma. *Am J Respir Crit Care Med* 2011;183(1):8-14.

11. Gill D, Sheehan NA, Wielscher M, et al. Age at menarche and lung function: a Mendelian randomization study. *Eur J Epidemiol* 2017;32(8):701-10.

For peer review only - http://bmjopen.bmj.com/site/about/guidelines.xhtml

12. Murri V, Antoniazzi F, Piazza M, et al. Lung Function in Women with Idiopathic Central Precocious Puberty: A Pilot Study. *Horm Res Paediatr* 2017;87(2):95-102.

13. Harik-Khan R, Wise RA, Lou C, et al. The effect of gestational parity on FEV1 in a group of healthy volunteer women. *Respir Med* 1999;93(6):382-8.

14. Carlson CL, Cushman M, Enright PL, et al. Hormone replacement therapy is associated with higher FEV1 in elderly women. *Am J Respir Crit Care Med* 2001;163(2):423-8.

15. Hayatbakhsh MR, Najman JM, O'Callaghan MJ, et al. Association between smoking and respiratory function before and after menopause. *Lung* 2011;189(1):65-71.

16. Amaral AF, Strachan DP, Gomez Real F, et al. Lower lung function associates with cessation of menstruation: UK Biobank data. *Eur Respir J* 2016;48(5):1288-97.

17. Triebner K, Matulonga B, Johannessen A, et al. Menopause Is Associated with Accelerated Lung Function Decline. *Am J Respir Crit Care Med* 2017;195(8):1058-65.

18. Campbell B, Davis SR, Abramson MJ, et al. Menopause, lung function and obstructive lung disease outcomes: a systematic review. *Climacteric* 2018;21(1):3-12.

19. Memoalia J, Anjum B, Singh N, et al. Decline in Pulmonary Function Tests after Menopause. *J Menopausal Med* 2018;24(1):34-40.

20. Cevrioglu AS, Fidan F, Unlu M, et al. The effects of hormone therapy on pulmonary function tests in postmenopausal women. *Maturitas* 2004;49(3):221-7.

21. Pata O, Atis S, Utku Oz A, et al. The effects of hormone replacement therapy type on pulmonary functions in postmenopausal women. *Maturitas* 2003;46(3):213-8.

22. Allen N, Sudlow C, Downey P, et al. UK Biobank: Current status and what it means for epidemiology. *Health Policy Technol* 2012;1(3):123-26.

23. Global Initiative for Chronic Obstructive Lung Disease (GOLD). Global strategy for the diagnosis, management, and prevention of chronic obstructive pulmonary disease (2019 report). GOLD 2019.

For peer review only

For peer review only - http://bmjopen.bmj.com/site/about/guidelines.xhtml

24. Marshall A, Altman DG, Holder RL, et al. Combining estimates of interest in prognostic modelling studies after multiple imputation: current practice and guidelines. *BMC Med Res Methodol* 2009;9:57.

25. Rossi A, Butorac-Petanjek B, Chilosi M, et al. Chronic obstructive pulmonary disease with mild airflow limitation: current knowledge and proposal for future research - a consensus document from six scientific societies. *Int J Chron Obstruct Pulmon Dis* 2017;12:2593-610.

26. Casanova C, Aguirre-Jaime A, de Torres JP, et al. Longitudinal assessment in COPD patients: multidimensional variability and outcomes. *Eur Respir J* 2014;43(3):745-53.

27. Cushman M, Legault C, Barrett-Connor E, et al. Effect of postmenopausal hormones on inflammation-sensitive proteins: the Postmenopausal Estrogen/Progestin Interventions (PEPI) Study. *Circulation* 1999;100(7):717-22.

28. Walsh BW, Paul S, Wild RA, et al. The effects of hormone replacement therapy and raloxifene on C-reactive protein and homocysteine in healthy postmenopausal women: a randomized, controlled trial. *J Clin Endocrinol Metab* 2000;85(1):214-8.

29. Gol M, Akan P, Dogan E, et al. Effects of estrogen, raloxifene, and hormone replacement therapy on serum C-reactive protein and homocysteine levels. *Maturitas* 2006;53(3):252-9.

30. Kirsch EA, Yuhanna IS, Chen Z, et al. Estrogen acutely stimulates endothelial nitric oxide synthase in H441 human airway epithelial cells. *Am J Respir Cell Mol Biol* 1999;20(4):658-66.

31. Degano B, Mourlanette P, Valmary S, et al. Differential effects of low and high-dose estradiol on airway reactivity in ovariectomized rats. *Respir Physiol Neurobiol* 2003;138(2-3):265-74.

32. Townsend EA, Thompson MA, Pabelick CM, et al. Rapid effects of estrogen on intracellular Ca2+ regulation in human airway smooth muscle. *Am J Physiol Lung Cell Mol Physiol* 2010;298(4):L521-30.

33. Ynuk B. Endocrine regulation of airway contractility is overlooked. *J Endocrinol* 2014;222(2):R61-R73.

For peer review only - http://bmjopen.bmj.com/site/about/guidelines.xhtml

34. Stamatiou R, Paraskeva E, Papagianni M, et al. The mitogenic effect of testosterone and 17β-estradiol on airway smooth muscle cells. *Steroids* 2011;76(4):400-08.

35. Ambhore NS, Katragadda R, Raju Kalidhindi RS, et al. Estrogen receptor beta signaling inhibits PDGF induced human airway smooth muscle proliferation. *Mol Cell Endocrinol* 2018;476:37-47.

36. Yan F, Gao H, Zhao H, et al. Roles of airway smooth muscle dysfunction in chronic obstructive pulmonary disease. *J Transl Med* 2018;16(1):262.

37. Straub RH. The Complex Role of Estrogens in Inflammation. *Endocr Rev* 2007;28(5):521-74.

38. Glassberg MK, Catanuto P, Shahzeidi S, et al. Estrogen deficiency promotes cigarette smoke-induced changes in the extracellular matrix in the lungs of aging female mice. *Transl Res* 2016;178:107-17.

39. Tam A, Bates JH, Churg A, et al. Sex-Related Differences in Pulmonary Function following 6 Months of Cigarette Exposure: Implications for Sexual Dimorphism in Mild COPD. *PLoS One* 2016;11(10):e0164835.

40. Simpson CR, Hippisley-Cox J, Sheikh A. Trends in the epidemiology of chronic obstructive pulmonary disease in England: a national study of 51 804 patients. *Br J Gen Pract* 2010;60(576):277-84.

For peer review only

For peer review only - http://bmjopen.bmj.com/site/about/guidelines.xhtml

FIGURE LEGEND

Figure 1. Flow Chart of Study Population.

COPD, chronic obstructive pulmonary disease. Missing data is in one or more of the following variables: height, ethnicity, educational attainment, income, Townsend deprivation index, smoking history, spirometry measurements, maternal COPD history, paternal COPD history, age at menarche, menopause status, age at natural menopause, parity, oral contraception use, hormone replacement therapy use or gynaecological surgery history.

For peer review only

For peer review only - http://bmjopen.bmj.com/site/about/guidelines.xhtml

TABLES

Table 1.

Distribution of observed and imputed data in UK Biobank women at baseline (N=273 441)

**Characteristic**	**% data imputed**	**Mean (SE) or % in observed data**	**Mean (SE) or % in imputed data**
Age (years)	0	56.35 (0.02)	56.35 (0.02)
<55		39.5	39.5
55-59		18.6	18.6
60-64		24.2	24.2
>64		17.8	17.8
Height (cm)	0.4	162.4 (0.01)	162.4 (0.01)
Body mass index (kg/m^2)	0		
<18.5		0.8	0.8
18.5-24.9		38.7	38.7
25.0-29.9		36.5	36.5
>29.9		24.1	24.1
Non-white ethnicity	0.5	5.4	5.4
Education	1.2		
College, university or other professional degree		36.9	37.3
A levels, AS levels or equivalent		11.8	11.9
O levels, GCSEs or equivalent		23.3	23.5
CSE or equivalent		5.4	5.4
NVQ, HND, HNC or equivalent		4.5	4.6
Other		17.0	17.3

For peer review only - http://bmjopen.bmj.com/site/about/guidelines.xhtml

Annual household income	0.4		
<£18,000		20.4	20.6
£18,000-£30,999		21.7	21.8
£31,000-£51,999		20.9	21.0
£52,000-£100,000		15.3	15.4
>£100,000		4.0	4.0
Do not know/prefer not to answer		17.3	17.4
Townsend deprivation index	0.1	-1.33 (0.01)	-1.33 (0.01)
Smoking history (pack-years)	14.3		
None		59.3	69.1
Up to 10		7.9	9.2
11-20		7.4	8.7
21-30		5.2	6.1
>30		5.9	6.9
Prevalent (baseline) COPD	0	0.8	0.8
Incident COPD hospitalisation/death	0	0.4	0.4
Spirometry[a]	28.4		
FEV_1 z-score		0.00001 (0.002)	-0.0008 (0.002)
FVC z-score		-0.000002 (0.002)	-0.001 (0.002)
FEV_1/FVC z-score		0.00003 (0.002)	0.002 (0.002)
Asthma	0	12.9	12.9
Maternal history of COPD	1.9		
No		90.0	91.7
Yes		6.0	6.1
Don't know		2.2	2.2

For peer review only - http://bmjopen.bmj.com/site/about/guidelines.xhtml

For peer review only

Paternal history of COPD	3.0		
No		81.7	84.2
Yes		10.3	10.6
Don't know		5.0	5.2
Age at menarche, years	3.3		
<12		19.4	20.0
12-15		71.7	74.1
>15		5.7	5.9
Menopause status	16.1		
No		23.4	26.4
Yes		60.5	73.6
Age at natural menopause, years	20.8		
<47		11.4	20.7
47-49		8.9	12.8
50-52		19.7	23.7
>52		15.8	16.3
Did not undergo natural menopause		23.4	26.4
Parity	1.9		
Nulliparous		14.7	15.0
Parous		83.4	85.0
1		11.9	12.2
2		33.7	34.4
3		24.8	25.3
>3		12.9	13.2
PCOS/ovarian cysts	0	1.7	1.7

For peer review only

For peer review only - http://bmjopen.bmj.com/site/about/guidelines.xhtml

Endometriosis	0	1.5	1.5
Oral contraception use, years	0.5		
Never		18.9	19.0
Ever		80.6	81.0
1		11.0	11.1
2-4		18.6	18.7
5-9		17.7	17.7
10-15		17.4	17.4
>15		16.0	16.0
Hormone replacement therapy, years	5.7		
Never		61.4	63.7
Ever		32.9	36.3
1-2		10.7	11.8
3-5		8.4	9.1
6-10		5.7	6.2
>10		8.2	9.2
Gynaecological surgery	1.8		
No		80.4	81.7
Hysterectomy		9.9	10.1
Bilateral oophorectomy		0.4	0.4
Both		7.6	7.8

SE, standard error; A levels, Advanced levels; AS levels, Advanced Subsidiary levels; O levels, Ordinary levels; GCSE, General Certificate of Secondary Educations; CSE, certificate of secondary education; NVQ, national vocational qualifications; HND, higher national diploma; HNC, higher national certificate; COPD, chronic obstructive pulmonary disease; PCOS, polycystic ovary syndrome

[a]Spirometry measures were standardised by age, sex and height

For peer review only - http://bmjopen.bmj.com/site/about/guidelines.xhtml

For peer review only

Table 2.

Associations of female reproductive health with incident COPD-related hospitalisation/death during follow-up (N=271 271)

Reproductive health indicator	% of participants	Person-years of follow-up	Number of COPD events	Age-adjusted		Multiple-adjusted[a]	
				Hazard ratio	95% CI	Hazard ratio	95% CI
Age at menarche							
<12 years	20.0	326 027	275	1.31	1.14, 1.51	1.15	1.00, 1.32
12-15 years	74.1	1 210 146	768	1.0	NA	1.0	NA
>15 years	5.9	95 771	95	1.61	1.29, 1.99	1.37	1.11, 1.71
Menopause status							
No	26.6	437 890	97	1.0	NA	1.0	NA
Yes	73.4	1 194 055	1041	1.47	1.12, 1.94	1.07	0.82, 1.41
Age at natural menopause[b]							
<47 years	20.3	202 811	270	2.21	1.83, 2.68	1.44	1.19, 1.75
47-49 years	15.8	158 718	140	1.55	1.24, 1.94	1.25	1.00, 1.57

For peer review only - http://bmjopen.bmj.com/site/about/guidelines.xhtml

50-52 years	35.4	354 774	219	1.0	NA	1.0	NA
>52 years	28.4	283 721	161	0.86	0.69, 1.06	0.93	0.75, 1.15
Parity, binary							
Nulliparous	15.1	244 938	116	0.77	0.63, 0.93	0.92	0.76, 1.12
Parous	84.9	1 387 009	1022	1.0	NA	1.0	NA
Parity, ordinal (5)							
0	15.1	244 938	116	1.0	NA	1.0	NA
1	12.2	198 124	139	1.41	1.10, 1.81	1.06	0.82, 1.35
2	34.4	562 339	308	0.96	0.78, 1.19	0.92	0.74, 1.14
3	25.3	413 347	293	1.24	1.00, 1.54	1.06	0.85, 1.33
>3	13.1	213 195	282	2.21	1.78, 2.75	1.45	1.16, 1.82
PCOS/ovarian cysts							
No	98.3	1 604 641	1108	1.0	NA	1.0	NA
Yes	1.7	27 304	30	1.61	1.12, 2.32	1.61	1.12, 2.32
Endometriosis							
No	98.5	1 607 817	1127	1.0	NA	1.0	NA

For peer review only

For peer review only - http://bmjopen.bmj.com/site/about/guidelines.xhtml

Yes	1.5	24 131	11	0.79	0.44, 1.43	0.85	0.47, 1.53
Oral contraception							
Never used	18.9	308 641	295	1.0	NA	1.0	NA
Ever used	81.1	1 323 303	843	0.95	0.83, 1.09	0.85	0.74, 0.97
Years of oral contraception use							
0	18.9	308 641	295	1.0	NA	1.0	NA
1	11.1	180 195	137	1.05	0.85, 1.29	0.84	0.69, 1.04
2-4	18.7	306 053	246	1.05	0.88, 1.24	0.95	0.80, 1.13
5-9	17.8	290 554	181	0.92	0.76, 1.11	0.86	0.71, 1.05
10-15	17.5	285 766	153	0.84	0.69, 1.03	0.74	0.61, 0.91
>15	16.1	260 734	126	0.83	0.67, 1.04	0.75	0.60, 0.93
HRT							
Never used	63.9	1 042 083	510	1.0	NA	1.0	NA
Ever used	36.1	589 861	628	1.49	1.31, 1.69	1.15	1.01, 1.30
Years of HRT							
0	63.9	1 042 083	510	1.0	NA	1.0	NA

For peer review only - http://bmjopen.bmj.com/site/about/guidelines.xhtml

1-2	11.7	190 902	240	1.83	1.54, 2.16	1.33	1.12, 1.57
3-5	9.1	147 962	162	1.63	1.34, 1.97	1.17	0.97, 1.42
6-10	6.2	100 921	74	1.00	0.77, 1.31	0.87	0.66, 1.14
>10	9.2	150 077	152	1.27	1.04, 1.55	1.06	0.87, 1.29
Gynaecological surgery							
No	81.8	1 335 512	765	1.0	NA	1.0	NA
Hysterectomy-alone	10.1	164 869	213	1.77	1.51, 2.07	1.49	1.28, 1.74
Bilateral oophorectomy-alone	0.4	5957	6	1.56	0.70, 3.48	1.36	0.61, 3.04
Both	7.8	125 607	154	1.68	1.41, 2.00	1.42	1.19, 1.69

COPD, chronic obstructive pulmonary disease; CI, confidence interval; PCOS, polycystic ovary syndrome; HRT, hormone replacement

[a]Multiple-adjusted for age, height, BMI (log-transformed), ethnicity, education, household income, Townsend deprivation index, smoking history, maternal COPD and paternal COPD

[b]Analysed in women who experienced natural menopause before baseline (n=159 571)

For peer review only

Table 3.

Associations of female reproductive health indicators with baseline spirometry measures (N=273 441)

Reproductive health indicator	Mean change in FEV_1 z-score (95% CI)		Mean change in FVC z-score (95% CI)		Mean change in FEV_1/FVC z-score (95% CI)	
	Unadjusted	Multiple-adjusted[a]	Unadjusted	Multiple-adjusted[a]	Unadjusted	Multiple-adjusted[a]
Age at menarche						
<12 years	-0.04 (-0.05, -0.03)	-0.01 (-0.02, 0.00)	-0.05 (-0.06, -0.04)	-0.01 (-0.02, -0.01)	0.02 (0.01, 0.03)	-0.02 (-0.03, -0.01)
12-15 years	0	0	0	0	0	0
>15 years	-0.08 (-0.10, -0.06)	-0.03 (-0.05, -0.01)	-0.06 (-0.08, -0.04)	-0.03 (-0.05, -0.02)	-0.05 (-0.07, -0.02)	0.00 (-0.02, 0.02)
Menopause status						
No	0	0	0	0	0	0
Yes	-0.03 (-0.04, -0.02)	-0.04 (-0.05, -0.02)	-0.03 (-0.04, -0.02)	-0.04 (-0.06, -0.03)	0.00 (-0.01, 0.01)	0.01 (-0.01, 0.02)
Age at natural menopause[b]						
<47 years	-0.11 (-0.12, -0.09)	-0.04 (-0.05, -0.02)	-0.09 (-0.11, -0.08)	-0.04 (-0.05, -0.02)	-0.05 (-0.07, -0.04)	-0.01 (-0.02, 0.01)
47-49 years	-0.05 (-0.07, -0.04)	-0.02 (-0.04, 0.00)	-0.04 (-0.06, -0.03)	-0.02 (-0.03, 0.00)	-0.03 (-0.05, -0.02)	-0.01 (-0.03, 0.01)
50-52 years	0	0	0	0	0	0

For peer review only - http://bmjopen.bmj.com/site/about/guidelines.xhtml

>52 years	0.01 (0.00, 0.03)	0.00 (-0.01, 0.02)	0.00 (-0.02, 0.01)	0.00 (-0.02, 0.01)	0.03 (0.02, 0.05)	0.01 (0.00, 0.02)
Parity, binary						
Nulliparous	-0.02 (-0.03, -0.01)	-0.04 (-0.06, -0.03)	-0.04 (-0.05, -0.03)	-0.07 (-0.08, -0.05)	0.04 (0.03, 0.05)	0.04 (0.03, 0.05)
Parous	0	0	0	0	0	0
Parity, ordinal (5)						
0	0	0	0	0	0	0
1	-0.02 (-0.04, -0.01)	0.01 (0.00, 0.03)	-0.01 (-0.02, 0.01)	0.02 (0.01, 0.04)	-0.04 (-0.06, -0.03)	-0.02 (-0.04, 0.00)
2	0.04 (0.03, 0.06)	0.04 (0.03, 0.06)	0.06 (0.05, 0.08)	0.07 (0.06, 0.08)	-0.03 (-0.05, -0.02)	-0.04 (-0.06, -0.03)
3	0.03 (0.02, 0.05)	0.05 (0.04, 0.07)	0.05 (0.04, 0.07)	0.08 (0.06, 0.09)	-0.04 (-0.06, -0.03)	-0.04 (-0.06, -0.03)
>3	-0.03 (-0.05, -0.01)	0.05 (0.04, 0.07)	-0.01 (-0.02, 0.01)	0.08 (0.07, 0.10)	-0.07 (-0.08, -0.05)	-0.06 (-0.07, -0.04)
PCOS/ovarian cysts						
No	0	0	0	0	0	0
Yes	-0.02 (-0.05, 0.02)	-0.01 (-0.04, 0.03)	-0.05 (-0.08, -0.01)	-0.03 (-0.07, 0.00)	0.06 (0.03, 0.09)	0.05 (0.02, 0.09)
Endometriosis						
No	0	0	0	0	0	0
Yes	0.01 (-0.03, 0.04)	0.01 (-0.02, 0.05)	-0.02 (-0.06, 0.01)	-0.02 (-0.05, 0.01)	0.06 (0.02, 0.10)	0.07 (0.03, 0.10)

For peer review only - http://bmjopen.bmj.com/site/about/guidelines.xhtml

Oral contraception						
Never used	0	0	0	0	0	0
Ever used	0.07 (0.05, 0.08)	0.03 (0.01, 0.04)	0.08 (0.07, 0.09)	0.02 (0.01, 0.03)	-0.01 (-0.03, 0.00)	0.01 (0.003, 0.03)
Years of oral contraception						
0	0	0	0	0	0	0
1	0.03 (0.01, 0.04)	0.01 (-0.01, 0.03)	0.04 (0.02, 0.06)	0.01 (-0.01, 0.03)	-0.03 (-0.04, -0.01)	0.01 (-0.004, 0.03)
2-4	0.05 (0.04, 0.07)	0.02 (0.01, 0.04)	0.06 (0.05, 0.08)	0.02 (0.01, 0.03)	0.00 (-0.02, 0.01)	0.02 (0.003, 0.03)
5-9	0.08 (0.07, 0.10)	0.03 (0.02, 0.05)	0.09 (0.08, 0.10)	0.03 (0.01, 0.04)	0.00 (-0.02, 0.01)	0.02 (0.003, 0.03)
10-15	0.08 (0.07, 0.10)	0.04 (0.02, 0.05)	0.10 (0.08, 0.11)	0.04 (0.02, 0.05)	-0.02 (-0.03, 0.00)	0.01 (-0.002, 0.03)
>15	0.07 (0.05, 0.08)	0.02 (0.01, 0.04)	0.08 (0.07, 0.10)	0.02 (0.00, 0.03)	-0.03 (-0.04, -0.01)	0.01 (-0.002, 0.03)
HRT						
Never used	0	0	0	0	0	0
Ever used	-0.02 (-0.02, -0.01)	0.00 (0.00, 0.01)	-0.01 (-0.01, 0.00)	0.00 (-0.01, 0.01)	-0.02 (-0.03, -0.01)	0.02 (0.01, 0.03)
Years of HRT						
0	0	0	0	0	0	0
1-2	-0.03 (-0.04, -0.01)	0.00 (-0.01, 0.02)	-0.01 (-0.03, 0.00)	0.00 (-0.02, 0.01)	-0.03 (-0.05, -0.02)	0.02 (0.01, 0.04)

For peer review only - http://bmjopen.bmj.com/site/about/guidelines.xhtml

3-5	-0.03 (-0.04, -0.01)	0.00 (-0.01, 0.02)	-0.02 (-0.03, 0.00)	0.00 (-0.02, 0.02)	-0.02 (-0.04, 0.00)	0.02 (0.00, 0.03)
6-10	0.00 (-0.02, 0.02)	0.01 (-0.01, 0.02)	0.00 (-0.02, 0.02)	0.00 (-0.02, 0.02)	0.00 (-0.02, 0.01)	0.02 (0.00, 0.04)
>10	0.00 (-0.02, 0.01)	0.00 (-0.01, 0.02)	0.01 (0.00, 0.03)	0.00 (-0.01, 0.02)	-0.03 (-0.05, -0.01)	0.01 (-0.01, 0.03)
Gynaecological surgery						
No	0	0	0	0	0	0
Hysterectomy	-0.03 (-0.05, -0.02)	0.01 (0.00, 0.03)	-0.05 (-0.06, -0.04)	0.00 (-0.02, 0.01)	0.03 (0.01, 0.04)	0.04 (0.02, 0.06)
Bilateral oophorectomy	-0.05 (-0.12, 0.02)	-0.02 (-0.08, 0.05)	-0.07 (-0.14, 0.00)	-0.04 (-0.11, 0.03)	0.04 (-0.03, 0.12)	0.05 (-0.02, 0.12)
Both	-0.04 (-0.05, -0.02)	0.00 (-0.01, 0.02)	-0.07 (-0.08, -0.05)	-0.02 (-0.04, -0.01)	0.05 (0.04, 0.07)	0.05 (0.04, 0.07)

FEV_1, forced expiratory volume in one-second; FVC, forced vital capacity; CI, confidence interval; PCOS, polycystic ovary syndrome; HRT, hormone replacement therapy

[a]Adjusted for age, BMI (log-transformed), ethnicity, education, household income, Townsend deprivation index, smoking history, asthma, maternal COPD and paternal COPD

[b]Analysed in women who experienced natural menopause before baseline (n=161 069)

For peer review only - http://bmjopen.bmj.com/site/about/guidelines.xhtml

Table 4.

Direction of association for female reproductive health indicators with risk of COPD-related hospitalisation/death and spirometry measures

Reproductive health indicator	COPD-related hospitalisation/death	FEV_1	FVC	FEV_1/FVC
Age at menarche				
<12 years	=	=	↓	↓
12-15 years	NA	NA	NA	NA
>15 years	↑	↓	↓	=
Menopause status				
No	NA	NA	NA	NA
Yes	=	↓	↓	=
Age at natural menopause				
<47 years	↑	↓	↓	=
47-49 years	=	=	=	=
50-52 years	NA	NA	NA	NA
>52 years	=	=	=	=
Parity, binary				
Nulliparous	=	↓	↓	↑
Parous	NA	NA	NA	NA
Parity, ordinal (5)				
0	NA	NA	NA	NA
1	=	=	↑	↓
2	=	↑	↑	↓
3	=	↑	↑	↓

For peer review only - http://bmjopen.bmj.com/site/about/guidelines.xhtml

For peer review only

>3	↑	↑	↑	↓
PCOS/ovarian cysts				
No	NA	NA	NA	NA
Yes	↑	=	=	↑
Endometriosis				
No	NA	NA	NA	NA
Yes	=	=	=	↑
Oral contraception				
Never used	NA	NA	NA	NA
Ever used	↓	↑	↑	=
Years of oral contraception				
0	NA	NA	NA	NA
1	=	=	=	=
2-4	=	↑	↑	↑
5-9	=	↑	↑	↑
10-15	↓	↑	↑	=
>15	↓	↑	=	=
Hormone replacement therapy				
Never used	NA	NA	NA	NA
Ever used	↑	=	=	↑
Years of hormone replacement				
0	NA	NA	NA	NA
1-2	↑	=	=	↑
3-5	=	=	=	=
6-10	=	=	=	=

For peer review only - http://bmjopen.bmj.com/site/about/guidelines.xhtml

For peer review only

>10	=	=	=	=
Gynaecological surgery				
No	NA	NA	NA	NA
Hysterectomy	↑	=	=	↑
Bilateral oophorectomy	=	=	=	=
Both	↑	=	↓	↑

COPD, chronic obstructive pulmonary disease; FEV_1, forced expiratory volume in one-second; FVC, forced vital capacity; PCOS, polycystic ovary syndrome; NA, not applicable where the category is the reference group

For peer review only

For peer review only - http://bmjopen.bmj.com/site/about/guidelines.xhtml

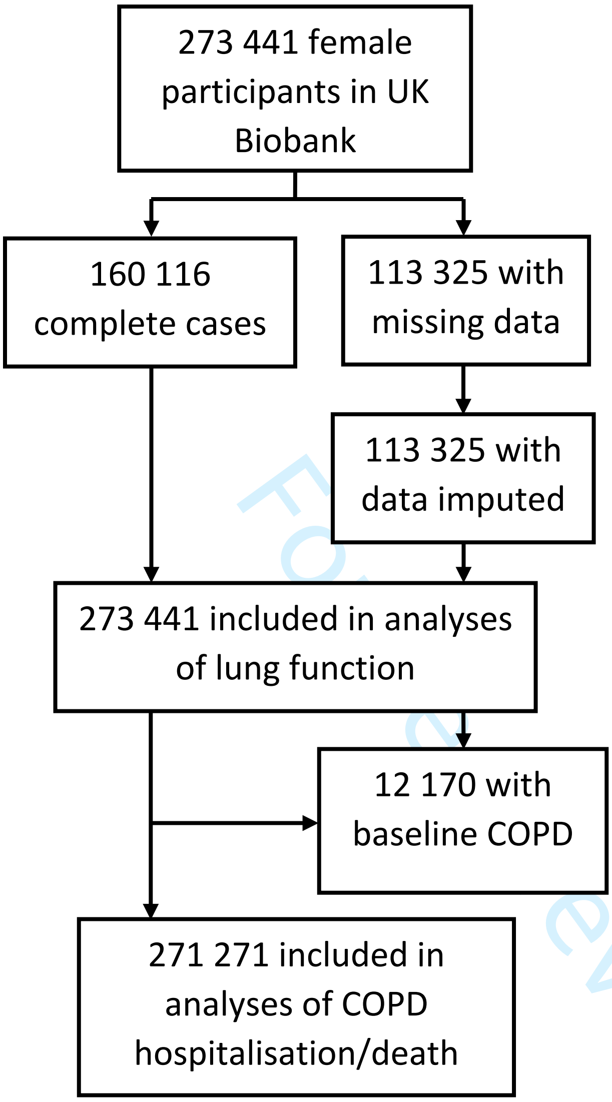

For peer review only

Online Supplementary File

Table S1. Variables used in multiple imputation model

Variable	Type of variable	Regression model to predict missing in this variable	How variable was entered when used to predict missing in other variables
Incident COPD hospitalisation/death	Dichotomous	Logistic regression	1 indicator variable
FEV_1 z-score	Continuous	Linear regression	Continuous
FVC z-score	Continuous	Linear regression	Continuous
FEV_1/FVC z-score	Continuous	Linear regression	Continuous
Age at menarche, years	Categorical (3)	Ordered logistic regression	2 indicator variables
Menopause status	Categorical (3)	Polytomous (unordered) regression	2 indicator variables
Age at menopause, years	Categorical (4)	Ordered logistic regression	3 indicator variables
Parity	Categorical (5)	Ordered logistic regression	4 indicator variables
History of ovarian cysts or polycystic ovary syndrome	Dichotomous	Logistic regression	Dichotomous
History of endometriosis	Dichotomous	Logistic regression	Dichotomous
Oral contraception ever use	Dichotomous	Passively imputed using years of OC use	N/A
Years of oral contraception	Categorical (6)	Ordered logistic regression	5 indicator variables
Gynaecological surgery	Categorical (4)	Polytomous (unordered) regression	3 indicator variables
HRT ever use	Dichotomous	Passively imputed using years of HRT use	N/A
Years of HRT use	Categorical (6)	Polytomous (unordered) regression	5 indicator variables

For peer review only - http://bmjopen.bmj.com/site/about/guidelines.xhtml

Age, years	Continuous	Linear regression	Continuous
Height, cm	Continuous	Linear regression	Continuous
Log body mass index	Continuous	Linear regression	Continuous
Ethnicity	Dichotomous	Logistic regression	Dichotomous
Qualifications	Categorical (6)	Polytomous (unordered) regression	6 indicator variables
Income	Categorical (6)	Polytomous (unordered) regression	5 indicator variables
Townsend deprivation index	Continuous	Linear regression	Continuous
Smoking pack-years	Categorical (5)	Ordered logistic regression	4 indicator variables
Maternal history of COPD	Dichotomous	Logistic regression	Dichotomous
Paternal history of COPD	Dichotomous	Logistic regression	Dichotomous
Asthma at baseline	Dichotomous	Logistic regression	Dichotomous

COPD, chronic obstructive pulmonary disease; FEV_1, forced expiratory volume in one-second; FVC, forced vital capacity; HRT, hormone replacement therapy

For peer review only

For peer review only - http://bmjopen.bmj.com/site/about/guidelines.xhtml

Table S2. Characteristics in UK Biobank women with and without spirometry data

Characteristic	Mean (SE) or % in women with spirometry	Mean (SE) or % in women without spirometry	*P* value
N	195 824	77 617	
Age (years)	56.18 (0.02)	56.78 (0.03)	<0.001
<55	20.9	20.3	
55-59	23.0	20.2	
60-64	30.5	29.7	
65 or higher	25.6	29.7	
Missing	0	0	
Height (cm)	162.6 (0.01)	162.0 (0.02)	<0.001
Missing	0.1	2.5	
Body mass index (kg/m^2)			<0.001
<18.5	0.6	1.2	
18.5-24.9	38.3	36.1	
25.0-29.9	37.5	35.7	
>29.9	23.4	25.3	
Missing	0.2	1.6	
Ethnicity			<0.001
White	95.0	92.2	
Other	4.7	7.1	
Missing	0.3	0.8	
Education			<0.001
College, university or other professional degree	37.9	34.4	
A levels, AS levels or equivalent	12.2	10.6	
O levels, GCSEs or equivalent	23.5	22.7	
CSE or equivalent	5.3	5.6	
NVQ, HND, HNC or equivalent	4.4	4.8	
Other	15.7	20.1	
Missing	1.0	1.8	
Annual household income (£)			<0.001
<18 000	19.2	23.4	

For peer review only - http://bmjopen.bmj.com/site/about/guidelines.xhtml

>18 000 and <31 000	21.7	21.6	
>31 000 and <52 000	21.7	19.0	
>52 000 and <100 000	16.3	12.8	
>100 000	4.3	3.0	
Do not know/prefer not to answer	16.4	19.5	
Missing	0.3	0.7	
Townsend deprivation index	-1.41 (0.01)	-1.14 (0.01)	<0.001
Missing	0.1	0.1	
Smoking history (pack-years)			<0.001
None	58.4	61.4	
<11	8.3	6.8	
11-20	7.7	6.7	
21-30	5.4	4.9	
>30	5.7	6.2	
Missing	14.5	14.0	
Incident COPD hospitalisation/death	0.3	0.6	<0.001
Missing	0.6	1.2	
Prevalent COPD at baseline	0.6	1.2	<0.001
Missing	<0.1	0	
Asthma			<0.001
No	86.9	87.6	
Yes	13.1	12.4	
Missing	<0.1	0	
Maternal history of COPD			<0.001
No	90.3	89.4	
Yes	6.0	5.8	
Don't know	2.0	2.4	
Missing	1.7	2.4	
Paternal history of COPD			<0.001
No	82.2	80.6	
Yes	10.2	10.3	
Don't know	4.8	5.5	
Missing	2.8	3.6	
Age at menarche			<0.001

For peer review only

For peer review only - http://bmjopen.bmj.com/site/about/guidelines.xhtml

<12	19.3	19.5	
12-15	72.2	70.3	
>15	5.5	6.2	
Missing	3.0	4.0	
Menopause status			<0.001
No	24.0	22.1	
Yes	60.3	61.0	
Missing	15.7	16.9	
Age at natural menopause			<0.001
<47	11.5	12.6	
47-49	9.4	9.4	
50-52	21.4	20.9	
>52	17.1	16.9	
Did not undergo natural menopause	35.6	34.6	
Missing	4.9	5.7	
Parity, binary			<0.001
Nulliparous	14.5	15.4	
Parous	83.8	82.3	
Parity, ordinal (5)			<0.001
0	14.5	15.4	
1	11.9	11.9	
2	34.1	32.7	
3	25.1	24.1	
>3	12.7	13.5	
Missing	1.7	2.4	
PCOS/ovarian cysts	1.7	1.7	0.60
Missing	0	0	
Endometriosis	1.5	1.5	0.81
Missing	0	0	
Oral contraception ever used	81.9	77.4	<0.001
Years of oral contraception use			<0.001
0	17.7	21.6	
1	10.8	11.6	

For peer review only

For peer review only - http://bmjopen.bmj.com/site/about/guidelines.xhtml

2-4	18.6	18.6	
5-9	18.1	16.5	
10-15	17.9	16.2	
>15	16.5	14.6	
Missing	0.3	1.0	
HRT ever used	37.8	38.7	<0.001
Years of HRT			<0.001
0	61.9	60.3	
1-2	10.5	11.1	
3-5	8.3	8.6	
6-10	5.7	5.5	
>10	8.2	8.2	
Missing	5.5	6.3	
Gynaecological surgery			<0.001
No	81.2	78.5	
Hysterectomy	9.6	10.5	
Bilateral oophorectomy	0.4	0.4	
Both	7.3	8.2	
Missing	1.5	2.4	

SE, standard error; A levels, Advanced levels; AS levels, Advanced Subsidiary levels; O levels, Ordinary levels; GCSE, General Certificate of Secondary Educations; CSE, certificate of secondary education; NVQ, national vocational qualifications; HND, higher national diploma; HNC, higher national certificate; COPD, chronic obstructive pulmonary disease; PCOS, polycystic ovary syndrome; HRT, hormone replacement therapy

For peer review only

For peer review only - http://bmjopen.bmj.com/site/about/guidelines.xhtml

Table S3. Associations of female reproductive health with incident COPD-related hospitalisation/death during follow-up in **women with $FEV_1/FVC \geq 0.7$** (N=246 539)

Reproductive health indicator	% of participants	Person-years of follow-up	Number of COPD events	Age-adjusted Hazard ratio	Age-adjusted 95% CI	Multiple-adjusted[a] Hazard ratio	Multiple-adjusted[a] 95% CI
Age at menarche							
<12 years	20.0	297 620	188	1.39	1.17, 1.65	1.19	1.00, 1.41
12-15 years	74.1	1 100 421	496	1.0	NA	1.0	NA
>15 years	5.9	86 105	62	1.63	1.25, 2.13	1.39	1.06, 1.81
Menopause status							
No	27.6	412 698	72	1.0	NA	1.0	NA
Yes	72.4	1 071 448	674	1.63	1.15, 2.30	1.00	0.73, 1.38
Age at natural menopause [b]							
<47 years	20.1	179 987	168	2.13	1.67, 2.73	1.46	1.15, 1.86
47-49 years	15.8	141 950	89	1.43	1.07, 1.91	1.29	0.97, 1.71
50-52 years	35.5	317 530	139	1.0	NA	1.0	NA
>52 years	28.6	254 316	109	0.98	0.75, 1.28	0.99	0.76, 1.29
Parity, binary							
Nulliparous	15.3	226 001	81	0.81	0.64, 1.02	0.95	0.75, 1.21
Parous	84.7	1 258 145	665	1.0	NA	1.0	NA
Parity, ordinal (5)							
0	15.3	226 001	81	1.0	NA	1.0	NA
1	12.2	180 449	86	1.27	0.94, 1.72	1.00	0.73, 1.35

For peer review only - http://bmjopen.bmj.com/site/about/guidelines.xhtml

For peer review only

2	34.4	511 071	206	0.94	0.72, 1.22	0.90	0.69, 1.17
3	25.2	374 838	190	1.16	0.89, 1.51	1.02	0.78, 1.33
>3	12.9	191 787	183	2.10	1.61, 2.74	1.40	1.07, 1.84
PCOS/ovarian cysts							
No	98.3	1 458 982	724	1.0	NA	1.0	NA
Yes	1.7	25 164	22	1.77	1.16, 2.71	1.77	1.16, 2.71
Endometriosis							
No	98.5	1 461 720	738	1.0	NA	1.0	NA
Yes	1.5	22 426	8	0.85	0.42, 1.70	0.89	0.44, 1.79
Oral contraception							
Never used	18.7	277 292	207	1.0	NA	1.0	NA
Ever used	81.3	1 206 854	539	0.84	0.71, 0.99	0.78	0.66, 0.92
Years of oral contraception							
0	18.7	277 292	207	1.0	NA	1.0	NA
1	11.1	163 663	90	0.97	0.75, 1.24	0.79	0.61, 1.01
2-4	18.7	277 839	164	0.97	0.79, 1.19	0.91	0.74, 1.12
5-9	17.8	264 919	109	0.76	0.60, 0.97	0.74	0.58, 0.94
10-15	17.6	261 462	100	0.75	0.59, 0.96	0.70	0.55, 0.90
>15	16.2	238 970	76	0.69	0.53, 0.91	0.65	0.50, 0.86
HRT							
Never used	64.6	957 821	333	1.0	NA	1.0	NA
Ever used	35.4	526 325	413	1.57	1.35, 1.84	1.20	1.03, 1.40

For peer review only - http://bmjopen.bmj.com/site/about/guidelines.xhtml

Years of HRT							
0	64.6	957 821	333	1.0	NA	1.0	NA
1-2	11.5	170 854	163	1.99	1.62, 2.43	1.42	1.16, 1.73
3-5	8.9	132 680	111	1.75	1.39, 2.20	1.26	1.00, 1.58
6-10	6.1	90 379	45	0.95	0.68, 1.33	0.83	0.59, 1.17
>10	8.9	132 413	94	1.28	1.00, 1.65	1.06	0.83, 1.37
Gynaecological surgery							
No	81.8	1 215 189	487	1.0	NA	1.0	NA
Hysterectomy	10.1	149 199	144	1.93	1.60, 2.34	1.57	1.30, 1.90
Bilateral oophorectomy	0.4	5454	6	2.36	1.05, 5.28	1.98	0.88, 4.45
Both	7.8	114 303	109	1.86	1.50, 2.30	1.52	1.23, 1.89

COPD, chronic obstructive pulmonary disease; FEV_1, forced expiratory volume in one-second; FVC, forced vital capacity; CI, confidence interval; PCOS, polycystic ovary syndrome; HRT, hormone replacement therapy; NA, not applicable

[a]Multiple-adjusted for age, height, BMI (log-transformed), ethnicity, education, household income, Townsend deprivation index, smoking history, maternal COPD and paternal COPD

[b]Analysed in women who experienced natural menopause before baseline (n=142 505)

For peer review only - http://bmjopen.bmj.com/site/about/guidelines.xhtml

For peer review only

Table S4. Associations of female reproductive health with incident COPD-related hospitalisation/death during follow-up with and without adjusting for **baseline smoking status** (N=271 271)

Reproductive health indicator	% of participants	Person-years of follow-up	Number of COPD events	Multiple-adjusted[a]		Further adjusted[b]	
				Hazard ratio	95% CI	Hazard ratio	95% CI
Age at menarche							
<12 years	20.0	326 027	275	1.15	1.00, 1.32	1.17	1.01, 1.34
12-15 years	74.1	1 210 146	768	1.0	NA	1.0	NA
>15 years	5.9	95 771	95	1.37	1.11, 1.71	1.32	1.06, 1.64
Menopause status							
No	26.6	437 890	97	1.0	NA	1.0	NA
Yes	73.4	1 194 055	1041	1.07	0.82, 1.41	1.03	0.78, 1.36
Age at natural menopause[c]							
<47 years	20.3	202 811	270	1.44	1.19, 1.75	1.43	1.18, 1.74
47-49 years	15.8	158 718	140	1.25	1.00, 1.57	1.24	0.99, 1.56
50-52 years	35.4	354 774	219	1.0	NA	1.0	NA
>52 years	28.4	283 721	161	0.93	0.75, 1.15	0.94	0.76, 1.16
Parity, binary							
Nulliparous	15.1	244 938	116	0.92	0.76, 1.12	0.92	0.75, 1.12
Parous	84.9	1 387 009	1022	1.0	NA	1.0	NA
Parity, ordinal (5)							
0	15.1	244 938	116	1.0	NA	1.0	NA
1	12.2	198 124	139	1.06	0.82, 1.35	1.06	0.82, 1.36

For peer review only - http://bmjopen.bmj.com/site/about/guidelines.xhtml

For peer review only

2	34.4	562 339	308	0.92	0.74, 1.14	0.93	0.75, 1.15
3	25.3	413 347	293	1.06	0.85, 1.33	1.07	0.86, 1.34
>3	13.1	213 195	282	1.45	1.16, 1.82	1.43	1.15, 1.79
PCOS/ovarian cysts							
No	98.3	1 604 641	1108	1.0	NA	1.0	NA
Yes	1.7	27 304	30	1.61	1.12, 2.32	1.65	1.14, 2.37
Endometriosis							
No	98.5	1 607 817	1127	1.0	NA	1.0	NA
Yes	1.5	24 131	11	0.85	0.47, 1.53	0.84	0.46, 1.53
Oral contraception							
Never used	18.9	308 641	295	1.0	NA	1.0	NA
Ever used	81.1	1 323 303	843	0.85	0.74, 0.97	0.87	0.75, 1.00
Years of oral contraception use							
0	18.9	308 641	295	1.0	NA	1.0	NA
1	11.1	180 195	137	0.84	0.69, 1.04	0.86	0.70, 1.06
2-4	18.7	306 053	246	0.95	0.80, 1.13	0.98	0.82, 1.16
5-9	17.8	290 554	181	0.86	0.71, 1.05	0.87	0.72, 1.06
10-15	17.5	285 766	153	0.74	0.61, 0.91	0.77	0.63, 0.94
>15	16.1	260 734	126	0.75	0.60, 0.93	0.77	0.62, 0.96
HRT							
Never used	63.9	1 042 083	510	1.0	NA	1.0	NA
Ever used	36.1	589 861	628	1.15	1.01, 1.30	1.16	1.03, 1.32

For peer review only - http://bmjopen.bmj.com/site/about/guidelines.xhtml

Years of HRT							
0	63.9	1 042 083	510	1.0	NA	1.0	NA
1-2	11.7	190 902	240	1.33	1.12, 1.57	1.36	1.14, 1.61
3-5	9.1	147 962	162	1.17	0.97, 1.42	1.17	0.96, 1.42
6-10	6.2	100 921	74	0.87	0.66, 1.14	0.88	0.67, 1.15
>10	9.2	150 077	152	1.06	0.87, 1.29	1.07	0.88, 1.31
Gynaecological surgery							
No	81.8	1 335 512	765	1.0	NA	1.0	NA
Hysterectomy-alone	10.1	164 869	213	1.49	1.28, 1.74	1.47	1.26, 1.72
Bilateral oophorectomy-alone	0.4	5957	6	1.36	0.61, 3.04	1.38	0.62, 3.08
Both	7.8	125 607	154	1.42	1.19, 1.69	1.39	1.17, 1.66

COPD, chronic obstructive pulmonary disease; CI, confidence interval; PCOS, polycystic ovary syndrome; HRT, hormone replacement

[a]Multiple-adjusted for age, height, BMI (log-transformed), ethnicity, education, household income, Townsend deprivation index, smoking history, maternal COPD and paternal COPD

[b]Further-adjusted for baseline smoking status

[c]Analysed in women who experienced natural menopause before baseline (n=159 571)

For peer review only

Table S5. Associations of female reproductive health indicators with baseline spirometry measures with and without adjusting for **baseline smoking status** (N=273 441)

**Reproductive health indicator**	**Mean change in FEV_1 z-score (95% CI)**		**Mean change in FVC z-score (95% CI)**		**Mean change in FEV_1/FVC z-score (95% CI)**	
	**Multiple-adjusted[a]**	**Further adjusted[b]**	**Multiple-adjusted[a]**	**Further adjusted[b]**	**Multiple-adjusted[a]**	**Further adjusted[b]**
Age at menarche						
<12 years	-0.01 (-0.02, 0.00)	-0.01 (-0.02, 0.00)	-0.01 (-0.02, -0.01)	-0.01 (-0.02, 0.01)	-0.02 (-0.03, -0.01)	-0.01 (-0.03, 0.00)
12-15 years	0	0	0	0	0	0
>15 years	-0.03 (-0.05, -0.01)	-0.03 (-0.05, -0.01)	-0.03 (-0.05, -0.02)	-0.03 (-0.05, -0.01)	0.00 (-0.02, 0.02)	0.00 (-0.02, 0.02)
Menopause status						
No	0	0	0	0	0	0
Yes	-0.04 (-0.05, -0.02)	-0.03 (-0.05, -0.02)	-0.04 (-0.06, -0.03)	-0.04 (-0.05, -0.02)	0.01 (-0.01, 0.02)	0.01 (0.00, 0.03)
Age at natural menopause[c]						
<47 years	-0.04 (-0.05, -0.02)	-0.03 (-0.05, -0.02)	-0.04 (-0.05, -0.02)	-0.04 (-0.05, -0.02)	-0.01 (-0.02, 0.01)	0.00 (-0.02, 0.01)
47-49 years	-0.02 (-0.04, 0.00)	-0.02 (-0.04, 0.00)	-0.02 (-0.03, 0.00)	-0.02 (-0.03, 0.00)	-0.01 (-0.03, 0.01)	-0.01 (-0.03, 0.01)
50-52 years	0	0	0	0	0	0
>52 years	0.00 (-0.01, 0.02)	0.00 (-0.01, 0.02)	0.00 (-0.02, 0.01)	0.00 (-0.02, 0.01)	0.01 (0.00, 0.02)	0.01 (-0.01, 0.02)
Parity, binary						
Nulliparous	-0.04 (-0.06, -0.03)	-0.04 (-0.06, -0.03)	-0.07 (-0.08, -0.05)	-0.07 (-0.08, -0.06)	0.04 (0.03, 0.05)	0.04 (0.03, 0.05)
Parous	0	0	0	0	0	0
Parity, ordinal (5)						
0	0	0	0	0	0	0

For peer review only - http://bmjopen.bmj.com/site/about/guidelines.xhtml

1	0.01 (0.00, 0.03)	0.01 (0.00, 0.03)	0.02 (0.01, 0.04)	0.03 (0.01, 0.04)	-0.02 (-0.04, 0.00)	-0.02 (-0.04, 0.00)
2	0.04 (0.03, 0.06)	0.04 (0.03, 0.06)	0.07 (0.06, 0.08)	0.07 (0.06, 0.08)	-0.04 (-0.06, -0.03)	-0.04 (-0.06, -0.03)
3	0.05 (0.04, 0.07)	0.05 (0.04, 0.07)	0.08 (0.06, 0.09)	0.08 (0.06, 0.09)	-0.04 (-0.06, -0.03)	-0.04 (-0.06, -0.03)
>3	0.05 (0.04, 0.07)	0.06 (0.04, 0.07)	0.08 (0.07, 0.10)	0.09 (0.07, 0.10)	-0.06 (-0.07, -0.04)	-0.05 (-0.07, -0.03)
PCOS/ovarian cysts						
No	0	0	0	0	0	0
Yes	-0.01 (-0.04, 0.03)	-0.01 (-0.04, 0.02)	-0.03 (-0.07, 0.00)	-0.04 (-0.07, 0.00)	0.05 (0.02, 0.09)	0.06 (0.03, 0.09)
Endometriosis						
No	0	0	0	0	0	0
Yes	0.01 (-0.02, 0.05)	0.01 (-0.03, 0.04)	-0.02 (-0.05, 0.01)	-0.03 (-0.06, 0.01)	0.07 (0.03, 0.10)	0.07 (0.03, 0.10)
Oral contraception						
Never used	0	0	0	0	0	0
Ever used	0.03 (0.01, 0.04)	0.02 (0.01, 0.04)	0.02 (0.01, 0.03)	0.02 (0.01, 0.03)	0.01 (0.00, 0.03)	0.01 (0.00, 0.03)
Years of oral contraception						
0	0	0	0	0	0	0
1	0.01 (-0.01, 0.03)	0.01 (-0.01, 0.03)	0.01 (-0.01, 0.03)	0.01 (-0.01, 0.02)	0.01 (0.00, 0.03)	0.01 (0.00, 0.03)
2-4	0.02 (0.01, 0.04)	0.02 (0.01, 0.04)	0.02 (0.01, 0.03)	0.02 (0.00, 0.03)	0.02 (0.00, 0.03)	0.01 (0.00, 0.03)
5-9	0.03 (0.02, 0.05)	0.03 (0.01, 0.04)	0.03 (0.01, 0.04)	0.02 (0.01, 0.04)	0.02 (0.00, 0.03)	0.02 (0.00, 0.03)
10-15	0.04 (0.02, 0.05)	0.04 (0.02, 0.05)	0.04 (0.02, 0.05)	0.03 (0.02, 0.05)	0.01 (0.00, 0.03)	0.01 (0.00, 0.03)

For peer review only - http://bmjopen.bmj.com/site/about/guidelines.xhtml

For peer review only

>15	0.02 (0.01, 0.04)	0.02 (0.01, 0.04)		0.02 (0.00, 0.03)	0.02 (0.00, 0.03)	0.01 (0.00, 0.03)	0.01 (0.00, 0.03)
HRT							
Never used	0	0		0	0	0	0
Ever used	0.00 (0.00, 0.01)	0.00 (-0.01, 0.01)		0.00 (-0.01, 0.01)	0.00 (-0.01, 0.01)	0.02 (0.01, 0.03)	0.02 (0.01, 0.03)
Years of HRT							
0	0	0		0	0	0	0
1-2	0.00 (-0.01, 0.02)	0.00 (-0.01, 0.02)		0.00 (-0.02, 0.01)	0.00 (-0.02, 0.01)	0.02 (0.01, 0.04)	0.02 (0.01, 0.04)
3-5	0.00 (-0.01, 0.02)	0.01 (-0.01, 0.02)		0.00 (-0.02, 0.02)	0.00 (-0.01, 0.02)	0.02 (0.00, 0.03)	0.02 (0.00, 0.03)
6-10	0.01 (-0.01, 0.02)	0.01 (-0.01, 0.02)		0.00 (-0.02, 0.02)	0.00 (-0.02, 0.02)	0.02 (0.00, 0.04)	0.02 (0.00, 0.04)
>10	0.00 (-0.01, 0.02)	0.00 (-0.01, 0.02)		0.00 (-0.01, 0.02)	0.00 (-0.01, 0.02)	0.01 (-0.01, 0.03)	0.01 (0.01, 0.03)
Gynaecological surgery							
No	0	0	NA	0	0	0	0
Hysterectomy	0.01 (0.00, 0.03)	0.02	0.00, 0.03	0.00 (-0.02, 0.01)	0.00 (-0.01, 0.01)	0.04 (0.02, 0.06)	0.04 (0.03, 0.06)
Bilateral oophorectomy	-0.02 (-0.08, 0.05)	-0.02	0.09, 0.05	-0.04 (-0.11, 0.03)	-0.04 (-0.01, 0.03)	0.05 (-0.02, 0.12)	0.04 (-0.04, 0.12)
Both	0.00 (-0.01, 0.02)	0.004	0.01, 0.02	-0.02 (-0.04, -0.01)	-0.02 (-0.04, 0.00)	0.05 (0.04, 0.07)	0.05 (0.04, 0.07)

FEV1, forced expiratory volume in one-second; FVC, forced vital capacity; CI, confidence interval; PCOS, polycystic ovary syndrome; HRT, hormone replacement therapy

[a]Adjusted for age, BMI (log-transformed), ethnicity, education, household income, Townsend deprivation index, smoking history, asthma, maternal COPD and paternal COPD

For peer review only - http://bmjopen.bmj.com/site/about/guidelines.xhtml

For peer review only

[b]Further-adjusted for baseline smoking status

[c]Analysed in women who experienced natural menopause before baseline (n=161 069)

For peer review only

For peer review only - http://bmjopen.bmj.com/site/about/guidelines.xhtml

Table S6. Associations of female reproductive health with incident COPD-related hospitalisation/death during follow-up in **complete cases** (N=220 773)

Reproductive health indicator	% of participants[a]	Person-years of follow-up	Number of COPD events	Age-adjusted Hazard ratio	Age-adjusted 95% CI	Multiple-adjusted[b] Hazard ratio	Multiple-adjusted[b] 95% CI
Age at menarche							
<12 years	19.4	257 579	222	1.37	1.17, 1.59	1.20	1.03, 1.41
12-15 years	72.1	958 578	598	1.0	NA	1.0	NA
>15 years	5.6	74 151	71	1.57	1.23, 2.01	1.34	1.05, 1.72
Menopausal status							
Pre	23.8	319 918	53	1.0	NA	1.0	NA
Post	60.4	799 833	673	1.83	1.32, 2.54	1.20	0.85, 1.68
Age at natural menopause[c]							
<47 years	32.4	174 207	224	2.12	1.75, 2.57	1.39	1.12, 1.72
47-49 years	17.9	139 602	124	1.58	1.26, 1.98	1.19	0.92, 1.53
50-52 years	28.1	314 777	194	1.0	NA	1.0	NA
>52 years	21.6	252 180	149	0.89	0.72, 1.10	0.92	0.73, 1.17
Parity, binary							
Nulliparous	15.1	200 749	98	0.81	0.65, 1.00	0.97	0.78, 1.20
Parous	83.3	1 107 959	807	1.0	NA	1.0	NA
Parity, ordinal (5)							
0	15.1	200 749	98	1.0	NA	1.0	NA
1	11.9	157 577	110	1.36	1.03, 1.78	1.00	0.76, 1.32
2	34.0	453 260	240	0.90	0.71, 1.13	0.84	0.67, 1.07

For peer review only - http://bmjopen.bmj.com/site/about/guidelines.xhtml

3	24.7	328 906	236	1.21	0.95, 1.53	1.03	0.81, 1.31
>3	12.7	168 215	221	2.11	1.66, 2.68	1.43	1.12, 1.83
PCOS/ovarian cysts							
No	98.3	1 307 646	895	1.0	NA	1.0	NA
Yes	1.7	22 016	22	1.47	0.97, 2.25	1.43	0.94, 2.19
Endometriosis							
No	98.5	1 310 012	907	1.0	NA	1.0	NA
Yes	1.5	19 651	10	0.89	0.48, 1.67	0.97	0.52, 1.80
Oral contraception							
Never used	19.1	253 792	222	1.0	NA	1.0	NA
Ever used	80.7	1 072 330	691	1.07	0.92, 1.25	0.94	0.80, 1.10
Years of oral contraception							
0	19.1	253 792	222	1.0	NA	1.0	NA
1	10.7	141 914	108	1.17	0.92, 1.47	0.93	0.74, 1.17
2-4	18.7	249 392	202	1.17	0.97, 1.42	1.04	0.86, 1.27
5-9	17.8	236 848	147	1.03	0.83, 1.27	0.94	0.76, 1.17
10-15	17.5	232 589	130	0.99	0.79, 1.23	0.85	0.68, 1.06
>15	16.0	211 587	104	0.96	0.75, 1.22	0.84	0.66, 1.07
HRT							
Never used	62.2	827 356	352	1.0	NA	1.0	NA
Ever used	37.5	497 994	562	1.78	1.55, 2.05	1.31	1.13, 1.51
Years of HRT							

For peer review only

For peer review only - http://bmjopen.bmj.com/site/about/guidelines.xhtml

0	62.2	827 356	352	1.0	NA	1.0	NA
1-2	10.5	138 603	173	2.12	1.76, 2.56	1.52	1.27, 1.83
3-5	8.3	110 741	117	1.82	1.47, 2.24	1.28	1.04, 1.58
6-10	5.6	74 932	57	1.21	0.92, 1.61	1.03	0.77, 1.36
>10	8.1	107 945	113	1.53	1.23, 1.90	1.24	1.00, 1.54
Gynaecological surgery							
No	81.0	1 077 823	603	1.0	NA	1.0	NA
Hysterectomy	9.7	129 561	163	1.75	1.47, 2.09	1.49	1.25, 1.77
Bilateral oophorectomy	0.4	4611	5	1.63	0.68, 3.94	1.45	0.60, 3.50
Both	7.5	98 833	119	1.67	1.37, 2.04	1.42	1.17, 1.74

COPD, chronic obstructive pulmonary disease; CI, confidence interval; PCOS, polycystic ovary syndrome; HRT, hormone replacement therapy; NA, not applicable

[a]Totals may not equal 100% due to missing values in this complete case analysis

[b]Multiple-adjusted for age, height, BMI (log-transformed), ethnicity, education, household income, Townsend deprivation index, smoking history, maternal COPD and paternal COPD

[c]Analysed in women who experienced natural menopause before baseline (n=129 961)

For peer review only - http://bmjopen.bmj.com/site/about/guidelines.xhtml

Table S7. Associations of female reproductive health indicators with baseline spirometry measures in **complete cases** (N=160 116)

Reproductive health indicator	Mean change in FEV_1 z-score (95% CI)		Mean change in FVC z-score (95% CI)		Mean change in FEV_1/FVC z-score (95% CI)	
	Unadjusted	Multiple-adjusted[b]	Unadjusted	Multiple-adjusted[b]	Unadjusted	Multiple-adjusted[b]
Age at menarche						
<12 years	-0.05 (-0.07, -0.04)	-0.01 (-0.03, 0.00)	-0.07 (-0.08, -0.06)	0.00 (-0.02, 0.01)	0.02 (0.01, 0.03)	-0.02 (-0.03, -0.01)
12-15 years	0	0	0	0	0	0
>15 years	-0.10 (-0.13, -0.08)	-0.04 (-0.06, -0.02)	-0.09 (-0.11, -0.07)	-0.05 (-0.07, -0.03)	-0.06 (-0.08, -0.04)	0.00 (-0.02, 0.02)
Menopause status						
No	0	0	0	0	0	0
Yes	-0.04 (-0.05, -0.03)	-0.06 (-0.08, -0.04)	-0.03 (-0.05, -0.02)	-0.06 (-0.07, -0.04)	-0.02 (-0.03, -0.01)	-0.02 (-0.03, 0.00)
Age at natural menopause[b]						
<47 years	-0.15 (-0.17, -0.13)	-0.06 (-0.07, -0.04)	-0.13 (-0.15, -0.11)	-0.06 (-0.08, -0.04)	-0.07 (-0.09, -0.05)	-0.01 (-0.03, 0.01)
47-49 years	-0.07 (-0.09, -0.05)	-0.03 (-0.05, -0.01)	-0.06 (-0.08, -0.04)	-0.03 (-0.04, -0.01)	-0.04 (-0.06, -0.02)	-0.01 (-0.03, 0.01)
50-52 years	0	0	0	0	0	0
>52 years	0.01 (-0.01, 0.02)	0.00 (-0.02, 0.02)	-0.01 (-0.03, 0.00)	-0.01 (-0.03, 0.01)	0.04 (0.03, 0.06)	0.02 (0.00, 0.03)
Parity, binary						
Nulliparous	-0.03 (-0.04, -0.01)	-0.06 (-0.07, -0.04)	-0.05 (-0.07, -0.04)	-0.08 (-0.09, -0.07)	0.05 (0.03, 0.06)	0.05 (0.03, 0.06)
Parous	0	0	0	0	0	0
Parity, ordinal (5)						
0	0	0	0	0	0	0
1	-0.03 (-0.05, -0.02)	0.01 (0.00, 0.03)	-0.01 (-0.02, 0.01)	0.03 (0.01, 0.04)	-0.06 (-0.08, -0.04)	-0.02 (-0.04, -0.01)
2	0.06 (0.04, 0.08)	0.06 (0.05, 0.07)	0.08 (0.07, 0.10)	0.09 (0.07, 0.10)	-0.03 (-0.05, -0.02)	-0.05 (-0.06, -0.03)

For peer review only - http://bmjopen.bmj.com/site/about/guidelines.xhtml

3	0.04 (0.03, 0.06)	0.07 (0.05, 0.08)	0.07 (0.05, 0.09)	0.10 (0.08, 0.11)	-0.05 (-0.06, -0.03)	-0.05 (-0.07, -0.03)
>3	-0.04 (-0.06, -0.02)	0.06 (0.04, 0.08)	-0.01 (-0.03, 0.01)	0.10 (0.08, 0.11)	-0.07 (-0.09, -0.06)	-0.06 (-0.08, -0.04)
PCOS/ovarian cysts						
No	0	0	0	0	0	0
Yes	-0.02 (-0.06, 0.02)	0.00 (-0.04, 0.03)	-0.06 (-0.09, -0.02)	-0.03 (-0.07, 0.00)	0.07 (0.03, 0.11)	0.07 (0.03, 0.11)
Endometriosis						
No	0	0	0	0	0	0
Yes	-0.01 (-0.05, 0.04)	0.00 (-0.03, 0.04)	-0.04 (-0.08, 0.01)	-0.03 (-0.07, 0.01)	0.06 (0.02, 0.10)	0.06 (0.03, 0.10)
Oral contraception						
Never used	0	0	0	0	0	0
Ever used	0.08 (0.07, 0.09)	0.03 (0.02, 0.04)	0.09 (0.08, 0.11)	0.03 (0.02, 0.04)	-0.01 (-0.02, 0.00)	0.02 (0.01, 0.03)
Years of oral contraception						
0	0	0	0	0	0	0
1	0.04 (0.02, 0.06)	0.02 (0.00, 0.03)	0.05 (0.03, 0.07)	0.01 (-0.01, 0.03)	-0.03 (-0.05, -0.01)	0.01 (-0.01, 0.03)
2-4	0.07 (0.05, 0.08)	0.03 (0.01, 0.05)	0.07 (0.06, 0.09)	0.02 (0.01, 0.04)	0.00 (-0.01, 0.02)	0.02 (0.01, 0.04)
5-9	0.10 (0.08, 0.11)	0.04 (0.02, 0.05)	0.11 (0.09, 0.12)	0.03 (0.02, 0.05)	0.00 (-0.01, 0.02)	0.02 (0.00, 0.04)
10-15	0.10 (0.09, 0.12)	0.05 (0.03, 0.06)	0.12 (0.11, 0.14)	0.05 (0.03, 0.06)	-0.02 (-0.03, 0.00)	0.02 (0.00, 0.03)
>15	0.08 (0.07, 0.10)	0.03 (0.01, 0.04)	0.10 (0.08, 0.12)	0.02 (0.01, 0.04)	-0.03 (-0.05, -0.01)	0.01 (-0.01, 0.03)
HRT						
Never used	0	0	0	0	0	0
Ever used	-0.03 (-0.04, -0.02)	0.00 (-0.01, 0.01)	-0.01 (-0.02, 0.00)	-0.01 (-0.02, 0.00)	-0.04 (-0.05, -0.03)	0.02 (0.01, 0.03)
Years of HRT						

For peer review only - http://bmjopen.bmj.com/site/about/guidelines.xhtml

0	0	0	0	0	0	0
1-2	-0.03 (-0.05, -0.02)	0.00 (-0.02, 0.01)	-0.02 (-0.03, 0.00)	-0.01 (-0.03, 0.01)	-0.05 (-0.06, -0.03)	0.02 (0.00, 0.03)
3-5	-0.04 (-0.06, -0.02)	0.00 (-0.02, 0.02)	-0.03 (-0.05, -0.01)	0.00 (-0.02, 0.02)	-0.04 (-0.05, -0.02)	0.01 (-0.01, 0.03)
6-10	0.00 (-0.02, 0.02)	0.01 (-0.01, 0.03)	0.01 (-0.02, 0.03)	0.00 (-0.02, 0.02)	-0.01 (-0.03, 0.01)	0.02 (0.00, 0.04)
>10	-0.01 (-0.03, 0.01)	0.00 (-0.02, 0.02)	0.01 (-0.01, 0.03)	0.00 (-0.02, 0.02)	-0.03 (-0.05, -0.02)	0.01 (-0.01, 0.03)
Gynaecological surgery						
No	0	0	0	0	0	0
Hysterectomy	-0.04 (-0.06, -0.03)	0.02 (0.00, 0.04)	-0.06 (-0.08, -0.05)	0.00 (-0.01, 0.02)	0.03 (0.02, 0.05)	0.05 (0.03, 0.07)
Bilateral oophorectomy	-0.07 (-0.15, 0.01)	-0.05 (-0.13, 0.03)	-0.10 (-0.18, -0.01)	-0.08 (-0.16, 0.00)	0.05 (-0.03, 0.14)	0.06 (-0.02, 0.14)
Both	-0.05 (-0.07, -0.03)	0.00 (-0.02, 0.02)	-0.08 (-0.10, -0.06)	-0.03 (-0.04, -0.01)	0.06 (0.04, 0.08)	0.06 (0.04, 0.08)

FEV_1, forced expiratory volume in one-second; FVC, forced vital capacity; CI, confidence interval; PCOS, polycystic ovary syndrome; HRT, hormone replacement therapy; NA, not applicable

[a]Adjusted for age, BMI (log-transformed), ethnicity, education, household income, Townsend deprivation index, smoking history, asthma, maternal COPD and paternal COPD

[b]Analysed in women who experienced natural menopause before baseline (n=121 153)

For peer review only - http://bmjopen.bmj.com/site/about/guidelines.xhtml

For peer review only

STROBE 2007 (v4) Statement—Checklist of items that should be included in reports of *cohort studies*

Section/Topic	Item #	Recommendation	Reported on page #
**Title and abstract**	1	(*a*) Indicate the study's design with a commonly used term in the title or the abstract	1
		(*b*) Provide in the abstract an informative and balanced summary of what was done and what was found	2
**Introduction**			
Background/rationale	2	Explain the scientific background and rationale for the investigation being reported	4
Objectives	3	State specific objectives, including any prespecified hypotheses	4-5
**Methods**			
Study design	4	Present key elements of study design early in the paper	6-7
Setting	5	Describe the setting, locations, and relevant dates, including periods of recruitment, exposure, follow-up, and data collection	5
Participants	6	(*a*) Give the eligibility criteria, and the sources and methods of selection of participants. Describe methods of follow-up	5
		(*b*) For matched studies, give matching criteria and number of exposed and unexposed	
Variables	7	Clearly define all outcomes, exposures, predictors, potential confounders, and effect modifiers. Give diagnostic criteria, if applicable	5-7
Data sources/ measurement	8*	For each variable of interest, give sources of data and details of methods of assessment (measurement). Describe comparability of assessment methods if there is more than one group	5-7
Bias	9	Describe any efforts to address potential sources of bias	6-7
Study size	10	Explain how the study size was arrived at	8
Quantitative variables	11	Explain how quantitative variables were handled in the analyses. If applicable, describe which groupings were chosen and why	6-7
Statistical methods	12	(*a*) Describe all statistical methods, including those used to control for confounding	6-7
		(*b*) Describe any methods used to examine subgroups and interactions	7
		(*c*) Explain how missing data were addressed	7
		(*d*) If applicable, explain how loss to follow-up was addressed	7
		(*e*) Describe any sensitivity analyses	7
**Results**			

For peer review only

Participants	13*	(a) Report numbers of individuals at each stage of study—eg numbers potentially eligible, examined for eligibility, confirmed eligible, included in the study, completing follow-up, and analysed	8 and Figure 1
		(b) Give reasons for non-participation at each stage	8 and Figure 1
		(c) Consider use of a flow diagram	Figure 1
Descriptive data	14*	(a) Give characteristics of study participants (eg demographic, clinical, social) and information on exposures and potential confounders	8 and 21-24
		(b) Indicate number of participants with missing data for each variable of interest	8 and 21-24
		(c) Summarise follow-up time (eg, average and total amount)	8
Outcome data	15*	Report numbers of outcome events or summary measures over time	8 and 25-28
Main results	16	(*a*) Give unadjusted estimates and, if applicable, confounder-adjusted estimates and their precision (eg, 95% confidence interval). Make clear which confounders were adjusted for and why they were included	25-32
		(*b*) Report category boundaries when continuous variables were categorized	5
		(*c*) If relevant, consider translating estimates of relative risk into absolute risk for a meaningful time period	
Other analyses	17	Report other analyses done—eg analyses of subgroups and interactions, and sensitivity analyses	10 and online supplementary file
**Discussion**			
Key results	18	Summarise key results with reference to study objectives	10
**Limitations**			
Interpretation	20	Give a cautious overall interpretation of results considering objectives, limitations, multiplicity of analyses, results from similar studies, and other relevant evidence	11-12
Generalisability	21	Discuss the generalisability (external validity) of the study results	13-14
**Other information**			
Funding	22	Give the source of funding and the role of the funders for the present study and, if applicable, for the original study on which the present article is based	2

*Give information separately for cases and controls in case-control studies and, if applicable, for exposed and unexposed groups in cohort and cross-sectional studies.

Note: An Explanation and Elaboration article discusses each checklist item and gives methodological background and published examples of transparent reporting. The STROBE checklist is best used in conjunction with this article (freely available on the Web sites of PLoS Medicine at http://www.plosmedicine.org/, Annals of Internal Medicine at http://www.annals.org/, and Epidemiology at http://www.epidem.com/). Information on the STROBE Initiative is available at www.strobe-statement.org.

For peer review only

BMJ Open

Female reproductive history in relation to chronic obstructive pulmonary disease and lung function in UK Biobank: a prospective population-based cohort study

Journal:	*BMJ Open*
Manuscript ID	bmjopen-2019-030318.R1
Article Type:	Original research
Date Submitted by the Author:	25-Jul-2019
Complete List of Authors:	Tang, Rosalind; University of Bristol Faculty of Health Sciences<br>Fraser, Abigail; University of Bristol, MRC Integrative Epidemiology Unit; University of Bristol, Department of Population Health Sciences<br>Magnus, Maria; University of Bristol, Department of Population Health Sciences; Norwegian Institute of Public Health, Centre for Fertility and Health
**Primary Subject Heading**:	Respiratory medicine
Secondary Subject Heading:	Reproductive medicine
Keywords:	chronic obstructive pulmonary disease, spirometry, menarche, menopause, oral contraceptives, hormone replacement therapy

SCHOLARONE™
Manuscripts

Female reproductive history in relation to chronic obstructive pulmonary disease and lung function in UK Biobank: a prospective population-based cohort study

Authors: Rosalind Tang[1,2]*, Abigail Fraser[3,4], Maria C. Magnus[3,4,5]

Author affiliations: [1]Bristol Medical School, Faculty of Health Sciences, University of Bristol, Bristol, United Kingdom; [2]Keenan Research Centre for Biomedical Science, St. Michael's Hospital, Toronto, Canada; [3]MRC Integrative Epidemiology Unit at the University of Bristol, Bristol, United Kingdom; [4]Department of Population Health Sciences, Bristol Medical School, University of Bristol, Bristol, United Kingdom; [5]Centre for Fertility and Health, Norwegian Institute of Public Health, Oslo, Norway.

***Corresponding author:** Rosalind Tang

Address: Musgrove Park Academy, Musgrove Park Hospital, Taunton, Somerset, TA1 5DA, United Kingdom

Email: rt16358@my.bristol.ac.uk

For peer review only

ABSTRACT

Objectives: Sex differences in respiratory physiology and predilection for developing chronic obstructive pulmonary disease (COPD) have been documented, suggesting that female sex hormones may influence pathogenesis. We investigated whether aspects of female reproductive health might play a role in risk of COPD among women.

Design: Population-based prospective cohort study.

Setting: UK Biobank recruited across 22 centres in the United Kingdom between 2006-2010.

Primary and secondary outcomes measures: We examined a range of female reproductive health indicators in relation to risk of COPD-related hospitalisation/death (n=271 271) using Cox proportional hazards regression; and lung function (n=273 441) using linear regression.

Results: Parity >3 was associated with greater risk of COPD-related hospitalisation/death (adjusted HR 1.45; 95% CI: 1.16, 1.82) and lower FEV_1/FVC (adjusted mean difference -0.06; 95% CI: -0.07, -0.04). Any oral contraception use was associated with lower risk of COPD-related hospitalisation/death (adjusted HR 0.85; 95% CI: 0.74, 0.97) and greater FEV_1/FVC (adjusted mean difference 0.01; 95% CI: 0.003, 0.03). Late menarche (age >15) and early menopause (age <47) were also associated with greater risk of COPD-related hospitalisation/death (but not lung function), while endometriosis was associated with greater FEV_1/FVC (not COPD-related hospitalisation/death). Early menarche (age <12 years) was associated with lower FEV_1/FVC (but not COPD hospitalisation/death). Associations with PCOS or ovarian cysts; any HRT use; hysterectomy-alone; and both hysterectomy and bilateral oophorectomy were in opposing directions for COPD-related hospitalisation/death (greater risk) and FEV_1/FVC (positive association).

Conclusions: Multiple female reproductive health indicators across the life course are associated with COPD-related hospitalisation/death and lung function. Further studies are necessary to understand the opposing associations of PCOS/ovarian cysts, HRT and hysterectomy with COPD and objective measures of airway obstruction.

For peer review only - http://bmjopen.bmj.com/site/about/guidelines.xhtml

Strengths and limitations of this study

- This large, population-based, prospective cohort study is the first to assess COPD risk with age at menarche, menopause status, hysterectomy, gynaecological surgery, endometriosis or PCOS.
- No study has examined a wide range of reproductive health indicators, spanning the reproductive and post-reproductive periods, with regards to COPD.
- We included a comprehensive list of potential confounders, such as age, height, body mass index, ethnicity, education, household income, Townsend deprivation index, smoking history, asthma, maternal COPD and paternal COPD.
- We relied on participants to self-report reproductive history and we cannot exclude a potential influence of selection bias, due to the low participation rate in UK Biobank.
- Data on environmental tobacco smoke were not available and we cannot exclude residual bias from this exposure.

For peer review only

For peer review only - http://bmjopen.bmj.com/site/about/guidelines.xhtml

INTRODUCTION

There are clear sex differences in lung anatomy and physiology throughout the life course.[1] This is also reflected in the risk of obstructive lung disease. For example, boys have greater risk of asthma during early childhood compared to girls, but this sex difference reverses after puberty, when girls are found to have the greater risk of new-onset asthma.[2] Chronic obstructive pulmonary disease (COPD) has traditionally been seen as a disease predominantly affecting men. However, in more recent years, the overall burden of the disease has shifted more towards women.[2 3] Women with COPD also have, on average, fewer pack-years of smoking history than men with COPD; and non-smokers with COPD are more often female.[1] This points towards a greater underlying susceptibility among women, and highlights the importance of understanding female-specific risk factors for COPD.

Female sex hormones are putative mediators of respiratory health, and regulate bronchodilation, cell proliferation, inflammation and metabolism of toxic cigarette smoke-related metabolites.[1 4-7] Notably, oestrogen and progesterone appear to have varying effects on lung function across the lifespan.[1] We know little, however, about female reproductive health indicators in relation to COPD risk.[1] These reproductive health indicators include markers of endogenous hormone exposure (age at menarche, age at menopause and length of reproductive lifespan), exogenous hormone exposure (oral contraceptive use and hormone replacement therapy), as well as pregnancy history. Of two existing studies, one study found no evidence of an association between hormone replacement therapy (HRT) and COPD risk,[8] while another reported a positive association between early age at first pregnancy (<20 years) and COPD-related mortality.[9] There is more evidence regarding the relationship between female reproductive health indicators and lung function. Early menarche,[10-12] nulliparity,[13] younger age at first pregnancy,[12] and postmenopausal status[14-19] have each been associated with reduced lung function. Evidence of the effects of HRT is contradictory.[14 15 20 21] In the present study, we therefore investigate associations of multiple female reproductive health

For peer review only - http://bmjopen.bmj.com/site/about/guidelines.xhtml

indicators across the lifespan with incidence of COPD hospitalisation/death and lung function in the UK Biobank cohort.

METHODS

Study population

Our study included female participants in UK Biobank (Figure 1), a population-based cohort study which recruited men and women age 40-69 years across 22 centres in England, Scotland and Wales between 2006-2010.[22] The response rate was 5.5%.[22] All participants provided written informed consent. Data on 273 441 women were available after withdrawals. At baseline, participants self-reported sociodemographic information, family medical history, early-life exposures, psychosocial history and medical history. Physical measurements included spirometry. Information on health outcomes after recruitment into the cohort was gathered prospectively via data linkages with hospital and death registers. Register data were available up to 28 February 2015 for England, 16 March 2015 for Wales and 28 October 2014 for Scotland. Data collection in UK Biobank was approved by the NHS National Research Ethics Service (Ref 11/NW/0382). UK Biobank does not require separate ethics approvals for specific analyses. A generic Research Tissue Bank approval for access to UK Biobank data and/or samples has been recommended by the National Research Ethics Service and obtained from the governing UK Biobank Research Ethics Committee (http://www.ukbiobank.ac.uk/wp-content/uploads/2012/09/Access-Procedures-2011-1.pdf). Applications to access the resource are approved by the UK Biobank Access Sub-Committee and Ethics & Governance Council.

Female reproductive health indicators

We investigated the following self-reported female reproductive health indicators: age at menarche (<12, 12-15, and >15 years), menopause status (no, yes), age at natural menopause (<47, 47-49, 50-52 and >52 years), parity (0, 1, 2, 3, and >3), history of polycystic ovary syndrome (PCOS) or ovarian cysts, history of endometriosis, oral contraception (OC) use (never/ever), years of OC use (0, 1, 2-4,

For peer review only - http://bmjopen.bmj.com/site/about/guidelines.xhtml

5-9, 10-15 and >15), hormone replacement therapy (HRT) use (never/ever), years of HRT use (0, 1-2, 3-5, 6-10, and >10), and gynaecological surgery (hysterectomy, oophorectomy and hysterectomy with bilateral oophorectomy). We calculated years of OC and HRT use as the differences between age at first and last use, and created categories according to quintiles.

Amaral et al. previously reported decreased lung function in postmenopausal women in UK Biobank.[16] We repeat these analyses for completeness alongside analyses of other female reproductive health indicators.

Respiratory outcomes

Prevalent COPD at baseline was identified using self-report and registrations before baseline in the hospital registers. Incident hospitalisations/deaths from COPD after baseline were identified using hospital and death registers. The following International Classification of Diseases (ICD) codes were used to capture COPD: ICD-9 codes 490-492, 494 and 496; or ICD-10 codes J40-J44.

Forced expiratory volume at one-second (FEV_1) and forced vital capacity (FVC) were measured using Vitalograph Pneumotrac 6800 (Vitalograph, UK) at baseline. Spirometry was not performed if participants had experienced a chest infection in the last month; had a lifetime history of detached retina or collapsed lung; had experienced a heart attack, eye surgery or surgery to the chest or abdomen in the last 3 months; were currently pregnant in the first or third trimester; or were currently using medication for tuberculosis. Two measurements were conducted if the difference between these measurements was ≤5%. A third measurement was conducted if the difference was >5%. We used the single greatest measurements of FEV_1 and FVC per participant. Post-bronchodilator spirometry was not available, although drug treatment was not withheld. Spirometry measurements were converted to internally standardised z-scores by age and height based on the Global Lung Initiative 2012 recommendations[23] to reduce bias related to age. These z-scores were used for all analyses.

For peer review only - http://bmjopen.bmj.com/site/about/guidelines.xhtml

Statistical analyses

We used Cox proportional hazards regression to examine the association of each female reproductive health indicator in relation to incidence of COPD hospitalisation/death. Participants without COPD at baseline were followed from enrolment until hospitalisation/death from COPD, death from other causes, or the end of follow-up if the participants were still alive and disease-free. The time axis for the Cox regression was calendar time. We examined deviations from the proportional hazard assumption using Schoenfeld residuals.[24] Age- and confounder-adjusted estimates are reported as hazard ratios (HR) with 95% confidence intervals (95% CI). The following potential confounders were included in multivariable analyses: age, height, log-transformed body mass index (BMI) at baseline, ethnicity (White or non-White), educational attainment (college, university or other professional degree; Advanced levels, Advanced Subsidiary levels or equivalent; Ordinary levels, General Certificate of Secondary Educations or equivalent; Certificate of Secondary Educations or equivalent; National Vocational Qualifications, Higher National Diploma, Higher National Certificate or equivalent; other), annual household income (<£18 000; £18 001-31 000; £31 001-£52 000; £52 001-£100 000; >£100 000), Townsend deprivation index, smoking history in pack-years (none, <11, 11-20, 21-30, >30), and parental history of COPD. We performed sensitivity analyses of 1) menopause status and age at natural menopause with further adjustment for HRT; 2) menopause status excluding participants age <45 years or >60 years at baseline to reduce potential bias by age; 3) all reproductive health indicators after excluding participants with respiratory conditions (i.e. COPD, asthma, tuberculosis or pneumonia) reported at baseline or $FEV_1/FVC<0.7$ at baseline, as they possibly had undiagnosed COPD;[25] 4) all reproductive health indicators with smoking history adjusted as two covariates (duration in years and average number of cigarettes/day) rather than one covariate (pack-years); 5) all reproductive health indicators with further adjustment for baseline smoking status (never, previous, current); 6) all reproductive health indicators after excluding participants with any history of smoking; and 7) all reproductive health indicators with further adjustment for other comorbidities (cardiovascular disease or diabetes) reported at baseline.

For peer review only - http://bmjopen.bmj.com/site/about/guidelines.xhtml

We used linear regression in the analysis of standardised spirometry measurements. The multivariable linear regression models were adjusted for the same covariates as for hospitalisation/death from COPD, with the addition of asthma at baseline. We reported results as mean difference in the spirometry measurements z-scores with 95% CI. These mean differences in z-scores can be converted to raw values by multiplication with the standard deviation in spirometry measurement for the given age and height group. Sensitivity analyses for standardised spirometry measurements were also conducted with: 1) menopause status excluding participants age <45 years or >60 years at baseline; 2) smoking history adjusted as two covariates (duration in years and average number of cigarettes/day); 3) further adjustment for baseline smoking status; and 4) further adjustment for comorbidities baseline (cardiovascular disease or diabetes).

We performed multiple imputation by chained equations to account for missing data. Exposures, covariates and outcomes were included in the imputation model. We imputed 20 datasets, which were then analysed using Rubin's rules.[26] Details of the imputation model are given in the online supplementary file (Table S1). All analyses were performed using Stata/MP 15 (StataCorp LLC, College Station, USA). Sensitivity analyses of the complete cases was performed for comparison with our imputed data.

Patient and public involvement

The present study did not involve patients and the public.

RESULTS

Of the 273 441 women included in this study, 2170 had prevalent COPD at baseline (Figure 1). These prevalent cases were excluded from the analysis of incident COPD-related hospitalisation/death. By the end of follow-up (median 6 years), there were 1138 incident cases of hospitalisation/death from COPD, yielding an incidence rate of 0.19 per 100 000 person years.

For peer review only - http://bmjopen.bmj.com/site/about/guidelines.xhtml

For peer review only

In UK Biobank, 28.4% of women did not have spirometry data, 16.1% were uncertain of or did not report menopause status and 14.3% did not report smoking history. The proportion of missing information on the other covariates were modest (Table 1). Overall, women with spirometry data were younger, more often White, had higher educational attainment, higher annual household income, less deprivation (lower Townsend index), greater smoking history and were more likely to have asthma than women who did not undergo spirometry assessment (online supplementary file Table S2). The distribution of participants' characteristics across the imputed datasets is shown in Table 1.

Late (age >15 years) menarche was associated with increased risk of hospitalisation/death from COPD compared to menarche between age 12-15. The multiple-adjusted HR was 1.37 (95% CI: 1.11, 1.71) (Table 2). There was a modest association between early menarche and COPD hospitalisation/death (HR 1.15; 95% CI: 1.00, 1.32). Early menarche (age <12 years) was associated with lower FEV_1/FVC (Table 3).

Risk of hospitalisation/death from COPD did not differ between pre- and postmenopausal women (Table 2), and this finding was similar after further adjusting for HRT use (HR 1.02; 95% CI: 0.78, 1.35). In examining age at menopause and risk hospitalisation/death from COPD among women who experienced natural menopause (n=159 571), the risk was higher in women who experienced menopause age <47 years (HR 1.44; 95% CI: 1.19, 1.75) when compared to age 50-52 (Table 2). This association remained after further adjustment for HRT use (HR 1.43; 95% CI: 1.18, 1.74). Postmenopausal status at baseline and early natural menopause were each associated with lower FEV_1 and FVC but not FEV_1/FVC (Table 3). These associations did not change after further adjustment for HRT ever use (results not shown). After restricting the sample to participants who were age 45-60 years (inclusive) at the time of reporting menopause status to reduce residual bias due to age, we found that menopause status was positively associated with COPD-related hospitalisation/death (HR

For peer review only

1.57; 95% CI: 1.07, 2.30), whilst associations with spirometry measures did not change (results not shown).

Hospitalisation/death from COPD did not differ between parous and nulliparous women, but parity >3 was associated with increased risk (HR 1.45; 95% CI: 1.16, 1.82) compared to nulliparity. Women who were nulliparous had lower FEV_1 and FVC but greater FEV_1/FVC compared to parous women, while women with parity >3 had greater FEV_1 and FVC but lower FEV_1/FVC compared to nulliparous women (Table 3).

A history of PCOS or ovarian cysts was associated with increased risk of hospitalisation/death from COPD (HR 1.61; 95% CI: 1.12, 2.32) and greater FEV_1/FVC (mean difference 0.05; 95% CI: 0.02, 0.09), while there was no evidence of an association with FEV_1 or FVC. A history of endometriosis was not associated with COPD hospitalisation/death, FEV_1 or FVC (Tables 2 and 3), though we observed a modest positive association with FEV_1/FVC (mean difference 0.07; 95% CI: 0.03, 0.10).

We observed several associations between exogenous hormone use and COPD hospitalisation/death. Women who had ever used OC had lower risk of hospitalisation/death from COPD compared to never users (HR 0.85; 95% CI: 0.74, 0.97) (Table 2). This appeared to be driven by OC use of >9 years (Table 2). In line with the observed findings for COPD, OC ever use and duration of use showed positive associations with FEV_1, FVC and FEV_1/FVC (Table 3).

Ever using HRT was associated with a modest increased risk of hospitalisation/death from COPD (HR 1.15; 95% CI: 1.01, 1.30) (Table 2). We observed no associations of HRT ever use with FEV_1 or FVC, but a positive association with FEV_1/FVC (Table 3). When we examined the duration of HRT use, we only observed increased hospitalisation/death from COPD (HR 1.33; 95% CI: 1.12, 1.57) and greater FEV_1/FVC (mean difference 0.02; 95% CI: 0.01, 0.04) with 1-2 years of use (Tables 2 and 3). There was no evidence of a dose-response relationship.

For peer review only - http://bmjopen.bmj.com/site/about/guidelines.xhtml

A history of hysterectomy (with or without bilateral oophorectomy), but not bilateral oophorectomy-alone, were associated with greater risk of COPD hospitalisation/death (Table 2). At the same time, hysterectomy was associated with higher, rather than lower, FEV_1/FVC (Tables 3). Hysterectomy with bilateral oophorectomy was also associated with lower FVC (Table 3).

The directions of association between individual female reproductive health indicators with COPD risk and spirometry measures are summarised in Table 4. 55 929 women had reported either a respiratory illness (asthma, COPD, tuberculosis and/or pneumonia) and/or FEV_1/FVC<0.7 at baseline. However, our findings after excluding these women from our analyses of COPD-related hospitalisation/death were similar to our main findings (online supplementary file Table S3).

We report the results of our sensitivity analyses, after 1) further adjusting for baseline smoking status; 2) adjusting for smoking history as two covariates including duration in years of smoking and average number of cigarettes per day; 3) excluding women who have ever smoked; or 4) further adjusting for cardiovascular disease or diabetes reported at baseline, in the online supplementary file (Tables S4-S8). The direction and magnitude of associations remained similar for risk of COPD hospitalisation/death (online supplementary file Tables S4 and S6) and change in spirometry measures (online supplementary file Tables S7 and S8) after further adjustment for the above covariates. In our analyses of never smokers (n=161 626), however, associations of COPD-related hospitalisation/death with early menopause, parity>3, PCOS/ovarian cysts, ever using oral contraception or hormone replacement therapy became null (online supplementary file Table S5). Early age at menarche (<12 years) was also positively associated with COPD-related hospitalisation/death in this restricted sample of never smokers (HR 1.39; 95% CI: 1.02, 1.90), whilst the remaining associations remained unchanged from the main analyses.

We also investigated associations of reproductive health indicators with risk of COPD-related mortality, and not hospitalisation, among women of UK Biobank. The associations from these analyses of 273 441 women (including those with COPD reported at baseline) were null for all

For peer review only - http://bmjopen.bmj.com/site/about/guidelines.xhtml

reproductive health indicators (online supplementary file Table S9), except age at menarche <12 years compared to 12-15 years (HR 1.52; 95% CI: 1.08, 2.13). However, there were only 179 COPD-related deaths in UK Biobank and many categories of our reproductive health indicators experienced fewer than ten events. Complete-case analyses of the risk of COPD hospitalisation/death (online supplementary file Table S10) and lung function (online supplementary file Table S11) also produced similar results to those from our imputed dataset.

DISCUSSION

In this large, prospective study, several female reproductive health indicators were associated with COPD hospitalisation/death and/or lung function. Parity greater than three was associated with decreased baseline FEV_1/FVC, a marker of airway obstruction, and increased risk of hospitalisation/death from COPD during follow-up. Ever using OC was associated with greater FEV_1/FVC and lower risk of COPD hospitalisation/death. Associations of hysterectomy, HRT use, and a history of PCOS or ovarian cysts showed inconsistent findings for FEV_1/FVC and COPD hospitalisation/death. This inconsistency may be explained by the fact that spirometry was measured in participants at baseline (age 40-69) and FEV_1 and FEV_1/FVC may have declined during follow-up. Conversely, many patients diagnosed with stage 1 COPD do not go on to develop COPD symptoms or COPD resulting in hospitalisation/death.[27] There is also high intra-individual variability in FEV_1.[28] Spirometry assessment at one time point may therefore not be as accurate at identifying clinically-relevant COPD compared to register data on hospitalisation/death.

A study of 424 797 postmenopausal women in the United States reported no difference in COPD mortality by parity.[9] In contrast, our present study reports higher risk of COPD hospitalisation/death among women with more than three births compared to nulliparous women. However, we investigated both COPD-related hospitalisation and death, and we were therefore able to capture less severe COPD cases. A study of 70 965 women in the Nurses' Health Study reported no evidence of an association between HRT ever use and COPD incidence, although the investigators included

For peer review only - http://bmjopen.bmj.com/site/about/guidelines.xhtml

For peer review only

both probable (self-reported physician diagnosis along with a record of spirometry or imaging at the time of diagnosis or COPD documented on a death register) and definite (self-reported physician diagnosis and FEV_1 <80% predicted) cases of COPD.[8] Here we report a positive association between HRT use and COPD hospitalisation/death. This is the first study to assess COPD risk with regards to age at menarche, menopause status, hysterectomy, gynaecological surgery, endometriosis or PCOS.

Two previous studies reported reduced FEV_1 in adult women who experienced menarche before age 10[10 12] but neither found evidence of an association between age at menarche and FEV_1/FVC. A Mendelian randomisation study of adolescent and adult women across multiple European cohorts, including a subset from UK Biobank, estimated a 24.8 mL increase in FVC per year increase in age at menarche among adult women and a -56.6 mL decrease in FVC per year increase in age at menarche among adolescent girls.[11] This study reported no association between age at menarche and FEV_1/FVC.[11] Notably, this Mendelian randomisation study did not explore non-linear associations between age at menarche and measures of lung function. We observed lower FEV_1 and FVC in adult women with late menarche, and lower FVC and FEV_1/FVC in adult women with early menarche.

A recent systematic review, which included studies from UK Biobank,[16] reported that postmenopausal status was associated with reduced FEV_1 and FVC but not FEV_1/FVC.[18] Current HRT use has been associated with both higher[14] and lower[15] lung function. One study reported an association between HRT use and greater FEV_1/FVC.[14] Others have identified differences in lung function according to continuous/cyclic and combined/oestrogen-only HRT.[20 21] Based on the information from participants in UK Biobank, we could not stratify by HRT subtypes.

There are sex differences in lung anatomy and physiology throughout the life course.[1 2 4] During gestation, female foetuses have fewer bronchi, smaller lungs and produce surfactant earlier than male foetuses.[1] Female neonates and children have greater lung function and faster lung growth than their male counterparts, and boys are more likely to be diagnosed with asthma during childhood than girls, while girls are twice as likely as boys to be diagnosed with asthma after

For peer review only - http://bmjopen.bmj.com/site/about/guidelines.xhtml

puberty.[1] These findings suggest that female sex steroids can accelerate lung maturation and termination of lung growth, which may partially explain why early menarche associates with lower FEV_1/FVC in adulthood. During the reproductive years, parity and PCOS relate to an increase in circulating oestrogen, which has been implicated in the pathogenesis of inflammatory lung diseases including COPD.[6 29-31] Although OC use appears to be protective against COPD, some researchers have suggested that OC use attenuates the fluctuations in sex steroids, which correlates with fluctuations in endothelial nitric oxide, a potent bronchodilator.[1] Additionally, exogenous hormones may not be as influential during reproductive years but may become more influential in after menopause, when endogenous hormone production is low.[6] This is supported by our findings of opposing associations with COPD risk for OC use (lower risk) during reproductive period and HRT use (higher risk) after menopause.

The effect of female sex hormones on COPD and lung health may vary depending on the net effects on airway smooth muscle, inflammation and cigarette smoke metabolism. Oestrogens can regulate the synthesis of nitric oxide, a potent bronchodilator, within human bronchial epithelium,[1 32] as well as regulate airway bronchoconstriction in response to acetylcholine or histamine.[33 34] There is also evidence that progesterone may affect airway smooth muscle constriction, although these reports are limited to animal studies.[35] In addition to regulating bronchodilation and bronchoconstriction, in vitro studies report that oestrogen can also modulate the proliferation of airway smooth muscle cells,[36 37] which is a key feature of COPD.[38] Oestrogen's pro- and anti-inflammatory effects have been documented in other female-predominant diseases[39] and may also play a role in COPD, as chronic inflammation is another hallmark of the disease. Finally, oestrogen is an important regulator of toxic cigarette smoke metabolites through cytochrome P450 enzymes.[1] In a mouse model of COPD, Glassberg et al. reported that oestradiol administration after ovariectomy protected against cigarette smoke-induced alveolar septal destruction, macrophage infiltration and decreased other markers of inflammation compared to ovariectomy without oestradiol administration.[40] In the same mouse model, however, non-ovariectomised female mice also had increased small airway

For peer review only

For peer review only - http://bmjopen.bmj.com/site/about/guidelines.xhtml

remodelling following cigarette smoke exposure when compared to both male mice and ovariectomised female mice.[41] These timing-, dose- and cell-dependent signalling pathways may explain why exposure to female sex steroids, and oestrogen in particular, have potentially contradictory effects on respiratory health.

We cannot exclude a potential influence of selection bias, due to the low participation rate in UK Biobank. Women in the cohort are less likely to have ever smoked, have greater educational attainment, but a similar number of offspring, as compared to the general UK population (https://www.ons.gov.uk/). There is also a lower prevalence of self-reported COPD in UK Biobank (0.8%) compared to physician-diagnosed COPD in the general female UK population (1.68%).[42] Overall, women participating in UK Biobank reflect a higher-educated, more health-conscious group, with more modest underlying risk for COPD compared to the general population. We can only speculate as to how these selection factors might have influenced our associations of interest, but we propose that the most likely direction of any selection bias is towards the null (resulting in an underestimation). Strengths of this study include the large, prospective design. However, we relied on participants to self-report reproductive history which might have led to measurement error. Years of OC or HRT use were estimated as the difference between ages at first and last use. This likely resulted in overestimation of the duration of use, which might have attenuated our findings. In addition, we were unable to distinguish between reversible and non-reversible airway obstruction at baseline, as a bronchodilator was not administered before spirometry was conducted. Although we adjusted for self-reported asthma at baseline, this may have led to overestimation of COPD-related airway obstruction among participants with undiagnosed asthma. Lastly, we were unable to adjust for environmental tobacco smoke exposure as these data were not collected in UK Biobank. We therefore cannot exclude the possibility of residual bias from environmental tobacco smoke.

In conclusion, our findings indicate that several female reproductive health indicators across the life course are associated with risk of COPD hospitalisation/death and add to the indirect evidence

For peer review only - http://bmjopen.bmj.com/site/about/guidelines.xhtml

suggesting that that female reproductive hormones may have a role in COPD pathogenesis. Further studies are necessary to understand the opposing associations of hysterectomy, HRT and PCOS/ovarian cysts with COPD and objective measures of airway obstruction.

ACKNOWLEDGEMENTS

This research has been conducted using the UK Biobank Resource under Application Number 6326. We thank all the participants and researchers involved.

CONTRIBUTORS

All authors provided substantial contributions to the design of the work. RT analysed the data and drafted the manuscript. MCM and AF supervised the study, provided further data interpretation and revised the manuscript. All authors approved the final version of the manuscript and agree to be accountable for all aspects of the work in ensuring that questions related to the accuracy or integrity of any part of the work are appropriately investigated and resolved.

DATA SHARING:

Data were obtained from the UK Biobank Resource via its Access Management System (http://www.ukbiobank.ac.uk/wp-content/uploads/2012/09/Access-Procedures-2011-1.pdf).

Funding: This work was supported by the UK Medical Research Council grant MC/UU/12013/5, which provides infrastructure funding to the MRC Integrative Epidemiology Unit at the University of Bristol, and grant MR/M009351/1 as a fellowship to AF. This work was partly supported by the Research Council of Norway's Centres of Excellence scheme grant 262700.

Competing interests: None declared

For peer review only - http://bmjopen.bmj.com/site/about/guidelines.xhtml

For peer review only

REFERENCES

1. Townsend EA, Miller VM, Prakash YS. Sex differences and sex steroids in lung health and disease. *Endocr Rev* 2012;33(1):1-47.

2. Raghavan D, Jain R. Increasing awareness of sex differences in airway diseases. *Respirology* 2016;21(3):449-59.

3. Raghavan D, Varkey A, Bartter T. Chronic obstructive pulmonary disease: the impact of gender. *Curr Opin Pulm Med* 2017;23(2):117-23.

4. Gonzalez-Arenas A, Agramonte-Hevia J. Sex steroid hormone effects in normal and pathologic conditions in lung physiology. *Mini Rev Med Chem* 2012;12(11):1055-62.

5. Kamil F, Pinzon I, Foreman MG. Sex and race factors in early-onset COPD. *Curr Opin Pulm Med* 2013;19(2):140-4.

6. Sathish V, Martin YN, Prakash YS. Sex steroid signaling: implications for lung diseases. *Pharmacol Ther* 2015;150:94-108.

7. Fuentes N, Silveyra P. Endocrine regulation of lung disease and inflammation. *Exp Biol Med (Maywood)* 2018;243(17-18):1313-22.

8. Barr RG, Wentowski CC, Grodstein F, et al. Prospective study of postmenopausal hormone use and newly diagnosed asthma and chronic obstructive pulmonary disease. *Arch Intern Med* 2004;164(4):379-86.

9. Gaudet MM, Carter BD, Hildebrand JS, et al. Associations of parity and age at first pregnancy with overall and cause-specific mortality in the Cancer Prevention Study II. *Fertil Steril* 2017;107(1):179-88.e6.

10. Macsali F, Real FG, Plana E, et al. Early age at menarche, lung function, and adult asthma. *Am J Respir Crit Care Med* 2011;183(1):8-14.

11. Gill D, Sheehan NA, Wielscher M, et al. Age at menarche and lung function: a Mendelian randomization study. *Eur J Epidemiol* 2017;32(8):701-10.

For peer review only - http://bmjopen.bmj.com/site/about/guidelines.xhtml

12. Murri V, Antoniazzi F, Piazza M, et al. Lung Function in Women with Idiopathic Central Precocious Puberty: A Pilot Study. *Horm Res Paediatr* 2017;87(2):95-102.

13. Harik-Khan R, Wise RA, Lou C, et al. The effect of gestational parity on FEV1 in a group of healthy volunteer women. *Respir Med* 1999;93(6):382-8.

14. Carlson CL, Cushman M, Enright PL, et al. Hormone replacement therapy is associated with higher FEV1 in elderly women. *Am J Respir Crit Care Med* 2001;163(2):423-8.

15. Hayatbakhsh MR, Najman JM, O'Callaghan MJ, et al. Association between smoking and respiratory function before and after menopause. *Lung* 2011;189(1):65-71.

16. Amaral AF, Strachan DP, Gomez Real F, et al. Lower lung function associates with cessation of menstruation: UK Biobank data. *Eur Respir J* 2016;48(5):1288-97.

17. Triebner K, Matulonga B, Johannessen A, et al. Menopause Is Associated with Accelerated Lung Function Decline. *Am J Respir Crit Care Med* 2017;195(8):1058-65.

18. Campbell B, Davis SR, Abramson MJ, et al. Menopause, lung function and obstructive lung disease outcomes: a systematic review. *Climacteric* 2018;21(1):3-12.

19. Memoalia J, Anjum B, Singh N, et al. Decline in Pulmonary Function Tests after Menopause. *J Menopausal Med* 2018;24(1):34-40.

20. Cevrioglu AS, Fidan F, Unlu M, et al. The effects of hormone therapy on pulmonary function tests in postmenopausal women. *Maturitas* 2004;49(3):221-7.

21. Pata O, Atis S, Utku Oz A, et al. The effects of hormone replacement therapy type on pulmonary functions in postmenopausal women. *Maturitas* 2003;46(3):213-8.

22. Allen N, Sudlow C, Downey P, et al. UK Biobank: Current status and what it means for epidemiology. *Health Policy Technol* 2012;1(3):123-26.

23. Quanjer PH, Stanojevic S, Cole TJ, et al. Multi-ethnic reference values for spirometry for the 3-95-yr age range: the global lung function 2012 equations. *Eur Respir J* 2012;40(6):1324–1343.

24. Schoenfeld D. The asymptotic properties of nonparametric tests for comparing survival distributions. *Biometrika* 1981;68(1):316–319.

For peer review only - http://bmjopen.bmj.com/site/about/guidelines.xhtml

25. Global Initiative for Chronic Obstructive Lung Disease (GOLD). Global strategy for the diagnosis, management, and prevention of chronic obstructive pulmonary disease (2019 report). GOLD 2019.

26. Marshall A, Altman DG, Holder RL, et al. Combining estimates of interest in prognostic modelling studies after multiple imputation: current practice and guidelines. *BMC Med Res Methodol* 2009;9:57.

27. Rossi A, Butorac-Petanjek B, Chilosi M, et al. Chronic obstructive pulmonary disease with mild airflow limitation: current knowledge and proposal for future research - a consensus document from six scientific societies. *Int J Chron Obstruct Pulmon Dis* 2017;12:2593-610.

28. Casanova C, Aguirre-Jaime A, de Torres JP, et al. Longitudinal assessment in COPD patients: multidimensional variability and outcomes. *Eur Respir J* 2014;43(3):745-53.

29. Cushman M, Legault C, Barrett-Connor E, et al. Effect of postmenopausal hormones on inflammation-sensitive proteins: the Postmenopausal Estrogen/Progestin Interventions (PEPI) Study. *Circulation* 1999;100(7):717-22.

30. Walsh BW, Paul S, Wild RA, et al. The effects of hormone replacement therapy and raloxifene on C-reactive protein and homocysteine in healthy postmenopausal women: a randomized, controlled trial. *J Clin Endocrinol Metab* 2000;85(1):214-8.

31. Gol M, Akan P, Dogan E, et al. Effects of estrogen, raloxifene, and hormone replacement therapy on serum C-reactive protein and homocysteine levels. *Maturitas* 2006;53(3):252-9.

32. Kirsch EA, Yuhanna IS, Chen Z, et al. Estrogen acutely stimulates endothelial nitric oxide synthase in H441 human airway epithelial cells. *Am J Respir Cell Mol Biol* 1999;20(4):658-66.

33. Degano B, Mourlanette P, Valmary S, et al. Differential effects of low and high-dose estradiol on airway reactivity in ovariectomized rats. *Respir Physiol Neurobiol* 2003;138(2-3):265-74.

34. Townsend EA, Thompson MA, Pabelick CM, et al. Rapid effects of estrogen on intracellular Ca2+ regulation in human airway smooth muscle. *Am J Physiol Lung Cell Mol Physiol* 2010;298(4):L521-30.

For peer review only - http://bmjopen.bmj.com/site/about/guidelines.xhtml

35. Ynuk B. Endocrine regulation of airway contractility is overlooked. *J Endocrinol* 2014;222(2):R61-R73.

36. Stamatiou R, Paraskeva E, Papagianni M, et al. The mitogenic effect of testosterone and 17β-estradiol on airway smooth muscle cells. *Steroids* 2011;76(4):400-08.

37. Ambhore NS, Katragadda R, Raju Kalidhindi RS, et al. Estrogen receptor beta signaling inhibits PDGF induced human airway smooth muscle proliferation. *Mol Cell Endocrinol* 2018;476:37-47.

38. Yan F, Gao H, Zhao H, et al. Roles of airway smooth muscle dysfunction in chronic obstructive pulmonary disease. *J Transl Med* 2018;16(1):262.

39. Straub RH. The Complex Role of Estrogens in Inflammation. *Endocr Rev* 2007;28(5):521-74.

40. Glassberg MK, Catanuto P, Shahzeidi S, et al. Estrogen deficiency promotes cigarette smoke-induced changes in the extracellular matrix in the lungs of aging female mice. *Transl Res* 2016;178:107-17.

41. Tam A, Bates JH, Churg A, et al. Sex-Related Differences in Pulmonary Function following 6 Months of Cigarette Exposure: Implications for Sexual Dimorphism in Mild COPD. *PLoS One* 2016;11(10):e0164835.

42. Simpson CR, Hippisley-Cox J, Sheikh A. Trends in the epidemiology of chronic obstructive pulmonary disease in England: a national study of 51 804 patients. *Br J Gen Pract* 2010;60(576):277-84.

For peer review only

For peer review only - http://bmjopen.bmj.com/site/about/guidelines.xhtml

FIGURE LEGEND

Figure 1. Flow Chart of Study Population.

COPD, chronic obstructive pulmonary disease. Missing data is in one or more of the following variables: height, ethnicity, educational attainment, income, Townsend deprivation index, smoking history, spirometry measurements, maternal COPD history, paternal COPD history, age at menarche, menopause status, age at natural menopause, parity, oral contraception use, hormone replacement therapy use or gynaecological surgery history.

For peer review only

For peer review only - http://bmjopen.bmj.com/site/about/guidelines.xhtml

TABLES

Table 1.

Distribution of observed and imputed data in UK Biobank women at baseline (N=273 441)

**Characteristic**	**% data imputed**	**Mean (SE) or % in observed data**	**Mean (SE) or % in imputed data**
Age (years)	0	56.35 (0.02)	56.35 (0.02)
<55		39.5	39.5
55-59		18.6	18.6
60-64		24.2	24.2
>64		17.8	17.8
Height (cm)	0.4	162.4 (0.01)	162.4 (0.01)
Body mass index (kg/m^2)	0		
<18.5		0.8	0.8
18.5-24.9		38.7	38.7
25.0-29.9		36.5	36.5
>29.9		24.1	24.1
Non-white ethnicity	0.5	5.4	5.4
Education	1.2		
College, university or other professional degree		36.9	37.3
A levels, AS levels or equivalent		11.8	11.9
O levels, GCSEs or equivalent		23.3	23.5
CSE or equivalent		5.4	5.4
NVQ, HND, HNC or equivalent		4.5	4.6
Other		17.0	17.3

For peer review only - http://bmjopen.bmj.com/site/about/guidelines.xhtml

Annual household income	0.4		
<£18,000		20.4	20.6
£18,000-£30,999		21.7	21.8
£31,000-£51,999		20.9	21.0
£52,000-£100,000		15.3	15.4
>£100,000		4.0	4.0
Do not know/prefer not to answer		17.3	17.4
Townsend deprivation index	0.1	-1.33 (0.01)	-1.33 (0.01)
Smoking history (pack-years)	14.3		
None		59.3	69.1
Up to 10		7.9	9.2
11-20		7.4	8.7
21-30		5.2	6.1
>30		5.9	6.9
Prevalent (baseline) COPD	0	0.8	0.8
Incident COPD hospitalisation/death	0	0.4	0.4
Spirometry[a]	28.4		
FEV$_1$ z-score		0.00001 (0.002)	-0.0008 (0.002)
FVC z-score		-0.000002 (0.002)	-0.001 (0.002)
FEV$_1$/FVC z-score		0.00003 (0.002)	0.002 (0.002)
Asthma	0	12.9	12.9
Maternal history of COPD	1.9		
No		90.0	91.7
Yes		6.0	6.1
Don’t know		2.2	2.2

For peer review only

Paternal history of COPD	3.0		
No		81.7	84.2
Yes		10.3	10.6
Don't know		5.0	5.2
Age at menarche, years	3.3		
<12		19.4	20.0
12-15		71.7	74.1
>15		5.7	5.9
Menopause status	16.1		
No		23.4	26.4
Yes		60.5	73.6
Age at natural menopause, years	20.8		
<47		11.4	20.7
47-49		8.9	12.8
50-52		19.7	23.7
>52		15.8	16.3
Did not undergo natural menopause		23.4	26.4
Parity	1.9		
Nulliparous		14.7	15.0
Parous		83.4	85.0
1		11.9	12.2
2		33.7	34.4
3		24.8	25.3
>3		12.9	13.2
PCOS/ovarian cysts	0	1.7	1.7

For peer review only

Endometriosis	0	1.5	1.5
Oral contraception use, years	0.5		
Never		18.9	19.0
Ever		80.6	81.0
1		11.0	11.1
2-4		18.6	18.7
5-9		17.7	17.7
10-15		17.4	17.4
>15		16.0	16.0
Hormone replacement therapy, years	5.7		
Never		61.4	63.7
Ever		32.9	36.3
1-2		10.7	11.8
3-5		8.4	9.1
6-10		5.7	6.2
>10		8.2	9.2
Gynaecological surgery	1.8		
No		80.4	81.7
Hysterectomy		9.9	10.1
Bilateral oophorectomy		0.4	0.4
Both		7.6	7.8

SE, standard error; A levels, Advanced levels; AS levels, Advanced Subsidiary levels; O levels, Ordinary levels; GCSE, General Certificate of Secondary Educations; CSE, certificate of secondary education; NVQ, national vocational qualifications; HND, higher national diploma; HNC, higher national certificate; COPD, chronic obstructive pulmonary disease; PCOS, polycystic ovary syndrome

[a]Spirometry measures were standardised by age, sex and height

For peer review only - http://bmjopen.bmj.com/site/about/guidelines.xhtml

For peer review only

Table 2.

Associations of female reproductive health with incident COPD-related hospitalisation/death during follow-up in women without COPD at baseline (N=271 271)

Reproductive health indicator	% of participants	Person-years of follow-up	Number of COPD events	Age-adjusted		Multiple-adjusted[a]	
				Hazard ratio	95% CI	Hazard ratio	95% CI
Age at menarche							
<12 years	20.0	326 027	275	1.31	1.14, 1.51	1.15	1.00, 1.32
12-15 years	74.1	1 210 146	768	1.0	NA	1.0	NA
>15 years	5.9	95 771	95	1.61	1.29, 1.99	1.37	1.11, 1.71
Menopause status							
No	26.6	437 890	97	1.0	NA	1.0	NA
Yes	73.4	1 194 055	1041	1.47	1.12, 1.94	1.07	0.82, 1.41
Age at natural menopause[b]							
<47 years	20.3	202 811	270	2.21	1.83, 2.68	1.44	1.19, 1.75
47-49 years	15.8	158 718	140	1.55	1.24, 1.94	1.25	1.00, 1.57

For peer review only - http://bmjopen.bmj.com/site/about/guidelines.xhtml

50-52 years	35.4	354 774	219	1.0	NA	1.0	NA
>52 years	28.4	283 721	161	0.86	0.69, 1.06	0.93	0.75, 1.15
Parity, binary							
Nulliparous	15.1	244 938	116	0.77	0.63, 0.93	0.92	0.76, 1.12
Parous	84.9	1 387 009	1022	1.0	NA	1.0	NA
Parity, ordered categories							
0	15.1	244 938	116	1.0	NA	1.0	NA
1	12.2	198 124	139	1.41	1.10, 1.81	1.06	0.82, 1.35
2	34.4	562 339	308	0.96	0.78, 1.19	0.92	0.74, 1.14
3	25.3	413 347	293	1.24	1.00, 1.54	1.06	0.85, 1.33
>3	13.1	213 195	282	2.21	1.78, 2.75	1.45	1.16, 1.82
PCOS/ovarian cysts							
No	98.3	1 604 641	1108	1.0	NA	1.0	NA
Yes	1.7	27 304	30	1.61	1.12, 2.32	1.61	1.12, 2.32
Endometriosis							
No	98.5	1 607 817	1127	1.0	NA	1.0	NA

For peer review only

For peer review only - http://bmjopen.bmj.com/site/about/guidelines.xhtml

Yes	1.5	24 131	11	0.79	0.44, 1.43	0.85	0.47, 1.53
Oral contraception							
Never used	18.9	308 641	295	1.0	NA	1.0	NA
Ever used	81.1	1 323 303	843	0.95	0.83, 1.09	0.85	0.74, 0.97
Years of oral contraception use							
0	18.9	308 641	295	1.0	NA	1.0	NA
1	11.1	180 195	137	1.05	0.85, 1.29	0.84	0.69, 1.04
2-4	18.7	306 053	246	1.05	0.88, 1.24	0.95	0.80, 1.13
5-9	17.8	290 554	181	0.92	0.76, 1.11	0.86	0.71, 1.05
10-15	17.5	285 766	153	0.84	0.69, 1.03	0.74	0.61, 0.91
>15	16.1	260 734	126	0.83	0.67, 1.04	0.75	0.60, 0.93
HRT							
Never used	63.9	1 042 083	510	1.0	NA	1.0	NA
Ever used	36.1	589 861	628	1.49	1.31, 1.69	1.15	1.01, 1.30
Years of HRT							
0	63.9	1 042 083	510	1.0	NA	1.0	NA

For peer review only - http://bmjopen.bmj.com/site/about/guidelines.xhtml

1-2	11.7	190 902	240	1.83	1.54, 2.16	1.33	1.12, 1.57
3-5	9.1	147 962	162	1.63	1.34, 1.97	1.17	0.97, 1.42
6-10	6.2	100 921	74	1.00	0.77, 1.31	0.87	0.66, 1.14
>10	9.2	150 077	152	1.27	1.04, 1.55	1.06	0.87, 1.29
Gynaecological surgery							
No	81.8	1 335 512	765	1.0	NA	1.0	NA
Hysterectomy	10.1	164 869	213	1.77	1.51, 2.07	1.49	1.28, 1.74
Bilateral oophorectomy	0.4	5957	6	1.56	0.70, 3.48	1.36	0.61, 3.04
Both	7.8	125 607	154	1.68	1.41, 2.00	1.42	1.19, 1.69

COPD, chronic obstructive pulmonary disease; CI, confidence interval; PCOS, polycystic ovary syndrome; HRT, hormone replacement

[a]Multiple-adjusted for age, height, BMI (log-transformed), ethnicity, education, household income, Townsend deprivation index, smoking history, maternal COPD and paternal COPD

[b]Age at menopause was analysed only among women who experienced natural menopause before baseline (n=159 571)

For peer review only

Table 3.

Associations of female reproductive health indicators with baseline spirometry measures (N=273 441)

Reproductive health indicator	Mean change in FEV_1 z-score (95% CI)		Mean change in FVC z-score (95% CI)		Mean change in FEV_1/FVC z-score (95% CI)	
	Unadjusted	Multiple-adjusted[a]	Unadjusted	Multiple-adjusted[a]	Unadjusted	Multiple-adjusted[a]
Age at menarche						
<12 years	-0.04 (-0.05, -0.03)	-0.01 (-0.02, 0.00)	-0.05 (-0.06, -0.04)	-0.01 (-0.02, -0.01)	0.02 (0.01, 0.03)	-0.02 (-0.03, -0.01)
12-15 years	0	0	0	0	0	0
>15 years	-0.08 (-0.10, -0.06)	-0.03 (-0.05, -0.01)	-0.06 (-0.08, -0.04)	-0.03 (-0.05, -0.02)	-0.05 (-0.07, -0.02)	0.00 (-0.02, 0.02)
Menopause status						
No	0	0	0	0	0	0
Yes	-0.03 (-0.04, -0.02)	-0.04 (-0.05, -0.02)	-0.03 (-0.04, -0.02)	-0.04 (-0.06, -0.03)	0.00 (-0.01, 0.01)	0.01 (-0.01, 0.02)
Age at natural menopause[b]						
<47 years	-0.11 (-0.12, -0.09)	-0.04 (-0.05, -0.02)	-0.09 (-0.11, -0.08)	-0.04 (-0.05, -0.02)	-0.05 (-0.07, -0.04)	-0.01 (-0.02, 0.01)
47-49 years	-0.05 (-0.07, -0.04)	-0.02 (-0.04, 0.00)	-0.04 (-0.06, -0.03)	-0.02 (-0.03, 0.00)	-0.03 (-0.05, -0.02)	-0.01 (-0.03, 0.01)
50-52 years	0	0	0	0	0	0

For peer review only - http://bmjopen.bmj.com/site/about/guidelines.xhtml

For peer review only

>52 years	0.01 (0.00, 0.03)	0.00 (-0.01, 0.02)	0.00 (-0.02, 0.01)	0.00 (-0.02, 0.01)	0.03 (0.02, 0.05)	0.01 (0.00, 0.02)
Parity, binary						
Nulliparous	-0.02 (-0.03, -0.01)	-0.04 (-0.06, -0.03)	-0.04 (-0.05, -0.03)	-0.07 (-0.08, -0.05)	0.04 (0.03, 0.05)	0.04 (0.03, 0.05)
Parous	0	0	0	0	0	0
Parity, ordered categories						
0	0	0	0	0	0	0
1	-0.02 (-0.04, -0.01)	0.01 (0.00, 0.03)	-0.01 (-0.02, 0.01)	0.02 (0.01, 0.04)	-0.04 (-0.06, -0.03)	-0.02 (-0.04, 0.00)
2	0.04 (0.03, 0.06)	0.04 (0.03, 0.06)	0.06 (0.05, 0.08)	0.07 (0.06, 0.08)	-0.03 (-0.05, -0.02)	-0.04 (-0.06, -0.03)
3	0.03 (0.02, 0.05)	0.05 (0.04, 0.07)	0.05 (0.04, 0.07)	0.08 (0.06, 0.09)	-0.04 (-0.06, -0.03)	-0.04 (-0.06, -0.03)
>3	-0.03 (-0.05, -0.01)	0.05 (0.04, 0.07)	-0.01 (-0.02, 0.01)	0.08 (0.07, 0.10)	-0.07 (-0.08, -0.05)	-0.06 (-0.07, -0.04)
PCOS/ovarian cysts						
No	0	0	0	0	0	0
Yes	-0.02 (-0.05, 0.02)	-0.01 (-0.04, 0.03)	-0.05 (-0.08, -0.01)	-0.03 (-0.07, 0.00)	0.06 (0.03, 0.09)	0.05 (0.02, 0.09)
Endometriosis						
No	0	0	0	0	0	0
Yes	0.01 (-0.03, 0.04)	0.01 (-0.02, 0.05)	-0.02 (-0.06, 0.01)	-0.02 (-0.05, 0.01)	0.06 (0.02, 0.10)	0.07 (0.03, 0.10)

For peer review only - http://bmjopen.bmj.com/site/about/guidelines.xhtml

Oral contraception						
Never used	0	0	0	0	0	0
Ever used	0.07 (0.05, 0.08)	0.03 (0.01, 0.04)	0.08 (0.07, 0.09)	0.02 (0.01, 0.03)	-0.01 (-0.03, 0.00)	0.01 (0.003, 0.03)
Years of oral contraception						
0	0	0	0	0	0	0
1	0.03 (0.01, 0.04)	0.01 (-0.01, 0.03)	0.04 (0.02, 0.06)	0.01 (-0.01, 0.03)	-0.03 (-0.04, -0.01)	0.01 (-0.004, 0.03)
2-4	0.05 (0.04, 0.07)	0.02 (0.01, 0.04)	0.06 (0.05, 0.08)	0.02 (0.01, 0.03)	0.00 (-0.02, 0.01)	0.02 (0.003, 0.03)
5-9	0.08 (0.07, 0.10)	0.03 (0.02, 0.05)	0.09 (0.08, 0.10)	0.03 (0.01, 0.04)	0.00 (-0.02, 0.01)	0.02 (0.003, 0.03)
10-15	0.08 (0.07, 0.10)	0.04 (0.02, 0.05)	0.10 (0.08, 0.11)	0.04 (0.02, 0.05)	-0.02 (-0.03, 0.00)	0.01 (-0.002, 0.03)
>15	0.07 (0.05, 0.08)	0.02 (0.01, 0.04)	0.08 (0.07, 0.10)	0.02 (0.00, 0.03)	-0.03 (-0.04, -0.01)	0.01 (-0.002, 0.03)
HRT						
Never used	0	0	0	0	0	0
Ever used	-0.02 (-0.02, -0.01)	0.00 (0.00, 0.01)	-0.01 (-0.01, 0.00)	0.00 (-0.01, 0.01)	-0.02 (-0.03, -0.01)	0.02 (0.01, 0.03)
Years of HRT						
0	0	0	0	0	0	0
1-2	-0.03 (-0.04, -0.01)	0.00 (-0.01, 0.02)	-0.01 (-0.03, 0.00)	0.00 (-0.02, 0.01)	-0.03 (-0.05, -0.02)	0.02 (0.01, 0.04)

For peer review only - http://bmjopen.bmj.com/site/about/guidelines.xhtml

For peer review only

3-5	-0.03 (-0.04, -0.01)	0.00 (-0.01, 0.02)	-0.02 (-0.03, 0.00)	0.00 (-0.02, 0.02)	-0.02 (-0.04, 0.00)	0.02 (0.00, 0.03)
6-10	0.00 (-0.02, 0.02)	0.01 (-0.01, 0.02)	0.00 (-0.02, 0.02)	0.00 (-0.02, 0.02)	0.00 (-0.02, 0.01)	0.02 (0.00, 0.04)
>10	0.00 (-0.02, 0.01)	0.00 (-0.01, 0.02)	0.01 (0.00, 0.03)	0.00 (-0.01, 0.02)	-0.03 (-0.05, -0.01)	0.01 (-0.01, 0.03)
Gynaecological surgery						
No	0	0	0	0	0	0
Hysterectomy	-0.03 (-0.05, -0.02)	0.01 (0.00, 0.03)	-0.05 (-0.06, -0.04)	0.00 (-0.02, 0.01)	0.03 (0.01, 0.04)	0.04 (0.02, 0.06)
Bilateral oophorectomy	-0.05 (-0.12, 0.02)	-0.02 (-0.08, 0.05)	-0.07 (-0.14, 0.00)	-0.04 (-0.11, 0.03)	0.04 (-0.03, 0.12)	0.05 (-0.02, 0.12)
Both	-0.04 (-0.05, -0.02)	0.00 (-0.01, 0.02)	-0.07 (-0.08, -0.05)	-0.02 (-0.04, -0.01)	0.05 (0.04, 0.07)	0.05 (0.04, 0.07)

FEV_1, forced expiratory volume in one-second; FVC, forced vital capacity; CI, confidence interval; PCOS, polycystic ovary syndrome; HRT, hormone replacement therapy

[a]Adjusted for age, BMI (log-transformed), ethnicity, education, household income, Townsend deprivation index, smoking history, asthma, maternal COPD and paternal COPD

[b]Age at menopause was analysed only among women who experienced natural menopause before baseline (n=161 069)

For peer review only

Table 4.

Direction of association for female reproductive health indicators with risk of COPD-related hospitalisation/death and spirometry measures

Reproductive health indicator	COPD-related hospitalisation/death	FEV_1	FVC	FEV_1/FVC
Age at menarche				
<12 years	=	=	↓	↓
12-15 years	NA	NA	NA	NA
>15 years	↑	↓	↓	=
Menopause status				
No	NA	NA	NA	NA
Yes	=	↓	↓	=
Age at natural menopause				
<47 years	↑	↓	↓	=
47-49 years	=	=	=	=
50-52 years	NA	NA	NA	NA
>52 years	=	=	=	=
Parity, binary				
Nulliparous	=	↓	↓	↑
Parous	NA	NA	NA	NA
Parity, ordered categories				
0	NA	NA	NA	NA
1	=	=	↑	↓
2	=	↑	↑	↓
3	=	↑	↑	↓

For peer review only - http://bmjopen.bmj.com/site/about/guidelines.xhtml

>3	↑	↑	↑	↓
PCOS/ovarian cysts				
No	NA	NA	NA	NA
Yes	↑	=	=	↑
Endometriosis				
No	NA	NA	NA	NA
Yes	=	=	=	↑
Oral contraception				
Never used	NA	NA	NA	NA
Ever used	↓	↑	↑	=
Years of oral contraception				
0	NA	NA	NA	NA
1	=	=	=	=
2-4	=	↑	↑	↑
5-9	=	↑	↑	↑
10-15	↓	↑	↑	=
>15	↓	↑	=	=
Hormone replacement therapy				
Never used	NA	NA	NA	NA
Ever used	↑	=	=	↑
Years of hormone replacement				
0	NA	NA	NA	NA
1-2	↑	=	=	↑
3-5	=	=	=	=
6-10	=	=	=	=

For peer review only - http://bmjopen.bmj.com/site/about/guidelines.xhtml

For peer review only

>10	=	=	=	=
Gynaecological surgery				
No	NA	NA	NA	NA
Hysterectomy	↑	=	=	↑
Bilateral oophorectomy	=	=	=	=
Both	↑	=	↓	↑

COPD, chronic obstructive pulmonary disease; FEV_1, forced expiratory volume in one-second; FVC, forced vital capacity; PCOS, polycystic ovary syndrome; NA, not applicable where the category is the reference group

For peer review only

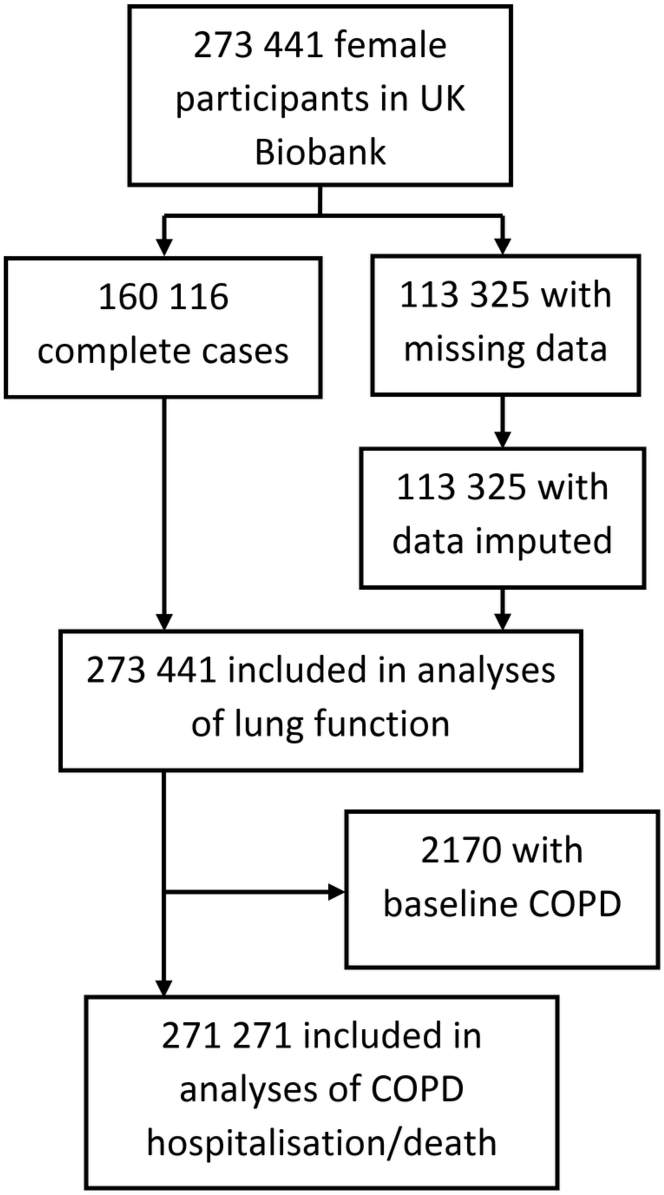

Figure 1. Flow Chart of Study Population.
COPD, chronic obstructive pulmonary disease. Missing data is in one or more of the following variables: height, ethnicity, educational attainment, income, Townsend deprivation index, smoking history, spirometry measurements, maternal COPD history, paternal COPD history, age at menarche, menopause status, age at natural menopause, parity, oral contraception use, hormone replacement therapy use or gynaecological surgery history.

80x117mm (300 x 300 DPI)

Online Supplementary File

Table S1. Variables used in multiple imputation model

**Variable**	**Type of variable**	**Regression model to predict missing in this variable**	**How variable was entered when used to predict missing in other variables**
Incident COPD hospitalisation	Dichotomous	Logistic regression	1 indicator variable
Incident COPD death	Dichotomous	Logistic regression	1 indicator variable
FEV_1 z-score	Continuous	Linear regression	Continuous
FVC z-score	Continuous	Linear regression	Continuous
FEV_1/FVC z-score	Continuous	Linear regression	Continuous
Age at menarche, years	Categorical (3)	Ordered logistic regression	2 indicator variables
Menopause status	Categorical (3)	Polytomous (unordered) regression	2 indicator variables
Age at menopause, years	Categorical (4)	Ordered logistic regression	3 indicator variables
Parity	Categorical (5)	Ordered logistic regression	4 indicator variables
History of ovarian cysts or polycystic ovary syndrome	Dichotomous	Logistic regression	Dichotomous
History of endometriosis	Dichotomous	Logistic regression	Dichotomous
Oral contraception ever use	Dichotomous	Passively imputed using years of OC use	N/A
Years of oral contraception	Categorical (6)	Ordered logistic regression	5 indicator variables
Gynaecological surgery	Categorical (4)	Polytomous (unordered) regression	3 indicator variables
HRT ever use	Dichotomous	Passively imputed using years of HRT use	N/A

For peer review only - http://bmjopen.bmj.com/site/about/guidelines.xhtml

For peer review only

Years of HRT use	Categorical (6)	Polytomous (unordered) regression	5 indicator variables
Age, years	Continuous	Linear regression	Continuous
Height, cm	Continuous	Linear regression	Continuous
Log body mass index	Continuous	Linear regression	Continuous
Ethnicity	Dichotomous	Logistic regression	Dichotomous
Qualifications	Categorical (6)	Polytomous (unordered) regression	6 indicator variables
Income	Categorical (6)	Polytomous (unordered) regression	5 indicator variables
Townsend deprivation index	Continuous	Linear regression	Continuous
Smoking pack-years	Categorical (5)	Ordered logistic regression	4 indicator variables
Smoking duration, years	Categorical (5)	Ordered logistic regression	4 indicator variables
Smoking intensity, cigarettes/day	Continuous	Linear regression	Continuous
Smoking status	Dichotomous	Logistic regression	Dichotomous
Maternal history of COPD	Dichotomous	Logistic regression	Dichotomous
Paternal history of COPD	Dichotomous	Logistic regression	Dichotomous
History of asthma	Dichotomous	Logistic regression	Dichotomous
History of pneumonia	Dichotomous	Logistic regression	Dichotomous
History of tuberculosis	Dichotomous	Logistic regression	Dichotomous
History of cardiovascular disease	Dichotomous	Logistic regression	Dichotomous
History of diabetes	Dichotomous	Logistic regression	Dichotomous

For peer review only - http://bmjopen.bmj.com/site/about/guidelines.xhtml

For peer review only

COPD, chronic obstructive pulmonary disease; FEV_1, forced expiratory volume in one-second; FVC, forced vital capacity; HRT, hormone replacement therapy

For peer review only

For peer review only - http://bmjopen.bmj.com/site/about/guidelines.xhtml

Table S2. Characteristics in UK Biobank women with and without spirometry data

Characteristic	Mean (SE) or % in women with spirometry	Mean (SE) or % in women without spirometry	*P* value
N	195 824	77 617	
Age (years)	56.18 (0.02)	56.78 (0.03)	<0.001
<55	20.9	20.3	
55-59	23.0	20.2	
60-64	30.5	29.7	
65 or higher	25.6	29.7	
Missing	0	0	
Height (cm)	162.6 (0.01)	162.0 (0.02)	<0.001
Missing	0.1	2.5	
Body mass index (kg/m^2)			<0.001
<18.5	0.6	1.2	
18.5-24.9	38.3	36.1	
25.0-29.9	37.5	35.7	
>29.9	23.4	25.3	
Missing	0.2	1.6	
Ethnicity			<0.001
White	95.0	92.2	
Other	4.7	7.1	
Missing	0.3	0.8	
Education			<0.001
College, university or other professional degree	37.9	34.4	
A levels, AS levels or equivalent	12.2	10.6	
O levels, GCSEs or equivalent	23.5	22.7	
CSE or equivalent	5.3	5.6	
NVQ, HND, HNC or equivalent	4.4	4.8	
Other	15.7	20.1	
Missing	1.0	1.8	
Annual household income (£)			<0.001
<18 000	19.2	23.4	

For peer review only - http://bmjopen.bmj.com/site/about/guidelines.xhtml

>18 000 and <31 000	21.7	21.6	
>31 000 and <52 000	21.7	19.0	
>52 000 and <100 000	16.3	12.8	
>100 000	4.3	3.0	
Do not know/prefer not to answer	16.4	19.5	
Missing	0.3	0.7	
Townsend deprivation index	-1.41 (0.01)	-1.14 (0.01)	<0.001
Missing	0.1	0.1	
Smoking history (pack-years)			<0.001
None	58.4	61.4	
<11	8.3	6.8	
11-20	7.7	6.7	
21-30	5.4	4.9	
>30	5.7	6.2	
Missing	14.5	14.0	
Incident COPD hospitalisation/death	0.3	0.6	<0.001
Missing	0.6	1.2	
Prevalent COPD at baseline	0.6	1.2	<0.001
Missing	<0.1	0	
Asthma			<0.001
No	86.9	87.6	
Yes	13.1	12.4	
Missing	<0.1	0	
Maternal history of COPD			<0.001
No	90.3	89.4	
Yes	6.0	5.8	
Don't know	2.0	2.4	
Missing	1.7	2.4	
Paternal history of COPD			<0.001
No	82.2	80.6	
Yes	10.2	10.3	
Don't know	4.8	5.5	
Missing	2.8	3.6	
Age at menarche			<0.001

For peer review only

For peer review only - http://bmjopen.bmj.com/site/about/guidelines.xhtml

<12	19.3	19.5	
12-15	72.2	70.3	
>15	5.5	6.2	
Missing	3.0	4.0	
Menopause status			<0.001
No	24.0	22.1	
Yes	60.3	61.0	
Missing	15.7	16.9	
Age at natural menopause			<0.001
<47	11.5	12.6	
47-49	9.4	9.4	
50-52	21.4	20.9	
>52	17.1	16.9	
Did not undergo natural menopause	35.6	34.6	
Missing	4.9	5.7	
Parity, binary			<0.001
Nulliparous	14.5	15.4	
Parous	83.8	82.3	
Parity, ordinal (5)			<0.001
0	14.5	15.4	
1	11.9	11.9	
2	34.1	32.7	
3	25.1	24.1	
>3	12.7	13.5	
Missing	1.7	2.4	
PCOS/ovarian cysts	1.7	1.7	0.60
Missing	0	0	
Endometriosis	1.5	1.5	0.81
Missing	0	0	
Oral contraception ever used	81.9	77.4	<0.001
Years of oral contraception use			<0.001
0	17.7	21.6	
1	10.8	11.6	

For peer review only

For peer review only - http://bmjopen.bmj.com/site/about/guidelines.xhtml

2-4	18.6	18.6	
5-9	18.1	16.5	
10-15	17.9	16.2	
>15	16.5	14.6	
Missing	0.3	1.0	
HRT ever used	37.8	38.7	<0.001
Years of HRT			<0.001
0	61.9	60.3	
1-2	10.5	11.1	
3-5	8.3	8.6	
6-10	5.7	5.5	
>10	8.2	8.2	
Missing	5.5	6.3	
Gynaecological surgery			<0.001
No	81.2	78.5	
Hysterectomy	9.6	10.5	
Bilateral oophorectomy	0.4	0.4	
Both	7.3	8.2	
Missing	1.5	2.4	

SE, standard error; A levels, Advanced levels; AS levels, Advanced Subsidiary levels; O levels, Ordinary levels; GCSE, General Certificate of Secondary Educations; CSE, certificate of secondary education; NVQ, national vocational qualifications; HND, higher national diploma; HNC, higher national certificate; COPD, chronic obstructive pulmonary disease; PCOS, polycystic ovary syndrome; HRT, hormone replacement therapy

For peer review only

For peer review only - http://bmjopen.bmj.com/site/about/guidelines.xhtml

Table S3. Associations of female reproductive health with incident COPD-related hospitalisation/death during follow-up in **women with $FEV_1/FVC \geq 0.7$ and without a history of respiratory illness**[a] (N=217 512)

Reproductive health indicator	% of participants	Person-years of follow-up	Number of COPD events	Age-adjusted		Multiple-adjusted[b]	
				Hazard ratio	95% CI	Hazard ratio	95% CI
Age at menarche							
<12 years	19.8	259 259	97	1.39	1.10, 1.76	1.27	1.00, 1.62
12-15 years	74.4	975 565	259	1.0	NA	1.0	NA
>15 years	5.8	75 410	36	1.84	1.29, 2.62	1.53	1.08, 2.19
Menopause status							
No	27.2	359 354	34	1.0	NA	1.0	NA
Yes	72.8	950 881	358	1.09	1.07, 1.12	0.88	0.56, 1.39
Age at natural menopause[c]							
<47 years	19.8	157 942	90	2.25	1.60, 3.15	1.59	1.14, 2.23
47-49 years	15.9	127 134	50	1.67	1.12, 2.47	1.40	0.94, 2.08
50-52 years	35.7	285 678	74	1.0	NA	1.0	NA
>52 years	28.6	228 061	57	0.89	0.61, 1.30	0.97	0.67, 1.42
Parity, binary							
Nulliparous	15.3	199 136	43	0.84	0.60, 1.15	0.93	0.67, 1.29
Parous	84.7	1 111 099	349	1.0	NA	1.0	NA
Parity, ordered categories							
0	15.3	199 136	43	1.0	NA	1.0	NA
1	12.2	159 386	49	1.37	0.91, 2.07	1.10	0.72, 1.66

For peer review only - http://bmjopen.bmj.com/site/about/guidelines.xhtml

For peer review only

2	34.7	455 068	115	0.95	0.67, 1.36	0.97	0.68, 1.39
3	25.2	330 200	98	1.12	0.77, 1.61	1.03	0.71, 1.49
>3	12.7	166 444	87	1.86	1.28, 2.70	1.34	0.92, 1.95
PCOS/ovarian cysts							
No	98.3	1 288 468	378	1.0	NA	1.0	NA
Yes	1.7	21 767	14	2.20	1.29, 3.76	2.23	1.31, 3.81
Endometriosis							
No	98.5	1 291 165	387	1.0	NA	1.0	NA
Yes	1.5	459 189	5	1.07	0.44, 2.58	1.09	0.45, 2.65
Oral contraception							
Never used	18.9	247 243	117	1.0	NA	1.0	NA
Ever used	81.1	1 062 992	275	0.81	0.65, 1.01	0.72	0.57, 0.91
Years of oral contraception							
0	18.9	247 243	117	1.0	NA	1.0	NA
1	10.9	143 083	41	0.83	0.58, 1.18	0.66	0.46, 0.94
2-4	18.6	244 583	86	0.94	0.71, 1.25	0.88	0.66, 1.17
5-9	17.8	234 184	57	0.75	0.54, 1.03	0.70	0.50, 0.97
10-15	17.6	230 642	51	0.74	0.53, 1.04	0.65	0.46, 0.92
>15	16.1	210 498	40	0.71	0.49, 1.03	0.62	0.43, 0.90
HRT							
Never used	65.0	851 046	181	1.0	NA	1.0	NA
Ever used	35.0	459 189	211	1.41	1.14, 1.74	1.11	0.90, 1.38

For peer review only - http://bmjopen.bmj.com/site/about/guidelines.xhtml

Years of HRT							
0	65.0	851 046	181	1.0	NA	1.0	NA
1-2	11.2	146 764	83	1.81	1.37, 2.38	1.33	1.01, 1.75
3-5	8.8	116 043	51	1.43	1.03, 1.98	1.07	0.77, 1.49
6-10	6.1	80 081	25	0.97	0.63, 1.50	0.85	0.55, 1.32
>10	8.8	116 300	52	1.21	0.87, 1.69	1.05	0.75, 1.46
Gynaecological surgery							
No	82.3	1 078 722	263	1.0	NA	1.0	NA
Hysterectomy	9.7	127 997	73	1.75	1.34, 2.29	1.54	1.18, 2.00
Bilateral oophorectomy	0.4	4749	4	2.86	1.06, 7.68	2.49	0.92, 6.70
Both	7.6	98 766	52	1.62	1.19, 2.19	1.39	1.03, 1.89

COPD, chronic obstructive pulmonary disease; FEV_1, forced expiratory volume in one-second; FVC, forced vital capacity; CI, confidence interval; PCOS, polycystic ovary syndrome; HRT, hormone replacement therapy; NA, not applicable

[a]Respiratory illnesses include COPD, asthma, tuberculosis or pneumonia reported at baseline

[b]Multiple-adjusted for age, height, BMI (log-transformed), ethnicity, education, household income, Townsend deprivation index, smoking history in pack-years, maternal COPD and paternal COPD

[c]Age at menopause was analysed only among women who experienced natural menopause before baseline (n=127 270)

For peer review only - http://bmjopen.bmj.com/site/about/guidelines.xhtml

Table S4. Associations of female reproductive health with incident COPD-related hospitalisation/death during follow-up, **further adjusting for smoking status or smoking duration and intensity** (N=271 271)

Reproductive health indicator	% of participants	Person-years of follow-up	Number of COPD events	Multiple-adjusted[a]		Smoking duration and intensity-adjusted[b]		Smoking status-adjusted[c]	
				Hazard ratio	95% CI	Hazard ratio	95% CI	Hazard ratio	95% CI
Age at menarche									
<12 years	20.0	326 027	275	1.15	1.00, 1.32	1.18	1.03, 1.36	1.17	1.01, 1.34
12-15 years	74.1	1 210 146	768	1.0	NA	1.0	NA	1.0	NA
>15 years	5.9	95 771	95	1.37	1.11, 1.71	1.33	1.07, 1.65	1.32	1.06, 1.64
Menopause status									
No	26.6	437 890	97	1.0	NA	1.0	NA	1.0	NA
Yes	73.4	1 194 055	1041	1.07	0.82, 1.41	1.06	0.81, 1.39	1.03	0.78, 1.36
Age at natural menopause[d]				1.44	1.19, 1.75	1.47	1.21, 1.79	1.43	1.18, 1.74
<47 years	20.3	202 811	270	1.25	1.00, 1.57	1.25	1.00, 1.57	1.24	0.99, 1.56
47-49 years	15.8	158 718	140	1.0	NA	1.0	NA	1.0	NA
50-52 years	35.4	354 774	219	0.93	0.75, 1.15	0.91	0.74, 1.13	0.94	0.76, 1.16
>52 years	28.4	283 721	161						
Parity, binary									
Nulliparous	15.1	244 938	116	0.92	0.76, 1.12	0.90	0.74, 1.09	0.92	0.75, 1.12
Parous	84.9	1 387 009	1022	1.0	NA	1.0	NA	1.0	NA

For peer review only - http://bmjopen.bmj.com/site/about/guidelines.xhtml

For peer review only

Parity, ordered categories										
0	15.1	244 938	116	1.0	NA	1.0	NA	1.0	NA
1	12.2	198 124	139	1.06	0.82, 1.35	1.10	0.86, 1.41	1.06	0.82, 1.36
2	34.4	562 339	308	0.92	0.74, 1.14	0.93	0.75, 1.16	0.93	0.75, 1.15
3	25.3	413 347	293	1.06	0.85, 1.33	1.10	0.88, 1.37	1.07	0.86, 1.34
>3	13.1	213 195	282	1.45	1.16, 1.82	1.50	1.20, 1.88	1.43	1.15, 1.79
PCOS/ovarian cysts									
No	98.3	1 604 641	1108	1.0	NA	1.0	NA	1.0	NA
Yes	1.7	27 304	30	1.61	1.12, 2.32	1.66	1.16, 2.39	1.65	1.14, 2.37
Endometriosis									
No	98.5	1 607 817	1127	1.0	NA	1.0	NA	1.0	NA
Yes	1.5	24 131	11	0.85	0.47, 1.53	0.84	0.46, 1.52	0.84	0.46, 1.53
Oral contraception									
Never used	18.9	308 641	295	1.0	NA	1.0	NA	1.0	NA
Ever used	81.1	1 323 303	843	0.85	0.74, 0.97	0.90	0.78, 1.03	0.87	0.75, 1.00
Years of oral contraception use	18.9	308 641	295	1.0	NA	1.0	NA	1.0	NA
0	11.1	180 195	137	0.84	0.69, 1.04	0.89	0.72, 1.09	0.86	0.70, 1.06
1	18.7	306 053	246	0.95	0.80, 1.13	1.00	0.84, 1.19	0.98	0.82, 1.16
2-4	17.8	290 554	181	0.86	0.71, 1.05	0.92	0.76, 1.11	0.87	0.72, 1.06
5-9	17.5	285 766	153	0.74	0.61, 0.91	0.80	0.66, 0.98	0.77	0.63, 0.94
10-15	16.1	260 734	126	0.75	0.60, 0.93	0.80	0.64, 1.00	0.77	0.62, 0.96

For peer review only - http://bmjopen.bmj.com/site/about/guidelines.xhtml

>15									
HRT									
Never used	63.9	1 042 083	510	1.0	NA	1.0	NA	1.0	NA
Ever used	36.1	589 861	628	1.15	1.01, 1.30	1.20	1.06, 1.36	1.16	1.03, 1.32
Years of HRT									
0	63.9	1 042 083	510	1.0	NA	1.0	NA	1.0	NA
1-2	11.7	190 902	240	1.33	1.12, 1.57	1.39	1.17, 1.65	1.36	1.14, 1.61
3-5	9.1	147 962	162	1.17	0.97, 1.42	1.23	1.01, 1.49	1.17	0.96, 1.42
6-10	6.2	100 921	74	0.87	0.66, 1.14	0.90	0.69, 1.18	0.88	0.67, 1.15
>10	9.2	150 077	152	1.06	0.87, 1.29	1.12	0.92, 1.36	1.07	0.88, 1.31
Gynaecological surgery									
No	81.8	1 335 512	765	1.0	NA	1.0	NA	1.0	NA
Hysterectomy	10.1	164 869	213	1.49	1.28, 1.74	1.49	1.27, 1.74	1.47	1.26, 1.72
Bilateral oophorectomy	0.4	5957	6	1.36	0.61, 3.04	1.34	0.60, 3.00	1.38	0.62, 3.08
Both	7.8	125 607	154	1.42	1.19, 1.69	1.41	1.18, 1.68	1.39	1.17, 1.66

COPD, chronic obstructive pulmonary disease; CI, confidence interval; PCOS, polycystic ovary syndrome; HRT, hormone replacement

[a]Multiple-adjusted for age, height, BMI (log-transformed), ethnicity, education, household income, Townsend deprivation index, smoking history in pack years, maternal COPD and paternal COPD

[b]Multiple-adjusted with smoking history as two covariates (duration in years and intensity in average number of cigarettes/day) rather than smoking pack-years

For peer review only - http://bmjopen.bmj.com/site/about/guidelines.xhtml

[c]Further adjusted from the multiple-adjusted analyses[a] for smoking status at baseline

[d]Age at menopause was analysed only among women who experienced natural menopause before baseline (n=159 571)

For peer review only

Table S5. Associations of female reproductive health with incident COPD-related hospitalisation/death during follow-up in **never smokers** (N=161 626)

**Reproductive health indicator**	**% of participants**	**Person-years of follow-up**	**Number of COPD events**	**Age-adjusted**		**Multiple-adjusted[a]**	
				**Hazard ratio**	**95% CI**	**Hazard ratio**	**95% CI**
Age at menarche							
<12 years	19.9	193 712	59	1.63	1.20, 2.22	1.39	1.02, 1.90
12-15 years	74.4	725 115	134	1.0	NA	1.0	NA
>15 years	5.7	55 624	18	1.82	1.11, 2.98	1.76	1.07, 2.89
Menopause status							
No	30.4	252 038	40	1.0	NA	1.0	NA
Yes	69.6	571 767	181	0.99	0.55, 1.77	0.98	0.55, 1.76
Age at natural menopause[b]							
<47 years	18.7	108 538	37	1.66	1.05, 2.64	1.53	0.96, 2.43
47-49 years	15.5	90 293	18	1.05	0.58, 1.91	1.03	0.57, 1.86
50-52 years	36.1	210 322	44	1.0	NA	1.0	NA
>52 years	29.7	172 378	33	0.85	0.54, 1.35	0.83	0.52, 1.32
Parity, binary							
Nulliparous	16.3	157 900	27	0.92	0.61, 1.38	0.94	0.62, 1.42
Parous	83.7	816 551	184	1.0	NA	1.0	NA
Parity, ordered categories							
0	16.3	157 900	27	1.0	NA	1.0	NA
1	11.2	109 141	24	1.21	0.70, 2.11	1.15	0.66, 2.00
2	35.2	343 543	69	0.97	0.62, 1.52	0.98	0.62, 1.54

For peer review only - http://bmjopen.bmj.com/site/about/guidelines.xhtml

For peer review only

3	24.8	242 317	60	1.17	0.74, 1.85	1.16	0.73, 1.84
>3	12.5	121 548	31	1.17	0.70, 1.96	1.03	0.61, 1.74
PCOS/ovarian cysts							
No	98.3	958 600	205	1.0	NA	1.0	NA
Yes	1.7	15 851	6	1.77	0.79, 3.99	1.73	0.77, 3.91
Endometriosis							
No	98.5	950 101	208	1.0	NA	1.0	NA
Yes	1.5	14 350	3	1.15	0.37, 3.61	1.10	0.35, 3.46
Oral contraception							
Never used	21.6	210 051	71	1.0	NA	1.0	NA
Ever used	78.4	764 401	140	0.74	0.55, 1.00	0.79	0.58, 1.06
Years of oral contraception use							
0	21.6	210 051	71	1.0	NA	1.0	NA
1	10.9	106 163	19	0.65	0.39, 1.10	0.67	0.40, 1.12
2-4	18.9	184 203	48	0.94	0.65, 1.36	0.97	0.67, 1.40
5-9	17.2	167 797	32	0.77	0.50, 1.17	0.82	0.53, 1.26
10-15	16.3	159 341	25	0.67	0.42, 1.07	0.73	0.45, 1.17
>15	15.1	146 894	16	0.50	0.29, 0.88	0.56	0.32, 0.97
HRT							
Never used	65.7	639 250	106	1.0	NA	1.0	NA
Ever used	34.3	335 202	105	1.25	0.94, 1.67	1.22	0.92, 1.62
Years of HRT							

For peer review only

For peer review only - http://bmjopen.bmj.com/site/about/guidelines.xhtml

0	65.7	639 250	106	1.0	NA	1.0	NA
1-2	9.6	93 783	39	1.79	1.23, 2.61	1.74	1.19, 2.53
3-5	7.6	74 345	17	0.99	0.59, 1.66	0.91	0.54, 1.53
6-10	5.3	51 998	15	1.16	0.67, 2.01	1.15	0.67, 1.99
>10	11.8	73 060	34	1.00	0.61, 1.64	1.02	0.61, 1.91
Gynaecological surgery							
No	82.2	801 428	136	1.0	NA	1.0	NA
Hysterectomy	9.9	96 682	40	1.92	1.34, 2.76	1.66	1.15, 2.39
Bilateral oophorectomy	0.4	3385	1	1.49	0.21, 10.63	1.47	0.21, 10.50
Both	7.5	72 956	34	2.12	1.45, 3.12	1.85	1.26, 2.72

COPD, chronic obstructive pulmonary disease; CI, confidence interval; PCOS, polycystic ovary syndrome; HRT, hormone replacement

[a]Multiple-adjusted for age, height, BMI (log-transformed), ethnicity, education, household income, Townsend deprivation index, maternal COPD and paternal COPD

[b]Age at menopause analysed only among women who experienced natural menopause before baseline (n=92 561)

For peer review only

For peer review only - http://bmjopen.bmj.com/site/about/guidelines.xhtml

Table S6. Associations of female reproductive health with incident COPD-related hospitalisation/death during follow-up, **further adjusting for comorbidities** (N=271 271)

Reproductive health indicator	% of participants	Person-years of follow-up	Number of COPD events	Multiple-adjusted[a]		Comorbidity-adjusted[c]	
				Hazard ratio	95% CI	Hazard ratio	95% CI
Age at menarche							
<12 years	20.0	326 027	275	1.15	1.00, 1.32	1.17	1.02, 1.35
12-15 years	74.1	1 210 146	768	1.0	NA	1.0	NA
>15 years	5.9	95 771	95	1.37	1.11, 1.71	1.32	1.06, 1.64
Menopause status							
No	26.6	437 890	97	1.0	NA	1.0	NA
Yes	73.4	1 194 055	1041	1.07	0.82, 1.41	1.06	0.81, 1.39
Age at natural menopause[d]							
<47 years	20.3	202 811	270	1.44	1.19, 1.75	1.46	1.21, 1.78
47-49 years	15.8	158 718	140	1.25	1.00, 1.57	1.26	1.01, 1.58
50-52 years	35.4	354 774	219	1.0	NA	1.0	NA
>52 years	28.4	283 721	161	0.93	0.75, 1.15	0.91	0.74, 1.13
Parity, binary							
Nulliparous	15.1	244 938	116	0.92	0.76, 1.12	0.90	0.74, 1.09
Parous	84.9	1 387 009	1022	1.0	NA	1.0	NA
Parity, ordered categories							
0	15.1	244 938	116	1.0	NA	1.0	NA
1	12.2	198 124	139	1.06	0.82, 1.35	1.10	0.86, 1.41

For peer review only - http://bmjopen.bmj.com/site/about/guidelines.xhtml

For peer review only

2	34.4	562 339	308	0.92	0.74, 1.14	0.93	0.75, 1.16
3	25.3	413 347	293	1.06	0.85, 1.33	1.10	0.88, 1.37
>3	13.1	213 195	282	1.45	1.16, 1.82	1.50	1.20, 1.87
PCOS/ovarian cysts							
No	98.3	1 604 641	1108	1.0	NA	1.0	NA
Yes	1.7	27 304	30	1.61	1.12, 2.32	1.64	1.14, 2.36
Endometriosis							
No	98.5	1 607 817	1127	1.0	NA	1.0	NA
Yes	1.5	24 131	11	0.85	0.47, 1.53	0.84	0.46, 1.53
Oral contraception							
Never used	18.9	308 641	295	1.0	NA	1.0	NA
Ever used	81.1	1 323 303	843	0.85	0.74, 0.97	0.91	0.79, 1.04
Years of oral contraception use							
0	18.9	308 641	295	1.0	NA	1.0	NA
1	11.1	180 195	137	0.84	0.69, 1.04	0.89	0.73, 1.10
2-4	18.7	306 053	246	0.95	0.80, 1.13	1.01	0.85, 1.20
5-9	17.8	290 554	181	0.86	0.71, 1.05	0.93	0.76, 1.12
10-15	17.5	285 766	153	0.74	0.61, 0.91	0.81	0.66, 0.99
>15	16.1	260 734	126	0.75	0.60, 0.93	0.81	0.65, 1.01
HRT							
Never used	63.9	1 042 083	510	1.0	NA	1.0	NA
Ever used	36.1	589 861	628	1.15	1.01, 1.30	1.20	1.05, 1.35

For peer review only - http://bmjopen.bmj.com/site/about/guidelines.xhtml

For peer review only

Years of HRT							
0	63.9	1 042 083	510	1.0	NA	1.0	NA
1-2	11.7	190 902	240	1.33	1.12, 1.57	1.38	1.16, 1.63
3-5	9.1	147 962	162	1.17	0.97, 1.42	1.22	1.01, 1.48
6-10	6.2	100 921	74	0.87	0.66, 1.14	0.90	0.69, 1.17
>10	9.2	150 077	152	1.06	0.87, 1.29	1.11	0.91, 1.35
Gynaecological surgery							
No	81.8	1 335 512	765	1.0	NA	1.0	NA
Hysterectomy	10.1	164 869	213	1.49	1.28, 1.74	1.47	1.26, 1.72
Bilateral oophorectomy	0.4	5957	6	1.36	0.61, 3.04	1.34	0.60, 2.99
Both	7.8	125 607	154	1.42	1.19, 1.69	1.39	1.17, 1.66

COPD, chronic obstructive pulmonary disease; CI, confidence interval; PCOS, polycystic ovary syndrome; HRT, hormone replacement

[a]Multiple-adjusted for age, height, BMI (log-transformed), ethnicity, education, household income, Townsend deprivation index, smoking history in pack years, maternal COPD and paternal COPD

[b]Further adjusted from the multiple-adjusted analyses[a] for a history of cardiovascular disease and/or diabetes at baseline

[c]Analysed in women who experienced natural menopause before baseline (n=159 571)

For peer review only

Table S7. Associations of female reproductive health indicators with baseline spirometry measures, **further adjusting for smoking status or smoking duration and intensity** (N=273 441)

Reproductive health indicator	Mean change in FEV_1 z-score (95% CI)		Mean change in FVC z-score (95% CI)		Mean change in FEV_1/FVC z-score (95% CI)	
	Smoking duration and intensity-adjusted[a]	Smoking status-adjusted[b]	Smoking duration and intensity-adjusted[a]	Smoking status-adjusted[b]	Smoking duration and intensity-adjusted [a]	Smoking status-adjusted [b]
Age at menarche						
<12 years	-0.01 (-0.02, 0.00)	-0.01 (-0.02, 0.00)	-0.01 (-0.02, 0.01)	-0.01 (-0.02, 0.01)	-0.02 (-0.03, -0.01)	-0.01 (-0.03, 0.00)
12-15 years	0	0	0	0	0	0
>15 years	-0.03 (-0.05, -0.01)	-0.03 (-0.05, -0.01)	-0.03 (-0.05, -0.02)	-0.03 (-0.05, -0.01)	0.00 (-0.02, 0.02)	0.00 (-0.02, 0.02)
Menopause status						
No	0	0	0	0	0	0
Yes	-0.04 (-0.05, -0.02)	-0.03 (-0.05, -0.02)	-0.04 (-0.05, -0.02)	-0.04 (-0.05, -0.02)	0.00 (-0.02, 0.01)	0.01 (0.00, 0.03)
Age at natural menopause[c]						
<47 years	-0.04 (-0.05, -0.02)	-0.03 (-0.05, -0.02)	-0.04 (-0.05, -0.02)	-0.04 (-0.05, -0.02)	-0.01 (-0.03, 0.00)	0.00 (-0.02, 0.01)
47-49 years	-0.02 (-0.04, 0.00)	-0.02 (-0.04, 0.00)	-0.02 (-0.04, 0.00)	-0.02 (-0.03, 0.00)	-0.01 (-0.03, 0.01)	-0.01 (-0.03, 0.01)
50-52 years	0	0	0	0	0	0
>52 years	0.00 (-0.01, 0.02)	0.00 (-0.01, 0.02)	0.00 (-0.02, 0.01)	0.00 (-0.02, 0.01)	0.01 (0.00, 0.03)	0.01 (-0.01, 0.02)
Parity, binary						
Nulliparous	-0.04 (-0.06, -0.03)	-0.04 (-0.06, -0.03)	-0.07 (-0.08, -0.05)	-0.07 (-0.08, -0.06)	0.04 (0.03, 0.05)	0.04 (0.03, 0.05)
Parous	0	0	0	0	0	0

For peer review only - http://bmjopen.bmj.com/site/about/guidelines.xhtml

Parity, ordered categories	0	0	0	0	0	0
0	0.01 (-0.01, 0.03)	0.01 (0.00, 0.03)	0.02 (0.01, 0.04)	0.03 (0.01, 0.04)	-0.02 (-0.04, -0.01)	-0.02 (-0.04, 0.00)
1	0.05 (0.03, 0.06)	0.04 (0.03, 0.06)	0.07 (0.06, 0.08)	0.07 (0.06, 0.08)	-0.04 (-0.06, -0.03)	-0.04 (-0.06, -0.03)
2	0.05 (0.04, 0.07)	0.05 (0.04, 0.07)	0.08 (0.07, 0.09)	0.08 (0.06, 0.09)	-0.04 (-0.06, -0.03)	-0.04 (-0.06, -0.03)
3	0.05 (0.04, 0.07)	0.06 (0.04, 0.07)	0.08 (0.07, 0.10)	0.09 (0.07, 0.10)	-0.06 (-0.08, -0.04)	-0.05 (-0.07, -0.03)
>3						
PCOS/ovarian cysts						
No	0	0	0	0	0	0
Yes	-0.01 (-0.04, 0.03)	-0.01 (-0.04, 0.02)	-0.03 (-0.07, 0.00)	-0.04 (-0.07, 0.00)	0.05 (0.02, 0.08)	0.06 (0.03, 0.09)
Endometriosis						
No	0	0	0	0	0	0
Yes	0.01 (-0.02, 0.05)	0.01 (-0.03, 0.04)	-0.02 (-0.05, 0.01)	-0.03 (-0.06, 0.01)	0.07 (0.03, 0.10)	0.07 (0.03, 0.10)
Oral contraception						
Never used	0	0	0	0	0	0
Ever used	0.02 (0.01, 0.03)	0.02 (0.01, 0.04)	0.02 (0.01, 0.03)	0.02 (0.01, 0.03)	0.01 (0.00, 0.02)	0.01 (0.00, 0.03)
Years of oral contraception						
0	0	0	0	0	0	0
1	0.01 (-0.01, 0.03)	0.01 (-0.01, 0.03)	0.01 (-0.01, 0.02)	0.01 (-0.01, 0.02)	0.01 (-0.01, 0.02)	0.01 (0.00, 0.03)

For peer review only

2-4	0.02 (0.01, 0.03)	0.02 (0.01, 0.04)	0.02 (0.00, 0.03)	0.02 (0.00, 0.03)	0.01 (0.00, 0.02)	0.01 (0.00, 0.03)
5-9	0.03 (0.01, 0.04)	0.03 (0.01, 0.04)	0.03 (0.01, 0.04)	0.02 (0.01, 0.04)	0.01 (-0.01, 0.02)	0.02 (0.00, 0.03)
10-15	0.03 (0.02, 0.05)	0.04 (0.02, 0.05)	0.03 (0.02, 0.05)	0.03 (0.02, 0.05)	0.00 (-0.01, 0.02)	0.01 (0.00, 0.03)
>15	0.02 (0.00, 0.03)	0.02 (0.01, 0.04)	0.02 (0.00, 0.03)	0.02 (0.00, 0.03)	0.00 (-0.01, 0.02)	0.01 (0.00, 0.03)
HRT						
Never used	0	0	0	0	0	0
Ever used	0.00 (-0.01, 0.01)	0.00 (-0.01, 0.01)	0.00 (-0.01, 0.01)	0.00 (-0.01, 0.01)	0.01 (0.00, 0.02)	0.02 (0.01, 0.03)
Years of HRT						
0	0	0	0	0	0	0
1-2	0.00 (-0.02, 0.01)	0.00 (-0.01, 0.02)	0.00 (-0.02, 0.01)	0.00 (-0.02, 0.01)	0.01 (0.00, 0.03)	0.02 (0.01, 0.04)
3-5	0.00 (-0.02, 0.02)	0.01 (-0.01, 0.02)	0.00 (-0.02, 0.01)	0.00 (-0.01, 0.02)	0.01 (-0.01, 0.02)	0.02 (0.00, 0.03)
6-10	0.00 (-0.02, 0.02)	0.01 (-0.01, 0.02)	0.00 (-0.02, 0.02)	0.00 (-0.02, 0.02)	0.01 (-0.01, 0.03)	0.02 (0.00, 0.04)
>10	-0.01 (-0.03, 0.01)	0.00 (-0.01, 0.02)	0.00 (-0.02, 0.02)	0.00 (-0.01, 0.02)	0.00 (-0.02, 0.02)	0.01 (0.01, 0.03)
Gynaecological surgery						
No	0	0	0	0	0	0
Hysterectomy	0.01 (0.00, 0.03)	0.02 (0.00, 0.03)	0.00 (-0.02, 0.01)	0.00 (-0.01, 0.01)	0.04 (0.02, 0.05)	0.04 (0.03, 0.06)
Bilateral oophorectomy	-0.02 (-0.09, 0.05)	-0.02 (-0.09, 0.05)	-0.04 (-0.11, 0.03)	-0.05 (-0.11, 0.03)	0.05 (-0.02, 0.12)	0.04 (-0.04, 0.12)
Both	0.00 (-0.01, 0.02)	0.00 (-0.01, 0.02)	-0.02 (-0.04, -0.01)	-0.02 (-0.04, 0.00)	0.05 (0.04, 0.07)	0.05 (0.04, 0.07)

FEV1, forced expiratory volume in one-second; FVC, forced vital capacity; CI, confidence interval; PCOS, polycystic ovary syndrome; HRT, hormone replacement therapy

For peer review only - http://bmjopen.bmj.com/site/about/guidelines.xhtml

[a]Adjusted for age, BMI (log-transformed), ethnicity, education, household income, Townsend deprivation index, smoking history duration, smoking history intensity, asthma, maternal COPD and paternal COPD

[b]Multiple-adjusted as above with an additional covariate of smoking status and adjusting for smoking history as one covariate (pack-years) rather than two covariates (smoking duration and smoking intensity)

[c]Analysed in women who experienced natural menopause before baseline (n=161 069)

For peer review only

For peer review only - http://bmjopen.bmj.com/site/about/guidelines.xhtml

Table S8. Associations of female reproductive health indicators with baseline spirometry measures, **further adjusting for comorbidities** (N=273 441)

**Reproductive health indicator**	**Mean change in FEV_1 z-score (95% CI)**		**Mean change in FVC z-score (95% CI)**		**Mean change in FEV_1/FVC z-score (95% CI)**	
	**Multiple-adjusted[a]**	**Further adjusted for comorbidities[b]**	**Multiple-adjusted[a]**	**Further adjusted for comorbidities[b]**	**Multiple-adjusted[a]**	**Further adjusted for comorbidities[b]**
Age at menarche						
<12 years	-0.01 (-0.02, 0.00)	-0.01 (-0.02, 0.00)	-0.01 (-0.02, -0.01)	-0.01 (-0.02, 0.01)	-0.02 (-0.03, -0.01)	-0.02 (-0.03, -0.01)
12-15 years	0	0	0	0	0	0
>15 years	-0.03 (-0.05, -0.01)	-0.03 (-0.05, -0.01)	-0.03 (-0.05, -0.02)	-0.03 (-0.05, -0.02)	0.00 (-0.02, 0.02)	0.00 (-0.02, 0.02)
Menopause status						
No	0	0	0	0	0	0
Yes	-0.04 (-0.05, -0.02)	-0.04 (-0.06, -0.03)	-0.04 (-0.06, -0.03)	-0.04 (-0.05, -0.02)	0.01 (-0.01, 0.02)	0.00 (-0.02, 0.01)
Age at natural menopause[c]						
<47 years	-0.04 (-0.05, -0.02)	-0.03 (-0.05, -0.02)	-0.04 (-0.05, -0.02)	-0.04 (-0.05, -0.02)	-0.01 (-0.02, 0.01)	-0.01 (-0.02, 0.01)
47-49 years	-0.02 (-0.04, 0.00)	-0.02 (-0.04, 0.00)	-0.02 (-0.03, 0.00)	-0.02 (-0.03, 0.00)	-0.01 (-0.03, 0.01)	-0.01 (-0.03, 0.01)
50-52 years	0	0	0	0	0	0
>52 years	0.00 (-0.01, 0.02)	0.00 (-0.01, 0.02)	0.00 (-0.02, 0.01)	0.00 (-0.02, 0.01)	0.01 (0.00, 0.02)	0.01 (0.00, 0.02)
Parity, binary						
Nulliparous	-0.04 (-0.06, -0.03)	-0.04 (-0.06, -0.03)	-0.07 (-0.08, -0.05)	-0.07 (-0.08, -0.05)	0.04 (0.03, 0.05)	0.04 (0.03, 0.05)
Parous	0	0	0	0	0	0
Parity, ordered categories						
0	0	0	0	0	0	0
1	0.01 (0.00, 0.03)	0.01 (0.00, 0.03)	0.02 (0.01, 0.04)	0.02 (0.01, 0.04)	-0.02 (-0.04, 0.00)	-0.02 (-0.04, 0.00)

For peer review only - http://bmjopen.bmj.com/site/about/guidelines.xhtml

For peer review only

2	0.04 (0.03, 0.06)	0.04 (0.03, 0.06)	0.07 (0.06, 0.08)	0.07 (0.06, 0.08)	-0.04 (-0.06, -0.03)	-0.04 (-0.06, -0.03)
3	0.05 (0.04, 0.07)	0.05 (0.04, 0.07)	0.08 (0.06, 0.09)	0.08 (0.06, 0.09)	-0.04 (-0.06, -0.03)	-0.04 (-0.06, -0.03)
>3	0.05 (0.04, 0.07)	0.05 (0.04, 0.07)	0.08 (0.07, 0.10)	0.08 (0.07, 0.10)	-0.06 (-0.07, -0.04)	-0.06 (-0.07, -0.04)
PCOS/ovarian cysts						
No	0	0	0	0	0	0
Yes	-0.01 (-0.04, 0.03)	-0.01 (-0.04, 0.03)	-0.03 (-0.07, 0.00)	-0.03 (-0.07, 0.00)	0.05 (0.02, 0.09)	0.05 (0.02, 0.09)
Endometriosis						
No	0	0	0	0	0	0
Yes	0.01 (-0.02, 0.05)	0.01 (-0.02, 0.05)	-0.02 (-0.05, 0.01)	-0.02 (-0.05, 0.01)	0.07 (0.03, 0.10)	0.07 (0.03, 0.10)
Oral contraception						
Never used	0	0	0	0	0	0
Ever used	0.03 (0.01, 0.04)	0.03 (0.01, 0.04)	0.02 (0.01, 0.03)	0.02 (0.01, 0.03)	0.01 (0.003, 0.03)	0.01 (0.00, 0.03)
Years of oral contraception						
0	0	0	0	0	0	0
1	0.01 (-0.01, 0.03)	0.01 (0.00, 0.03)	0.01 (-0.01, 0.03)	0.01 (-0.01, 0.03)	0.01 (0.00, 0.03)	0.01 (0.00, 0.03)
2-4	0.02 (0.01, 0.04)	0.02 (0.01, 0.04)	0.02 (0.01, 0.03)	0.02 (0.01, 0.03)	0.02 (0.00, 0.03)	0.02 (0.00, 0.03)
5-9	0.03 (0.02, 0.05)	0.03 (0.02, 0.05)	0.03 (0.01, 0.04)	0.03 (0.01, 0.04)	0.02 (0.00, 0.03)	0.02 (0.00, 0.03)
10-15	0.04 (0.02, 0.05)	0.04 (0.02, 0.05)	0.04 (0.02, 0.05)	0.03 (0.02, 0.05)	0.01 (0.00, 0.03)	0.01 (0.00, 0.03)
>15	0.02 (0.01, 0.04)	0.02 (0.01, 0.04)	0.02 (0.00, 0.03)	0.02 (0.00, 0.03)	0.01 (0.00, 0.03)	0.01 (0.00, 0.03)
HRT						
Never used	0	0	0	0	0	0
Ever used	0.00 (0.00, 0.01)	0.00 (-0.01, 0.01)	0.00 (-0.01, 0.01)	0.00 (-0.01, 0.01)	0.02 (0.01, 0.03)	0.02 (0.01, 0.03)

For peer review only - http://bmjopen.bmj.com/site/about/guidelines.xhtml

Years of HRT						
0	0	0	0	0	0	0
1-2	0.00 (-0.01, 0.02)	0.01 (-0.01, 0.02)	0.00 (-0.02, 0.01)	0.00 (-0.02, 0.01)	0.02 (0.01, 0.04)	0.02 (0.00, 0.04)
3-5	0.00 (-0.01, 0.02)	0.00 (-0.01, 0.02)	0.00 (-0.02, 0.02)	0.00 (-0.01, 0.02)	0.02 (0.00, 0.03)	0.01 (0.00, 0.03)
6-10	0.01 (-0.01, 0.02)	0.01 (-0.01, 0.03)	0.00 (-0.02, 0.02)	0.00 (-0.02, 0.02)	0.02 (0.00, 0.04)	0.02 (0.00, 0.04)
>10	0.00 (-0.01, 0.02)	0.00 (-0.01, 0.02)	0.00 (-0.01, 0.02)	0.00 (-0.01, 0.02)	0.01 (-0.01, 0.03)	0.01 (-0.01, 0.03)
Gynaecological surgery						
No	0	0	0	0	0	0
Hysterectomy	0.01 (0.00, 0.03)	0.02 (0.00, 0.03)	0.00 (-0.02, 0.01)	0.00 (-0.01, 0.01)	0.04 (0.02, 0.06)	0.04 (0.02, 0.06)
Bilateral oophorectomy	-0.02 (-0.08, 0.05)	-0.01 (-0.08, 0.05)	-0.04 (-0.11, 0.03)	-0.04 (-0.11, 0.03)	0.05 (-0.02, 0.12)	0.05 (-0.02, 0.12)
Both	0.00 (-0.01, 0.02)	0.01 (-0.01, 0.02)	-0.02 (-0.04, -0.01)	-0.02 (-0.03, 0.00)	0.05 (0.04, 0.07)	0.05 (0.04, 0.07)

FEV1, forced expiratory volume in one-second; FVC, forced vital capacity; CI, confidence interval; PCOS, polycystic ovary syndrome; HRT, hormone replacement therapy

[a]Adjusted for age, BMI (log-transformed), ethnicity, education, household income, Townsend deprivation index, smoking history in pack-years, asthma, maternal COPD and paternal COPD

[b]Further adjusted from the multiple-adjusted analyses[a] for a history of cardiovascular disease and/or diabetes at baseline

[c]Analysed in women who experienced natural menopause before baseline (n=161 069)

For peer review only - http://bmjopen.bmj.com/site/about/guidelines.xhtml

Table S9. Associations of female reproductive health with incident COPD-related **death** during follow-up (N=273 441)

Reproductive health indicator	% of participants	Person-years of follow-up	Number of COPD deaths	Age-adjusted Hazard ratio	Age-adjusted 95% CI	Multiple-adjusted[a] Hazard ratio	Multiple-adjusted[a] 95% CI
Age at menarche							
<12 years	20.0	332 700	51	1.62	1.15, 2.27	1.52	1.08, 2.13
12-15 years	74.1	1 229 881	115	1.0	NA	1.0	NA
>15 years	5.9	97 672	13	1.49	0.84, 2.65	1.18	0.66, 2.11
Menopausal status							
Pre	26.4	461 595	6	1.0	NA	1.0	NA
Post	73.6	1 198 658	173	2.03	0.76	1.39	0.54, 3.63
Age at natural menopause[b]							
<47 years	20.7	200 666	47	2.47	1.54, 3.97	1.49	0.92, 2.42
47-49 years	12.8	154 965	28	1.92	1.10, 3.34	1.49	0.85, 2.61
50-52 years	23.7	345 140	35	1.0	NA	1.0	NA
>52 years	16.3	275 067	28	0.87	0.51, 1.48	0.96	0.56, 1.64
Parity, binary							
Nulliparous	15.0	248 399	18	0.83	0.51, 1.35	1.04	0.63, 1.70
Parous	85.0	1 411 854	161	1.0	NA	1.0	NA
Parity, ordered categories							
0	15.0	248 399	18	1.0	NA	1.0	NA
1	12.2	201 277	28	1.79	0.99, 3.23	1.24	0.69, 2.26
2	34.4	571 145	44	0.82	0.47, 1.42	0.77	0.45, 1.35

For peer review only - http://bmjopen.bmj.com/site/about/guidelines.xhtml

3	25.3	420 337	41	1.03	0.59, 1.80	0.86	0.49, 1.50
>3	13.2	219 093	48	2.07	1.20, 3.58	1.22	0.70, 2.13
PCOS/ovarian cysts							
No	98.3	1 632 388	175	1.0	NA	1.0	NA
Yes	1.7	27 861	4	1.45	0.54, 3.91	1.52	0.56, 4.11
Endometriosis							
No	98.5	1 635 728	178	1.0	NA	1.0	NA
Yes	1.5	24 526	1	0.50	0.07, 3.60	0.61	0.08, 4.34
Oral contraception							
Never used	19.0	315 862	50	1.0	NA	1.0	NA
Ever used	81.0	1 344 391	129	1.03	0.74, 1.45	0.87	0.62, 1.22
Years of oral contraception							
0	19.0	315 862	50	1.0	NA	1.0	NA
1	11.1	183 949	26	1.30	0.80, 2.12	0.97	0.60, 1.57
2-4	18.7	311 688	33	0.92	0.59, 1.45	0.81	0.52, 1.27
5-9	17.7	294 898	22	0.83	0.50, 1.38	0.74	0.45, 1.24
10-15	17.4	289 787	25	1.04	0.64, 1.71	0.85	0.52, 1.39
>15	16.0	264 067	23	1.25	0.75, 2.09	1.05	0.63, 1.75
HRT							
Never used	63.7	1 024 004	72	1.0	NA	1.0	NA
Ever used	36.6	636 249	107	1.33	0.98, 1.81	0.99	0.73, 1.35
Years of HRT							

For peer review only - http://bmjopen.bmj.com/site/about/guidelines.xhtml

0	63.7	1 024 004	72	1.0	NA	1.0	NA
1-2	11.8	178 712	38	1.53	1.00, 2.34	1.05	0.69, 1.61
3-5	9.1	140 626	35	1.82	1.17, 2.82	1.29	0.83, 2.00
6-10	6.2	94 520	10	0.71	0.34, 1.48	0.64	0.31, 1.33
>10	9.2	222 391	24	1.00	0.60, 1.67	0.83	0.50, 1.38
Gynaecological surgery							
No	81.7	1 350 007	120	1.0	NA	1.0	NA
Hysterectomy	10.1	172 764	36	1.63	1.11, 2.38	1.37	0.94, 2.01
Bilateral oophorectomy	0.4	6130	1	1.50	0.21, 10.71	1.32	0.18, 9.48
Both	7.8	131 351	23	1.40	0.89, 2.20	1.19	0.75, 1.87

COPD, chronic obstructive pulmonary disease; CI, confidence interval; PCOS, polycystic ovary syndrome; HRT, hormone replacement

[a]Multiple-adjusted for age, height, BMI (log-transformed), ethnicity, education, household income, Townsend deprivation index, smoking history, maternal COPD and paternal COPD

[b]Age at menopause was analysed only among women who experienced natural menopause before baseline (n=161 069)

For peer review only

Table S10. Associations of female reproductive health with incident COPD-related hospitalisation/death during follow-up in **complete cases** (N=220 773)

Reproductive health indicator	% of participants[a]	Person-years of follow-up	Number of COPD events	Age-adjusted Hazard ratio	Age-adjusted 95% CI	Multiple-adjusted[b] Hazard ratio	Multiple-adjusted[b] 95% CI
Age at menarche							
<12 years	19.4	257 579	222	1.37	1.17, 1.59	1.20	1.03, 1.41
12-15 years	72.1	958 578	598	1.0	NA	1.0	NA
>15 years	5.6	74 151	71	1.57	1.23, 2.01	1.34	1.05, 1.72
Menopausal status							
Pre	23.8	319 918	53	1.0	NA	1.0	NA
Post	60.4	799 833	673	1.83	1.32, 2.54	1.20	0.85, 1.68
Age at natural menopause[c]							
<47 years	32.4	174 207	224	2.12	1.75, 2.57	1.39	1.12, 1.72
47-49 years	17.9	139 602	124	1.58	1.26, 1.98	1.19	0.92, 1.53
50-52 years	28.1	314 777	194	1.0	NA	1.0	NA
>52 years	21.6	252 180	149	0.89	0.72, 1.10	0.92	0.73, 1.17
Parity, binary							
Nulliparous	15.1	200 749	98	0.81	0.65, 1.00	0.97	0.78, 1.20
Parous	83.3	1 107 959	807	1.0	NA	1.0	NA
Parity, ordered categories							
0	15.1	200 749	98	1.0	NA	1.0	NA
1	11.9	157 577	110	1.36	1.03, 1.78	1.00	0.76, 1.32
2	34.0	453 260	240	0.90	0.71, 1.13	0.84	0.67, 1.07

For peer review only - http://bmjopen.bmj.com/site/about/guidelines.xhtml

3	24.7	328 906	236	1.21	0.95, 1.53	1.03	0.81, 1.31
>3	12.7	168 215	221	2.11	1.66, 2.68	1.43	1.12, 1.83
PCOS/ovarian cysts							
No	98.3	1 307 646	895	1.0	NA	1.0	NA
Yes	1.7	22 016	22	1.47	0.97, 2.25	1.43	0.94, 2.19
Endometriosis							
No	98.5	1 310 012	907	1.0	NA	1.0	NA
Yes	1.5	19 651	10	0.89	0.48, 1.67	0.97	0.52, 1.80
Oral contraception							
Never used	19.1	253 792	222	1.0	NA	1.0	NA
Ever used	80.7	1 072 330	691	1.07	0.92, 1.25	0.94	0.80, 1.10
Years of oral contraception							
0	19.1	253 792	222	1.0	NA	1.0	NA
1	10.7	141 914	108	1.17	0.92, 1.47	0.93	0.74, 1.17
2-4	18.7	249 392	202	1.17	0.97, 1.42	1.04	0.86, 1.27
5-9	17.8	236 848	147	1.03	0.83, 1.27	0.94	0.76, 1.17
10-15	17.5	232 589	130	0.99	0.79, 1.23	0.85	0.68, 1.06
>15	16.0	211 587	104	0.96	0.75, 1.22	0.84	0.66, 1.07
HRT							
Never used	62.2	827 356	352	1.0	NA	1.0	NA
Ever used	37.5	497 994	562	1.78	1.55, 2.05	1.31	1.13, 1.51
Years of HRT							

For peer review only - http://bmjopen.bmj.com/site/about/guidelines.xhtml

0	62.2	827 356	352	1.0	NA	1.0	NA
1-2	10.5	138 603	173	2.12	1.76, 2.56	1.52	1.27, 1.83
3-5	8.3	110 741	117	1.82	1.47, 2.24	1.28	1.04, 1.58
6-10	5.6	74 932	57	1.21	0.92, 1.61	1.03	0.77, 1.36
>10	8.1	107 945	113	1.53	1.23, 1.90	1.24	1.00, 1.54
Gynaecological surgery							
No	81.0	1 077 823	603	1.0	NA	1.0	NA
Hysterectomy	9.7	129 561	163	1.75	1.47, 2.09	1.49	1.25, 1.77
Bilateral oophorectomy	0.4	4611	5	1.63	0.68, 3.94	1.45	0.60, 3.50
Both	7.5	98 833	119	1.67	1.37, 2.04	1.42	1.17, 1.74

COPD, chronic obstructive pulmonary disease; CI, confidence interval; PCOS, polycystic ovary syndrome; HRT, hormone replacement therapy; NA, not applicable

[a]Totals may not equal 100% due to missing values in this complete case analysis

[b]Multiple-adjusted for age, height, BMI (log-transformed), ethnicity, education, household income, Townsend deprivation index, smoking history in pack years, maternal COPD and paternal COPD

[c]Analysed in women who experienced natural menopause before baseline (n=129 961)

For peer review only - http://bmjopen.bmj.com/site/about/guidelines.xhtml

Table S11. Associations of female reproductive health indicators with baseline spirometry measures in **complete cases** (N=160 116)

Reproductive health indicator	Mean change in FEV_1 z-score (95% CI)		Mean change in FVC z-score (95% CI)		Mean change in FEV_1/FVC z-score (95% CI)	
	**Unadjusted**	**Multiple-adjusted[a]**	**Unadjusted**	**Multiple-adjusted[a]**	**Unadjusted**	**Multiple-adjusted[a]**
Age at menarche						
<12 years	-0.05 (-0.07, -0.04)	-0.01 (-0.03, 0.00)	-0.07 (-0.08, -0.06)	0.00 (-0.02, 0.01)	0.02 (0.01, 0.03)	-0.02 (-0.03, -0.01)
12-15 years	0	0	0	0	0	0
>15 years	-0.10 (-0.13, -0.08)	-0.04 (-0.06, -0.02)	-0.09 (-0.11, -0.07)	-0.05 (-0.07, -0.03)	-0.06 (-0.08, -0.04)	0.00 (-0.02, 0.02)
Menopause status						
No	0	0	0	0	0	0
Yes	-0.04 (-0.05, -0.03)	-0.06 (-0.08, -0.04)	-0.03 (-0.05, -0.02)	-0.06 (-0.07, -0.04)	-0.02 (-0.03, -0.01)	-0.02 (-0.03, 0.00)
Age at natural menopause[b]						
<47 years	-0.15 (-0.17, -0.13)	-0.06 (-0.07, -0.04)	-0.13 (-0.15, -0.11)	-0.06 (-0.08, -0.04)	-0.07 (-0.09, -0.05)	-0.01 (-0.03, 0.01)
47-49 years	-0.07 (-0.09, -0.05)	-0.03 (-0.05, -0.01)	-0.06 (-0.08, -0.04)	-0.03 (-0.04, -0.01)	-0.04 (-0.06, -0.02)	-0.01 (-0.03, 0.01)
50-52 years	0	0	0	0	0	0
>52 years	0.01 (-0.01, 0.02)	0.00 (-0.02, 0.02)	-0.01 (-0.03, 0.00)	-0.01 (-0.03, 0.01)	0.04 (0.03, 0.06)	0.02 (0.00, 0.03)
Parity, binary						
Nulliparous	-0.03 (-0.04, -0.01)	-0.06 (-0.07, -0.04)	-0.05 (-0.07, -0.04)	-0.08 (-0.09, -0.07)	0.05 (0.03, 0.06)	0.05 (0.03, 0.06)
Parous	0	0	0	0	0	0
Parity, ordered categories						
0	0	0	0	0	0	0
1	-0.03 (-0.05, -0.02)	0.01 (0.00, 0.03)	-0.01 (-0.02, 0.01)	0.03 (0.01, 0.04)	-0.06 (-0.08, -0.04)	-0.02 (-0.04, -0.01)
2	0.06 (0.04, 0.08)	0.06 (0.05, 0.07)	0.08 (0.07, 0.10)	0.09 (0.07, 0.10)	-0.03 (-0.05, -0.02)	-0.05 (-0.06, -0.03)

For peer review only - http://bmjopen.bmj.com/site/about/guidelines.xhtml

3	0.04 (0.03, 0.06)	0.07 (0.05, 0.08)	0.07 (0.05, 0.09)	0.10 (0.08, 0.11)	-0.05 (-0.06, -0.03)	-0.05 (-0.07, -0.03)
>3	-0.04 (-0.06, -0.02)	0.06 (0.04, 0.08)	-0.01 (-0.03, 0.01)	0.10 (0.08, 0.11)	-0.07 (-0.09, -0.06)	-0.06 (-0.08, -0.04)
PCOS/ovarian cysts						
No	0	0	0	0	0	0
Yes	-0.02 (-0.06, 0.02)	0.00 (-0.04, 0.03)	-0.06 (-0.09, -0.02)	-0.03 (-0.07, 0.00)	0.07 (0.03, 0.11)	0.07 (0.03, 0.11)
Endometriosis						
No	0	0	0	0	0	0
Yes	-0.01 (-0.05, 0.04)	0.00 (-0.03, 0.04)	-0.04 (-0.08, 0.01)	-0.03 (-0.07, 0.01)	0.06 (0.02, 0.10)	0.06 (0.03, 0.10)
Oral contraception						
Never used	0	0	0	0	0	0
Ever used	0.08 (0.07, 0.09)	0.03 (0.02, 0.04)	0.09 (0.08, 0.11)	0.03 (0.02, 0.04)	-0.01 (-0.02, 0.00)	0.02 (0.01, 0.03)
Years of oral contraception						
0	0	0	0	0	0	0
1	0.04 (0.02, 0.06)	0.02 (0.00, 0.03)	0.05 (0.03, 0.07)	0.01 (-0.01, 0.03)	-0.03 (-0.05, -0.01)	0.01 (-0.01, 0.03)
2-4	0.07 (0.05, 0.08)	0.03 (0.01, 0.05)	0.07 (0.06, 0.09)	0.02 (0.01, 0.04)	0.00 (-0.01, 0.02)	0.02 (0.01, 0.04)
5-9	0.10 (0.08, 0.11)	0.04 (0.02, 0.05)	0.11 (0.09, 0.12)	0.03 (0.02, 0.05)	0.00 (-0.01, 0.02)	0.02 (0.00, 0.04)
10-15	0.10 (0.09, 0.12)	0.05 (0.03, 0.06)	0.12 (0.11, 0.14)	0.05 (0.03, 0.06)	-0.02 (-0.03, 0.00)	0.02 (0.00, 0.03)
>15	0.08 (0.07, 0.10)	0.03 (0.01, 0.04)	0.10 (0.08, 0.12)	0.02 (0.01, 0.04)	-0.03 (-0.05, -0.01)	0.01 (-0.01, 0.03)
HRT						
Never used	0	0	0	0	0	0
Ever used	-0.03 (-0.04, -0.02)	0.00 (-0.01, 0.01)	-0.01 (-0.02, 0.00)	-0.01 (-0.02, 0.00)	-0.04 (-0.05, -0.03)	0.02 (0.01, 0.03)
Years of HRT						

For peer review only - http://bmjopen.bmj.com/site/about/guidelines.xhtml

0	0	0	0	0	0	0
1-2	-0.03 (-0.05, -0.02)	0.00 (-0.02, 0.01)	-0.02 (-0.03, 0.00)	-0.01 (-0.03, 0.01)	-0.05 (-0.06, -0.03)	0.02 (0.00, 0.03)
3-5	-0.04 (-0.06, -0.02)	0.00 (-0.02, 0.02)	-0.03 (-0.05, -0.01)	0.00 (-0.02, 0.02)	-0.04 (-0.05, -0.02)	0.01 (-0.01, 0.03)
6-10	0.00 (-0.02, 0.02)	0.01 (-0.01, 0.03)	0.01 (-0.02, 0.03)	0.00 (-0.02, 0.02)	-0.01 (-0.03, 0.01)	0.02 (0.00, 0.04)
>10	-0.01 (-0.03, 0.01)	0.00 (-0.02, 0.02)	0.01 (-0.01, 0.03)	0.00 (-0.02, 0.02)	-0.03 (-0.05, -0.02)	0.01 (-0.01, 0.03)
Gynaecological surgery						
No	0	0	0	0	0	0
Hysterectomy	-0.04 (-0.06, -0.03)	0.02 (0.00, 0.04)	-0.06 (-0.08, -0.05)	0.00 (-0.01, 0.02)	0.03 (0.02, 0.05)	0.05 (0.03, 0.07)
Bilateral oophorectomy	-0.07 (-0.15, 0.01)	-0.05 (-0.13, 0.03)	-0.10 (-0.18, -0.01)	-0.08 (-0.16, 0.00)	0.05 (-0.03, 0.14)	0.06 (-0.02, 0.14)
Both	-0.05 (-0.07, -0.03)	0.00 (-0.02, 0.02)	-0.08 (-0.10, -0.06)	-0.03 (-0.04, -0.01)	0.06 (0.04, 0.08)	0.06 (0.04, 0.08)

FEV_1, forced expiratory volume in one-second; FVC, forced vital capacity; CI, confidence interval; PCOS, polycystic ovary syndrome; HRT, hormone replacement therapy; NA, not applicable

[a]Adjusted for age, BMI (log-transformed), ethnicity, education, household income, Townsend deprivation index, smoking history pack-years, asthma, maternal COPD and paternal COPD

[b]Analysed in women who experienced natural menopause before baseline (n=121 153)

For peer review only - http://bmjopen.bmj.com/site/about/guidelines.xhtml

STROBE 2007 (v4) Statement—Checklist of items that should be included in reports of *cohort studies*

Section/Topic	Item #	Recommendation	Reported on page #
**Title and abstract**	1	(*a*) Indicate the study's design with a commonly used term in the title or the abstract	1
		(*b*) Provide in the abstract an informative and balanced summary of what was done and what was found	2
**Introduction**			
Background/rationale	2	Explain the scientific background and rationale for the investigation being reported	4
Objectives	3	State specific objectives, including any prespecified hypotheses	4-5
**Methods**			
Study design	4	Present key elements of study design early in the paper	6-7
Setting	5	Describe the setting, locations, and relevant dates, including periods of recruitment, exposure, follow-up, and data collection	5
Participants	6	(*a*) Give the eligibility criteria, and the sources and methods of selection of participants. Describe methods of follow-up	5
		(*b*) For matched studies, give matching criteria and number of exposed and unexposed	
Variables	7	Clearly define all outcomes, exposures, predictors, potential confounders, and effect modifiers. Give diagnostic criteria, if applicable	5-7
Data sources/ measurement	8*	For each variable of interest, give sources of data and details of methods of assessment (measurement). Describe comparability of assessment methods if there is more than one group	5-7
Bias	9	Describe any efforts to address potential sources of bias	6-7
Study size	10	Explain how the study size was arrived at	8
Quantitative variables	11	Explain how quantitative variables were handled in the analyses. If applicable, describe which groupings were chosen and why	6-7
Statistical methods	12	(*a*) Describe all statistical methods, including those used to control for confounding	6-7
		(*b*) Describe any methods used to examine subgroups and interactions	7
		(*c*) Explain how missing data were addressed	7
		(*d*) If applicable, explain how loss to follow-up was addressed	7
		(*e*) Describe any sensitivity analyses	7
**Results**			

For peer review only

Participants	13*	(a) Report numbers of individuals at each stage of study—eg numbers potentially eligible, examined for eligibility, confirmed eligible, included in the study, completing follow-up, and analysed	8 and Figure 1
		(b) Give reasons for non-participation at each stage	8 and Figure 1
		(c) Consider use of a flow diagram	Figure 1
Descriptive data	14*	(a) Give characteristics of study participants (eg demographic, clinical, social) and information on exposures and potential confounders	8 and 21-24
		(b) Indicate number of participants with missing data for each variable of interest	8 and 21-24
		(c) Summarise follow-up time (eg, average and total amount)	8
Outcome data	15*	Report numbers of outcome events or summary measures over time	8 and 25-28
Main results	16	(*a*) Give unadjusted estimates and, if applicable, confounder-adjusted estimates and their precision (eg, 95% confidence interval). Make clear which confounders were adjusted for and why they were included	25-32
		(*b*) Report category boundaries when continuous variables were categorized	5
		(*c*) If relevant, consider translating estimates of relative risk into absolute risk for a meaningful time period	
Other analyses	17	Report other analyses done—eg analyses of subgroups and interactions, and sensitivity analyses	10 and online supplementary file
**Discussion**			
Key results	18	Summarise key results with reference to study objectives	10
**Limitations**			
Interpretation	20	Give a cautious overall interpretation of results considering objectives, limitations, multiplicity of analyses, results from similar studies, and other relevant evidence	11-12
Generalisability	21	Discuss the generalisability (external validity) of the study results	13-14
**Other information**			
Funding	22	Give the source of funding and the role of the funders for the present study and, if applicable, for the original study on which the present article is based	2

*Give information separately for cases and controls in case-control studies and, if applicable, for exposed and unexposed groups in cohort and cross-sectional studies.

Note: An Explanation and Elaboration article discusses each checklist item and gives methodological background and published examples of transparent reporting. The STROBE checklist is best used in conjunction with this article (freely available on the Web sites of PLoS Medicine at http://www.plosmedicine.org/, Annals of Internal Medicine at http://www.annals.org/, and Epidemiology at http://www.epidem.com/). Information on the STROBE Initiative is available at www.strobe-statement.org.

For peer review only

BMJ Open

Female reproductive history in relation to chronic obstructive pulmonary disease and lung function in UK Biobank: a prospective population-based cohort study

Journal:	*BMJ Open*
Manuscript ID	bmjopen-2019-030318.R2
Article Type:	Original research
Date Submitted by the Author:	05-Sep-2019
Complete List of Authors:	Tang, Rosalind; University of Bristol Faculty of Health Sciences<br>Fraser, Abigail; University of Bristol, MRC Integrative Epidemiology Unit; University of Bristol, Department of Population Health Sciences<br>Magnus, Maria; University of Bristol, Department of Population Health Sciences; Norwegian Institute of Public Health, Centre for Fertility and Health
**Primary Subject Heading**:	Respiratory medicine
Secondary Subject Heading:	Reproductive medicine, Epidemiology, Obstetrics and gynaecology
Keywords:	chronic obstructive pulmonary disease, spirometry, menarche, menopause, oral contraceptives, hormone replacement therapy

SCHOLARONE™
Manuscripts

Female reproductive history in relation to chronic obstructive pulmonary disease and lung function in UK Biobank: a prospective population-based cohort study

Authors: Rosalind Tang[1,2]*, Abigail Fraser[3,4], Maria C. Magnus[3,4,5]

Author affiliations: [1]Bristol Medical School, Faculty of Health Sciences, University of Bristol, Bristol, United Kingdom; [2]Keenan Research Centre for Biomedical Science, St. Michael's Hospital, Toronto, Canada; [3]MRC Integrative Epidemiology Unit at the University of Bristol, Bristol, United Kingdom; [4]Department of Population Health Sciences, Bristol Medical School, University of Bristol, Bristol, United Kingdom; [5]Centre for Fertility and Health, Norwegian Institute of Public Health, Oslo, Norway.

***Corresponding author:** Rosalind Tang

Address: Bristol Medical School, University of Bristol, 5 Tyndall Avenue, Bristol, BS8 1UD, United Kingdom

Email: rt16358@bristol.ac.uk

For peer review only

For peer review only - http://bmjopen.bmj.com/site/about/guidelines.xhtml

ABSTRACT

Objectives: Sex differences in respiratory physiology and predilection for developing chronic obstructive pulmonary disease (COPD) have been documented, suggesting that female sex hormones may influence pathogenesis. We investigated whether aspects of female reproductive health might play a role in risk of COPD among women.

Design: Population-based prospective cohort study.

Setting: UK Biobank recruited across 22 centres in the United Kingdom between 2006-2010.

Primary and secondary outcomes measures: We examined a range of female reproductive health indicators in relation to risk of COPD-related hospitalisation/death (n=271 271) using Cox proportional hazards regression; and lung function (n=273 441) using linear regression.

Results: Parity >3 was associated with greater risk of COPD-related hospitalisation/death (adjusted HR 1.45; 95% CI: 1.16, 1.82) and lower FEV_1/FVC (adjusted mean difference -0.06; 95% CI: -0.07, -0.04). Any oral contraception use was associated with lower risk of COPD-related hospitalisation/death (adjusted HR 0.85; 95% CI: 0.74, 0.97) and greater FEV_1/FVC (adjusted mean difference 0.01; 95% CI: 0.003, 0.03). Late menarche (age >15) and early menopause (age <47) were also associated with greater risk of COPD-related hospitalisation/death (but not lung function), while endometriosis was associated with greater FEV_1/FVC (not COPD-related hospitalisation/death). Early menarche (age <12 years) was associated with lower FEV_1/FVC (but not COPD hospitalisation/death). Associations with PCOS or ovarian cysts; any HRT use; hysterectomy-alone; and both hysterectomy and bilateral oophorectomy were in opposing directions for COPD-related hospitalisation/death (greater risk) and FEV_1/FVC (positive association).

Conclusions: Multiple female reproductive health indicators across the life course are associated with COPD-related hospitalisation/death and lung function. Further studies are necessary to understand the opposing associations of PCOS/ovarian cysts, HRT and hysterectomy with COPD and objective measures of airway obstruction.

For peer review only - http://bmjopen.bmj.com/site/about/guidelines.xhtml

Strengths and limitations of this study

- This large, population-based, prospective cohort study is the first to assess COPD risk with age at menarche, menopause status, hysterectomy, gynaecological surgery, endometriosis or PCOS.
- No study has examined a wide range of reproductive health indicators, spanning the reproductive and post-reproductive periods, with regards to COPD.
- We included a comprehensive list of potential confounders, such as age, height, body mass index, ethnicity, education, household income, Townsend deprivation index, smoking history, asthma, maternal COPD and paternal COPD.
- We relied on participants to self-report reproductive history and we cannot exclude a potential influence of selection bias, due to the low participation rate in UK Biobank.
- Data on environmental tobacco smoke were not available and we cannot exclude residual bias from this exposure.

For peer review only

For peer review only - http://bmjopen.bmj.com/site/about/guidelines.xhtml

INTRODUCTION

There are clear sex differences in lung anatomy and physiology throughout the life course.[1] This is also reflected in the risk of obstructive lung disease. For example, boys have greater risk of asthma during early childhood compared to girls, but this sex difference reverses after puberty, when girls are found to have the greater risk of new-onset asthma.[2] Chronic obstructive pulmonary disease (COPD) has traditionally been seen as a disease predominantly affecting men. However, in more recent years, the overall burden of the disease has shifted more towards women.[2 3] Women with COPD also have, on average, fewer pack-years of smoking history than men with COPD; and non-smokers with COPD are more often female.[1] This points towards a greater underlying susceptibility among women, and highlights the importance of understanding female-specific risk factors for COPD.

Female sex hormones are putative mediators of respiratory health, and regulate bronchodilation, cell proliferation, inflammation and metabolism of toxic cigarette smoke-related metabolites.[1 4-7] Notably, oestrogen and progesterone appear to have varying effects on lung function across the lifespan.[1] We know little, however, about female reproductive health indicators in relation to COPD risk.[1] These reproductive health indicators include markers of endogenous hormone exposure (age at menarche, age at menopause and length of reproductive lifespan), exogenous hormone exposure (oral contraceptive use and hormone replacement therapy), as well as pregnancy history. Of two existing studies, one study found no evidence of an association between hormone replacement therapy (HRT) and COPD risk,[8] while another reported a positive association between early age at first pregnancy (<20 years) and COPD-related mortality.[9] There is more evidence regarding the relationship between female reproductive health indicators and lung function. Early menarche,[10-12] nulliparity,[13] younger age at first pregnancy,[12] and postmenopausal status[14-19] have each been associated with reduced lung function. Evidence of the effects of HRT is contradictory.[14 15 20 21] In the present study, we therefore investigate associations of multiple female reproductive health

For peer review only - http://bmjopen.bmj.com/site/about/guidelines.xhtml

indicators across the lifespan with incidence of COPD hospitalisation/death and lung function in the UK Biobank cohort.

METHODS

Study population

Our study included female participants in UK Biobank (Figure 1), a population-based cohort study which recruited men and women age 40-69 years across 22 centres in England, Wales and Scotland between 2006-2010.[22] The response rate was 5.5%.[22] All participants provided written informed consent. Data on 273 441 women were available after withdrawals. At baseline, participants self-reported sociodemographic information, family medical history, early-life exposures, psychosocial history and medical history. Physical measurements included spirometry. Information on health outcomes after recruitment into the cohort was gathered prospectively via data linkages with hospital and death registers. Register data were available up to 28 February 2015 for England, 16 March 2015 for Wales and 28 October 2014 for Scotland. Data collection in UK Biobank was approved by the NHS National Research Ethics Service (Ref 11/NW/0382). UK Biobank does not require separate ethics approvals for specific analyses. A generic Research Tissue Bank approval for access to UK Biobank data and/or samples has been recommended by the National Research Ethics Service and has been obtained from the governing UK Biobank Research Ethics Committee (http://www.ukbiobank.ac.uk/wp-content/uploads/2012/09/Access-Procedures-2011-1.pdf). Applications to access the resource are approved by the UK Biobank Access Sub-Committee and Ethics & Governance Council.

Female reproductive health indicators

We investigated the following self-reported female reproductive health indicators: age at menarche (<12, 12-15, and >15 years), menopause status (no, yes), age at natural menopause (<47, 47-49, 50-52 and >52 years), parity (0, 1, 2, 3, and >3), history of polycystic ovary syndrome (PCOS) or ovarian cysts, history of endometriosis, oral contraception (OC) use (never/ever), years of OC use (0, 1, 2-4,

For peer review only

5-9, 10-15 and >15), hormone replacement therapy (HRT) use (never/ever), years of HRT use (0, 1-2, 3-5, 6-10, and >10), and gynaecological surgery (hysterectomy, oophorectomy and hysterectomy with bilateral oophorectomy). We calculated years of OC and HRT use as the differences between age at first and last use, and created categories according to quintiles.

Amaral et al. previously reported decreased lung function in postmenopausal women in UK Biobank.[16] We repeat these analyses for completeness alongside analyses of other female reproductive health indicators.

For peer review only

Respiratory outcomes

Prevalent COPD at baseline was identified using self-report and registrations before baseline in the hospital registers. Incident hospitalisations/deaths from COPD after baseline were identified using hospital and death registers. The following International Classification of Diseases (ICD) codes were used to capture COPD: ICD-9 codes 490-492, 494 and 496; or ICD-10 codes J40-J44.

Forced expiratory volume at one-second (FEV_1) and forced vital capacity (FVC) were measured using Vitalograph Pneumotrac 6800 (Vitalograph, UK) at baseline. Spirometry was not performed if participants had experienced a chest infection in the last month; had a lifetime history of detached retina or collapsed lung; had experienced a heart attack, eye surgery or surgery to the chest or abdomen in the last 3 months; were currently pregnant in the first or third trimester; or were currently using medication for tuberculosis. Two measurements were conducted if the difference between these measurements was ≤5%. A third measurement was conducted if the difference was >5%. We used the single greatest measurements of FEV_1 and FVC per participant. Post-bronchodilator spirometry was not available, although drug treatment was not withheld. Spirometry measurements were converted to internally standardised z-scores by age and height based on the Global Lung Initiative 2012 recommendations[23] to reduce bias related to age. These z-scores were used for all analyses.

For peer review only - http://bmjopen.bmj.com/site/about/guidelines.xhtml

Statistical analyses

We used Cox proportional hazards regression to examine the association of each female reproductive health indicator in relation to incidence of COPD hospitalisation/death. Participants without COPD at baseline were followed from enrolment until hospitalisation/death from COPD, death from other causes, or the end of follow-up if the participants were still alive and disease-free. The time axis for the Cox regression was calendar time. We examined deviations from the proportional hazard assumption using Schoenfeld residuals.[24] Age- and confounder-adjusted estimates are reported as hazard ratios (HR) with 95% confidence intervals (95% CI). The following potential confounders were included in multivariable analyses: age, height, log-transformed body mass index (BMI) at baseline, ethnicity (White or non-White), educational attainment (college, university or other professional degree; Advanced levels, Advanced Subsidiary levels or equivalent; Ordinary levels, General Certificate of Secondary Educations or equivalent; Certificate of Secondary Educations or equivalent; National Vocational Qualifications, Higher National Diploma, Higher National Certificate or equivalent; other), annual household income (<£18 000; £18 001-31 000; £31 001-£52 000; £52 001-£100 000; >£100 000), Townsend deprivation index, smoking history in pack-years (none, <11, 11-20, 21-30, >30), and parental history of COPD. We performed sensitivity analyses of 1) menopause status and age at natural menopause with further adjustment for HRT; 2) menopause status excluding participants age <45 years or >60 years at baseline to reduce potential bias by age; 3) all reproductive health indicators after excluding participants with respiratory conditions (i.e. COPD, asthma, tuberculosis or pneumonia) reported at baseline or $FEV_1/FVC<0.7$ at baseline, as they possibly had undiagnosed COPD;[25] 4) all reproductive health indicators with smoking history adjusted as two covariates (duration in years and average number of cigarettes/day) rather than one covariate (pack-years); 5) all reproductive health indicators with further adjustment for baseline smoking status (never, previous, current); 6) all reproductive health indicators after excluding participants with any history of smoking; and 7) all reproductive health indicators with further adjustment for other comorbidities (cardiovascular disease or diabetes) reported at baseline.

For peer review only - http://bmjopen.bmj.com/site/about/guidelines.xhtml

We used linear regression in the analysis of standardised spirometry measurements. The multivariable linear regression models were adjusted for the same covariates as for hospitalisation/death from COPD, with the addition of asthma at baseline. We reported results as mean difference in the spirometry measurements z-scores with 95% CI. These mean differences in z-scores can be converted to raw values by multiplication with the standard deviation in spirometry measurement for the given age and height group. Sensitivity analyses for standardised spirometry measurements were also conducted with: 1) menopause status excluding participants age <45 years or >60 years at baseline; 2) smoking history adjusted as two covariates (duration in years and average number of cigarettes/day); 3) further adjustment for baseline smoking status; and 4) further adjustment for comorbidities baseline (cardiovascular disease or diabetes).

We performed multiple imputation by chained equations to account for missing data. Exposures, covariates and outcomes were included in the imputation model. We imputed 20 datasets, which were then analysed using Rubin's rules.[26] Details of the imputation model are given in the online supplementary file (Table S1). All analyses were performed using Stata/MP 15 (StataCorp LLC, College Station, USA). Sensitivity analyses of the complete cases was performed for comparison with our imputed data.

Patient and public involvement

The present study did not involve patients and the public.

RESULTS

Of the 273 441 women included in this study, 2170 had prevalent COPD at baseline (Figure 1). These prevalent cases were excluded from the analysis of incident COPD-related hospitalisation/death. By the end of follow-up (median 6 years), there were 1138 incident cases of hospitalisation/death from COPD, yielding an incidence rate of 0.19 per 100 000 person years.

For peer review only - http://bmjopen.bmj.com/site/about/guidelines.xhtml

In UK Biobank, 28.4% of women did not have spirometry data, 16.1% were uncertain of or did not report menopause status and 14.3% did not report smoking history. The proportion of missing information on the other covariates were modest (Table 1). Overall, women with spirometry data were younger, more often White, had higher educational attainment, higher annual household income, less deprivation (lower Townsend index), greater smoking history and were more likely to have asthma than women who did not undergo spirometry assessment (online supplementary file Table S2). The distribution of participants' characteristics across the imputed datasets is shown in Table 1.

Late (age >15 years) menarche was associated with increased risk of hospitalisation/death from COPD compared to menarche between age 12-15. The multiple-adjusted HR was 1.37 (95% CI: 1.11, 1.71) (Table 2). There was a modest association between early menarche and COPD hospitalisation/death (HR 1.15; 95% CI: 1.00, 1.32). Early menarche (age <12 years) was associated with lower FEV_1/FVC (Table 3).

Risk of hospitalisation/death from COPD did not differ between pre- and postmenopausal women (Table 2), and this finding was similar after further adjusting for HRT use (HR 1.02; 95% CI: 0.78, 1.35). In examining age at menopause and risk hospitalisation/death from COPD among women who experienced natural menopause (n=159 571), the risk was higher in women who experienced menopause age <47 years (HR 1.44; 95% CI: 1.19, 1.75) when compared to age 50-52 (Table 2). This association remained after further adjustment for HRT use (HR 1.43; 95% CI: 1.18, 1.74). Postmenopausal status at baseline and early natural menopause were each associated with lower FEV_1 and FVC but not FEV_1/FVC (Table 3). These associations did not change after further adjustment for HRT ever use (results not shown). After restricting the sample to participants who were age 45-60 years (inclusive) at the time of reporting menopause status to reduce residual bias due to age, we found that menopause status was positively associated with COPD-related hospitalisation/death (HR

For peer review only - http://bmjopen.bmj.com/site/about/guidelines.xhtml

1.57; 95% CI: 1.07, 2.30), whilst associations with spirometry measures did not change (results not shown).

Hospitalisation/death from COPD did not differ between parous and nulliparous women, but parity >3 was associated with increased risk (HR 1.45; 95% CI: 1.16, 1.82) compared to nulliparity. Women who were nulliparous had lower FEV_1 and FVC but greater FEV_1/FVC compared to parous women, while women with parity >3 had greater FEV_1 and FVC but lower FEV_1/FVC compared to nulliparous women (Table 3).

A history of PCOS or ovarian cysts was associated with increased risk of hospitalisation/death from COPD (HR 1.61; 95% CI: 1.12, 2.32) and greater FEV_1/FVC (mean difference 0.05; 95% CI: 0.02, 0.09), while there was no evidence of an association with FEV_1 or FVC. A history of endometriosis was not associated with COPD hospitalisation/death, FEV_1 or FVC (Tables 2 and 3), though we observed a modest positive association with FEV_1/FVC (mean difference 0.07; 95% CI: 0.03, 0.10).

We observed several associations between exogenous hormone use and COPD hospitalisation/death. Women who had ever used OC had lower risk of hospitalisation/death from COPD compared to never users (HR 0.85; 95% CI: 0.74, 0.97) (Table 2). This appeared to be driven by OC use of >9 years (Table 2). In line with the observed findings for COPD, OC ever use and duration of use showed positive associations with FEV_1, FVC and FEV_1/FVC (Table 3).

Ever using HRT was associated with a modest increased risk of hospitalisation/death from COPD (HR 1.15; 95% CI: 1.01, 1.30) (Table 2). We observed no associations of HRT ever use with FEV_1 or FVC, but a positive association with FEV_1/FVC (Table 3). When we examined the duration of HRT use, we only observed increased hospitalisation/death from COPD (HR 1.33; 95% CI: 1.12, 1.57) and greater FEV_1/FVC (mean difference 0.02; 95% CI: 0.01, 0.04) with 1-2 years of use (Tables 2 and 3). There was no evidence of a dose-response relationship.

For peer review only - http://bmjopen.bmj.com/site/about/guidelines.xhtml

A history of hysterectomy (with or without bilateral oophorectomy), but not bilateral oophorectomy-alone, were associated with greater risk of COPD hospitalisation/death (Table 2). At the same time, hysterectomy was associated with higher, rather than lower, FEV_1/FVC (Tables 3). Hysterectomy with bilateral oophorectomy was also associated with lower FVC (Table 3).

The directions of association between individual female reproductive health indicators with COPD risk and spirometry measures are summarised in Table 4. 55 929 women had reported either a respiratory illness (asthma, COPD, tuberculosis and/or pneumonia) and/or FEV_1/FVC<0.7 at baseline. However, our findings after excluding these women from our analyses of COPD-related hospitalisation/death were similar to our main findings (online supplementary file Table S3).

We report the results of our sensitivity analyses, after 1) further adjusting for baseline smoking status; 2) adjusting for smoking history as two covariates including duration in years of smoking and average number of cigarettes per day; 3) excluding women who have ever smoked; or 4) further adjusting for cardiovascular disease or diabetes reported at baseline, in the online supplementary file (Tables S4-S8). The direction and magnitude of associations remained similar for risk of COPD hospitalisation/death (online supplementary file Tables S4 and S6) and change in spirometry measures (online supplementary file Tables S7 and S8) after further adjustment for the above covariates. In our analyses of never smokers (n=161 626), however, associations of COPD-related hospitalisation/death with early menopause, parity>3, PCOS/ovarian cysts, ever using oral contraception or hormone replacement therapy became null (online supplementary file Table S5). Early age at menarche (<12 years) was also positively associated with COPD-related hospitalisation/death in this restricted sample of never smokers (HR 1.39; 95% CI: 1.02, 1.90), whilst the remaining associations remained unchanged from the main analyses.

We also investigated associations of reproductive health indicators with risk of COPD-related mortality, and not hospitalisation, among women of UK Biobank. The associations from these analyses of 273 441 women (including those with COPD reported at baseline) were null for all

For peer review only - http://bmjopen.bmj.com/site/about/guidelines.xhtml

For peer review only

reproductive health indicators (online supplementary file Table S9), except age at menarche <12 years compared to 12-15 years (HR 1.52; 95% CI: 1.08, 2.13). However, there were only 179 COPD-related deaths in UK Biobank and many categories of our reproductive health indicators experienced fewer than ten events. Complete-case analyses of the risk of COPD hospitalisation/death (online supplementary file Table S10) and lung function (online supplementary file Table S11) also produced similar results to those from our imputed dataset.

DISCUSSION

In this large, prospective study, several female reproductive health indicators were associated with COPD hospitalisation/death and/or lung function. Parity greater than three was associated with decreased baseline FEV_1/FVC, a marker of airway obstruction, and increased risk of hospitalisation/death from COPD during follow-up. Ever using OC was associated with greater FEV_1/FVC and lower risk of COPD hospitalisation/death. Associations of hysterectomy, HRT use, and a history of PCOS or ovarian cysts showed inconsistent findings for FEV_1/FVC and COPD hospitalisation/death. This inconsistency may be explained by the fact that spirometry was measured in participants at baseline (age 40-69) and FEV_1 and FEV_1/FVC may have declined during follow-up. Conversely, many patients diagnosed with stage 1 COPD do not go on to develop COPD symptoms or COPD resulting in hospitalisation/death.[27] There is also high intra-individual variability in FEV_1.[28] Spirometry assessment at one time point may therefore not be as accurate at identifying clinically-relevant COPD compared to register data on hospitalisation/death.

A study of 424 797 postmenopausal women in the United States reported no difference in COPD mortality by parity.[9] In contrast, our present study reports higher risk of COPD hospitalisation/death among women with more than three births compared to nulliparous women. However, we investigated both COPD-related hospitalisation and death, and we were therefore able to capture less severe COPD cases. A study of 70 965 women in the Nurses' Health Study reported no evidence of an association between HRT ever use and COPD incidence, although the investigators included

For peer review only - http://bmjopen.bmj.com/site/about/guidelines.xhtml

both probable (self-reported physician diagnosis along with a record of spirometry or imaging at the time of diagnosis or COPD documented on a death register) and definite (self-reported physician diagnosis and FEV_1 <80% predicted) cases of COPD.[8] Here we report a positive association between HRT use and COPD hospitalisation/death. This is the first study to assess COPD risk with regards to age at menarche, menopause status, hysterectomy, gynaecological surgery, endometriosis or PCOS.

Two previous studies reported reduced FEV_1 in adult women who experienced menarche before age 10[10 12] but neither found evidence of an association between age at menarche and FEV_1/FVC. A Mendelian randomisation study of adolescent and adult women across multiple European cohorts, including a subset from UK Biobank, estimated a 24.8 mL increase in FVC per year increase in age at menarche among adult women and a -56.6 mL decrease in FVC per year increase in age at menarche among adolescent girls.[11] This study reported no association between age at menarche and FEV_1/FVC.[11] Notably, this Mendelian randomisation study did not explore non-linear associations between age at menarche and measures of lung function. We observed lower FEV_1 and FVC in adult women with late menarche, and lower FVC and FEV_1/FVC in adult women with early menarche.

A recent systematic review, which included studies from UK Biobank,[16] reported that postmenopausal status was associated with reduced FEV_1 and FVC but not FEV_1/FVC.[18] Current HRT use has been associated with both higher[14] and lower[15] lung function. One study reported an association between HRT use and greater FEV_1/FVC.[14] Others have identified differences in lung function according to continuous/cyclic and combined/oestrogen-only HRT.[20 21] Based on the information from participants in UK Biobank, we could not stratify by HRT subtypes.

There are sex differences in lung anatomy and physiology throughout the life course.[1 2 4] During gestation, female foetuses have fewer bronchi, smaller lungs and produce surfactant earlier than male foetuses.[1] Female neonates and children have greater lung function and faster lung growth than their male counterparts, and boys are more likely to be diagnosed with asthma during childhood than girls, while girls are twice as likely as boys to be diagnosed with asthma after

For peer review only - http://bmjopen.bmj.com/site/about/guidelines.xhtml

puberty.[1] These findings suggest that female sex steroids can accelerate lung maturation and termination of lung growth, which may partially explain why early menarche associates with lower FEV_1/FVC in adulthood. During the reproductive years, parity and PCOS relate to an increase in circulating oestrogen, which has been implicated in the pathogenesis of inflammatory lung diseases including COPD.[6 29-31] Although OC use appears to be protective against COPD, some researchers have suggested that OC use attenuates the fluctuations in sex steroids, which correlates with fluctuations in endothelial nitric oxide, a potent bronchodilator.[1] Additionally, exogenous hormones may not be as influential during reproductive years but may become more influential in after menopause, when endogenous hormone production is low.[6] This is supported by our findings of opposing associations with COPD risk for OC use (lower risk) during reproductive period and HRT use (higher risk) after menopause.

The effect of female sex hormones on COPD and lung health may vary depending on the net effects on airway smooth muscle, inflammation and cigarette smoke metabolism. Oestrogens can regulate the synthesis of nitric oxide, a potent bronchodilator, within human bronchial epithelium,[1 32] as well as regulate airway bronchoconstriction in response to acetylcholine or histamine.[33 34] There is also evidence that progesterone may affect airway smooth muscle constriction, although these reports are limited to animal studies.[35] In addition to regulating bronchodilation and bronchoconstriction, in vitro studies report that oestrogen can also modulate the proliferation of airway smooth muscle cells,[36 37] which is a key feature of COPD.[38] Oestrogen's pro- and anti-inflammatory effects have been documented in other female-predominant diseases[39] and may also play a role in COPD, as chronic inflammation is another hallmark of the disease. Finally, oestrogen is an important regulator of toxic cigarette smoke metabolites through cytochrome P450 enzymes.[1] In a mouse model of COPD, Glassberg et al. reported that oestradiol administration after ovariectomy protected against cigarette smoke-induced alveolar septal destruction, macrophage infiltration and decreased other markers of inflammation compared to ovariectomy without oestradiol administration.[40] In the same mouse model, however, non-ovariectomised female mice also had increased small airway

For peer review only - http://bmjopen.bmj.com/site/about/guidelines.xhtml

For peer review only

remodelling following cigarette smoke exposure when compared to both male mice and ovariectomised female mice.[41] These timing-, dose- and cell-dependent signalling pathways may explain why exposure to female sex steroids, and oestrogen in particular, have potentially contradictory effects on respiratory health.

We cannot exclude a potential influence of selection bias, due to the low participation rate in UK Biobank. Women in the cohort are less likely to have ever smoked, have greater educational attainment, but a similar number of offspring, as compared to the general UK population (https://www.ons.gov.uk/). There is also a lower prevalence of self-reported COPD in UK Biobank (0.8%) compared to physician-diagnosed COPD in the general female UK population (1.68%).[42] Overall, women participating in UK Biobank reflect a higher-educated, more health-conscious group, with more modest underlying risk for COPD compared to the general population. We can only speculate as to how these selection factors might have influenced our associations of interest, but we propose that the most likely direction of any selection bias is towards the null (resulting in an underestimation). Strengths of this study include the large, prospective design. However, we relied on participants to self-report reproductive history which might have led to measurement error. Years of OC or HRT use were estimated as the difference between ages at first and last use. This likely resulted in overestimation of the duration of use, which might have attenuated our findings. In addition, we were unable to distinguish between reversible and non-reversible airway obstruction at baseline, as a bronchodilator was not administered before spirometry was conducted. Although we adjusted for self-reported asthma at baseline, this may have led to overestimation of COPD-related airway obstruction among participants with undiagnosed asthma. Lastly, we were unable to adjust for environmental tobacco smoke exposure as these data were not collected in UK Biobank. We therefore cannot exclude the possibility of residual bias from environmental tobacco smoke.

In conclusion, our findings indicate that several female reproductive health indicators across the life course are associated with risk of COPD hospitalisation/death and add to the indirect evidence

For peer review only - http://bmjopen.bmj.com/site/about/guidelines.xhtml

suggesting that that female reproductive hormones may have a role in COPD pathogenesis. Further studies are necessary to understand the opposing associations of hysterectomy, HRT and PCOS/ovarian cysts with COPD and objective measures of airway obstruction.

ACKNOWLEDGEMENTS

This research has been conducted using the UK Biobank Resource under Application Number 6326. We thank all the participants and researchers involved.

CONTRIBUTORS

All authors provided substantial contributions to the design of the work. RT analysed the data and drafted the manuscript. MCM and AF supervised the study, provided further data interpretation and revised the manuscript. All authors approved the final version of the manuscript and agree to be accountable for all aspects of the work in ensuring that questions related to the accuracy or integrity of any part of the work are appropriately investigated and resolved.

DATA SHARING:

Data were obtained from the UK Biobank Resource via its Access Management System (http://www.ukbiobank.ac.uk/wp-content/uploads/2012/09/Access-Procedures-2011-1.pdf).

Funding: This work was supported by the UK Medical Research Council grant MC/UU/12013/5, which provides infrastructure funding to the MRC Integrative Epidemiology Unit at the University of Bristol, and grant MR/M009351/1 as a fellowship to AF. This work was partly supported by the Research Council of Norway's Centres of Excellence scheme grant 262700.

Competing interests: None declared

For peer review only - http://bmjopen.bmj.com/site/about/guidelines.xhtml

For peer review only

REFERENCES

1. Townsend EA, Miller VM, Prakash YS. Sex differences and sex steroids in lung health and disease. *Endocr Rev* 2012;33(1):1-47.

2. Raghavan D, Jain R. Increasing awareness of sex differences in airway diseases. *Respirology* 2016;21(3):449-59.

3. Raghavan D, Varkey A, Bartter T. Chronic obstructive pulmonary disease: the impact of gender. *Curr Opin Pulm Med* 2017;23(2):117-23.

4. Gonzalez-Arenas A, Agramonte-Hevia J. Sex steroid hormone effects in normal and pathologic conditions in lung physiology. *Mini Rev Med Chem* 2012;12(11):1055-62.

5. Kamil F, Pinzon I, Foreman MG. Sex and race factors in early-onset COPD. *Curr Opin Pulm Med* 2013;19(2):140-4.

6. Sathish V, Martin YN, Prakash YS. Sex steroid signaling: implications for lung diseases. *Pharmacol Ther* 2015;150:94-108.

7. Fuentes N, Silveyra P. Endocrine regulation of lung disease and inflammation. *Exp Biol Med (Maywood)* 2018;243(17-18):1313-22.

8. Barr RG, Wentowski CC, Grodstein F, et al. Prospective study of postmenopausal hormone use and newly diagnosed asthma and chronic obstructive pulmonary disease. *Arch Intern Med* 2004;164(4):379-86.

9. Gaudet MM, Carter BD, Hildebrand JS, et al. Associations of parity and age at first pregnancy with overall and cause-specific mortality in the Cancer Prevention Study II. *Fertil Steril* 2017;107(1):179-88.e6.

10. Macsali F, Real FG, Plana E, et al. Early age at menarche, lung function, and adult asthma. *Am J Respir Crit Care Med* 2011;183(1):8-14.

11. Gill D, Sheehan NA, Wielscher M, et al. Age at menarche and lung function: a Mendelian randomization study. *Eur J Epidemiol* 2017;32(8):701-10.

For peer review only

12. Murri V, Antoniazzi F, Piazza M, et al. Lung Function in Women with Idiopathic Central Precocious Puberty: A Pilot Study. *Horm Res Paediatr* 2017;87(2):95-102.

13. Harik-Khan R, Wise RA, Lou C, et al. The effect of gestational parity on FEV1 in a group of healthy volunteer women. *Respir Med* 1999;93(6):382-8.

14. Carlson CL, Cushman M, Enright PL, et al. Hormone replacement therapy is associated with higher FEV1 in elderly women. *Am J Respir Crit Care Med* 2001;163(2):423-8.

15. Hayatbakhsh MR, Najman JM, O'Callaghan MJ, et al. Association between smoking and respiratory function before and after menopause. *Lung* 2011;189(1):65-71.

16. Amaral AF, Strachan DP, Gomez Real F, et al. Lower lung function associates with cessation of menstruation: UK Biobank data. *Eur Respir J* 2016;48(5):1288-97.

17. Triebner K, Matulonga B, Johannessen A, et al. Menopause Is Associated with Accelerated Lung Function Decline. *Am J Respir Crit Care Med* 2017;195(8):1058-65.

18. Campbell B, Davis SR, Abramson MJ, et al. Menopause, lung function and obstructive lung disease outcomes: a systematic review. *Climacteric* 2018;21(1):3-12.

19. Memoalia J, Anjum B, Singh N, et al. Decline in Pulmonary Function Tests after Menopause. *J Menopausal Med* 2018;24(1):34-40.

20. Cevrioglu AS, Fidan F, Unlu M, et al. The effects of hormone therapy on pulmonary function tests in postmenopausal women. *Maturitas* 2004;49(3):221-7.

21. Pata O, Atis S, Utku Oz A, et al. The effects of hormone replacement therapy type on pulmonary functions in postmenopausal women. *Maturitas* 2003;46(3):213-8.

22. Allen N, Sudlow C, Downey P, et al. UK Biobank: Current status and what it means for epidemiology. *Health Policy Technol* 2012;1(3):123-26.

23. Quanjer PH, Stanojevic S, Cole TJ, et al. Multi-ethnic reference values for spirometry for the 3-95-yr age range: the global lung function 2012 equations. *Eur Respir J* 2012;40(6):1324–1343.

24. Schoenfeld D. The asymptotic properties of nonparametric tests for comparing survival distributions. *Biometrika* 1981;68(1):316–319.

For peer review only - http://bmjopen.bmj.com/site/about/guidelines.xhtml

25. Global Initiative for Chronic Obstructive Lung Disease (GOLD). Global strategy for the diagnosis, management, and prevention of chronic obstructive pulmonary disease (2019 report). GOLD 2019.

26. Marshall A, Altman DG, Holder RL, et al. Combining estimates of interest in prognostic modelling studies after multiple imputation: current practice and guidelines. *BMC Med Res Methodol* 2009;9:57.

27. Rossi A, Butorac-Petanjek B, Chilosi M, et al. Chronic obstructive pulmonary disease with mild airflow limitation: current knowledge and proposal for future research - a consensus document from six scientific societies. *Int J Chron Obstruct Pulmon Dis* 2017;12:2593-610.

28. Casanova C, Aguirre-Jaime A, de Torres JP, et al. Longitudinal assessment in COPD patients: multidimensional variability and outcomes. *Eur Respir J* 2014;43(3):745-53.

29. Cushman M, Legault C, Barrett-Connor E, et al. Effect of postmenopausal hormones on inflammation-sensitive proteins: the Postmenopausal Estrogen/Progestin Interventions (PEPI) Study. *Circulation* 1999;100(7):717-22.

30. Walsh BW, Paul S, Wild RA, et al. The effects of hormone replacement therapy and raloxifene on C-reactive protein and homocysteine in healthy postmenopausal women: a randomized, controlled trial. *J Clin Endocrinol Metab* 2000;85(1):214-8.

31. Gol M, Akan P, Dogan E, et al. Effects of estrogen, raloxifene, and hormone replacement therapy on serum C-reactive protein and homocysteine levels. *Maturitas* 2006;53(3):252-9.

32. Kirsch EA, Yuhanna IS, Chen Z, et al. Estrogen acutely stimulates endothelial nitric oxide synthase in H441 human airway epithelial cells. *Am J Respir Cell Mol Biol* 1999;20(4):658-66.

33. Degano B, Mourlanette P, Valmary S, et al. Differential effects of low and high-dose estradiol on airway reactivity in ovariectomized rats. *Respir Physiol Neurobiol* 2003;138(2-3):265-74.

34. Townsend EA, Thompson MA, Pabelick CM, et al. Rapid effects of estrogen on intracellular Ca2+ regulation in human airway smooth muscle. *Am J Physiol Lung Cell Mol Physiol* 2010;298(4):L521-30.

For peer review only - http://bmjopen.bmj.com/site/about/guidelines.xhtml

35. Ynuk B. Endocrine regulation of airway contractility is overlooked. *J Endocrinol* 2014;222(2):R61-R73.

36. Stamatiou R, Paraskeva E, Papagianni M, et al. The mitogenic effect of testosterone and 17β-estradiol on airway smooth muscle cells. *Steroids* 2011;76(4):400-08.

37. Ambhore NS, Katragadda R, Raju Kalidhindi RS, et al. Estrogen receptor beta signaling inhibits PDGF induced human airway smooth muscle proliferation. *Mol Cell Endocrinol* 2018;476:37-47.

38. Yan F, Gao H, Zhao H, et al. Roles of airway smooth muscle dysfunction in chronic obstructive pulmonary disease. *J Transl Med* 2018;16(1):262.

39. Straub RH. The Complex Role of Estrogens in Inflammation. *Endocr Rev* 2007;28(5):521-74.

40. Glassberg MK, Catanuto P, Shahzeidi S, et al. Estrogen deficiency promotes cigarette smoke-induced changes in the extracellular matrix in the lungs of aging female mice. *Transl Res* 2016;178:107-17.

41. Tam A, Bates JH, Churg A, et al. Sex-Related Differences in Pulmonary Function following 6 Months of Cigarette Exposure: Implications for Sexual Dimorphism in Mild COPD. *PLoS One* 2016;11(10):e0164835.

42. Simpson CR, Hippisley-Cox J, Sheikh A. Trends in the epidemiology of chronic obstructive pulmonary disease in England: a national study of 51 804 patients. *Br J Gen Pract* 2010;60(576):277-84.

For peer review only

For peer review only - http://bmjopen.bmj.com/site/about/guidelines.xhtml

FIGURE LEGEND

Figure 1. Flow Chart of Study Population.

COPD denotes chronic obstructive pulmonary disease. Missing data is in one or more of the following variables: height, ethnicity, educational attainment, income, Townsend deprivation index, smoking history, spirometry measurements, maternal COPD history, paternal COPD history, age at menarche, menopause status, age at natural menopause, parity, oral contraception use, hormone replacement therapy use or gynaecological surgery history.

For peer review only

For peer review only - http://bmjopen.bmj.com/site/about/guidelines.xhtml

TABLES

Table 1.

Distribution of observed and imputed data in UK Biobank women at baseline (N=273 441)

Characteristic	% data imputed	Mean (SE) or % in observed data	Mean (SE) or % in imputed data
Age (years)	0	56.35 (0.02)	56.35 (0.02)
<55		39.5	39.5
55-59		18.6	18.6
60-64		24.2	24.2
>64		17.8	17.8
Height (cm)	0.4	162.4 (0.01)	162.4 (0.01)
Body mass index (kg/m^2)	0		
<18.5		0.8	0.8
18.5-24.9		38.7	38.7
25.0-29.9		36.5	36.5
>29.9		24.1	24.1
Non-white ethnicity	0.5	5.4	5.4
Education	1.2		
College, university or other professional degree		36.9	37.3
A levels, AS levels or equivalent		11.8	11.9
O levels, GCSEs or equivalent		23.3	23.5
CSE or equivalent		5.4	5.4
NVQ, HND, HNC or equivalent		4.5	4.6
Other		17.0	17.3

For peer review only - http://bmjopen.bmj.com/site/about/guidelines.xhtml

Annual household income	0.4		
<£18,000		20.4	20.6
£18,000-£30,999		21.7	21.8
£31,000-£51,999		20.9	21.0
£52,000-£100,000		15.3	15.4
>£100,000		4.0	4.0
Do not know/prefer not to answer		17.3	17.4
Townsend deprivation index	0.1	-1.33 (0.01)	-1.33 (0.01)
Smoking history (pack-years)	14.3		
None		59.3	69.1
Up to 10		7.9	9.2
11-20		7.4	8.7
21-30		5.2	6.1
>30		5.9	6.9
Prevalent (baseline) COPD	0	0.8	0.8
Incident COPD hospitalisation/death	0	0.4	0.4
Spirometry[a]	28.4		
FEV_1 z-score		0.00001 (0.002)	-0.0008 (0.002)
FVC z-score		-0.000002 (0.002)	-0.001 (0.002)
FEV_1/FVC z-score		0.00003 (0.002)	0.002 (0.002)
Asthma	0	12.9	12.9
Maternal history of COPD	1.9		
No		90.0	91.7
Yes		6.0	6.1
Don't know		2.2	2.2

For peer review only - http://bmjopen.bmj.com/site/about/guidelines.xhtml

Paternal history of COPD	3.0		
No		81.7	84.2
Yes		10.3	10.6
Don't know		5.0	5.2
Age at menarche, years	3.3		
<12		19.4	20.0
12-15		71.7	74.1
>15		5.7	5.9
Menopause status	16.1		
No		23.4	26.4
Yes		60.5	73.6
Age at natural menopause, years	20.8		
<47		11.4	20.7
47-49		8.9	12.8
50-52		19.7	23.7
>52		15.8	16.3
Did not undergo natural menopause		23.4	26.4
Parity	1.9		
Nulliparous		14.7	15.0
Parous		83.4	85.0
1		11.9	12.2
2		33.7	34.4
3		24.8	25.3
>3		12.9	13.2
PCOS/ovarian cysts	0	1.7	1.7

For peer review only - http://bmjopen.bmj.com/site/about/guidelines.xhtml
For peer review only

Endometriosis	0	1.5	1.5
Oral contraception use, years	0.5		
Never		18.9	19.0
Ever		80.6	81.0
1		11.0	11.1
2-4		18.6	18.7
5-9		17.7	17.7
10-15		17.4	17.4
>15		16.0	16.0
Hormone replacement therapy, years	5.7		
Never		61.4	63.7
Ever		32.9	36.3
1-2		10.7	11.8
3-5		8.4	9.1
6-10		5.7	6.2
>10		8.2	9.2
Gynaecological surgery	1.8		
No		80.4	81.7
Hysterectomy		9.9	10.1
Bilateral oophorectomy		0.4	0.4
Both		7.6	7.8

SE, standard error; A levels, Advanced levels; AS levels, Advanced Subsidiary levels; O levels, Ordinary levels; GCSE, General Certificate of Secondary Educations; CSE, certificate of secondary education; NVQ, national vocational qualifications; HND, higher national diploma; HNC, higher national certificate; COPD, chronic obstructive pulmonary disease; PCOS, polycystic ovary syndrome

[a]Spirometry measures were standardised by age, sex and height

For peer review only - http://bmjopen.bmj.com/site/about/guidelines.xhtml

For peer review only

Table 2.

Cox regression analyses of female reproductive health indicators with incident COPD-related hospitalisation/death during follow-up in women without COPD at baseline (N=271 271)

Reproductive health indicator	% of participants	Person-years of follow-up	Number of COPD events	Age-adjusted		Multiple-adjusted[a]	
				Hazard ratio	95% CI	Hazard ratio	95% CI
Age at menarche							
<12 years	20.0	326 027	275	1.31	1.14, 1.51	1.15	1.00, 1.32
12-15 years	74.1	1 210 146	768	1.0	NA	1.0	NA
>15 years	5.9	95 771	95	1.61	1.29, 1.99	1.37	1.11, 1.71
Menopause status							
No	26.6	437 890	97	1.0	NA	1.0	NA
Yes	73.4	1 194 055	1041	1.47	1.12, 1.94	1.07	0.82, 1.41
Age at natural menopause[b]							
<47 years	20.3	202 811	270	2.21	1.83, 2.68	1.44	1.19, 1.75
47-49 years	15.8	158 718	140	1.55	1.24, 1.94	1.25	1.00, 1.57

For peer review only - http://bmjopen.bmj.com/site/about/guidelines.xhtml

50-52 years	35.4	354 774	219	1.0	NA	1.0	NA
>52 years	28.4	283 721	161	0.86	0.69, 1.06	0.93	0.75, 1.15
Parity, binary							
Nulliparous	15.1	244 938	116	0.77	0.63, 0.93	0.92	0.76, 1.12
Parous	84.9	1 387 009	1022	1.0	NA	1.0	NA
Parity, ordered categories							
0	15.1	244 938	116	1.0	NA	1.0	NA
1	12.2	198 124	139	1.41	1.10, 1.81	1.06	0.82, 1.35
2	34.4	562 339	308	0.96	0.78, 1.19	0.92	0.74, 1.14
3	25.3	413 347	293	1.24	1.00, 1.54	1.06	0.85, 1.33
>3	13.1	213 195	282	2.21	1.78, 2.75	1.45	1.16, 1.82
PCOS/ovarian cysts							
No	98.3	1 604 641	1108	1.0	NA	1.0	NA
Yes	1.7	27 304	30	1.61	1.12, 2.32	1.61	1.12, 2.32
Endometriosis							
No	98.5	1 607 817	1127	1.0	NA	1.0	NA

For peer review only - http://bmjopen.bmj.com/site/about/guidelines.xhtml

Yes	1.5	24 131	11	0.79	0.44, 1.43	0.85	0.47, 1.53
Oral contraception							
Never used	18.9	308 641	295	1.0	NA	1.0	NA
Ever used	81.1	1 323 303	843	0.95	0.83, 1.09	0.85	0.74, 0.97
Years of oral contraception use							
0	18.9	308 641	295	1.0	NA	1.0	NA
1	11.1	180 195	137	1.05	0.85, 1.29	0.84	0.69, 1.04
2-4	18.7	306 053	246	1.05	0.88, 1.24	0.95	0.80, 1.13
5-9	17.8	290 554	181	0.92	0.76, 1.11	0.86	0.71, 1.05
10-15	17.5	285 766	153	0.84	0.69, 1.03	0.74	0.61, 0.91
>15	16.1	260 734	126	0.83	0.67, 1.04	0.75	0.60, 0.93
HRT							
Never used	63.9	1 042 083	510	1.0	NA	1.0	NA
Ever used	36.1	589 861	628	1.49	1.31, 1.69	1.15	1.01, 1.30
Years of HRT							
0	63.9	1 042 083	510	1.0	NA	1.0	NA

For peer review only

For peer review only - http://bmjopen.bmj.com/site/about/guidelines.xhtml

1-2	11.7	190 902	240	1.83	1.54, 2.16	1.33	1.12, 1.57
3-5	9.1	147 962	162	1.63	1.34, 1.97	1.17	0.97, 1.42
6-10	6.2	100 921	74	1.00	0.77, 1.31	0.87	0.66, 1.14
>10	9.2	150 077	152	1.27	1.04, 1.55	1.06	0.87, 1.29
Gynaecological surgery							
No	81.8	1 335 512	765	1.0	NA	1.0	NA
Hysterectomy	10.1	164 869	213	1.77	1.51, 2.07	1.49	1.28, 1.74
Bilateral oophorectomy	0.4	5957	6	1.56	0.70, 3.48	1.36	0.61, 3.04
Both	7.8	125 607	154	1.68	1.41, 2.00	1.42	1.19, 1.69

COPD, chronic obstructive pulmonary disease; CI, confidence interval; PCOS, polycystic ovary syndrome; HRT, hormone replacement

[a]Multiple-adjusted for age, height, BMI (log-transformed), ethnicity, education, household income, Townsend deprivation index, smoking history, maternal COPD and paternal COPD

[b]Age at menopause was analysed only among women who experienced natural menopause before baseline (n=159 571)

For peer review only - http://bmjopen.bmj.com/site/about/guidelines.xhtml

Table 3.

Linear regression analyses of female reproductive health indicators with baseline spirometry measures (N=273 441)

Reproductive health indicator	Mean change in FEV_1 z-score (95% CI)		Mean change in FVC z-score (95% CI)		Mean change in FEV_1/FVC z-score (95% CI)	
	Unadjusted	Multiple-adjusted[a]	Unadjusted	Multiple-adjusted[a]	Unadjusted	Multiple-adjusted[a]
Age at menarche						
<12 years	-0.04 (-0.05, -0.03)	-0.01 (-0.02, 0.00)	-0.05 (-0.06, -0.04)	-0.01 (-0.02, -0.01)	0.02 (0.01, 0.03)	-0.02 (-0.03, -0.01)
12-15 years	0	0	0	0	0	0
>15 years	-0.08 (-0.10, -0.06)	-0.03 (-0.05, -0.01)	-0.06 (-0.08, -0.04)	-0.03 (-0.05, -0.02)	-0.05 (-0.07, -0.02)	0.00 (-0.02, 0.02)
Menopause status						
No	0	0	0	0	0	0
Yes	-0.03 (-0.04, -0.02)	-0.04 (-0.05, -0.02)	-0.03 (-0.04, -0.02)	-0.04 (-0.06, -0.03)	0.00 (-0.01, 0.01)	0.01 (-0.01, 0.02)
Age at natural menopause[b]						
<47 years	-0.11 (-0.12, -0.09)	-0.04 (-0.05, -0.02)	-0.09 (-0.11, -0.08)	-0.04 (-0.05, -0.02)	-0.05 (-0.07, -0.04)	-0.01 (-0.02, 0.01)
47-49 years	-0.05 (-0.07, -0.04)	-0.02 (-0.04, 0.00)	-0.04 (-0.06, -0.03)	-0.02 (-0.03, 0.00)	-0.03 (-0.05, -0.02)	-0.01 (-0.03, 0.01)
50-52 years	0	0	0	0	0	0

For peer review only - http://bmjopen.bmj.com/site/about/guidelines.xhtml

>52 years	0.01 (0.00, 0.03)	0.00 (-0.01, 0.02)	0.00 (-0.02, 0.01)	0.00 (-0.02, 0.01)	0.03 (0.02, 0.05)	0.01 (0.00, 0.02)
Parity, binary						
Nulliparous	-0.02 (-0.03, -0.01)	-0.04 (-0.06, -0.03)	-0.04 (-0.05, -0.03)	-0.07 (-0.08, -0.05)	0.04 (0.03, 0.05)	0.04 (0.03, 0.05)
Parous	0	0	0	0	0	0
Parity, ordered categories						
0	0	0	0	0	0	0
1	-0.02 (-0.04, -0.01)	0.01 (0.00, 0.03)	-0.01 (-0.02, 0.01)	0.02 (0.01, 0.04)	-0.04 (-0.06, -0.03)	-0.02 (-0.04, 0.00)
2	0.04 (0.03, 0.06)	0.04 (0.03, 0.06)	0.06 (0.05, 0.08)	0.07 (0.06, 0.08)	-0.03 (-0.05, -0.02)	-0.04 (-0.06, -0.03)
3	0.03 (0.02, 0.05)	0.05 (0.04, 0.07)	0.05 (0.04, 0.07)	0.08 (0.06, 0.09)	-0.04 (-0.06, -0.03)	-0.04 (-0.06, -0.03)
>3	-0.03 (-0.05, -0.01)	0.05 (0.04, 0.07)	-0.01 (-0.02, 0.01)	0.08 (0.07, 0.10)	-0.07 (-0.08, -0.05)	-0.06 (-0.07, -0.04)
PCOS/ovarian cysts						
No	0	0	0	0	0	0
Yes	-0.02 (-0.05, 0.02)	-0.01 (-0.04, 0.03)	-0.05 (-0.08, -0.01)	-0.03 (-0.07, 0.00)	0.06 (0.03, 0.09)	0.05 (0.02, 0.09)
Endometriosis						
No	0	0	0	0	0	0
Yes	0.01 (-0.03, 0.04)	0.01 (-0.02, 0.05)	-0.02 (-0.06, 0.01)	-0.02 (-0.05, 0.01)	0.06 (0.02, 0.10)	0.07 (0.03, 0.10)

For peer review only - http://bmjopen.bmj.com/site/about/guidelines.xhtml

Oral contraception						
Never used	0	0	0	0	0	0
Ever used	0.07 (0.05, 0.08)	0.03 (0.01, 0.04)	0.08 (0.07, 0.09)	0.02 (0.01, 0.03)	-0.01 (-0.03, 0.00)	0.01 (0.003, 0.03)
Years of oral contraception						
0	0	0	0	0	0	0
1	0.03 (0.01, 0.04)	0.01 (-0.01, 0.03)	0.04 (0.02, 0.06)	0.01 (-0.01, 0.03)	-0.03 (-0.04, -0.01)	0.01 (-0.004, 0.03)
2-4	0.05 (0.04, 0.07)	0.02 (0.01, 0.04)	0.06 (0.05, 0.08)	0.02 (0.01, 0.03)	0.00 (-0.02, 0.01)	0.02 (0.003, 0.03)
5-9	0.08 (0.07, 0.10)	0.03 (0.02, 0.05)	0.09 (0.08, 0.10)	0.03 (0.01, 0.04)	0.00 (-0.02, 0.01)	0.02 (0.003, 0.03)
10-15	0.08 (0.07, 0.10)	0.04 (0.02, 0.05)	0.10 (0.08, 0.11)	0.04 (0.02, 0.05)	-0.02 (-0.03, 0.00)	0.01 (-0.002, 0.03)
>15	0.07 (0.05, 0.08)	0.02 (0.01, 0.04)	0.08 (0.07, 0.10)	0.02 (0.00, 0.03)	-0.03 (-0.04, -0.01)	0.01 (-0.002, 0.03)
HRT						
Never used	0	0	0	0	0	0
Ever used	-0.02 (-0.02, -0.01)	0.00 (0.00, 0.01)	-0.01 (-0.01, 0.00)	0.00 (-0.01, 0.01)	-0.02 (-0.03, -0.01)	0.02 (0.01, 0.03)
Years of HRT						
0	0	0	0	0	0	0
1-2	-0.03 (-0.04, -0.01)	0.00 (-0.01, 0.02)	-0.01 (-0.03, 0.00)	0.00 (-0.02, 0.01)	-0.03 (-0.05, -0.02)	0.02 (0.01, 0.04)

For peer review only - http://bmjopen.bmj.com/site/about/guidelines.xhtml

For peer review only

3-5	-0.03 (-0.04, -0.01)	0.00 (-0.01, 0.02)	-0.02 (-0.03, 0.00)	0.00 (-0.02, 0.02)	-0.02 (-0.04, 0.00)	0.02 (0.00, 0.03)
6-10	0.00 (-0.02, 0.02)	0.01 (-0.01, 0.02)	0.00 (-0.02, 0.02)	0.00 (-0.02, 0.02)	0.00 (-0.02, 0.01)	0.02 (0.00, 0.04)
>10	0.00 (-0.02, 0.01)	0.00 (-0.01, 0.02)	0.01 (0.00, 0.03)	0.00 (-0.01, 0.02)	-0.03 (-0.05, -0.01)	0.01 (-0.01, 0.03)
Gynaecological surgery						
No	0	0	0	0	0	0
Hysterectomy	-0.03 (-0.05, -0.02)	0.01 (0.00, 0.03)	-0.05 (-0.06, -0.04)	0.00 (-0.02, 0.01)	0.03 (0.01, 0.04)	0.04 (0.02, 0.06)
Bilateral oophorectomy	-0.05 (-0.12, 0.02)	-0.02 (-0.08, 0.05)	-0.07 (-0.14, 0.00)	-0.04 (-0.11, 0.03)	0.04 (-0.03, 0.12)	0.05 (-0.02, 0.12)
Both	-0.04 (-0.05, -0.02)	0.00 (-0.01, 0.02)	-0.07 (-0.08, -0.05)	-0.02 (-0.04, -0.01)	0.05 (0.04, 0.07)	0.05 (0.04, 0.07)

FEV_1, forced expiratory volume in one-second; FVC, forced vital capacity; CI, confidence interval; PCOS, polycystic ovary syndrome; HRT, hormone replacement therapy

[a]Adjusted for age, BMI (log-transformed), ethnicity, education, household income, Townsend deprivation index, smoking history, asthma, maternal COPD and paternal COPD

[b]Age at menopause was analysed only among women who experienced natural menopause before baseline (n=161 069)

For peer review only - http://bmjopen.bmj.com/site/about/guidelines.xhtml

For peer review only

Table 4.

Direction of association for female reproductive health indicators with risk of COPD-related hospitalisation/death and spirometry measures

Reproductive health indicator	COPD-related hospitalisation/death	FEV_1	FVC	FEV_1/FVC
Age at menarche				
<12 years	=	=	↓	↓
12-15 years	NA	NA	NA	NA
>15 years	↑	↓	↓	=
Menopause status				
No	NA	NA	NA	NA
Yes	=	↓	↓	=
Age at natural menopause				
<47 years	↑	↓	↓	=
47-49 years	=	=	=	=
50-52 years	NA	NA	NA	NA
>52 years	=	=	=	=
Parity, binary				
Nulliparous	=	↓	↓	↑
Parous	NA	NA	NA	NA
Parity, ordered categories				
0	NA	NA	NA	NA
1	=	=	↑	↓
2	=	↑	↑	↓
3	=	↑	↑	↓

For peer review only - http://bmjopen.bmj.com/site/about/guidelines.xhtml

For peer review only

>3	↑	↑	↑	↓
PCOS/ovarian cysts				
No	NA	NA	NA	NA
Yes	↑	=	=	↑
Endometriosis				
No	NA	NA	NA	NA
Yes	=	=	=	↑
Oral contraception				
Never used	NA	NA	NA	NA
Ever used	↓	↑	↑	=
Years of oral contraception				
0	NA	NA	NA	NA
1	=	=	=	=
2-4	=	↑	↑	↑
5-9	=	↑	↑	↑
10-15	↓	↑	↑	=
>15	↓	↑	=	=
Hormone replacement therapy				
Never used	NA	NA	NA	NA
Ever used	↑	=	=	↑
Years of hormone replacement				
0	NA	NA	NA	NA
1-2	↑	=	=	↑
3-5	=	=	=	=
6-10	=	=	=	=

For peer review only - http://bmjopen.bmj.com/site/about/guidelines.xhtml

For peer review only

>10	=	=	=	=
Gynaecological surgery				
No	NA	NA	NA	NA
Hysterectomy	↑	=	=	↑
Bilateral oophorectomy	=	=	=	=
Both	↑	=	↓	↑

COPD, chronic obstructive pulmonary disease; FEV_1, forced expiratory volume in one-second; FVC, forced vital capacity; PCOS, polycystic ovary syndrome; NA, not applicable where the category is the reference group

For peer review only

For peer review only - http://bmjopen.bmj.com/site/about/guidelines.xhtml

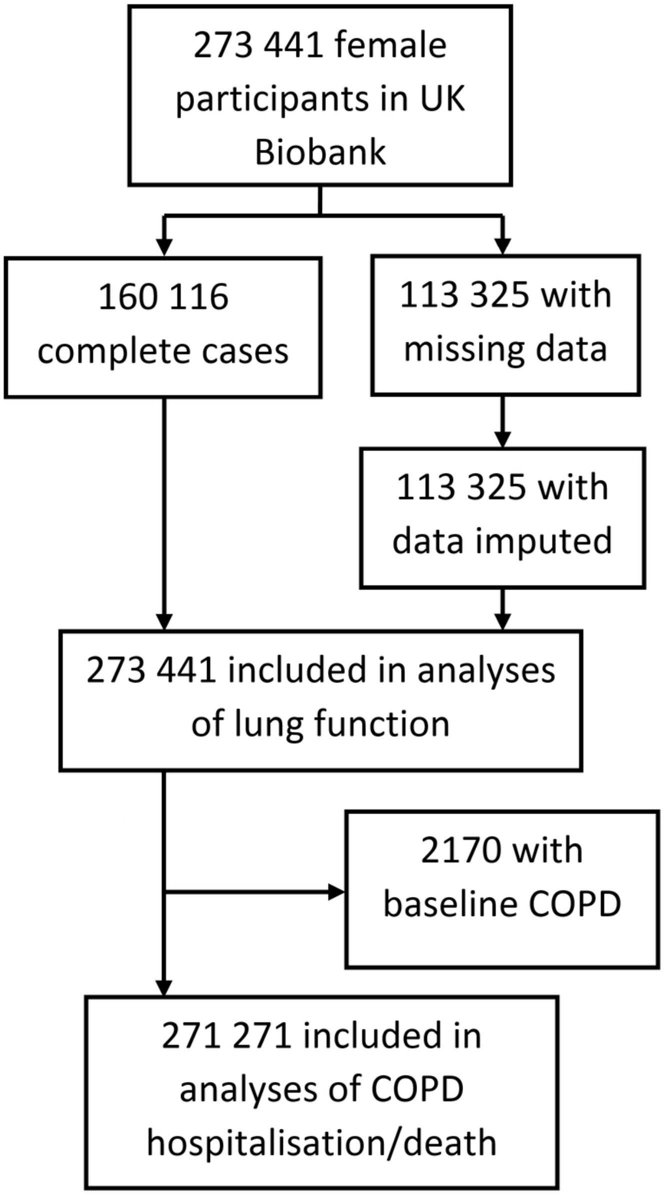

Figure 1. Flow Chart of Study Population.
COPD, chronic obstructive pulmonary disease. Missing data is in one or more of the following variables: height, ethnicity, educational attainment, income, Townsend deprivation index, smoking history, spirometry measurements, maternal COPD history, paternal COPD history, age at menarche, menopause status, age at natural menopause, parity, oral contraception use, hormone replacement therapy use or gynaecological surgery history.

80x117mm (300 x 300 DPI)

Online Supplementary File

Table S1. Variables used in multiple imputation model

Variable	Type of variable	Regression model to predict missing in this variable	How variable was entered when used to predict missing in other variables
Incident COPD hospitalisation	Dichotomous	Logistic regression	1 indicator variable
Incident COPD death	Dichotomous	Logistic regression	1 indicator variable
FEV_1 z-score	Continuous	Linear regression	Continuous
FVC z-score	Continuous	Linear regression	Continuous
FEV_1/FVC z-score	Continuous	Linear regression	Continuous
Age at menarche, years	Categorical (3)	Ordered logistic regression	2 indicator variables
Menopause status	Categorical (3)	Polytomous (unordered) regression	2 indicator variables
Age at menopause, years	Categorical (4)	Ordered logistic regression	3 indicator variables
Parity	Categorical (5)	Ordered logistic regression	4 indicator variables
History of ovarian cysts or polycystic ovary syndrome	Dichotomous	Logistic regression	Dichotomous
History of endometriosis	Dichotomous	Logistic regression	Dichotomous
Oral contraception ever use	Dichotomous	Passively imputed using years of OC use	N/A
Years of oral contraception	Categorical (6)	Ordered logistic regression	5 indicator variables
Gynaecological surgery	Categorical (4)	Polytomous (unordered) regression	3 indicator variables
HRT ever use	Dichotomous	Passively imputed using years of HRT use	N/A

For peer review only - http://bmjopen.bmj.com/site/about/guidelines.xhtml

For peer review only

Years of HRT use	Categorical (6)	Polytomous (unordered) regression	5 indicator variables
Age, years	Continuous	Linear regression	Continuous
Height, cm	Continuous	Linear regression	Continuous
Log body mass index	Continuous	Linear regression	Continuous
Ethnicity	Dichotomous	Logistic regression	Dichotomous
Qualifications	Categorical (6)	Polytomous (unordered) regression	6 indicator variables
Income	Categorical (6)	Polytomous (unordered) regression	5 indicator variables
Townsend deprivation index	Continuous	Linear regression	Continuous
Smoking pack-years	Categorical (5)	Ordered logistic regression	4 indicator variables
Smoking duration, years	Categorical (5)	Ordered logistic regression	4 indicator variables
Smoking intensity, cigarettes/day	Continuous	Linear regression	Continuous
Smoking status	Dichotomous	Logistic regression	Dichotomous
Maternal history of COPD	Dichotomous	Logistic regression	Dichotomous
Paternal history of COPD	Dichotomous	Logistic regression	Dichotomous
History of asthma	Dichotomous	Logistic regression	Dichotomous
History of pneumonia	Dichotomous	Logistic regression	Dichotomous
History of tuberculosis	Dichotomous	Logistic regression	Dichotomous
History of cardiovascular disease	Dichotomous	Logistic regression	Dichotomous
History of diabetes	Dichotomous	Logistic regression	Dichotomous

For peer review only - http://bmjopen.bmj.com/site/about/guidelines.xhtml

For peer review only

COPD, chronic obstructive pulmonary disease; FEV_1, forced expiratory volume in one-second; FVC, forced vital capacity; HRT, hormone replacement therapy

For peer review only

For peer review only - http://bmjopen.bmj.com/site/about/guidelines.xhtml

Table S2. Characteristics in UK Biobank women with and without spirometry data

Characteristic	Mean (SE) or % in women with spirometry	Mean (SE) or % in women without spirometry	*P* value
N	195 824	77 617	
Age (years)	56.18 (0.02)	56.78 (0.03)	<0.001
<55	20.9	20.3	
55-59	23.0	20.2	
60-64	30.5	29.7	
65 or higher	25.6	29.7	
Missing	0	0	
Height (cm)	162.6 (0.01)	162.0 (0.02)	<0.001
Missing	0.1	2.5	
Body mass index (kg/m^2)			<0.001
<18.5	0.6	1.2	
18.5-24.9	38.3	36.1	
25.0-29.9	37.5	35.7	
>29.9	23.4	25.3	
Missing	0.2	1.6	
Ethnicity			<0.001
White	95.0	92.2	
Other	4.7	7.1	
Missing	0.3	0.8	
Education			<0.001
College, university or other professional degree	37.9	34.4	
A levels, AS levels or equivalent	12.2	10.6	
O levels, GCSEs or equivalent	23.5	22.7	
CSE or equivalent	5.3	5.6	
NVQ, HND, HNC or equivalent	4.4	4.8	
Other	15.7	20.1	
Missing	1.0	1.8	
Annual household income (£)			<0.001
<18 000	19.2	23.4	

For peer review only - http://bmjopen.bmj.com/site/about/guidelines.xhtml

For peer review only

>18 000 and <31 000	21.7	21.6	
>31 000 and <52 000	21.7	19.0	
>52 000 and <100 000	16.3	12.8	
>100 000	4.3	3.0	
Do not know/prefer not to answer	16.4	19.5	
Missing	0.3	0.7	
Townsend deprivation index	-1.41 (0.01)	-1.14 (0.01)	<0.001
Missing	0.1	0.1	
Smoking history (pack-years)			<0.001
None	58.4	61.4	
<11	8.3	6.8	
11-20	7.7	6.7	
21-30	5.4	4.9	
>30	5.7	6.2	
Missing	14.5	14.0	
Incident COPD hospitalisation/death	0.3	0.6	<0.001
Missing	0.6	1.2	
Prevalent COPD at baseline	0.6	1.2	<0.001
Missing	<0.1	0	
Asthma			<0.001
No	86.9	87.6	
Yes	13.1	12.4	
Missing	<0.1	0	
Maternal history of COPD			<0.001
No	90.3	89.4	
Yes	6.0	5.8	
Don't know	2.0	2.4	
Missing	1.7	2.4	
Paternal history of COPD			<0.001
No	82.2	80.6	
Yes	10.2	10.3	
Don't know	4.8	5.5	
Missing	2.8	3.6	
Age at menarche			<0.001

For peer review only - http://bmjopen.bmj.com/site/about/guidelines.xhtml

For peer review only

<12	19.3	19.5	
12-15	72.2	70.3	
>15	5.5	6.2	
Missing	3.0	4.0	
Menopause status			<0.001
No	24.0	22.1	
Yes	60.3	61.0	
Missing	15.7	16.9	
Age at natural menopause			<0.001
<47	11.5	12.6	
47-49	9.4	9.4	
50-52	21.4	20.9	
>52	17.1	16.9	
Did not undergo natural menopause	35.6	34.6	
Missing	4.9	5.7	
Parity, binary			<0.001
Nulliparous	14.5	15.4	
Parous	83.8	82.3	
Parity, ordinal (5)			<0.001
0	14.5	15.4	
1	11.9	11.9	
2	34.1	32.7	
3	25.1	24.1	
>3	12.7	13.5	
Missing	1.7	2.4	
PCOS/ovarian cysts	1.7	1.7	0.60
Missing	0	0	
Endometriosis	1.5	1.5	0.81
Missing	0	0	
Oral contraception ever used	81.9	77.4	<0.001
Years of oral contraception use			<0.001
0	17.7	21.6	
1	10.8	11.6	

For peer review only

For peer review only - http://bmjopen.bmj.com/site/about/guidelines.xhtml

2-4	18.6	18.6	
5-9	18.1	16.5	
10-15	17.9	16.2	
>15	16.5	14.6	
Missing	0.3	1.0	
HRT ever used	37.8	38.7	<0.001
Years of HRT			<0.001
0	61.9	60.3	
1-2	10.5	11.1	
3-5	8.3	8.6	
6-10	5.7	5.5	
>10	8.2	8.2	
Missing	5.5	6.3	
Gynaecological surgery			<0.001
No	81.2	78.5	
Hysterectomy	9.6	10.5	
Bilateral oophorectomy	0.4	0.4	
Both	7.3	8.2	
Missing	1.5	2.4	

SE, standard error; A levels, Advanced levels; AS levels, Advanced Subsidiary levels; O levels, Ordinary levels; GCSE, General Certificate of Secondary Educations; CSE, certificate of secondary education; NVQ, national vocational qualifications; HND, higher national diploma; HNC, higher national certificate; COPD, chronic obstructive pulmonary disease; PCOS, polycystic ovary syndrome; HRT, hormone replacement therapy

For peer review only

For peer review only - http://bmjopen.bmj.com/site/about/guidelines.xhtml

Table S3. Cox regression analyses of female reproductive health indicators with incident COPD-related hospitalisation/death during follow-up in **women with FEV_1/FVC ≥ 0.7 and without a history of respiratory illness**[a] (N=217 512)

Reproductive health indicator	% of participants	Person-years of follow-up	Number of COPD events	Age-adjusted Hazard ratio	Age-adjusted 95% CI	Multiple-adjusted[b] Hazard ratio	Multiple-adjusted[b] 95% CI
Age at menarche							
<12 years	19.8	259 259	97	1.39	1.10, 1.76	1.27	1.00, 1.62
12-15 years	74.4	975 565	259	1.0	NA	1.0	NA
>15 years	5.8	75 410	36	1.84	1.29, 2.62	1.53	1.08, 2.19
Menopause status							
No	27.2	359 354	34	1.0	NA	1.0	NA
Yes	72.8	950 881	358	1.09	1.07, 1.12	0.88	0.56, 1.39
Age at natural menopause[c]							
<47 years	19.8	157 942	90	2.25	1.60, 3.15	1.59	1.14, 2.23
47-49 years	15.9	127 134	50	1.67	1.12, 2.47	1.40	0.94, 2.08
50-52 years	35.7	285 678	74	1.0	NA	1.0	NA
>52 years	28.6	228 061	57	0.89	0.61, 1.30	0.97	0.67, 1.42
Parity, binary							
Nulliparous	15.3	199 136	43	0.84	0.60, 1.15	0.93	0.67, 1.29
Parous	84.7	1 111 099	349	1.0	NA	1.0	NA
Parity, ordered categories							
0	15.3	199 136	43	1.0	NA	1.0	NA
1	12.2	159 386	49	1.37	0.91, 2.07	1.10	0.72, 1.66

For peer review only - http://bmjopen.bmj.com/site/about/guidelines.xhtml

2	34.7	455 068	115	0.95	0.67, 1.36	0.97	0.68, 1.39
3	25.2	330 200	98	1.12	0.77, 1.61	1.03	0.71, 1.49
>3	12.7	166 444	87	1.86	1.28, 2.70	1.34	0.92, 1.95
PCOS/ovarian cysts							
No	98.3	1 288 468	378	1.0	NA	1.0	NA
Yes	1.7	21 767	14	2.20	1.29, 3.76	2.23	1.31, 3.81
Endometriosis							
No	98.5	1 291 165	387	1.0	NA	1.0	NA
Yes	1.5	459 189	5	1.07	0.44, 2.58	1.09	0.45, 2.65
Oral contraception							
Never used	18.9	247 243	117	1.0	NA	1.0	NA
Ever used	81.1	1 062 992	275	0.81	0.65, 1.01	0.72	0.57, 0.91
Years of oral contraception							
0	18.9	247 243	117	1.0	NA	1.0	NA
1	10.9	143 083	41	0.83	0.58, 1.18	0.66	0.46, 0.94
2-4	18.6	244 583	86	0.94	0.71, 1.25	0.88	0.66, 1.17
5-9	17.8	234 184	57	0.75	0.54, 1.03	0.70	0.50, 0.97
10-15	17.6	230 642	51	0.74	0.53, 1.04	0.65	0.46, 0.92
>15	16.1	210 498	40	0.71	0.49, 1.03	0.62	0.43, 0.90
HRT							
Never used	65.0	851 046	181	1.0	NA	1.0	NA
Ever used	35.0	459 189	211	1.41	1.14, 1.74	1.11	0.90, 1.38

For peer review only

Years of HRT							
0	65.0	851 046	181	1.0	NA	1.0	NA
1-2	11.2	146 764	83	1.81	1.37, 2.38	1.33	1.01, 1.75
3-5	8.8	116 043	51	1.43	1.03, 1.98	1.07	0.77, 1.49
6-10	6.1	80 081	25	0.97	0.63, 1.50	0.85	0.55, 1.32
>10	8.8	116 300	52	1.21	0.87, 1.69	1.05	0.75, 1.46
Gynaecological surgery							
No	82.3	1 078 722	263	1.0	NA	1.0	NA
Hysterectomy	9.7	127 997	73	1.75	1.34, 2.29	1.54	1.18, 2.00
Bilateral oophorectomy	0.4	4749	4	2.86	1.06, 7.68	2.49	0.92, 6.70
Both	7.6	98 766	52	1.62	1.19, 2.19	1.39	1.03, 1.89

COPD, chronic obstructive pulmonary disease; FEV_1, forced expiratory volume in one-second; FVC, forced vital capacity; CI, confidence interval; PCOS, polycystic ovary syndrome; HRT, hormone replacement therapy; NA, not applicable

[a]Respiratory illnesses include COPD, asthma, tuberculosis or pneumonia reported at baseline

[b]Multiple-adjusted for age, height, BMI (log-transformed), ethnicity, education, household income, Townsend deprivation index, smoking history in pack-years, maternal COPD and paternal COPD

[c]Age at menopause was analysed only among women who experienced natural menopause before baseline (n=127 270)

For peer review only - http://bmjopen.bmj.com/site/about/guidelines.xhtml

For peer review only

Table S4. Cox regression analyses of female reproductive health indicators with incident COPD-related hospitalisation/death during follow-up, **further adjusting for smoking status or smoking duration and intensity** (N=271 271)

Reproductive health indicator	% of participants	Person-years of follow-up	Number of COPD events	Multiple-adjusted[a]		Smoking duration and intensity-adjusted[b]		Smoking status-adjusted[c]	
				Hazard ratio	95% CI	Hazard ratio	95% CI	Hazard ratio	95% CI
Age at menarche									
<12 years	20.0	326 027	275	1.15	1.00, 1.32	1.18	1.03, 1.36	1.17	1.01, 1.34
12-15 years	74.1	1 210 146	768	1.0	NA	1.0	NA	1.0	NA
>15 years	5.9	95 771	95	1.37	1.11, 1.71	1.33	1.07, 1.65	1.32	1.06, 1.64
Menopause status									
No	26.6	437 890	97	1.0	NA	1.0	NA	1.0	NA
Yes	73.4	1 194 055	1041	1.07	0.82, 1.41	1.06	0.81, 1.39	1.03	0.78, 1.36
Age at natural menopause[d]				1.44	1.19, 1.75	1.47	1.21, 1.79	1.43	1.18, 1.74
<47 years	20.3	202 811	270	1.25	1.00, 1.57	1.25	1.00, 1.57	1.24	0.99, 1.56
47-49 years	15.8	158 718	140	1.0	NA	1.0	NA	1.0	NA
50-52 years	35.4	354 774	219	0.93	0.75, 1.15	0.91	0.74, 1.13	0.94	0.76, 1.16
>52 years	28.4	283 721	161						
Parity, binary									
Nulliparous	15.1	244 938	116	0.92	0.76, 1.12	0.90	0.74, 1.09	0.92	0.75, 1.12
Parous	84.9	1 387 009	1022	1.0	NA	1.0	NA	1.0	NA

For peer review only

For peer review only - http://bmjopen.bmj.com/site/about/guidelines.xhtml

Parity, ordered categories										
0	15.1	244 938	116	1.0	NA	1.0	NA	1.0	NA	
1	12.2	198 124	139	1.06	0.82, 1.35	1.10	0.86, 1.41	1.06	0.82, 1.36	
2	34.4	562 339	308	0.92	0.74, 1.14	0.93	0.75, 1.16	0.93	0.75, 1.15	
3	25.3	413 347	293	1.06	0.85, 1.33	1.10	0.88, 1.37	1.07	0.86, 1.34	
>3	13.1	213 195	282	1.45	1.16, 1.82	1.50	1.20, 1.88	1.43	1.15, 1.79	
PCOS/ovarian cysts										
No	98.3	1 604 641	1108	1.0	NA	1.0	NA	1.0	NA	
Yes	1.7	27 304	30	1.61	1.12, 2.32	1.66	1.16, 2.39	1.65	1.14, 2.37	
Endometriosis										
No	98.5	1 607 817	1127	1.0	NA	1.0	NA	1.0	NA	
Yes	1.5	24 131	11	0.85	0.47, 1.53	0.84	0.46, 1.52	0.84	0.46, 1.53	
Oral contraception										
Never used	18.9	308 641	295	1.0	NA	1.0	NA	1.0	NA	
Ever used	81.1	1 323 303	843	0.85	0.74, 0.97	0.90	0.78, 1.03	0.87	0.75, 1.00	
Years of oral contraception use	18.9	308 641	295	1.0	NA	1.0	NA	1.0	NA	
0	11.1	180 195	137	0.84	0.69, 1.04	0.89	0.72, 1.09	0.86	0.70, 1.06	
1	18.7	306 053	246	0.95	0.80, 1.13	1.00	0.84, 1.19	0.98	0.82, 1.16	
2-4	17.8	290 554	181	0.86	0.71, 1.05	0.92	0.76, 1.11	0.87	0.72, 1.06	
5-9	17.5	285 766	153	0.74	0.61, 0.91	0.80	0.66, 0.98	0.77	0.63, 0.94	
10-15	16.1	260 734	126	0.75	0.60, 0.93	0.80	0.64, 1.00	0.77	0.62, 0.96	

For peer review only - http://bmjopen.bmj.com/site/about/guidelines.xhtml

For peer review only

>15									
HRT									
Never used	63.9	1 042 083	510	1.0	NA	1.0	NA	1.0	NA
Ever used	36.1	589 861	628	1.15	1.01, 1.30	1.20	1.06, 1.36	1.16	1.03, 1.32
Years of HRT									
0	63.9	1 042 083	510	1.0	NA	1.0	NA	1.0	NA
1-2	11.7	190 902	240	1.33	1.12, 1.57	1.39	1.17, 1.65	1.36	1.14, 1.61
3-5	9.1	147 962	162	1.17	0.97, 1.42	1.23	1.01, 1.49	1.17	0.96, 1.42
6-10	6.2	100 921	74	0.87	0.66, 1.14	0.90	0.69, 1.18	0.88	0.67, 1.15
>10	9.2	150 077	152	1.06	0.87, 1.29	1.12	0.92, 1.36	1.07	0.88, 1.31
Gynaecological surgery									
No	81.8	1 335 512	765	1.0	NA	1.0	NA	1.0	NA
Hysterectomy	10.1	164 869	213	1.49	1.28, 1.74	1.49	1.27, 1.74	1.47	1.26, 1.72
Bilateral oophorectomy	0.4	5957	6	1.36	0.61, 3.04	1.34	0.60, 3.00	1.38	0.62, 3.08
Both	7.8	125 607	154	1.42	1.19, 1.69	1.41	1.18, 1.68	1.39	1.17, 1.66

COPD, chronic obstructive pulmonary disease; CI, confidence interval; PCOS, polycystic ovary syndrome; HRT, hormone replacement

[a]Multiple-adjusted for age, height, BMI (log-transformed), ethnicity, education, household income, Townsend deprivation index, smoking history in pack years, maternal COPD and paternal COPD

[b]Multiple-adjusted with smoking history as two covariates (duration in years and intensity in average number of cigarettes/day) rather than smoking pack-years

For peer review only - http://bmjopen.bmj.com/site/about/guidelines.xhtml

For peer review only

[c]Further adjusted from the multiple-adjusted analyses[a] for smoking status at baseline

[d]Age at menopause was analysed only among women who experienced natural menopause before baseline (n=159 571)

For peer review only

Table S5. Cox regression analyses of female reproductive health indicators with incident COPD-related hospitalisation/death during follow-up in **never smokers** (N=161 626)

Reproductive health indicator	% of participants	Person-years of follow-up	Number of COPD events	Age-adjusted		Multiple-adjusted[a]	
				Hazard ratio	95% CI	Hazard ratio	95% CI
Age at menarche							
<12 years	19.9	193 712	59	1.63	1.20, 2.22	1.39	1.02, 1.90
12-15 years	74.4	725 115	134	1.0	NA	1.0	NA
>15 years	5.7	55 624	18	1.82	1.11, 2.98	1.76	1.07, 2.89
Menopause status							
No	30.4	252 038	40	1.0	NA	1.0	NA
Yes	69.6	571 767	181	0.99	0.55, 1.77	0.98	0.55, 1.76
Age at natural menopause[b]							
<47 years	18.7	108 538	37	1.66	1.05, 2.64	1.53	0.96, 2.43
47-49 years	15.5	90 293	18	1.05	0.58, 1.91	1.03	0.57, 1.86
50-52 years	36.1	210 322	44	1.0	NA	1.0	NA
>52 years	29.7	172 378	33	0.85	0.54, 1.35	0.83	0.52, 1.32
Parity, binary							
Nulliparous	16.3	157 900	27	0.92	0.61, 1.38	0.94	0.62, 1.42
Parous	83.7	816 551	184	1.0	NA	1.0	NA
Parity, ordered categories							
0	16.3	157 900	27	1.0	NA	1.0	NA
1	11.2	109 141	24	1.21	0.70, 2.11	1.15	0.66, 2.00

For peer review only

2	35.2	343 543	69	0.97	0.62, 1.52	0.98	0.62, 1.54
3	24.8	242 317	60	1.17	0.74, 1.85	1.16	0.73, 1.84
>3	12.5	121 548	31	1.17	0.70, 1.96	1.03	0.61, 1.74
PCOS/ovarian cysts							
No	98.3	958 600	205	1.0	NA	1.0	NA
Yes	1.7	15 851	6	1.77	0.79, 3.99	1.73	0.77, 3.91
Endometriosis							
No	98.5	950 101	208	1.0	NA	1.0	NA
Yes	1.5	14 350	3	1.15	0.37, 3.61	1.10	0.35, 3.46
Oral contraception							
Never used	21.6	210 051	71	1.0	NA	1.0	NA
Ever used	78.4	764 401	140	0.74	0.55, 1.00	0.79	0.58, 1.06
Years of oral contraception use							
0	21.6	210 051	71	1.0	NA	1.0	NA
1	10.9	106 163	19	0.65	0.39, 1.10	0.67	0.40, 1.12
2-4	18.9	184 203	48	0.94	0.65, 1.36	0.97	0.67, 1.40
5-9	17.2	167 797	32	0.77	0.50, 1.17	0.82	0.53, 1.26
10-15	16.3	159 341	25	0.67	0.42, 1.07	0.73	0.45, 1.17
>15	15.1	146 894	16	0.50	0.29, 0.88	0.56	0.32, 0.97
HRT							
Never used	65.7	639 250	106	1.0	NA	1.0	NA
Ever used	34.3	335 202	105	1.25	0.94, 1.67	1.22	0.92, 1.62

For peer review only - http://bmjopen.bmj.com/site/about/guidelines.xhtml

For peer review only

Years of HRT							
0	65.7	639 250	106	1.0	NA	1.0	NA
1-2	9.6	93 783	39	1.79	1.23, 2.61	1.74	1.19, 2.53
3-5	7.6	74 345	17	0.99	0.59, 1.66	0.91	0.54, 1.53
6-10	5.3	51 998	15	1.16	0.67, 2.01	1.15	0.67, 1.99
>10	11.8	73 060	34	1.00	0.61, 1.64	1.02	0.61, 1.91
Gynaecological surgery							
No	82.2	801 428	136	1.0	NA	1.0	NA
Hysterectomy	9.9	96 682	40	1.92	1.34, 2.76	1.66	1.15, 2.39
Bilateral oophorectomy	0.4	3385	1	1.49	0.21, 10.63	1.47	0.21, 10.50
Both	7.5	72 956	34	2.12	1.45, 3.12	1.85	1.26, 2.72

COPD, chronic obstructive pulmonary disease; CI, confidence interval; PCOS, polycystic ovary syndrome; HRT, hormone replacement

[a]Multiple-adjusted for age, height, BMI (log-transformed), ethnicity, education, household income, Townsend deprivation index, maternal COPD and paternal COPD

[b]Age at menopause analysed only among women who experienced natural menopause before baseline (n=92 561)

For peer review only

For peer review only - http://bmjopen.bmj.com/site/about/guidelines.xhtml

Table S6. Cox regression analyses of female reproductive health indicators with incident COPD-related hospitalisation/death during follow-up, **further adjusting for comorbidities** (N=271 271)

Reproductive health indicator	% of participants	Person-years of follow-up	Number of COPD events	Multiple-adjusted[a]		Comorbidity-adjusted[c]	
				Hazard ratio	95% CI	Hazard ratio	95% CI
Age at menarche							
<12 years	20.0	326 027	275	1.15	1.00, 1.32	1.17	1.02, 1.35
12-15 years	74.1	1 210 146	768	1.0	NA	1.0	NA
>15 years	5.9	95 771	95	1.37	1.11, 1.71	1.32	1.06, 1.64
Menopause status							
No	26.6	437 890	97	1.0	NA	1.0	NA
Yes	73.4	1 194 055	1041	1.07	0.82, 1.41	1.06	0.81, 1.39
Age at natural menopause[d]							
<47 years	20.3	202 811	270	1.44	1.19, 1.75	1.46	1.21, 1.78
47-49 years	15.8	158 718	140	1.25	1.00, 1.57	1.26	1.01, 1.58
50-52 years	35.4	354 774	219	1.0	NA	1.0	NA
>52 years	28.4	283 721	161	0.93	0.75, 1.15	0.91	0.74, 1.13
Parity, binary							
Nulliparous	15.1	244 938	116	0.92	0.76, 1.12	0.90	0.74, 1.09
Parous	84.9	1 387 009	1022	1.0	NA	1.0	NA
Parity, ordered categories							
0	15.1	244 938	116	1.0	NA	1.0	NA
1	12.2	198 124	139	1.06	0.82, 1.35	1.10	0.86, 1.41

For peer review only - http://bmjopen.bmj.com/site/about/guidelines.xhtml

2	34.4	562 339	308	0.92	0.74, 1.14	0.93	0.75, 1.16
3	25.3	413 347	293	1.06	0.85, 1.33	1.10	0.88, 1.37
>3	13.1	213 195	282	1.45	1.16, 1.82	1.50	1.20, 1.87
PCOS/ovarian cysts							
No	98.3	1 604 641	1108	1.0	NA	1.0	NA
Yes	1.7	27 304	30	1.61	1.12, 2.32	1.64	1.14, 2.36
Endometriosis							
No	98.5	1 607 817	1127	1.0	NA	1.0	NA
Yes	1.5	24 131	11	0.85	0.47, 1.53	0.84	0.46, 1.53
Oral contraception							
Never used	18.9	308 641	295	1.0	NA	1.0	NA
Ever used	81.1	1 323 303	843	0.85	0.74, 0.97	0.91	0.79, 1.04
Years of oral contraception use							
0	18.9	308 641	295	1.0	NA	1.0	NA
1	11.1	180 195	137	0.84	0.69, 1.04	0.89	0.73, 1.10
2-4	18.7	306 053	246	0.95	0.80, 1.13	1.01	0.85, 1.20
5-9	17.8	290 554	181	0.86	0.71, 1.05	0.93	0.76, 1.12
10-15	17.5	285 766	153	0.74	0.61, 0.91	0.81	0.66, 0.99
>15	16.1	260 734	126	0.75	0.60, 0.93	0.81	0.65, 1.01
HRT							
Never used	63.9	1 042 083	510	1.0	NA	1.0	NA
Ever used	36.1	589 861	628	1.15	1.01, 1.30	1.20	1.05, 1.35

For peer review only - http://bmjopen.bmj.com/site/about/guidelines.xhtml

For peer review only

Years of HRT							
0	63.9	1 042 083	510	1.0	NA	1.0	NA
1-2	11.7	190 902	240	1.33	1.12, 1.57	1.38	1.16, 1.63
3-5	9.1	147 962	162	1.17	0.97, 1.42	1.22	1.01, 1.48
6-10	6.2	100 921	74	0.87	0.66, 1.14	0.90	0.69, 1.17
>10	9.2	150 077	152	1.06	0.87, 1.29	1.11	0.91, 1.35
Gynaecological surgery							
No	81.8	1 335 512	765	1.0	NA	1.0	NA
Hysterectomy	10.1	164 869	213	1.49	1.28, 1.74	1.47	1.26, 1.72
Bilateral oophorectomy	0.4	5957	6	1.36	0.61, 3.04	1.34	0.60, 2.99
Both	7.8	125 607	154	1.42	1.19, 1.69	1.39	1.17, 1.66

COPD, chronic obstructive pulmonary disease; CI, confidence interval; PCOS, polycystic ovary syndrome; HRT, hormone replacement

[a]Multiple-adjusted for age, height, BMI (log-transformed), ethnicity, education, household income, Townsend deprivation index, smoking history in pack years, maternal COPD and paternal COPD

[b]Further adjusted from the multiple-adjusted analyses[a] for a history of cardiovascular disease and/or diabetes at baseline

[c]Analysed in women who experienced natural menopause before baseline (n=159 571)

For peer review only

Table S7. Linear regression analyses of female reproductive health indicators with baseline spirometry measures, **further adjusting for smoking status or smoking duration and intensity** (N=273 441)

Reproductive health indicator	Mean change in FEV_1 z-score (95% CI)		Mean change in FVC z-score (95% CI)		Mean change in FEV_1/FVC z-score (95% CI)	
	Smoking duration and intensity-adjusted[a]	Smoking status-adjusted[b]	Smoking duration and intensity-adjusted[a]	Smoking status-adjusted[b]	Smoking duration and intensity-adjusted[a]	Smoking status-adjusted[b]
Age at menarche						
<12 years	-0.01 (-0.02, 0.00)	-0.01 (-0.02, 0.00)	-0.01 (-0.02, 0.01)	-0.01 (-0.02, 0.01)	-0.02 (-0.03, -0.01)	-0.01 (-0.03, 0.00)
12-15 years	0	0	0	0	0	0
>15 years	-0.03 (-0.05, -0.01)	-0.03 (-0.05, -0.01)	-0.03 (-0.05, -0.02)	-0.03 (-0.05, -0.01)	0.00 (-0.02, 0.02)	0.00 (-0.02, 0.02)
Menopause status						
No	0	0	0	0	0	0
Yes	-0.04 (-0.05, -0.02)	-0.03 (-0.05, -0.02)	-0.04 (-0.05, -0.02)	-0.04 (-0.05, -0.02)	0.00 (-0.02, 0.01)	0.01 (0.00, 0.03)
Age at natural menopause[c]						
<47 years	-0.04 (-0.05, -0.02)	-0.03 (-0.05, -0.02)	-0.04 (-0.05, -0.02)	-0.04 (-0.05, -0.02)	-0.01 (-0.03, 0.00)	0.00 (-0.02, 0.01)
47-49 years	-0.02 (-0.04, 0.00)	-0.02 (-0.04, 0.00)	-0.02 (-0.04, 0.00)	-0.02 (-0.03, 0.00)	-0.01 (-0.03, 0.01)	-0.01 (-0.03, 0.01)
50-52 years	0	0	0	0	0	0
>52 years	0.00 (-0.01, 0.02)	0.00 (-0.01, 0.02)	0.00 (-0.02, 0.01)	0.00 (-0.02, 0.01)	0.01 (0.00, 0.03)	0.01 (-0.01, 0.02)
Parity, binary						
Nulliparous	-0.04 (-0.06, -0.03)	-0.04 (-0.06, -0.03)	-0.07 (-0.08, -0.05)	-0.07 (-0.08, -0.06)	0.04 (0.03, 0.05)	0.04 (0.03, 0.05)
Parous	0	0	0	0	0	0

For peer review only - http://bmjopen.bmj.com/site/about/guidelines.xhtml

Parity, ordered categories	0	0	0	0	0	0
0	0.01 (-0.01, 0.03)	0.01 (0.00, 0.03)	0.02 (0.01, 0.04)	0.03 (0.01, 0.04)	-0.02 (-0.04, -0.01)	-0.02 (-0.04, 0.00)
1	0.05 (0.03, 0.06)	0.04 (0.03, 0.06)	0.07 (0.06, 0.08)	0.07 (0.06, 0.08)	-0.04 (-0.06, -0.03)	-0.04 (-0.06, -0.03)
2	0.05 (0.04, 0.07)	0.05 (0.04, 0.07)	0.08 (0.07, 0.09)	0.08 (0.06, 0.09)	-0.04 (-0.06, -0.03)	-0.04 (-0.06, -0.03)
3	0.05 (0.04, 0.07)	0.06 (0.04, 0.07)	0.08 (0.07, 0.10)	0.09 (0.07, 0.10)	-0.06 (-0.08, -0.04)	-0.05 (-0.07, -0.03)
>3						
PCOS/ovarian cysts						
No	0	0	0	0	0	0
Yes	-0.01 (-0.04, 0.03)	-0.01 (-0.04, 0.02)	-0.03 (-0.07, 0.00)	-0.04 (-0.07, 0.00)	0.05 (0.02, 0.08)	0.06 (0.03, 0.09)
Endometriosis						
No	0	0	0	0	0	0
Yes	0.01 (-0.02, 0.05)	0.01 (-0.03, 0.04)	-0.02 (-0.05, 0.01)	-0.03 (-0.06, 0.01)	0.07 (0.03, 0.10)	0.07 (0.03, 0.10)
Oral contraception						
Never used	0	0	0	0	0	0
Ever used	0.02 (0.01, 0.03)	0.02 (0.01, 0.04)	0.02 (0.01, 0.03)	0.02 (0.01, 0.03)	0.01 (0.00, 0.02)	0.01 (0.00, 0.03)
Years of oral contraception						
0	0	0	0	0	0	0
1	0.01 (-0.01, 0.03)	0.01 (-0.01, 0.03)	0.01 (-0.01, 0.02)	0.01 (-0.01, 0.02)	0.01 (-0.01, 0.02)	0.01 (0.00, 0.03)

For peer review only - http://bmjopen.bmj.com/site/about/guidelines.xhtml

For peer review only

2-4	0.02 (0.01, 0.03)	0.02 (0.01, 0.04)	0.02 (0.00, 0.03)	0.02 (0.00, 0.03)	0.01 (0.00, 0.02)	0.01 (0.00, 0.03)
5-9	0.03 (0.01, 0.04)	0.03 (0.01, 0.04)	0.03 (0.01, 0.04)	0.02 (0.01, 0.04)	0.01 (-0.01, 0.02)	0.02 (0.00, 0.03)
10-15	0.03 (0.02, 0.05)	0.04 (0.02, 0.05)	0.03 (0.02, 0.05)	0.03 (0.02, 0.05)	0.00 (-0.01, 0.02)	0.01 (0.00, 0.03)
>15	0.02 (0.00, 0.03)	0.02 (0.01, 0.04)	0.02 (0.00, 0.03)	0.02 (0.00, 0.03)	0.00 (-0.01, 0.02)	0.01 (0.00, 0.03)
HRT						
Never used	0	0	0	0	0	0
Ever used	0.00 (-0.01, 0.01)	0.00 (-0.01, 0.01)	0.00 (-0.01, 0.01)	0.00 (-0.01, 0.01)	0.01 (0.00, 0.02)	0.02 (0.01, 0.03)
Years of HRT						
0	0	0	0	0	0	0
1-2	0.00 (-0.02, 0.01)	0.00 (-0.01, 0.02)	0.00 (-0.02, 0.01)	0.00 (-0.02, 0.01)	0.01 (0.00, 0.03)	0.02 (0.01, 0.04)
3-5	0.00 (-0.02, 0.02)	0.01 (-0.01, 0.02)	0.00 (-0.02, 0.01)	0.00 (-0.01, 0.02)	0.01 (-0.01, 0.02)	0.02 (0.00, 0.03)
6-10	0.00 (-0.02, 0.02)	0.01 (-0.01, 0.02)	0.00 (-0.02, 0.02)	0.00 (-0.02, 0.02)	0.01 (-0.01, 0.03)	0.02 (0.00, 0.04)
>10	-0.01 (-0.03, 0.01)	0.00 (-0.01, 0.02)	0.00 (-0.02, 0.02)	0.00 (-0.01, 0.02)	0.00 (-0.02, 0.02)	0.01 (0.01, 0.03)
Gynaecological surgery						
No	0	0	0	0	0	0
Hysterectomy	0.01 (0.00, 0.03)	0.02 (0.00, 0.03)	0.00 (-0.02, 0.01)	0.00 (-0.01, 0.01)	0.04 (0.02, 0.05)	0.04 (0.03, 0.06)
Bilateral oophorectomy	-0.02 (-0.09, 0.05)	-0.02 (-0.09, 0.05)	-0.04 (-0.11, 0.03)	-0.05 (-0.11, 0.03)	0.05 (-0.02, 0.12)	0.04 (-0.04, 0.12)
Both	0.00 (-0.01, 0.02)	0.00 (-0.01, 0.02)	-0.02 (-0.04, -0.01)	-0.02 (-0.04, 0.00)	0.05 (0.04, 0.07)	0.05 (0.04, 0.07)

FEV1, forced expiratory volume in one-second; FVC, forced vital capacity; CI, confidence interval; PCOS, polycystic ovary syndrome; HRT, hormone replacement therapy

For peer review only - http://bmjopen.bmj.com/site/about/guidelines.xhtml

For peer review only

[a]Adjusted for age, BMI (log-transformed), ethnicity, education, household income, Townsend deprivation index, smoking history duration, smoking history intensity, asthma, maternal COPD and paternal COPD

[b]Multiple-adjusted as above with an additional covariate of smoking status and adjusting for smoking history as one covariate (pack-years) rather than two covariates (smoking duration and smoking intensity)

[c]Analysed in women who experienced natural menopause before baseline (n=161 069)

For peer review only

For peer review only - http://bmjopen.bmj.com/site/about/guidelines.xhtml

Table S8. Linear regression analyses of female reproductive health indicators with baseline spirometry measures, **further adjusting for comorbidities** (N=273 441)

Reproductive health indicator	Mean change in FEV_1 z-score (95% CI)		Mean change in FVC z-score (95% CI)		Mean change in FEV_1/FVC z-score (95% CI)	
	Multiple-adjusted[a]	Further adjusted for comorbidities[b]	Multiple-adjusted[a]	Further adjusted for comorbidities[b]	Multiple-adjusted[a]	Further adjusted for comorbidities[b]
Age at menarche						
<12 years	-0.01 (-0.02, 0.00)	-0.01 (-0.02, 0.00)	-0.01 (-0.02, -0.01)	-0.01 (-0.02, 0.01)	-0.02 (-0.03, -0.01)	-0.02 (-0.03, -0.01)
12-15 years	0	0	0	0	0	0
>15 years	-0.03 (-0.05, -0.01)	-0.03 (-0.05, -0.01)	-0.03 (-0.05, -0.02)	-0.03 (-0.05, -0.02)	0.00 (-0.02, 0.02)	0.00 (-0.02, 0.02)
Menopause status						
No	0	0	0	0	0	0
Yes	-0.04 (-0.05, -0.02)	-0.04 (-0.06, -0.03)	-0.04 (-0.06, -0.03)	-0.04 (-0.05, -0.02)	0.01 (-0.01, 0.02)	0.00 (-0.02, 0.01)
Age at natural menopause[c]						
<47 years	-0.04 (-0.05, -0.02)	-0.03 (-0.05, -0.02)	-0.04 (-0.05, -0.02)	-0.04 (-0.05, -0.02)	-0.01 (-0.02, 0.01)	-0.01 (-0.02, 0.01)
47-49 years	-0.02 (-0.04, 0.00)	-0.02 (-0.04, 0.00)	-0.02 (-0.03, 0.00)	-0.02 (-0.03, 0.00)	-0.01 (-0.03, 0.01)	-0.01 (-0.03, 0.01)
50-52 years	0	0	0	0	0	0
>52 years	0.00 (-0.01, 0.02)	0.00 (-0.01, 0.02)	0.00 (-0.02, 0.01)	0.00 (-0.02, 0.01)	0.01 (0.00, 0.02)	0.01 (0.00, 0.02)
Parity, binary						
Nulliparous	-0.04 (-0.06, -0.03)	-0.04 (-0.06, -0.03)	-0.07 (-0.08, -0.05)	-0.07 (-0.08, -0.05)	0.04 (0.03, 0.05)	0.04 (0.03, 0.05)
Parous	0	0	0	0	0	0
Parity, ordered categories						
0	0	0	0	0	0	0

For peer review only - http://bmjopen.bmj.com/site/about/guidelines.xhtml

1	0.01 (0.00, 0.03)	0.01 (0.00, 0.03)	0.02 (0.01, 0.04)	0.02 (0.01, 0.04)	-0.02 (-0.04, 0.00)	-0.02 (-0.04, 0.00)
2	0.04 (0.03, 0.06)	0.04 (0.03, 0.06)	0.07 (0.06, 0.08)	0.07 (0.06, 0.08)	-0.04 (-0.06, -0.03)	-0.04 (-0.06, -0.03)
3	0.05 (0.04, 0.07)	0.05 (0.04, 0.07)	0.08 (0.06, 0.09)	0.08 (0.06, 0.09)	-0.04 (-0.06, -0.03)	-0.04 (-0.06, -0.03)
>3	0.05 (0.04, 0.07)	0.05 (0.04, 0.07)	0.08 (0.07, 0.10)	0.08 (0.07, 0.10)	-0.06 (-0.07, -0.04)	-0.06 (-0.07, -0.04)
PCOS/ovarian cysts						
No	0	0	0	0	0	0
Yes	-0.01 (-0.04, 0.03)	-0.01 (-0.04, 0.03)	-0.03 (-0.07, 0.00)	-0.03 (-0.07, 0.00)	0.05 (0.02, 0.09)	0.05 (0.02, 0.09)
Endometriosis						
No	0	0	0	0	0	0
Yes	0.01 (-0.02, 0.05)	0.01 (-0.02, 0.05)	-0.02 (-0.05, 0.01)	-0.02 (-0.05, 0.01)	0.07 (0.03, 0.10)	0.07 (0.03, 0.10)
Oral contraception						
Never used	0	0	0	0	0	0
Ever used	0.03 (0.01, 0.04)	0.03 (0.01, 0.04)	0.02 (0.01, 0.03)	0.02 (0.01, 0.03)	0.01 (0.003, 0.03)	0.01 (0.00, 0.03)
Years of oral contraception						
0	0	0	0	0	0	0
1	0.01 (-0.01, 0.03)	0.01 (0.00, 0.03)	0.01 (-0.01, 0.03)	0.01 (-0.01, 0.03)	0.01 (0.00, 0.03)	0.01 (0.00, 0.03)
2-4	0.02 (0.01, 0.04)	0.02 (0.01, 0.04)	0.02 (0.01, 0.03)	0.02 (0.01, 0.03)	0.02 (0.00, 0.03)	0.02 (0.00, 0.03)
5-9	0.03 (0.02, 0.05)	0.03 (0.02, 0.05)	0.03 (0.01, 0.04)	0.03 (0.01, 0.04)	0.02 (0.00, 0.03)	0.02 (0.00, 0.03)
10-15	0.04 (0.02, 0.05)	0.04 (0.02, 0.05)	0.04 (0.02, 0.05)	0.03 (0.02, 0.05)	0.01 (0.00, 0.03)	0.01 (0.00, 0.03)
>15	0.02 (0.01, 0.04)	0.02 (0.01, 0.04)	0.02 (0.00, 0.03)	0.02 (0.00, 0.03)	0.01 (0.00, 0.03)	0.01 (0.00, 0.03)
HRT						
Never used	0	0	0	0	0	0

For peer review only

Ever used	0.00 (0.00, 0.01)	0.00 (-0.01, 0.01)	0.00 (-0.01, 0.01)	0.00 (-0.01, 0.01)	0.02 (0.01, 0.03)	0.02 (0.01, 0.03)
Years of HRT						
0	0	0	0	0	0	0
1-2	0.00 (-0.01, 0.02)	0.01 (-0.01, 0.02)	0.00 (-0.02, 0.01)	0.00 (-0.02, 0.01)	0.02 (0.01, 0.04)	0.02 (0.00, 0.04)
3-5	0.00 (-0.01, 0.02)	0.00 (-0.01, 0.02)	0.00 (-0.02, 0.02)	0.00 (-0.01, 0.02)	0.02 (0.00, 0.03)	0.01 (0.00, 0.03)
6-10	0.01 (-0.01, 0.02)	0.01 (-0.01, 0.03)	0.00 (-0.02, 0.02)	0.00 (-0.02, 0.02)	0.02 (0.00, 0.04)	0.02 (0.00, 0.04)
>10	0.00 (-0.01, 0.02)	0.00 (-0.01, 0.02)	0.00 (-0.01, 0.02)	0.00 (-0.01, 0.02)	0.01 (-0.01, 0.03)	0.01 (-0.01, 0.03)
Gynaecological surgery						
No	0	0	0	0	0	0
Hysterectomy	0.01 (0.00, 0.03)	0.02 (0.00, 0.03)	0.00 (-0.02, 0.01)	0.00 (-0.01, 0.01)	0.04 (0.02, 0.06)	0.04 (0.02, 0.06)
Bilateral oophorectomy	-0.02 (-0.08, 0.05)	-0.01 (-0.08, 0.05)	-0.04 (-0.11, 0.03)	-0.04 (-0.11, 0.03)	0.05 (-0.02, 0.12)	0.05 (-0.02, 0.12)
Both	0.00 (-0.01, 0.02)	0.01 (-0.01, 0.02)	-0.02 (-0.04, -0.01)	-0.02 (-0.03, 0.00)	0.05 (0.04, 0.07)	0.05 (0.04, 0.07)

FEV1, forced expiratory volume in one-second; FVC, forced vital capacity; CI, confidence interval; PCOS, polycystic ovary syndrome; HRT, hormone replacement therapy

[a]Adjusted for age, BMI (log-transformed), ethnicity, education, household income, Townsend deprivation index, smoking history in pack-years, asthma, maternal COPD and paternal COPD

[b]Further adjusted from the multiple-adjusted analyses[a] for a history of cardiovascular disease and/or diabetes at baseline

[c]Analysed in women who experienced natural menopause before baseline (n=161 069)

For peer review only - http://bmjopen.bmj.com/site/about/guidelines.xhtml

Table S9. Cox regression analyses of female reproductive health indicators with incident COPD-related **death** during follow-up (N=273 441)

Reproductive health indicator	% of participants	Person-years of follow-up	Number of COPD deaths	Age-adjusted		Multiple-adjusted[a]	
				Hazard ratio	95% CI	Hazard ratio	95% CI
Age at menarche							
<12 years	20.0	332 700	51	1.62	1.15, 2.27	1.52	1.08, 2.13
12-15 years	74.1	1 229 881	115	1.0	NA	1.0	NA
>15 years	5.9	97 672	13	1.49	0.84, 2.65	1.18	0.66, 2.11
Menopausal status							
Pre	26.4	461 595	6	1.0	NA	1.0	NA
Post	73.6	1 198 658	173	2.03	0.76	1.39	0.54, 3.63
Age at natural menopause[b]							
<47 years	20.7	200 666	47	2.47	1.54, 3.97	1.49	0.92, 2.42
47-49 years	12.8	154 965	28	1.92	1.10, 3.34	1.49	0.85, 2.61
50-52 years	23.7	345 140	35	1.0	NA	1.0	NA
>52 years	16.3	275 067	28	0.87	0.51, 1.48	0.96	0.56, 1.64
Parity, binary							
Nulliparous	15.0	248 399	18	0.83	0.51, 1.35	1.04	0.63, 1.70
Parous	85.0	1 411 854	161	1.0	NA	1.0	NA
Parity, ordered categories							
0	15.0	248 399	18	1.0	NA	1.0	NA
1	12.2	201 277	28	1.79	0.99, 3.23	1.24	0.69, 2.26
2	34.4	571 145	44	0.82	0.47, 1.42	0.77	0.45, 1.35

For peer review only - http://bmjopen.bmj.com/site/about/guidelines.xhtml

3	25.3	420 337	41	1.03	0.59, 1.80	0.86	0.49, 1.50
>3	13.2	219 093	48	2.07	1.20, 3.58	1.22	0.70, 2.13
PCOS/ovarian cysts							
No	98.3	1 632 388	175	1.0	NA	1.0	NA
Yes	1.7	27 861	4	1.45	0.54, 3.91	1.52	0.56, 4.11
Endometriosis							
No	98.5	1 635 728	178	1.0	NA	1.0	NA
Yes	1.5	24 526	1	0.50	0.07, 3.60	0.61	0.08, 4.34
Oral contraception							
Never used	19.0	315 862	50	1.0	NA	1.0	NA
Ever used	81.0	1 344 391	129	1.03	0.74, 1.45	0.87	0.62, 1.22
Years of oral contraception							
0	19.0	315 862	50	1.0	NA	1.0	NA
1	11.1	183 949	26	1.30	0.80, 2.12	0.97	0.60, 1.57
2-4	18.7	311 688	33	0.92	0.59, 1.45	0.81	0.52, 1.27
5-9	17.7	294 898	22	0.83	0.50, 1.38	0.74	0.45, 1.24
10-15	17.4	289 787	25	1.04	0.64, 1.71	0.85	0.52, 1.39
>15	16.0	264 067	23	1.25	0.75, 2.09	1.05	0.63, 1.75
HRT							
Never used	63.7	1 024 004	72	1.0	NA	1.0	NA
Ever used	36.6	636 249	107	1.33	0.98, 1.81	0.99	0.73, 1.35
Years of HRT							

For peer review only - http://bmjopen.bmj.com/site/about/guidelines.xhtml

0	63.7	1 024 004	72	1.0	NA	1.0	NA
1-2	11.8	178 712	38	1.53	1.00, 2.34	1.05	0.69, 1.61
3-5	9.1	140 626	35	1.82	1.17, 2.82	1.29	0.83, 2.00
6-10	6.2	94 520	10	0.71	0.34, 1.48	0.64	0.31, 1.33
>10	9.2	222 391	24	1.00	0.60, 1.67	0.83	0.50, 1.38
Gynaecological surgery							
No	81.7	1 350 007	120	1.0	NA	1.0	NA
Hysterectomy	10.1	172 764	36	1.63	1.11, 2.38	1.37	0.94, 2.01
Bilateral oophorectomy	0.4	6130	1	1.50	0.21, 10.71	1.32	0.18, 9.48
Both	7.8	131 351	23	1.40	0.89, 2.20	1.19	0.75, 1.87

COPD, chronic obstructive pulmonary disease; CI, confidence interval; PCOS, polycystic ovary syndrome; HRT, hormone replacement

[a]Multiple-adjusted for age, height, BMI (log-transformed), ethnicity, education, household income, Townsend deprivation index, smoking history, maternal COPD and paternal COPD

[b]Age at menopause was analysed only among women who experienced natural menopause before baseline (n=161 069)

For peer review only

Table S10. Cox regression analyses of female reproductive health indicators with incident COPD-related hospitalisation/death during follow-up in **complete cases** (N=220 773)

Reproductive health indicator	% of participants[a]	Person-years of follow-up	Number of COPD events	Age-adjusted		Multiple-adjusted[b]	
				Hazard ratio	95% CI	Hazard ratio	95% CI
Age at menarche							
<12 years	19.4	257 579	222	1.37	1.17, 1.59	1.20	1.03, 1.41
12-15 years	72.1	958 578	598	1.0	NA	1.0	NA
>15 years	5.6	74 151	71	1.57	1.23, 2.01	1.34	1.05, 1.72
Menopausal status							
Pre	23.8	319 918	53	1.0	NA	1.0	NA
Post	60.4	799 833	673	1.83	1.32, 2.54	1.20	0.85, 1.68
Age at natural menopause[c]							
<47 years	32.4	174 207	224	2.12	1.75, 2.57	1.39	1.12, 1.72
47-49 years	17.9	139 602	124	1.58	1.26, 1.98	1.19	0.92, 1.53
50-52 years	28.1	314 777	194	1.0	NA	1.0	NA
>52 years	21.6	252 180	149	0.89	0.72, 1.10	0.92	0.73, 1.17
Parity, binary							
Nulliparous	15.1	200 749	98	0.81	0.65, 1.00	0.97	0.78, 1.20
Parous	83.3	1 107 959	807	1.0	NA	1.0	NA
Parity, ordered categories							
0	15.1	200 749	98	1.0	NA	1.0	NA
1	11.9	157 577	110	1.36	1.03, 1.78	1.00	0.76, 1.32

For peer review only - http://bmjopen.bmj.com/site/about/guidelines.xhtml

2	34.0	453 260	240	0.90	0.71, 1.13	0.84	0.67, 1.07
3	24.7	328 906	236	1.21	0.95, 1.53	1.03	0.81, 1.31
>3	12.7	168 215	221	2.11	1.66, 2.68	1.43	1.12, 1.83
PCOS/ovarian cysts							
No	98.3	1 307 646	895	1.0	NA	1.0	NA
Yes	1.7	22 016	22	1.47	0.97, 2.25	1.43	0.94, 2.19
Endometriosis							
No	98.5	1 310 012	907	1.0	NA	1.0	NA
Yes	1.5	19 651	10	0.89	0.48, 1.67	0.97	0.52, 1.80
Oral contraception							
Never used	19.1	253 792	222	1.0	NA	1.0	NA
Ever used	80.7	1 072 330	691	1.07	0.92, 1.25	0.94	0.80, 1.10
Years of oral contraception							
0	19.1	253 792	222	1.0	NA	1.0	NA
1	10.7	141 914	108	1.17	0.92, 1.47	0.93	0.74, 1.17
2-4	18.7	249 392	202	1.17	0.97, 1.42	1.04	0.86, 1.27
5-9	17.8	236 848	147	1.03	0.83, 1.27	0.94	0.76, 1.17
10-15	17.5	232 589	130	0.99	0.79, 1.23	0.85	0.68, 1.06
>15	16.0	211 587	104	0.96	0.75, 1.22	0.84	0.66, 1.07
HRT							
Never used	62.2	827 356	352	1.0	NA	1.0	NA
Ever used	37.5	497 994	562	1.78	1.55, 2.05	1.31	1.13, 1.51

For peer review only - http://bmjopen.bmj.com/site/about/guidelines.xhtml

Years of HRT							
0	62.2	827 356	352	1.0	NA	1.0	NA
1-2	10.5	138 603	173	2.12	1.76, 2.56	1.52	1.27, 1.83
3-5	8.3	110 741	117	1.82	1.47, 2.24	1.28	1.04, 1.58
6-10	5.6	74 932	57	1.21	0.92, 1.61	1.03	0.77, 1.36
>10	8.1	107 945	113	1.53	1.23, 1.90	1.24	1.00, 1.54
Gynaecological surgery							
No	81.0	1 077 823	603	1.0	NA	1.0	NA
Hysterectomy	9.7	129 561	163	1.75	1.47, 2.09	1.49	1.25, 1.77
Bilateral oophorectomy	0.4	4611	5	1.63	0.68, 3.94	1.45	0.60, 3.50
Both	7.5	98 833	119	1.67	1.37, 2.04	1.42	1.17, 1.74

COPD, chronic obstructive pulmonary disease; CI, confidence interval; PCOS, polycystic ovary syndrome; HRT, hormone replacement therapy; NA, not applicable

[a]Totals may not equal 100% due to missing values in this complete case analysis

[b]Multiple-adjusted for age, height, BMI (log-transformed), ethnicity, education, household income, Townsend deprivation index, smoking history in pack years, maternal COPD and paternal COPD

[c]Analysed in women who experienced natural menopause before baseline (n=129 961)

For peer review only - http://bmjopen.bmj.com/site/about/guidelines.xhtml

For peer review only

Table S11. Linear regression analyses of female reproductive health indicators with baseline spirometry measures in **complete cases** (N=160 116)

**Reproductive health indicator**	**Mean change in FEV_1 z-score (95% CI)**		**Mean change in FVC z-score (95% CI)**		**Mean change in FEV_1/FVC z-score (95% CI)**	
	**Unadjusted**	**Multiple-adjusted[a]**	**Unadjusted**	**Multiple-adjusted[a]**	**Unadjusted**	**Multiple-adjusted[a]**
Age at menarche						
<12 years	-0.05 (-0.07, -0.04)	-0.01 (-0.03, 0.00)	-0.07 (-0.08, -0.06)	0.00 (-0.02, 0.01)	0.02 (0.01, 0.03)	-0.02 (-0.03, -0.01)
12-15 years	0	0	0	0	0	0
>15 years	-0.10 (-0.13, -0.08)	-0.04 (-0.06, -0.02)	-0.09 (-0.11, -0.07)	-0.05 (-0.07, -0.03)	-0.06 (-0.08, -0.04)	0.00 (-0.02, 0.02)
Menopause status						
No	0	0	0	0	0	0
Yes	-0.04 (-0.05, -0.03)	-0.06 (-0.08, -0.04)	-0.03 (-0.05, -0.02)	-0.06 (-0.07, -0.04)	-0.02 (-0.03, -0.01)	-0.02 (-0.03, 0.00)
Age at natural menopause[b]						
<47 years	-0.15 (-0.17, -0.13)	-0.06 (-0.07, -0.04)	-0.13 (-0.15, -0.11)	-0.06 (-0.08, -0.04)	-0.07 (-0.09, -0.05)	-0.01 (-0.03, 0.01)
47-49 years	-0.07 (-0.09, -0.05)	-0.03 (-0.05, -0.01)	-0.06 (-0.08, -0.04)	-0.03 (-0.04, -0.01)	-0.04 (-0.06, -0.02)	-0.01 (-0.03, 0.01)
50-52 years	0	0	0	0	0	0
>52 years	0.01 (-0.01, 0.02)	0.00 (-0.02, 0.02)	-0.01 (-0.03, 0.00)	-0.01 (-0.03, 0.01)	0.04 (0.03, 0.06)	0.02 (0.00, 0.03)
Parity, binary						
Nulliparous	-0.03 (-0.04, -0.01)	-0.06 (-0.07, -0.04)	-0.05 (-0.07, -0.04)	-0.08 (-0.09, -0.07)	0.05 (0.03, 0.06)	0.05 (0.03, 0.06)
Parous	0	0	0	0	0	0
Parity, ordered categories						
0	0	0	0	0	0	0
1	-0.03 (-0.05, -0.02)	0.01 (0.00, 0.03)	-0.01 (-0.02, 0.01)	0.03 (0.01, 0.04)	-0.06 (-0.08, -0.04)	-0.02 (-0.04, -0.01)
2	0.06 (0.04, 0.08)	0.06 (0.05, 0.07)	0.08 (0.07, 0.10)	0.09 (0.07, 0.10)	-0.03 (-0.05, -0.02)	-0.05 (-0.06, -0.03)

For peer review only - http://bmjopen.bmj.com/site/about/guidelines.xhtml

3	0.04 (0.03, 0.06)	0.07 (0.05, 0.08)	0.07 (0.05, 0.09)	0.10 (0.08, 0.11)	-0.05 (-0.06, -0.03)	-0.05 (-0.07, -0.03)
>3	-0.04 (-0.06, -0.02)	0.06 (0.04, 0.08)	-0.01 (-0.03, 0.01)	0.10 (0.08, 0.11)	-0.07 (-0.09, -0.06)	-0.06 (-0.08, -0.04)
PCOS/ovarian cysts						
No	0	0	0	0	0	0
Yes	-0.02 (-0.06, 0.02)	0.00 (-0.04, 0.03)	-0.06 (-0.09, -0.02)	-0.03 (-0.07, 0.00)	0.07 (0.03, 0.11)	0.07 (0.03, 0.11)
Endometriosis						
No	0	0	0	0	0	0
Yes	-0.01 (-0.05, 0.04)	0.00 (-0.03, 0.04)	-0.04 (-0.08, 0.01)	-0.03 (-0.07, 0.01)	0.06 (0.02, 0.10)	0.06 (0.03, 0.10)
Oral contraception						
Never used	0	0	0	0	0	0
Ever used	0.08 (0.07, 0.09)	0.03 (0.02, 0.04)	0.09 (0.08, 0.11)	0.03 (0.02, 0.04)	-0.01 (-0.02, 0.00)	0.02 (0.01, 0.03)
Years of oral contraception						
0	0	0	0	0	0	0
1	0.04 (0.02, 0.06)	0.02 (0.00, 0.03)	0.05 (0.03, 0.07)	0.01 (-0.01, 0.03)	-0.03 (-0.05, -0.01)	0.01 (-0.01, 0.03)
2-4	0.07 (0.05, 0.08)	0.03 (0.01, 0.05)	0.07 (0.06, 0.09)	0.02 (0.01, 0.04)	0.00 (-0.01, 0.02)	0.02 (0.01, 0.04)
5-9	0.10 (0.08, 0.11)	0.04 (0.02, 0.05)	0.11 (0.09, 0.12)	0.03 (0.02, 0.05)	0.00 (-0.01, 0.02)	0.02 (0.00, 0.04)
10-15	0.10 (0.09, 0.12)	0.05 (0.03, 0.06)	0.12 (0.11, 0.14)	0.05 (0.03, 0.06)	-0.02 (-0.03, 0.00)	0.02 (0.00, 0.03)
>15	0.08 (0.07, 0.10)	0.03 (0.01, 0.04)	0.10 (0.08, 0.12)	0.02 (0.01, 0.04)	-0.03 (-0.05, -0.01)	0.01 (-0.01, 0.03)
HRT						
Never used	0	0	0	0	0	0
Ever used	-0.03 (-0.04, -0.02)	0.00 (-0.01, 0.01)	-0.01 (-0.02, 0.00)	-0.01 (-0.02, 0.00)	-0.04 (-0.05, -0.03)	0.02 (0.01, 0.03)
Years of HRT						

For peer review only - http://bmjopen.bmj.com/site/about/guidelines.xhtml

For peer review only

0	0	0	0	0	0	0
1-2	-0.03 (-0.05, -0.02)	0.00 (-0.02, 0.01)	-0.02 (-0.03, 0.00)	-0.01 (-0.03, 0.01)	-0.05 (-0.06, -0.03)	0.02 (0.00, 0.03)
3-5	-0.04 (-0.06, -0.02)	0.00 (-0.02, 0.02)	-0.03 (-0.05, -0.01)	0.00 (-0.02, 0.02)	-0.04 (-0.05, -0.02)	0.01 (-0.01, 0.03)
6-10	0.00 (-0.02, 0.02)	0.01 (-0.01, 0.03)	0.01 (-0.02, 0.03)	0.00 (-0.02, 0.02)	-0.01 (-0.03, 0.01)	0.02 (0.00, 0.04)
>10	-0.01 (-0.03, 0.01)	0.00 (-0.02, 0.02)	0.01 (-0.01, 0.03)	0.00 (-0.02, 0.02)	-0.03 (-0.05, -0.02)	0.01 (-0.01, 0.03)
Gynaecological surgery						
No	0	0	0	0	0	0
Hysterectomy	-0.04 (-0.06, -0.03)	0.02 (0.00, 0.04)	-0.06 (-0.08, -0.05)	0.00 (-0.01, 0.02)	0.03 (0.02, 0.05)	0.05 (0.03, 0.07)
Bilateral oophorectomy	-0.07 (-0.15, 0.01)	-0.05 (-0.13, 0.03)	-0.10 (-0.18, -0.01)	-0.08 (-0.16, 0.00)	0.05 (-0.03, 0.14)	0.06 (-0.02, 0.14)
Both	-0.05 (-0.07, -0.03)	0.00 (-0.02, 0.02)	-0.08 (-0.10, -0.06)	-0.03 (-0.04, -0.01)	0.06 (0.04, 0.08)	0.06 (0.04, 0.08)

FEV_1, forced expiratory volume in one-second; FVC, forced vital capacity; CI, confidence interval; PCOS, polycystic ovary syndrome; HRT, hormone replacement therapy; NA, not applicable

[a]Adjusted for age, BMI (log-transformed), ethnicity, education, household income, Townsend deprivation index, smoking history pack-years, asthma, maternal COPD and paternal COPD

[b]Analysed in women who experienced natural menopause before baseline (n=121 153)

For peer review only - http://bmjopen.bmj.com/site/about/guidelines.xhtml

STROBE 2007 (v4) Statement—Checklist of items that should be included in reports of *cohort studies*

Section/Topic	Item #	Recommendation	Reported on page #
**Title and abstract**	1	(*a*) Indicate the study's design with a commonly used term in the title or the abstract	1
		(*b*) Provide in the abstract an informative and balanced summary of what was done and what was found	2
**Introduction**			
Background/rationale	2	Explain the scientific background and rationale for the investigation being reported	4
Objectives	3	State specific objectives, including any prespecified hypotheses	4-5
**Methods**			
Study design	4	Present key elements of study design early in the paper	6-7
Setting	5	Describe the setting, locations, and relevant dates, including periods of recruitment, exposure, follow-up, and data collection	5
Participants	6	(*a*) Give the eligibility criteria, and the sources and methods of selection of participants. Describe methods of follow-up	5
		(*b*) For matched studies, give matching criteria and number of exposed and unexposed	
Variables	7	Clearly define all outcomes, exposures, predictors, potential confounders, and effect modifiers. Give diagnostic criteria, if applicable	5-7
Data sources/ measurement	8*	For each variable of interest, give sources of data and details of methods of assessment (measurement). Describe comparability of assessment methods if there is more than one group	5-7
Bias	9	Describe any efforts to address potential sources of bias	6-7
Study size	10	Explain how the study size was arrived at	8
Quantitative variables	11	Explain how quantitative variables were handled in the analyses. If applicable, describe which groupings were chosen and why	6-7
Statistical methods	12	(*a*) Describe all statistical methods, including those used to control for confounding	6-7
		(*b*) Describe any methods used to examine subgroups and interactions	7
		(*c*) Explain how missing data were addressed	7
		(*d*) If applicable, explain how loss to follow-up was addressed	7
		(*e*) Describe any sensitivity analyses	7
**Results**			

For peer review only

Participants	13*	(a) Report numbers of individuals at each stage of study—eg numbers potentially eligible, examined for eligibility, confirmed eligible, included in the study, completing follow-up, and analysed	8 and Figure 1
		(b) Give reasons for non-participation at each stage	8 and Figure 1
		(c) Consider use of a flow diagram	Figure 1
Descriptive data	14*	(a) Give characteristics of study participants (eg demographic, clinical, social) and information on exposures and potential confounders	8 and 21-24
		(b) Indicate number of participants with missing data for each variable of interest	8 and 21-24
		(c) Summarise follow-up time (eg, average and total amount)	8
Outcome data	15*	Report numbers of outcome events or summary measures over time	8 and 25-28
Main results	16	(*a*) Give unadjusted estimates and, if applicable, confounder-adjusted estimates and their precision (eg, 95% confidence interval). Make clear which confounders were adjusted for and why they were included	25-32
		(*b*) Report category boundaries when continuous variables were categorized	5
		(*c*) If relevant, consider translating estimates of relative risk into absolute risk for a meaningful time period	
Other analyses	17	Report other analyses done—eg analyses of subgroups and interactions, and sensitivity analyses	10 and online supplementary file
**Discussion**			
Key results	18	Summarise key results with reference to study objectives	10
**Limitations**			
Interpretation	20	Give a cautious overall interpretation of results considering objectives, limitations, multiplicity of analyses, results from similar studies, and other relevant evidence	11-12
Generalisability	21	Discuss the generalisability (external validity) of the study results	13-14
**Other information**			
Funding	22	Give the source of funding and the role of the funders for the present study and, if applicable, for the original study on which the present article is based	2

*Give information separately for cases and controls in case-control studies and, if applicable, for exposed and unexposed groups in cohort and cross-sectional studies.

Note: An Explanation and Elaboration article discusses each checklist item and gives methodological background and published examples of transparent reporting. The STROBE checklist is best used in conjunction with this article (freely available on the Web sites of PLoS Medicine at http://www.plosmedicine.org/, Annals of Internal Medicine at http://www.annals.org/, and Epidemiology at http://www.epidem.com/). Information on the STROBE Initiative is available at www.strobe-statement.org.

For peer review only
